# Supplementary material for: Stereoselective alkoxycarbonylation of unactivated C(sp3)–H bonds with alkyl chloroformates via Pd(II)/Pd(IV) catalysis
Source: Nat Commun. 2016 Sep 28;7:12901. doi: 10.1038/ncomms12901 (PMC5052708; doi:10.1038/ncomms12901)
Supplement: Supplementary Information — Supplementary Figures 1-67, Supplementary Tables 1-12, Supplementary Methods and Supplementary References [file ncomms12901-s1.pdf]

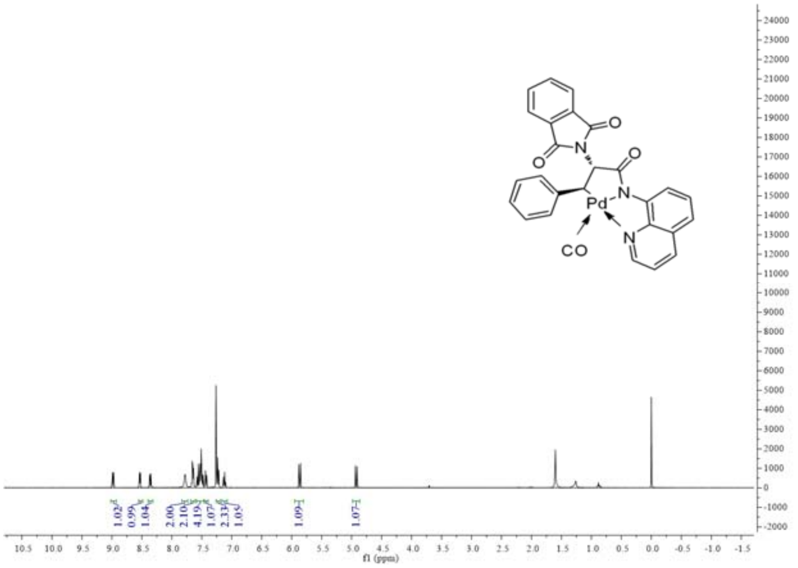

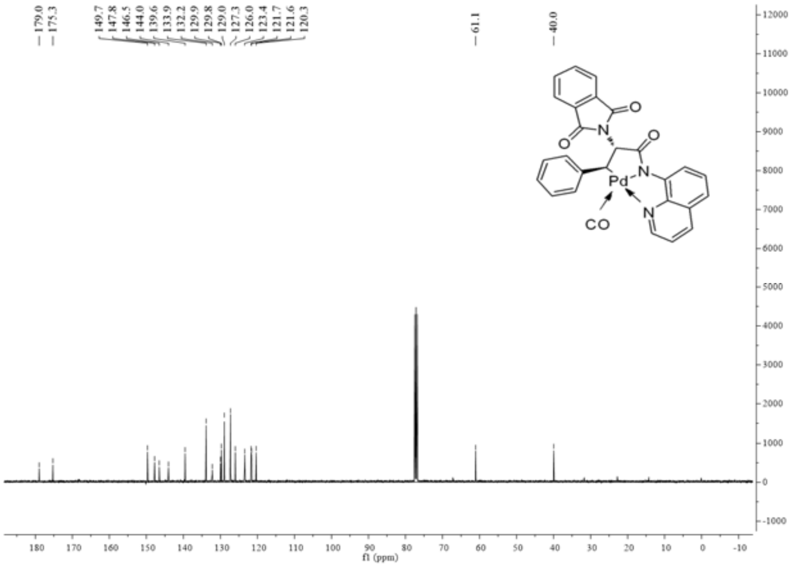

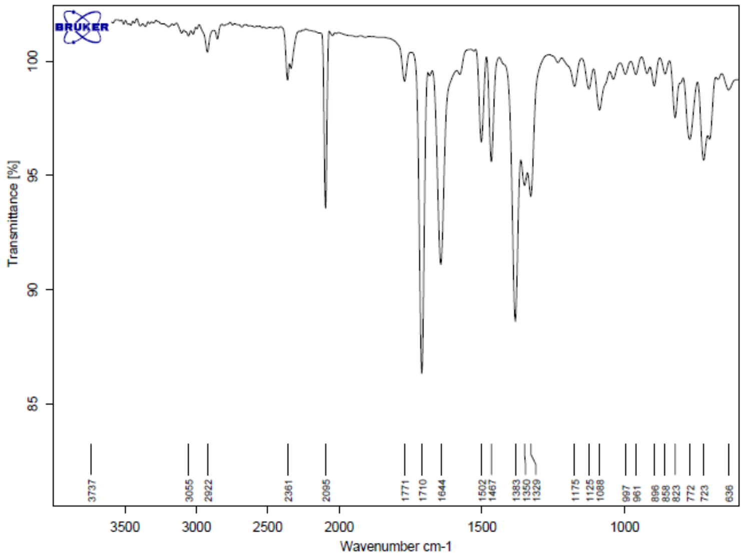

Supplementary Figure 1. ^{1}H, ^{13}C NMR and IR spectra for complex II

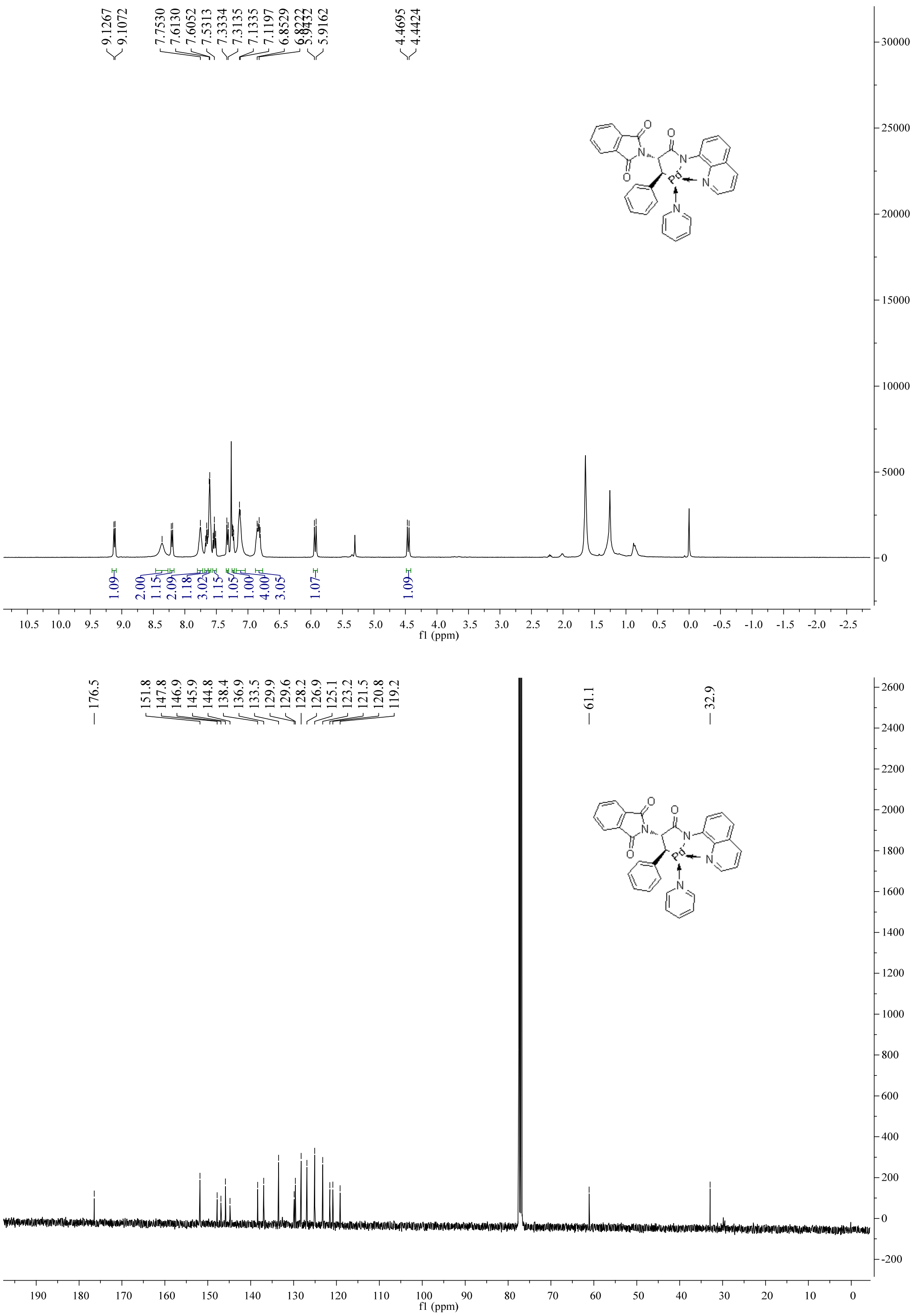

Supplementary Figure 2. ^{1}H and ^{13}C NMR spectra for III

[illegible]

AD-H , n-hex : iPrOH=55/45, 0.9 ml/min, 220 nm

分析者	: System Administrator
样品名	: LG-Phe
样品ID	:
进样体积	: 10
数据文件	: LG-Phe1.lcd
方法文件	: 13.lcm
报告格式文件	: xt.lsr
分析日期/时间	: 2016-6-17 09:56:50
处理日期/时间	: 2016-6-17 10:27:56
重复进样计数	: 1
描述	: AD-H , n-hex : iPrOH=55/45, 0.9 ml/min, 220 nm

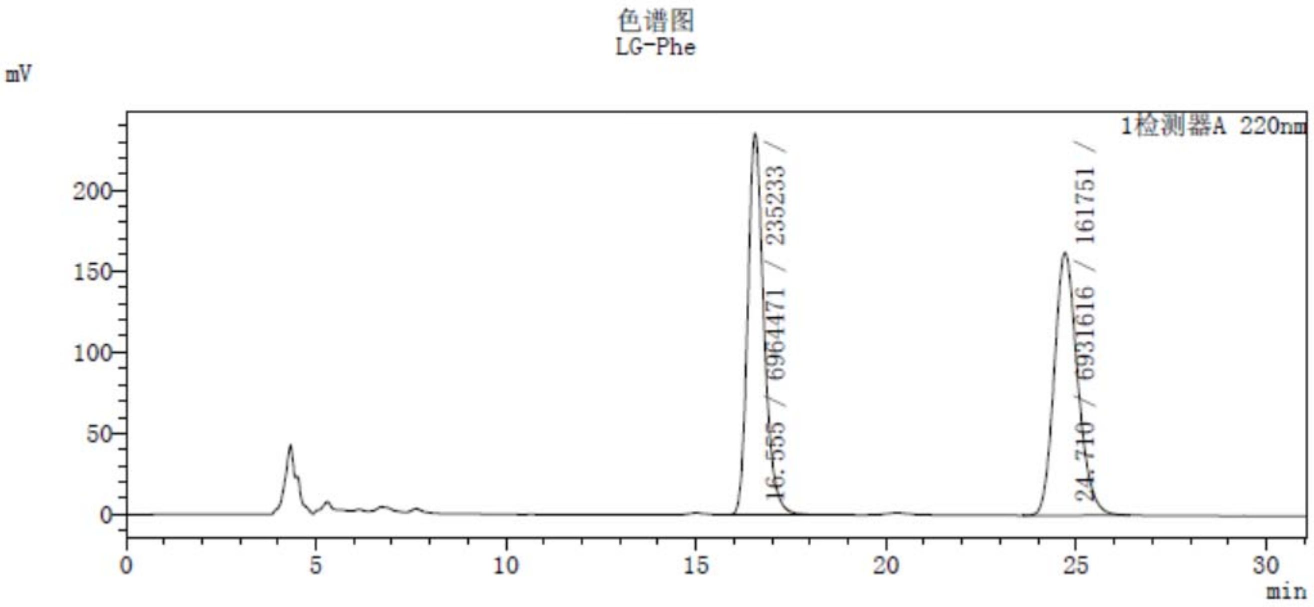

峰表

检测器A 220nm

峰号	保留时间	面积	高度	标记	面积%
1	16.555	6964471	235233	M	50.118
2	24.710	6931616	161751	M	49.882
总计		13896087	396985		100.000

Supplementary Figure 3. HPLC spectrum for racemic 1a

AD-H , n-hex : iPrOH=55/45, 0.9 ml/min, 220 nm

分析者	: System Administrator
样品名	: LGL-Phe
样品ID	:
进样体积	: 2.5
数据文件	: LGL-Phe1.lcd
方法文件	: 13.lcm
报告格式文件	: xt.lsr
分析日期/时间	: 2016-6-17 11:00:01
处理日期/时间	: 2016-6-17 11:31:19
重复进样计数	: 1
描述	: AD-H , n-hex : iPrOH=55/45, 0.9 ml/min, 220 nm

色谱图
LGL-Phe

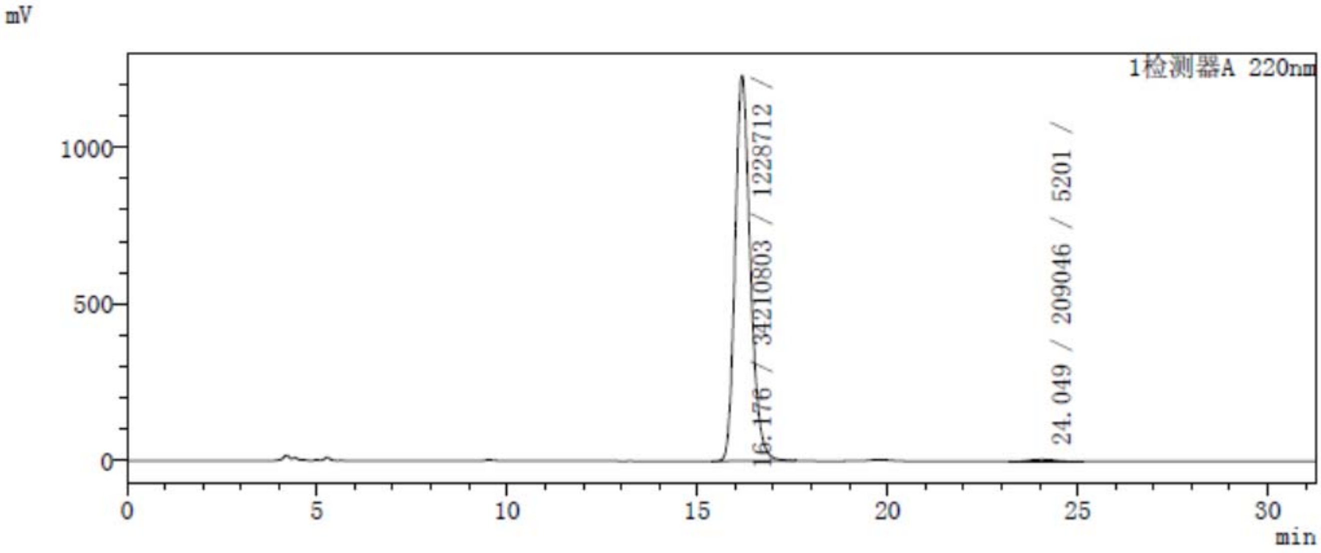

峰表

检测器A 220nm

峰号	保留时间	面积	高度	标记	面积%
1	16.176	34210803	1228712	M	99.393
2	24.049	209046	5201	M	0.607
总计		34419848	1233913		100.000

Supplementary Figure 4. HPLC spectrum for 1a

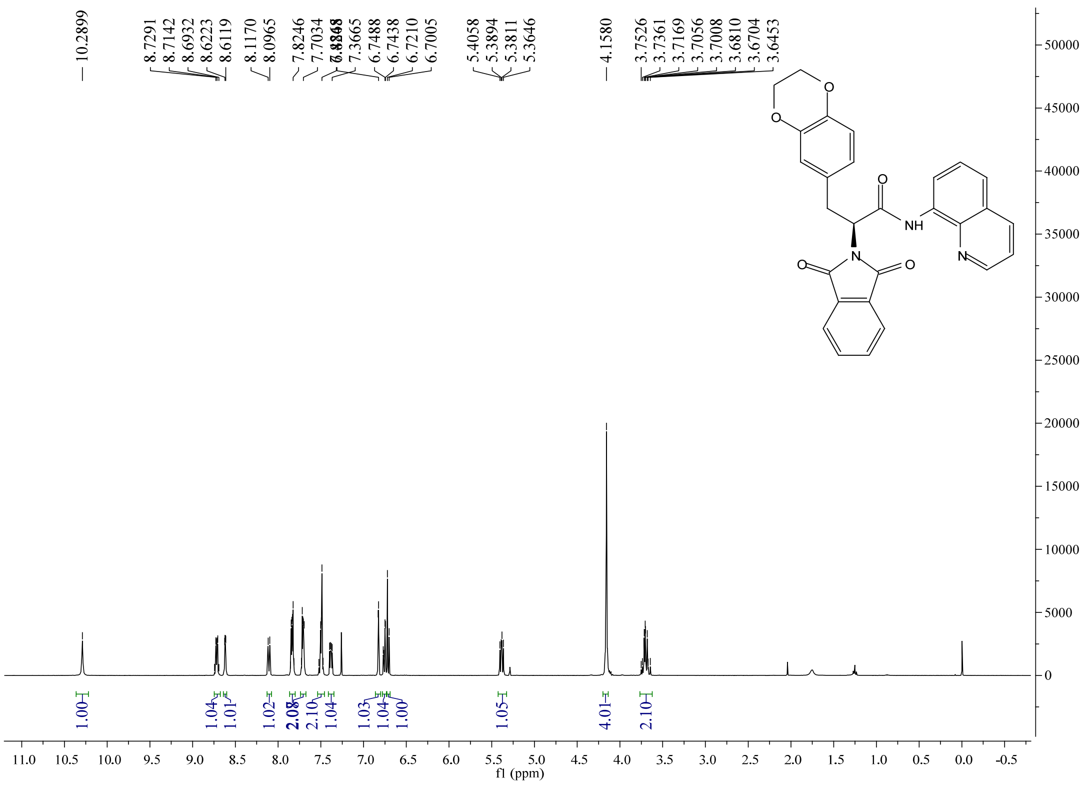

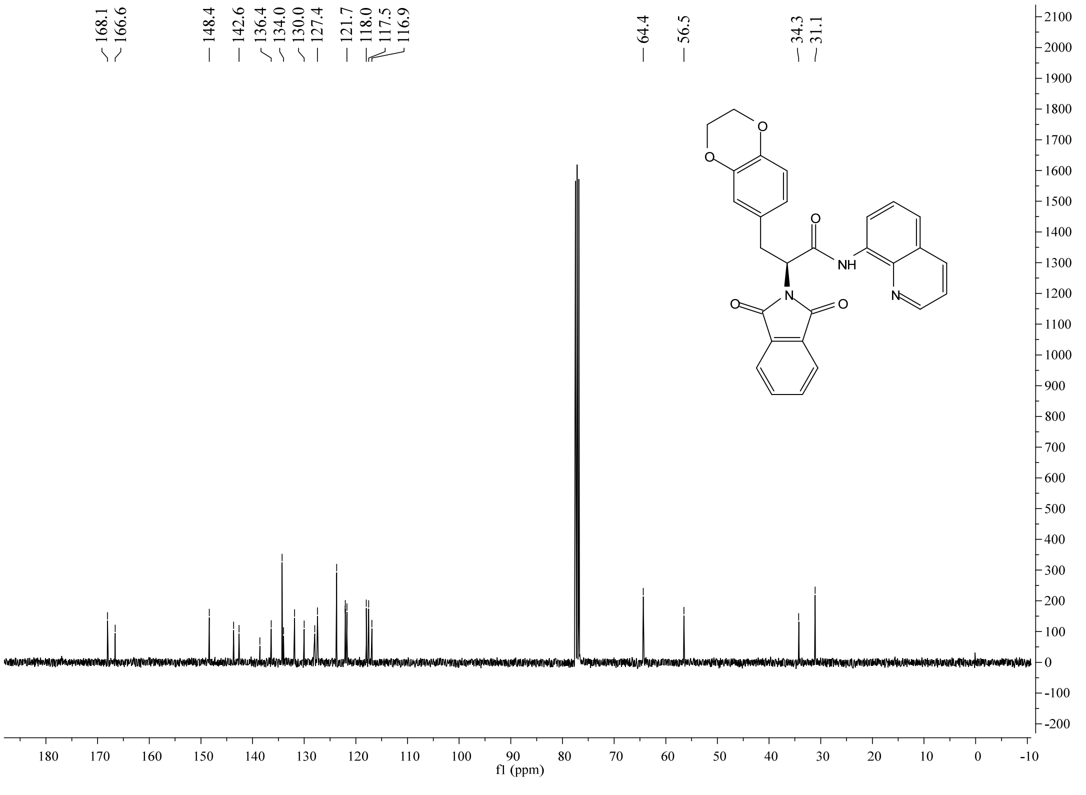

Supplementary Figure 5. ^{1}H and ^{13}C NMR spectra for 2q

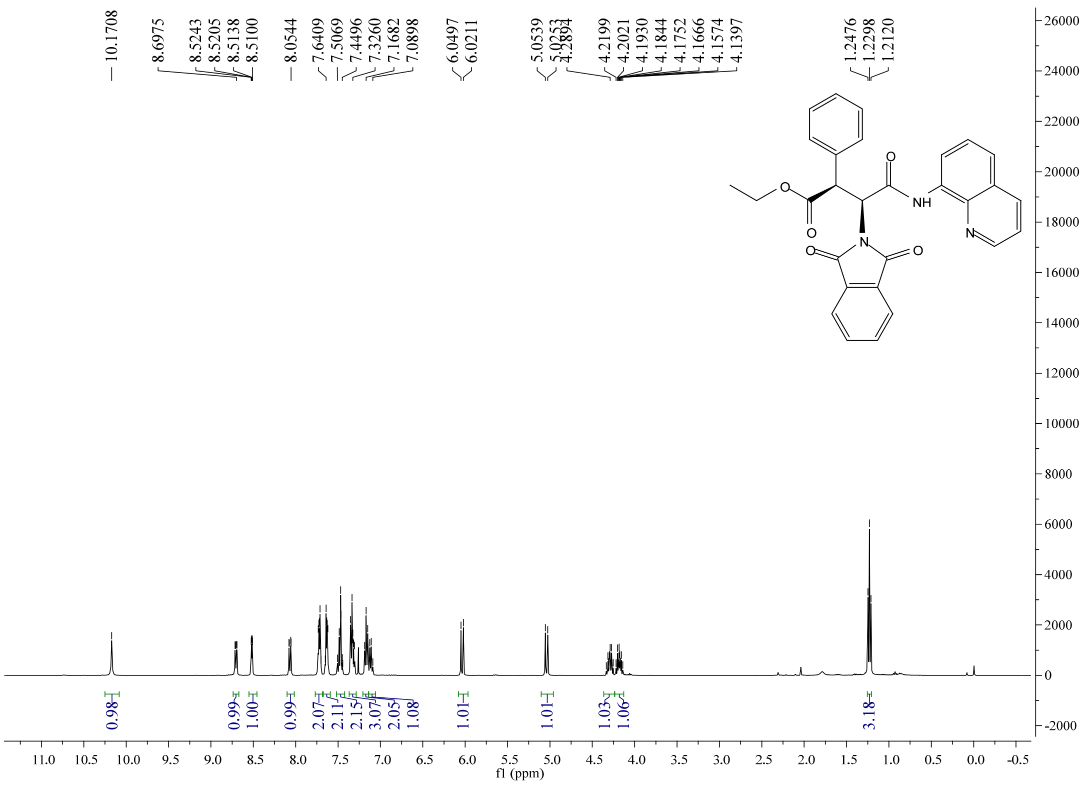

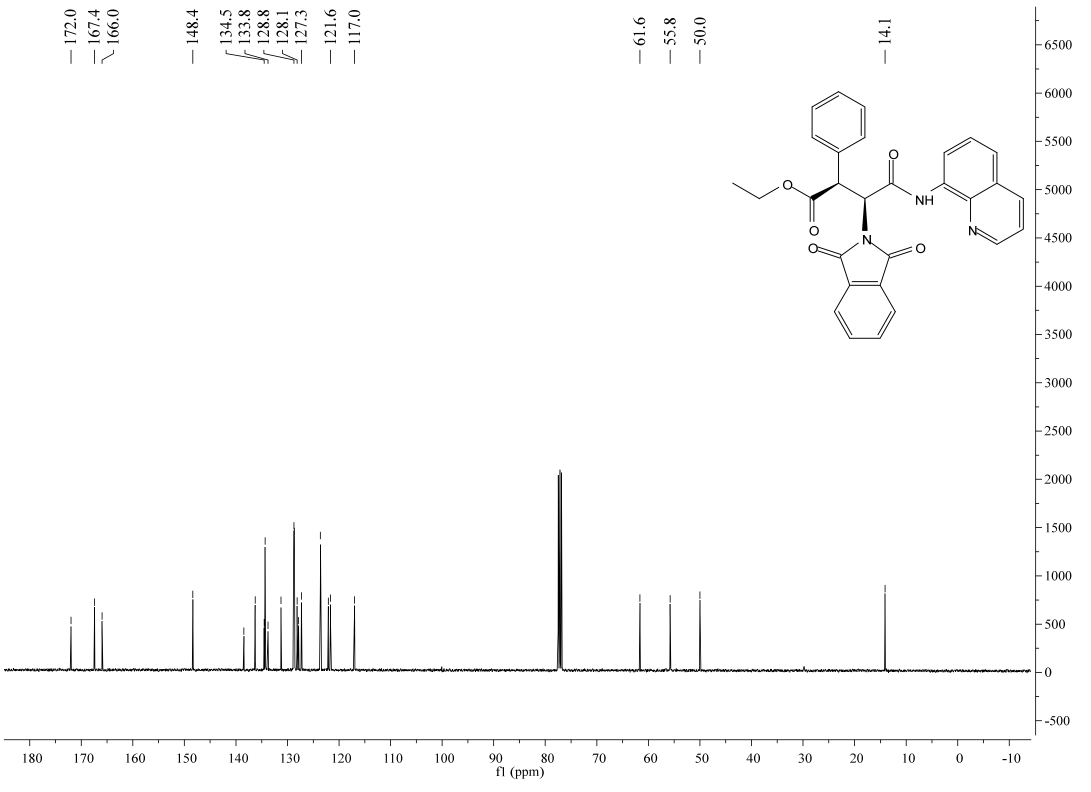

Supplementary Figure 6. ^{1}H and ^{13}C NMR spectra for 3a

AD-H , n-hex : iPrOH=55/45, 0.9 ml/min, 220 nm

分析者	: System Administrator
样品名	: DL-PHEOET
样品ID	:
进样体积	: 10
数据文件	: DL-PHEOET1.lcd
方法文件	: 13.lcm
报告格式文件	: xt.lsr
分析日期/时间	: 2016-6-16 14:43:50
处理日期/时间	: 2016-6-16 15:24:43
重复进样计数	: 1
描述	: AD-H , n-hex : iPrOH=55/45, 0.9 ml/min, 220 nm

色谱图
DL-PHEOET

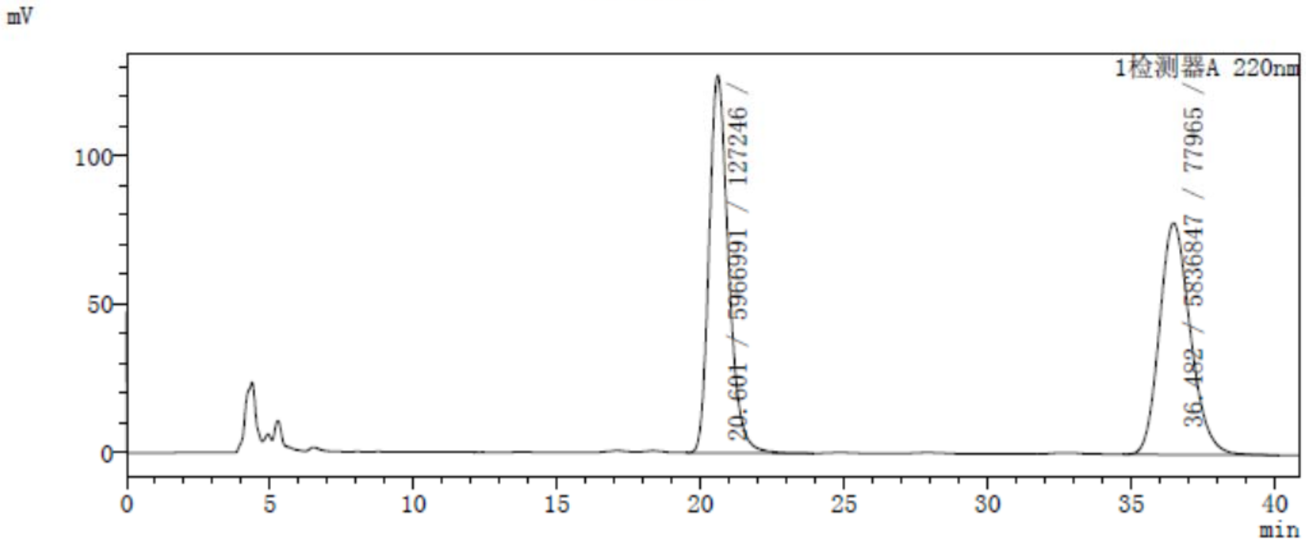

峰表

检测器A 220nm

峰号	保留时间	面积	高度	标记	面积%
1	20.601	5966991	127246		50.551
2	36.482	5836847	77965		49.449
总计		11803838	205211		100.000

Supplementary Figure 7. HPLC spectrum for racemic 3a

AD-H , n-hex : iPrOH=55/45, 0.9 ml/min, 220 nm

分析者	: System Administrator
样品名	: L-16
样品ID	:
进样体积	: 10
数据文件	: L-16. lcd
方法文件	: 13. lcm
报告格式文件	: xt. lsr
分析日期/时间	: 2016-6-16 16:23:22
处理日期/时间	: 2016-6-16 17:04:08
重复进样计数	: 1
描述	: AD-H , n-hex : iPrOH=55/45, 0.9 ml/min, 220 nm

色谱图
L-16

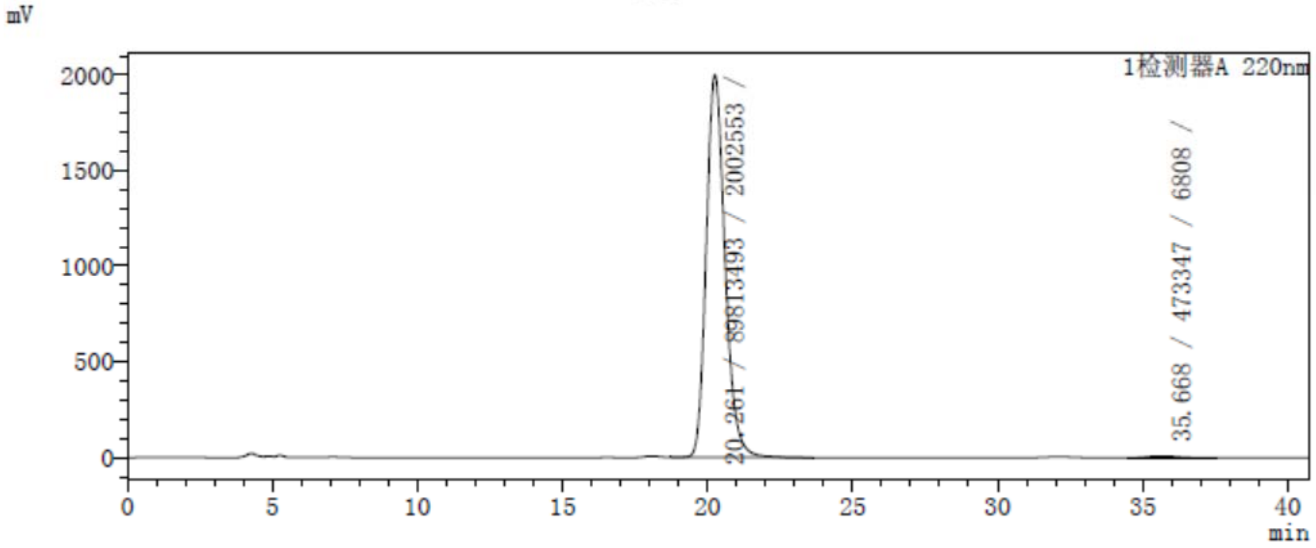

峰表

检测器A 220nm

峰号	保留时间	面积	高度	标记	面积%
1	20.261	89813493	2002553		99.476
2	35.668	473347	6808	M	0.524
总计		90286840	2009361		100.000

Supplementary Figure 8. HPLC spectrum for 3a (reaction time: 16 h)

AD-H , n-hex : iPrOH=55/45, 0.9 ml/min, 220 nm

分析者	:	System Administrator
样品名	:	LG5-44-1
样品ID	:	
进样体积	:	10
数据文件	:	LG5-44-1.lcd
方法文件	:	13.lcm
报告格式文件	:	xt.lsr
分析日期/时间	:	2016-6-16 17:05:07
处理日期/时间	:	2016-6-16 17:51:15
重复进样计数	:	1
描述	:	AD-H , n-hex : iPrOH=55/45, 0.9 ml/min, 220 nm

色谱图
LG5-44-1

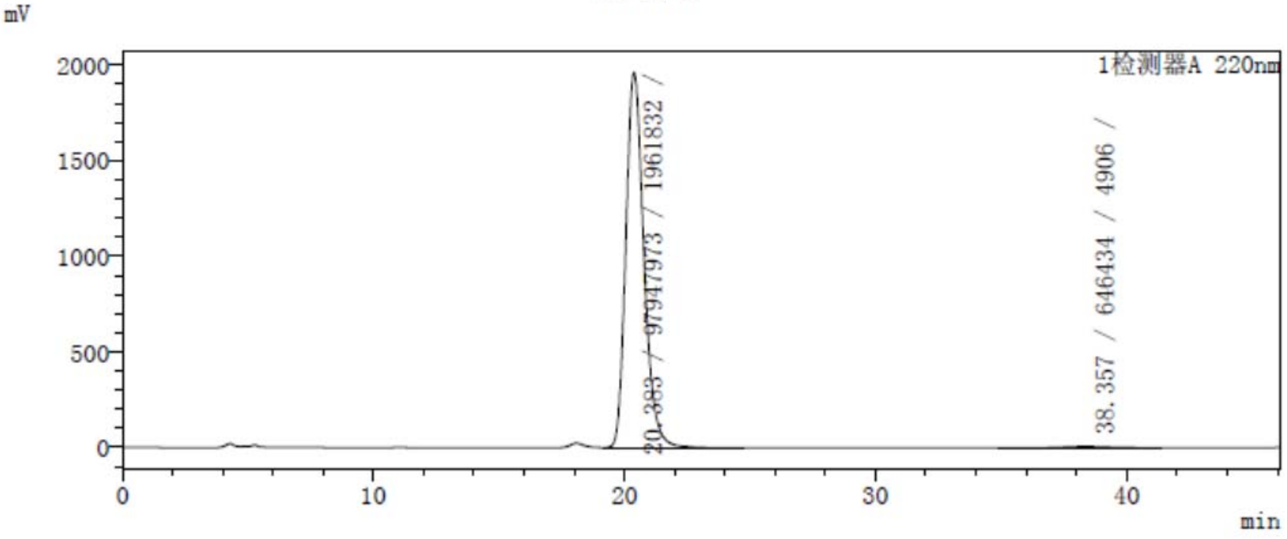

峰表

检测器A 220nm

峰号	保留时间	面积	高度	标记	面积%
1	20.383	97947973	1961832		99.344
2	38.357	646434	4906		0.656
总计		98594406	1966737		100.000

Supplementary Figure 9. HPLC spectrum for 3a (reaction time: 24 h)

AD-H , n-hex : iPrOH=55/45, 0.9 ml/min, 220 nm

分析者 : System Administrator
样品名 : LG5-44-2
样品ID :
进样体积 : 2.5
数据文件 : LG5-44-2.lcd
方法文件 : 13.lcm
报告格式文件 : xt.lsr
分析日期/时间 : 2016-6-16 22:27:25
处理日期/时间 : 2016-6-16 23:08:45
重复进样计数 : 1
描述 : AD-H , n-hex : iPrOH=55/45, 0.9 ml/min, 220 nm

色谱图
LG5-44-2

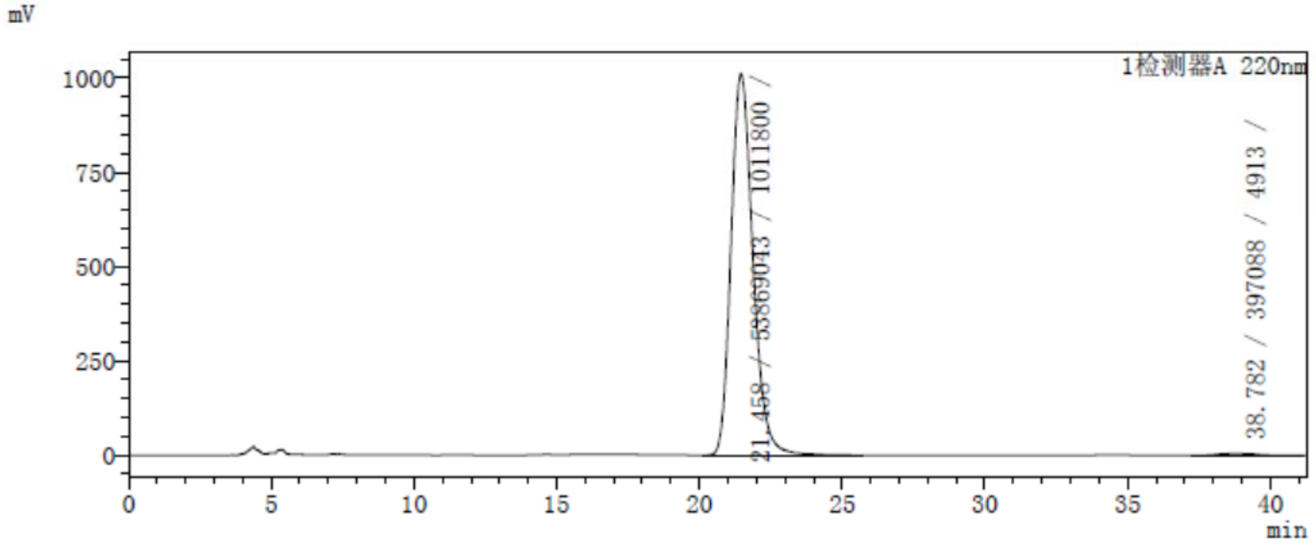

峰表

检测器A 220nm

峰号	保留时间	面积	高度	标记	面积%
1	21.458	53869043	1011800		99.268
2	38.782	397088	4913		0.732
总计		54266132	1016713		100.000

Supplementary Figure 10. HPLC spectrum for 3a (reaction time: 48 h)

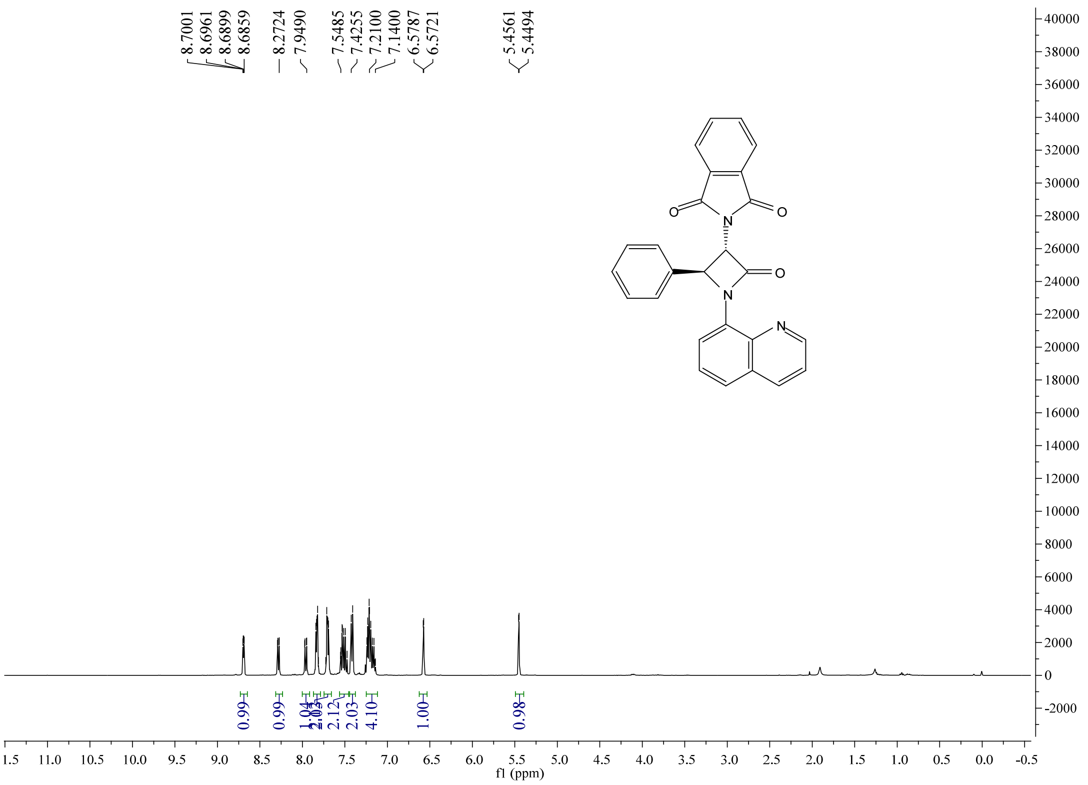

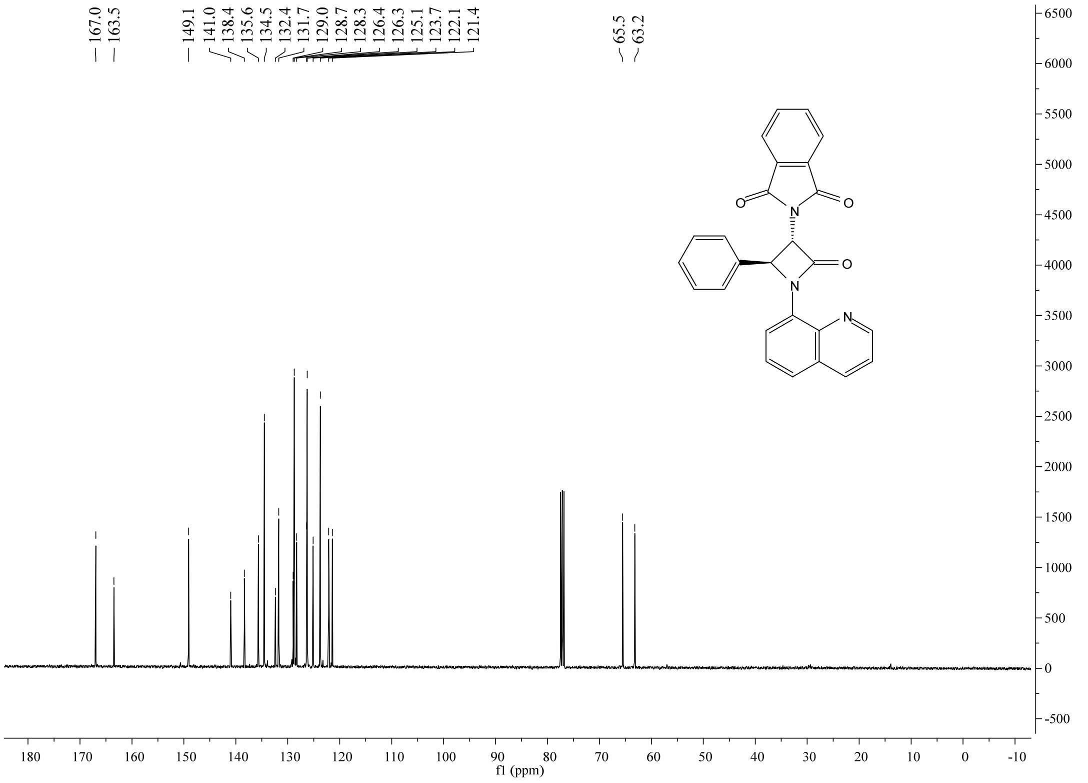

Supplementary Figure 11. ^{1}H and ^{13}C NMR spectra for 3aa

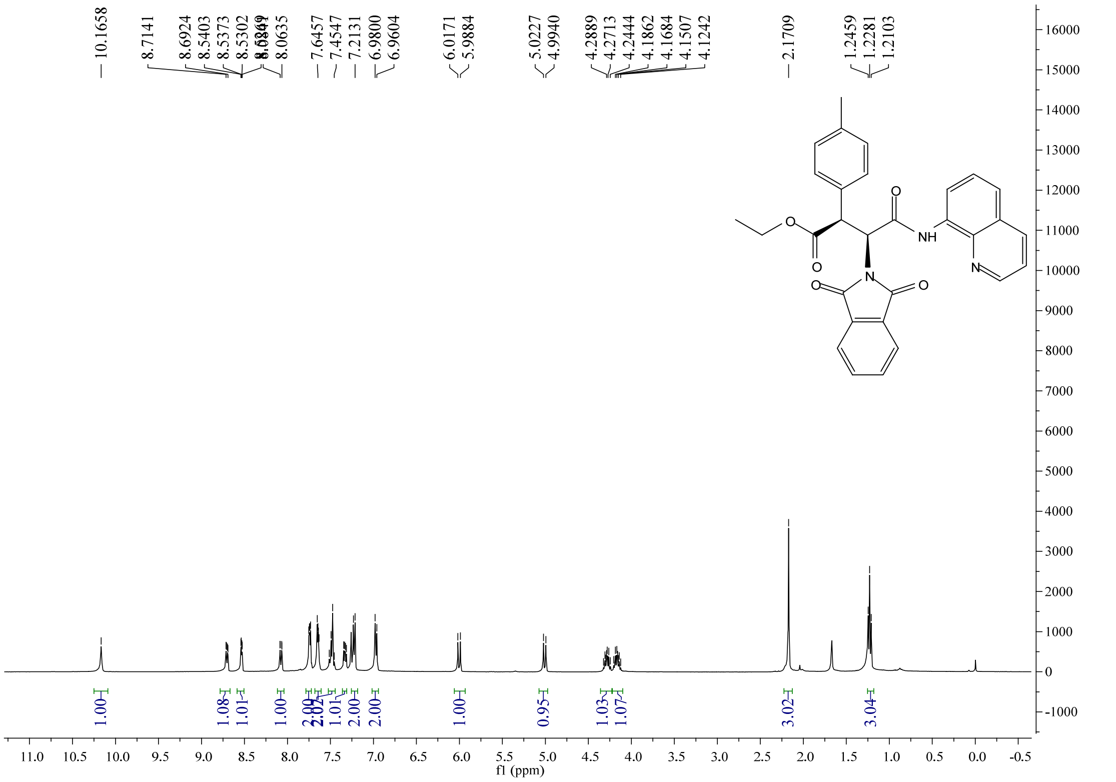

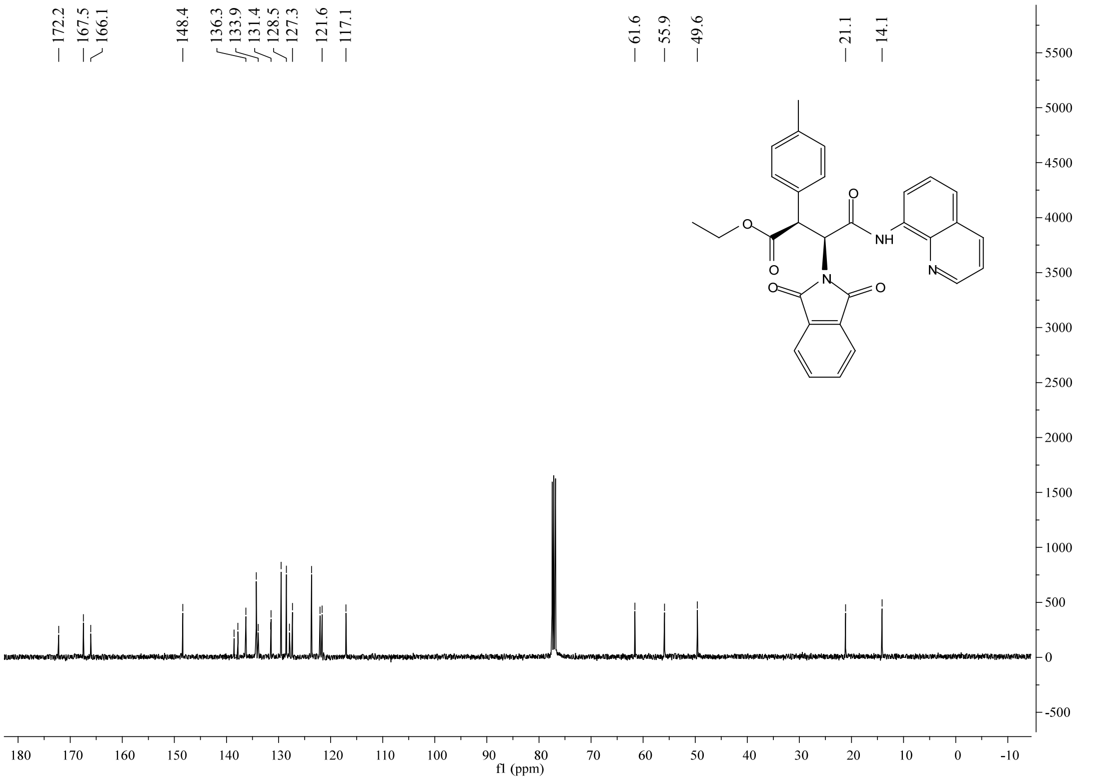

Supplementary Figure 12. ^{1}H and ^{13}C NMR spectra for 3b

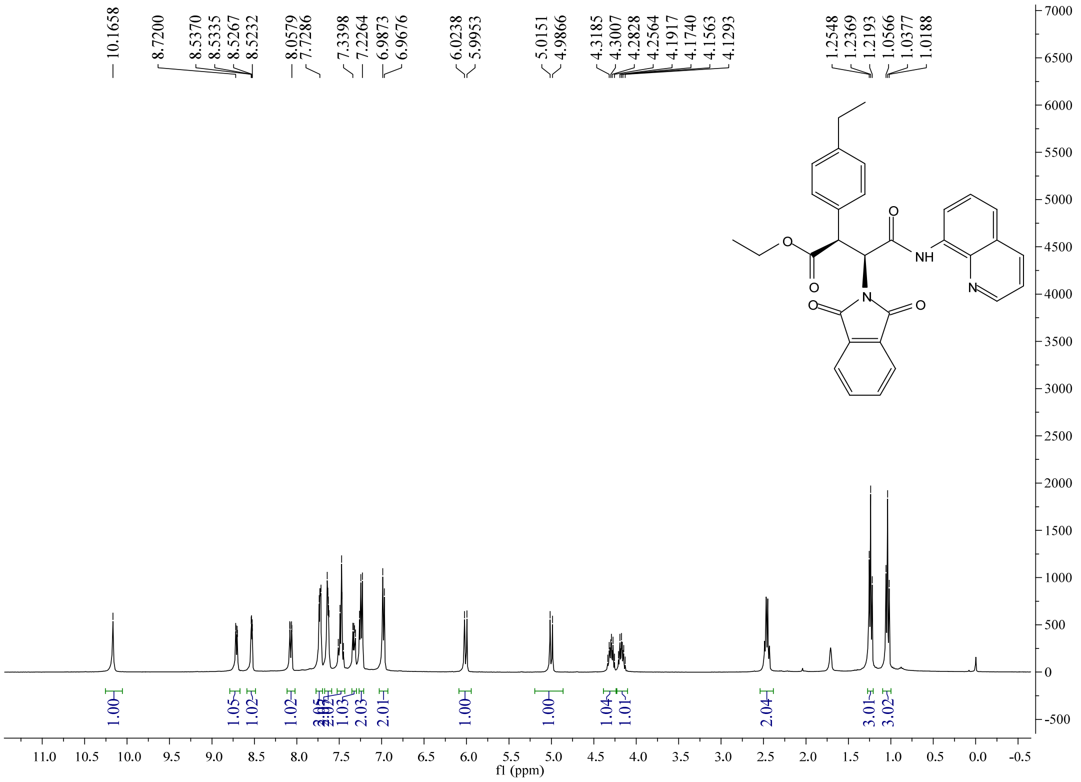

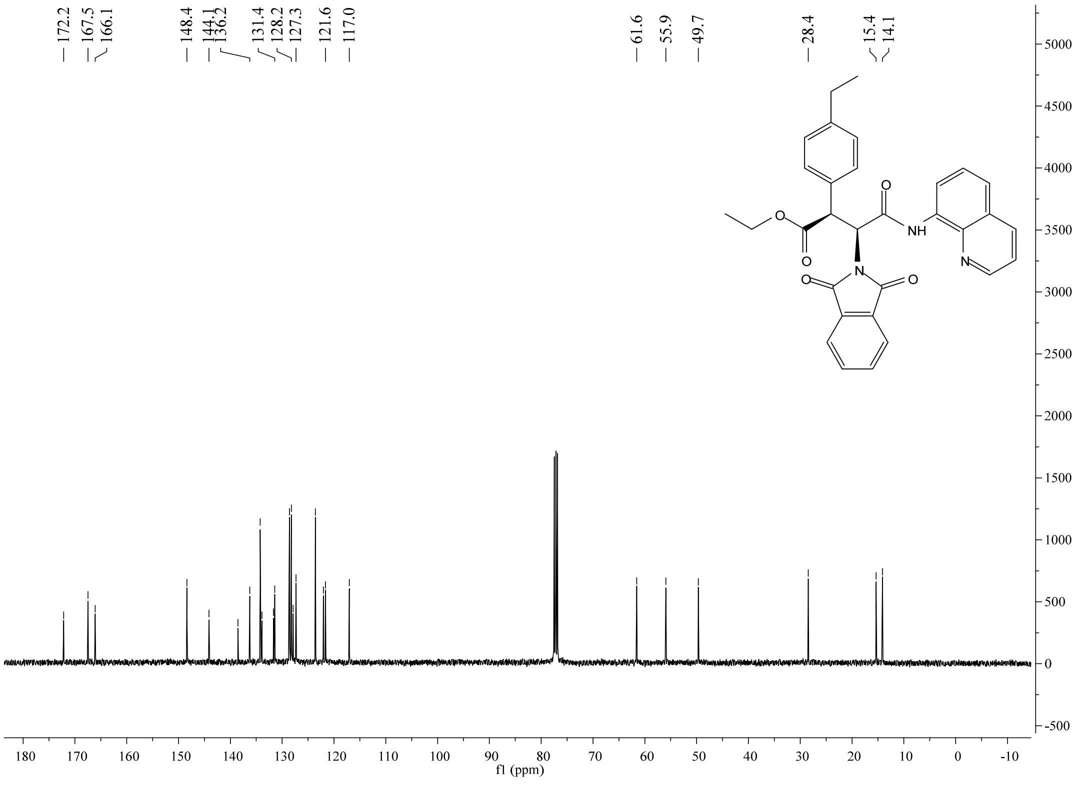

Supplementary Figure 13. ^{1}H and ^{13}C NMR spectra for 3c

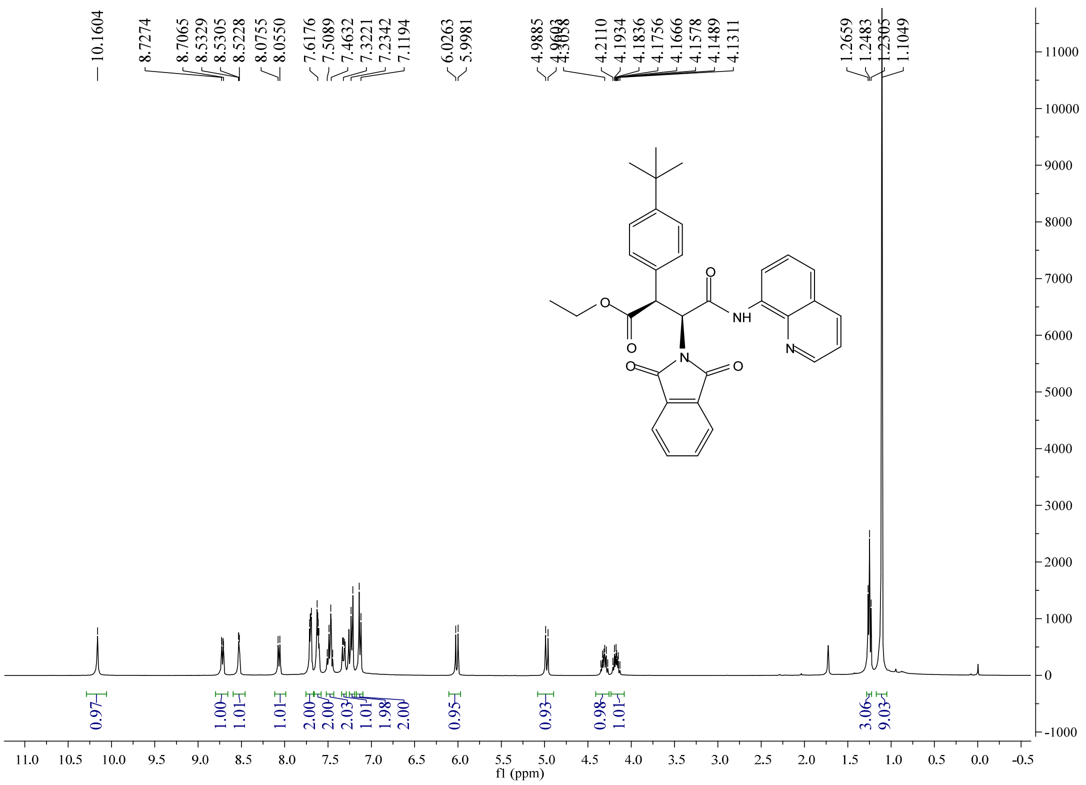

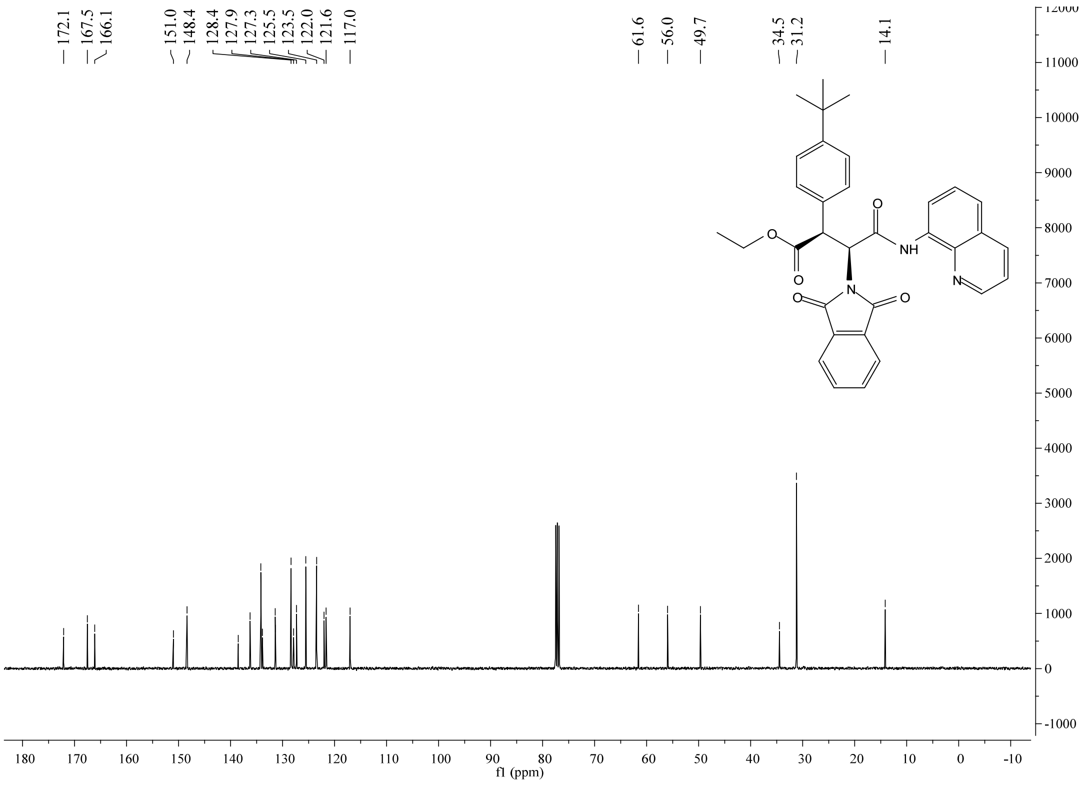

Supplementary Figure 14. ^{1}H and ^{13}C NMR spectra for 3d

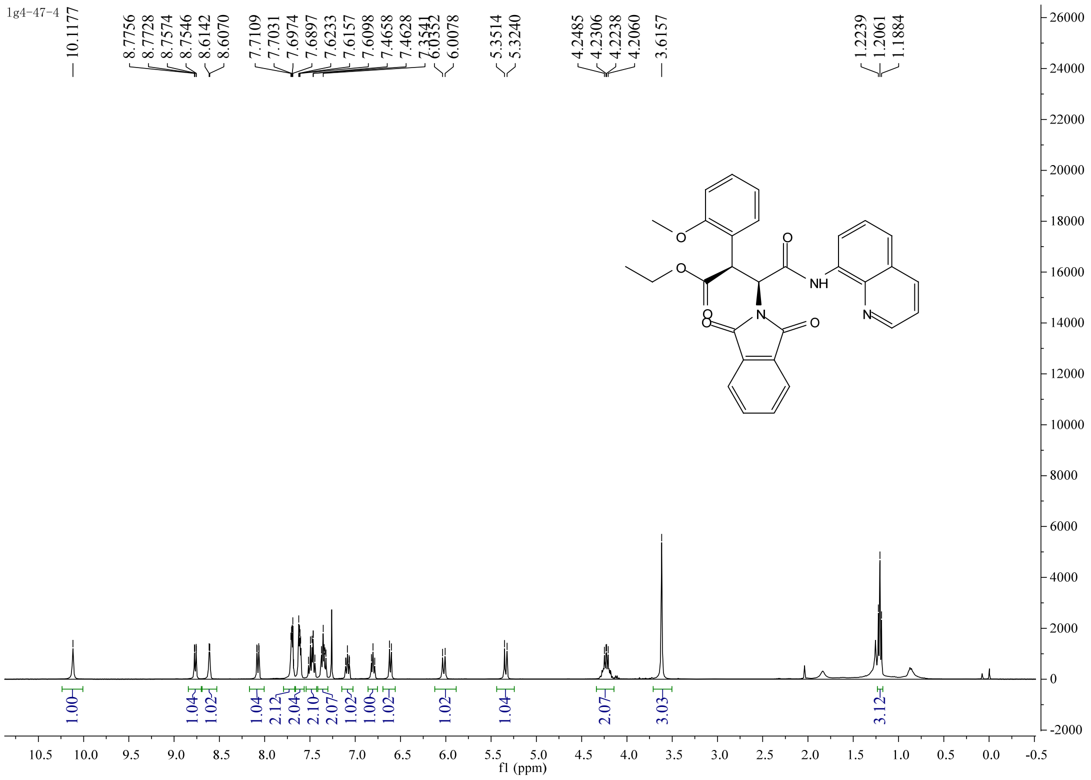

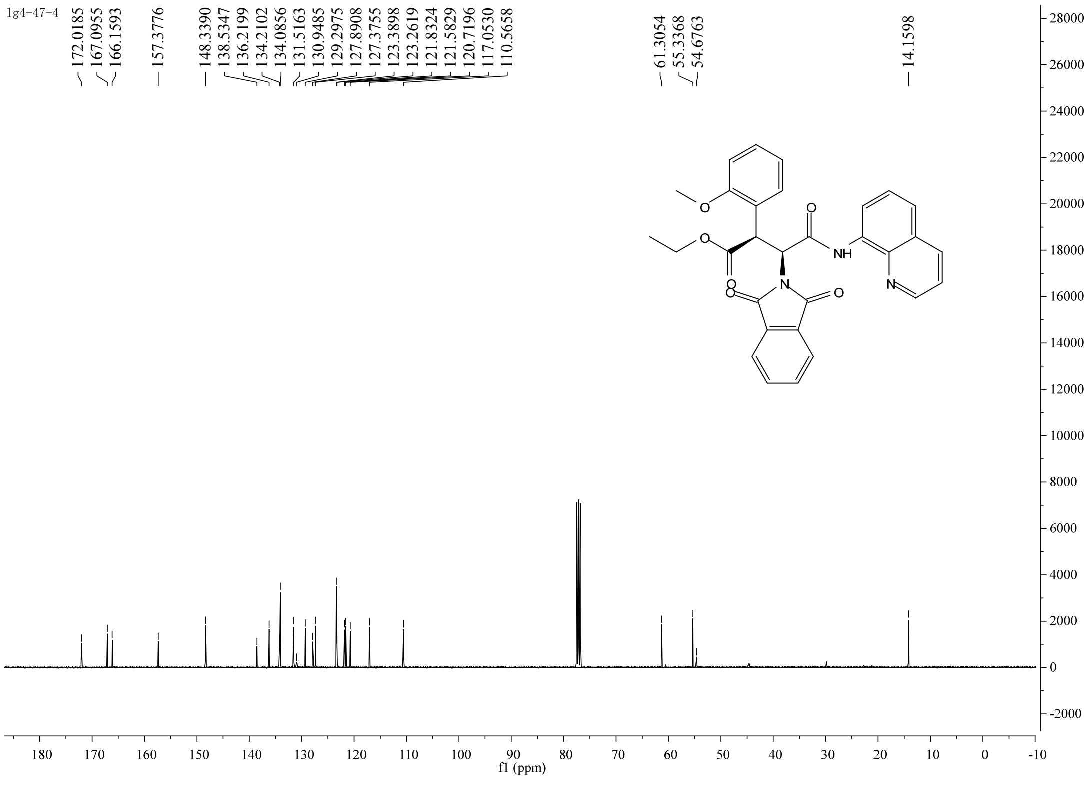

Supplementary Figure 15. ^{1}H and ^{13}C NMR spectra for 3e

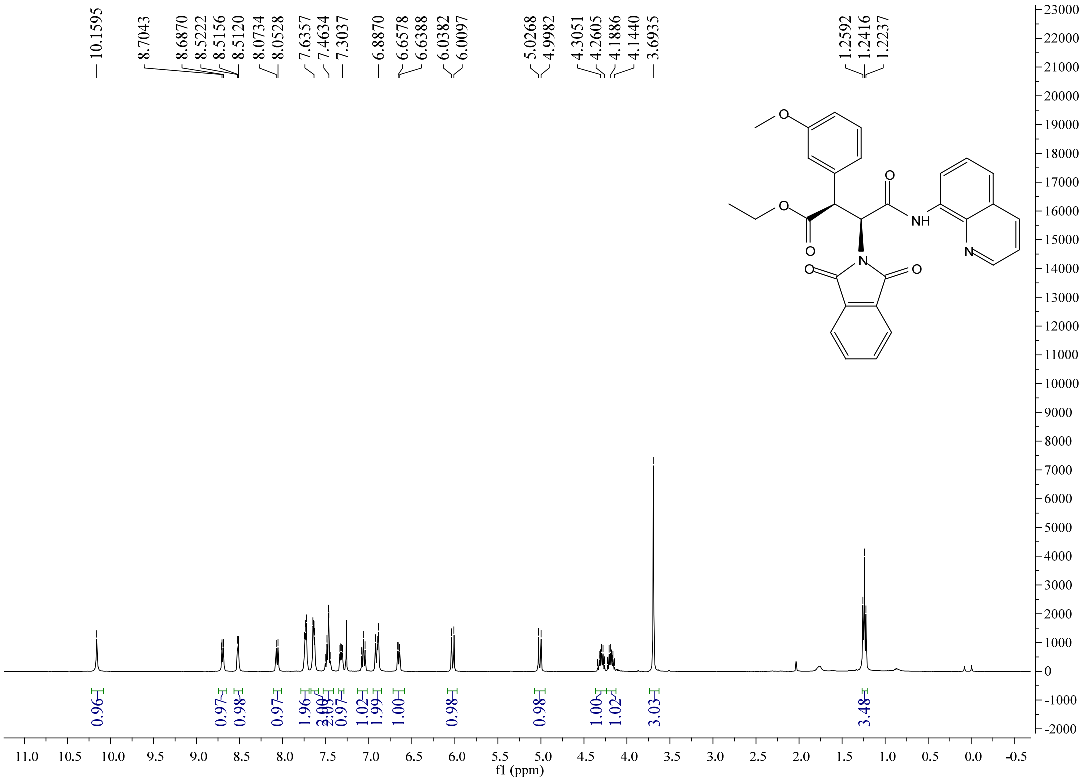

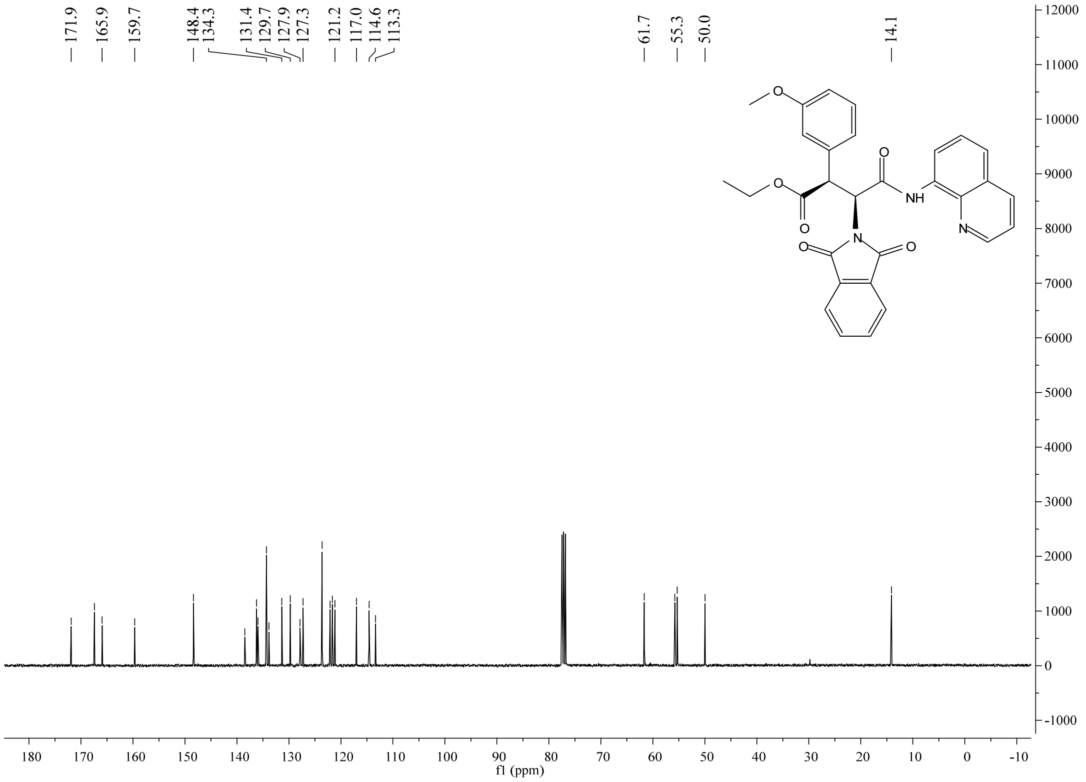

Supplementary Figure 16. ^{1}H and ^{13}C NMR spectra for 3f

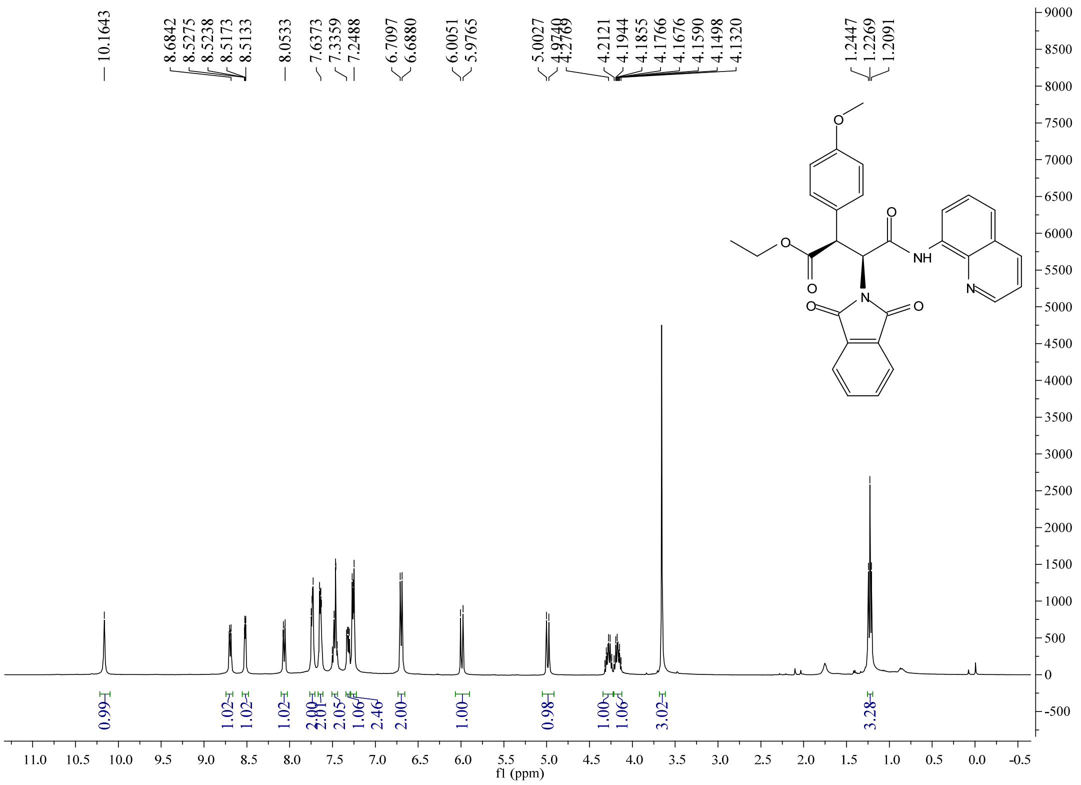

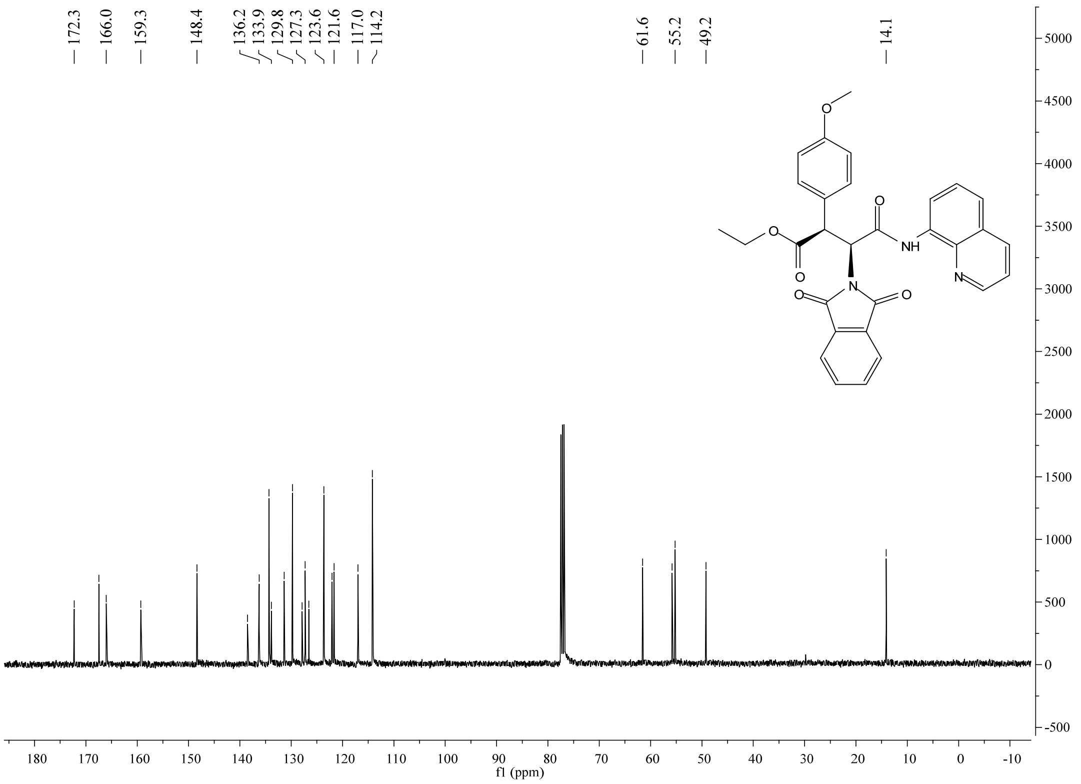

Supplementary Figure 17. ^{1}H and ^{13}C NMR spectra for 3g

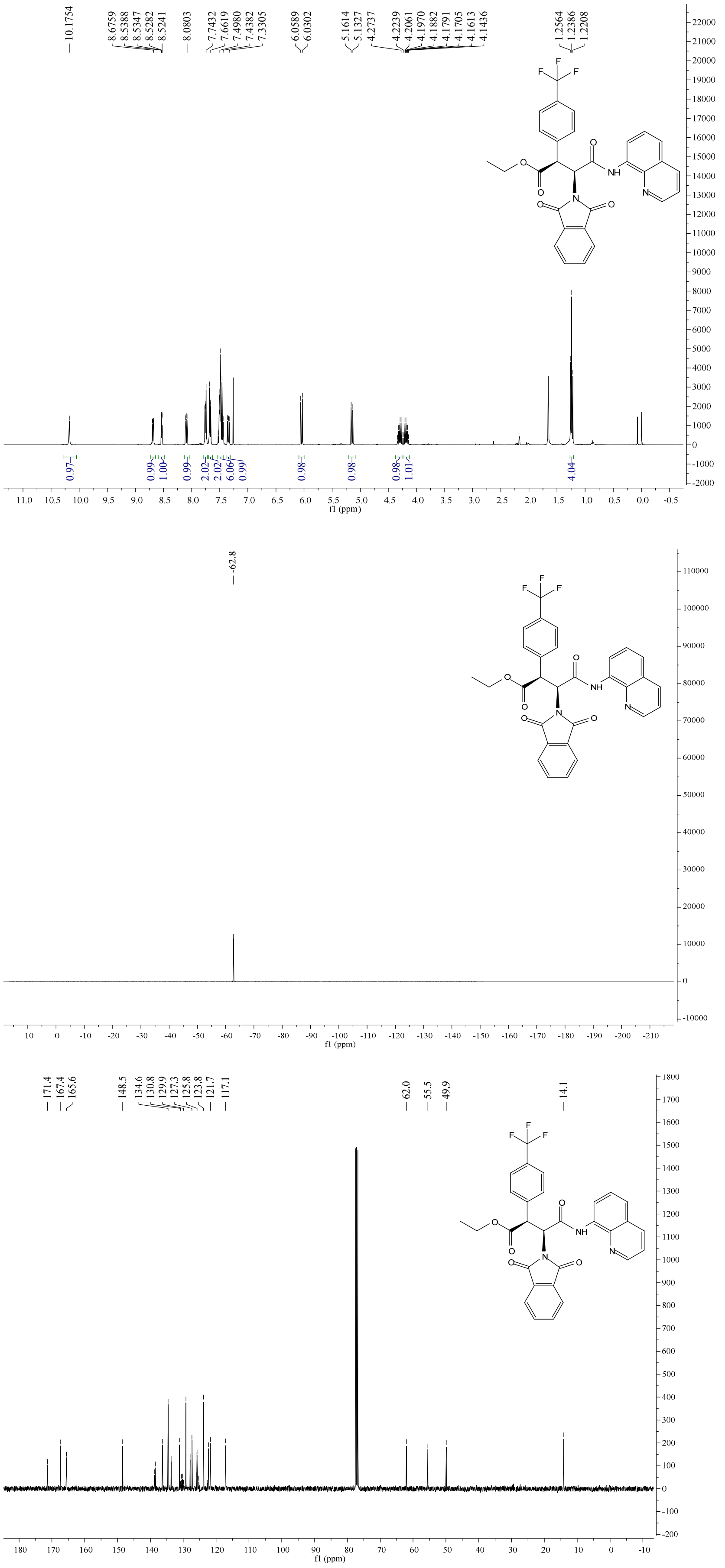

Supplementary Figure 18. ^{1}**H,** ^{19}**F and** ^{13}**C NMR spectra for 3h**

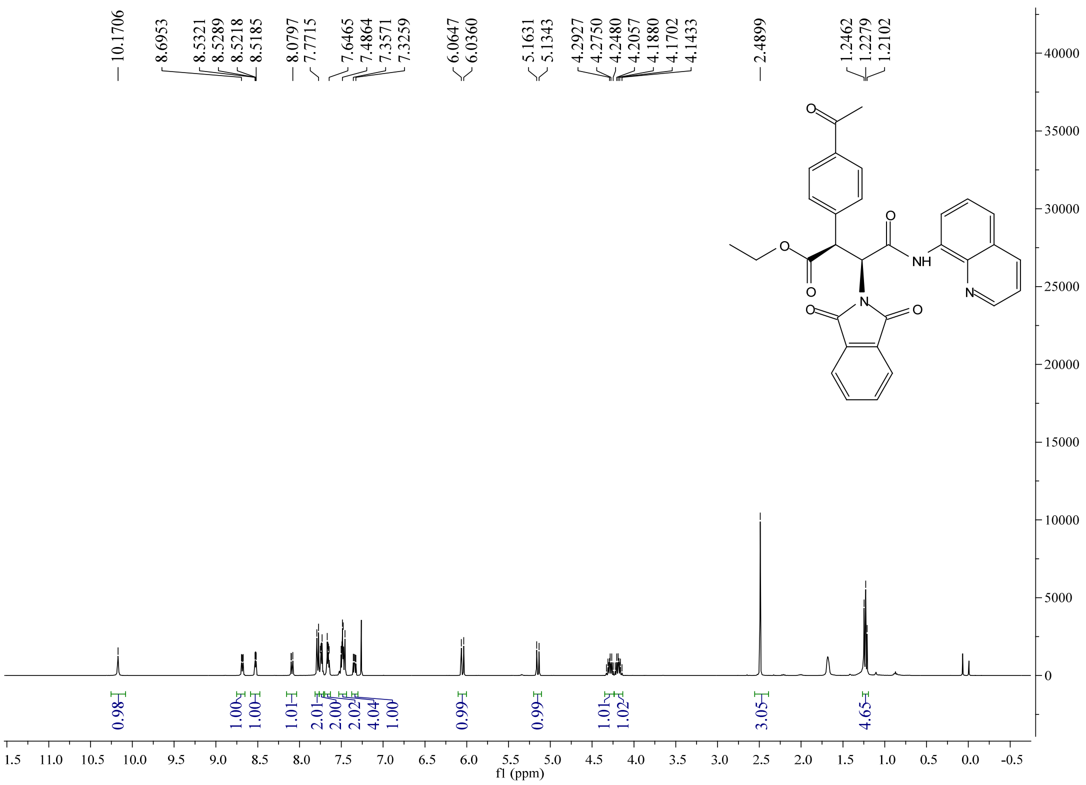

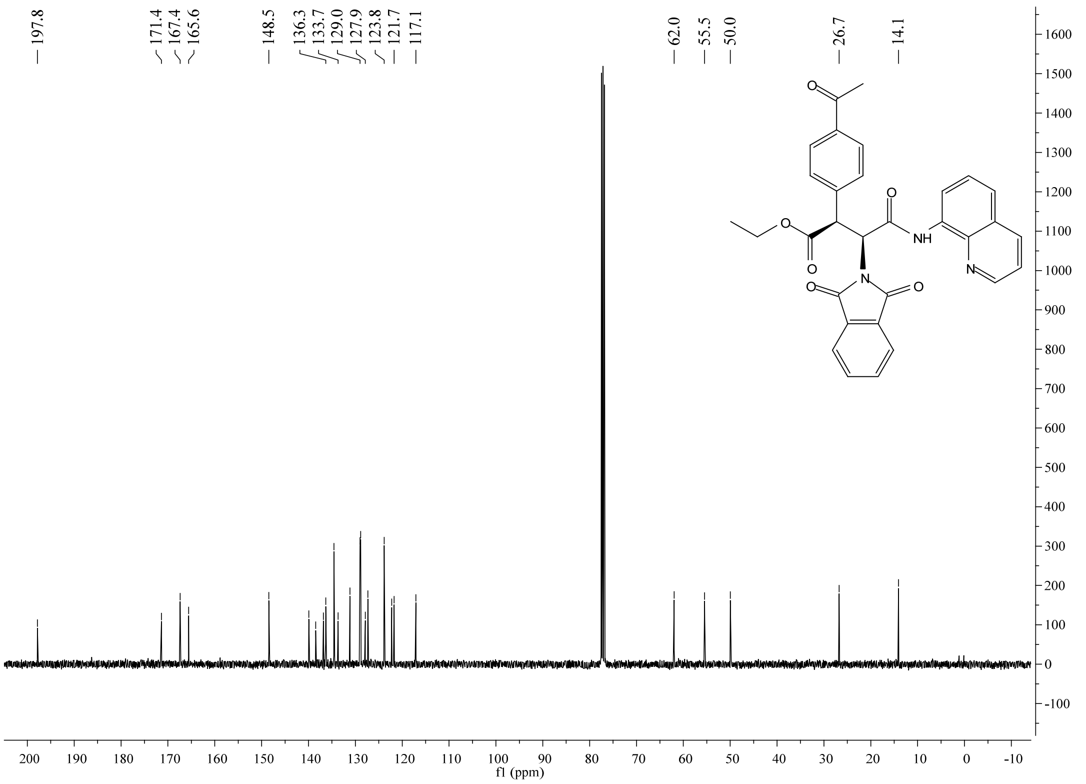

Supplementary Figure 19. ^{1}H and ^{13}C NMR spectra for 3i

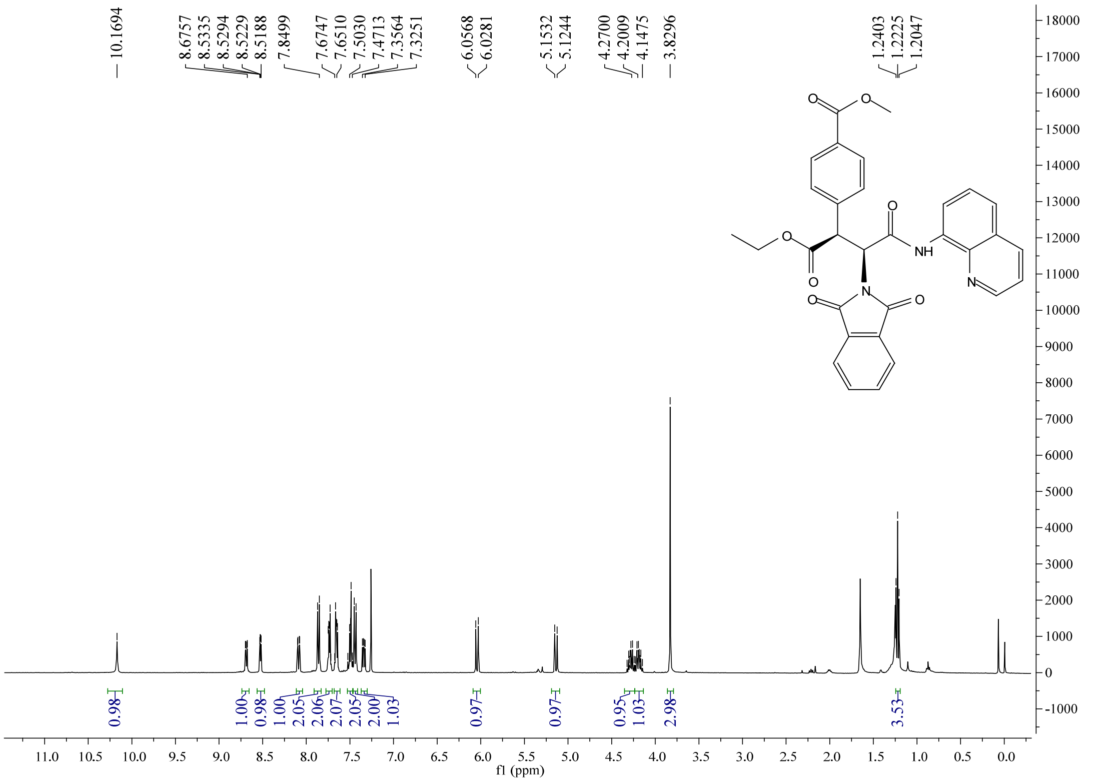

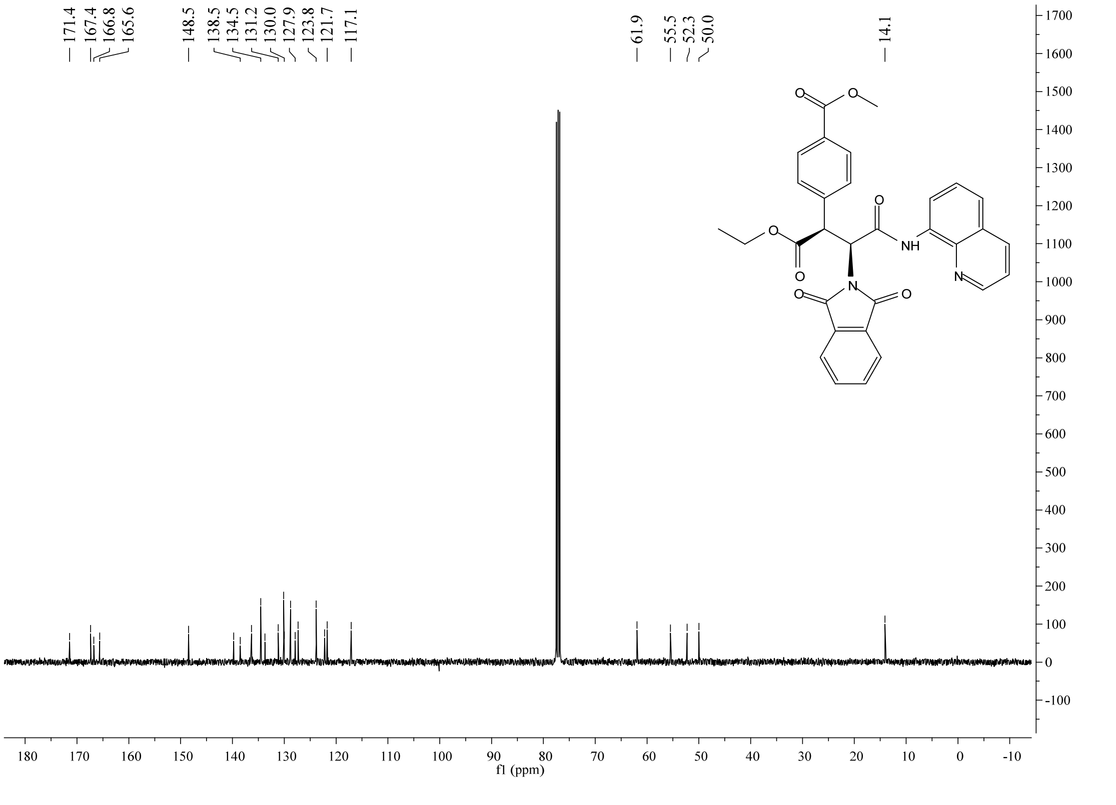

Supplementary Figure 20. ^{1}H and ^{13}C NMR spectra for 3j

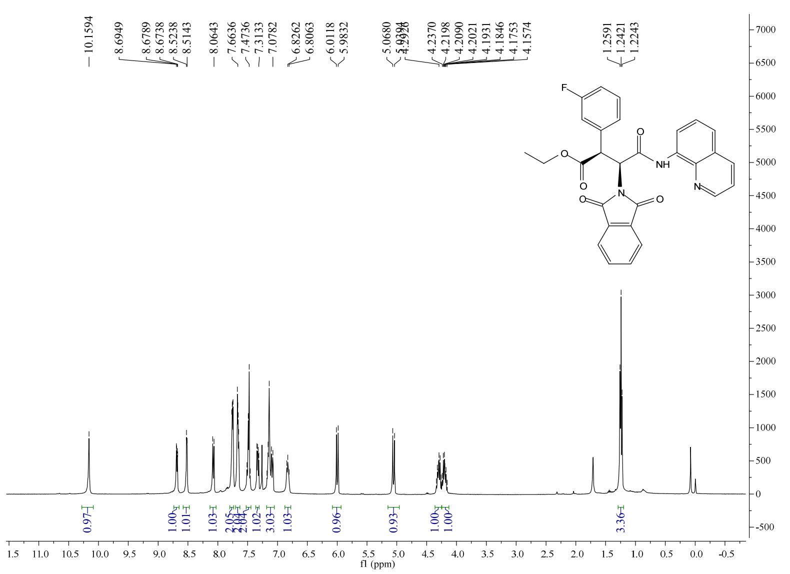

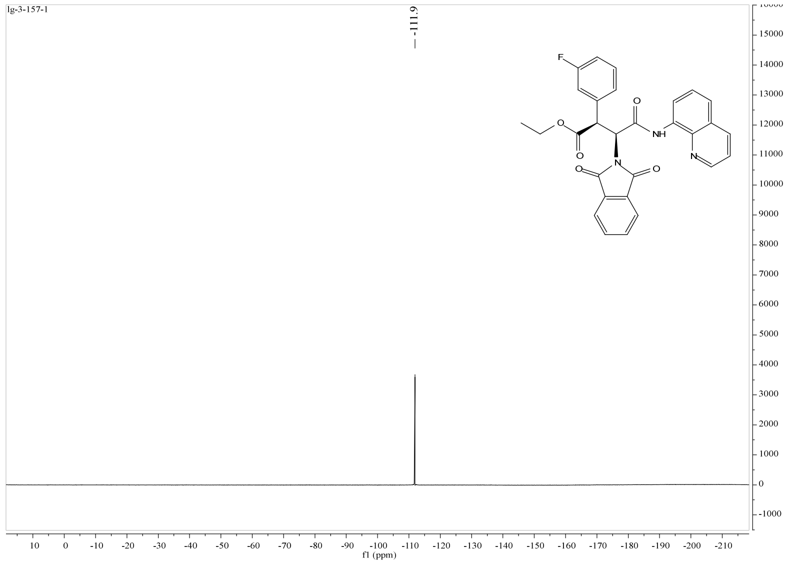

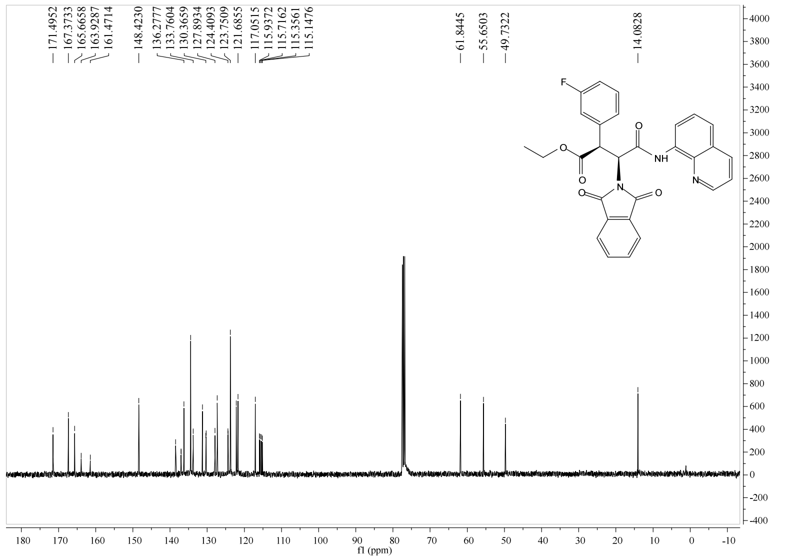

Supplementary Figure 21. ^{1}H, ^{19}F and ^{13}C NMR spectra for 3k

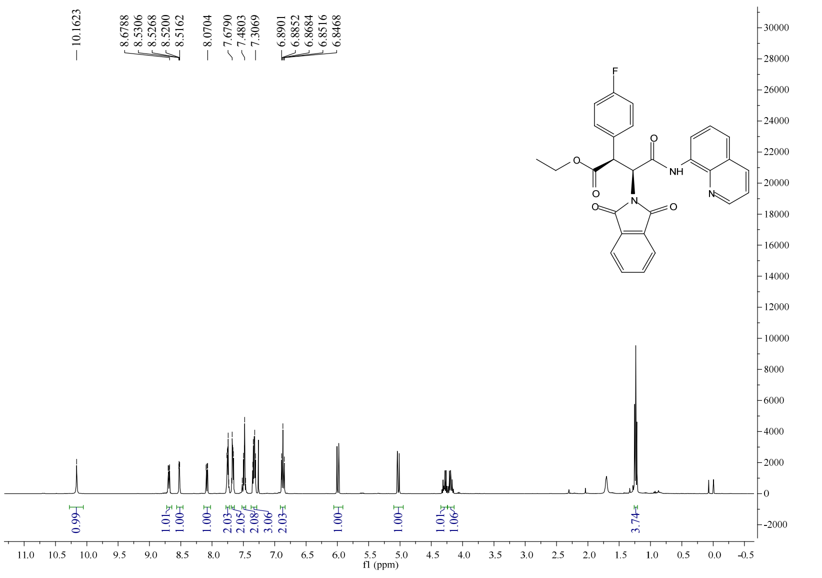

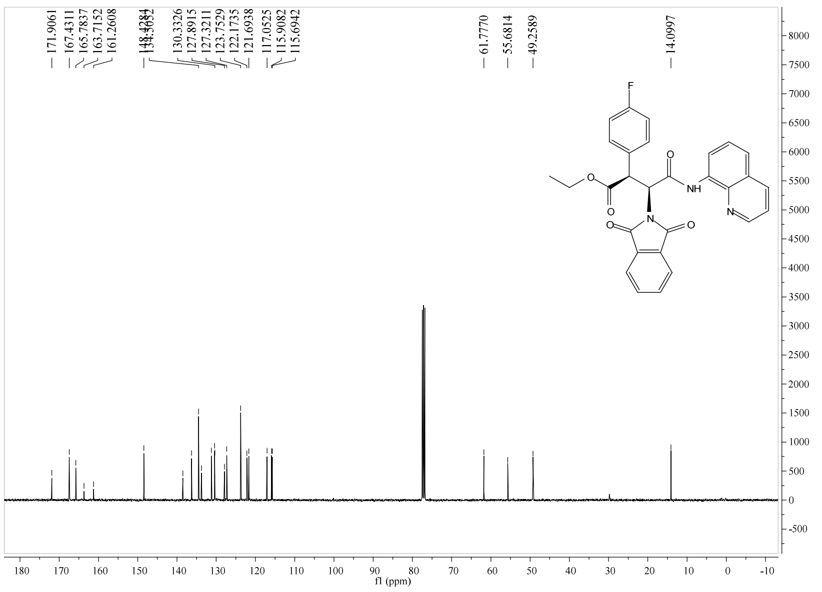

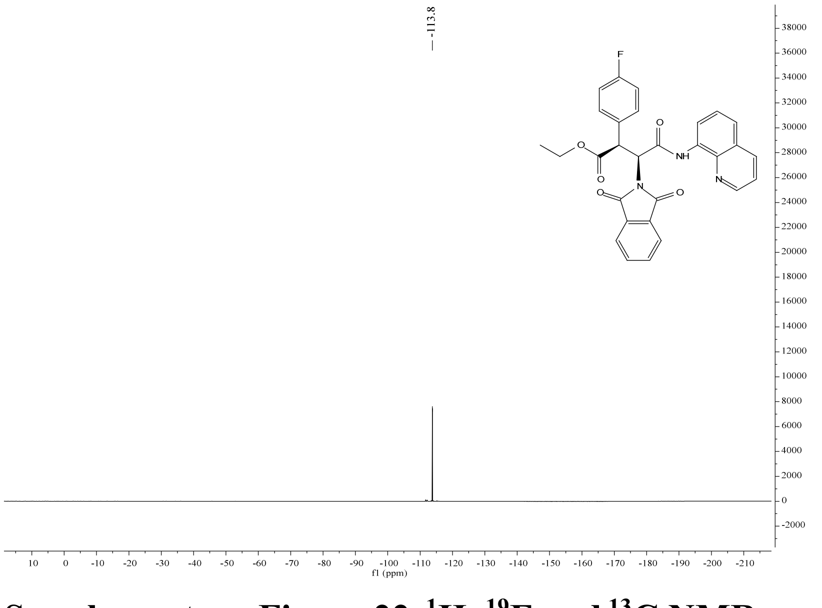

Supplementary Figure 22. ^{1}H, ^{19}F and ^{13}C NMR spectra for 3l

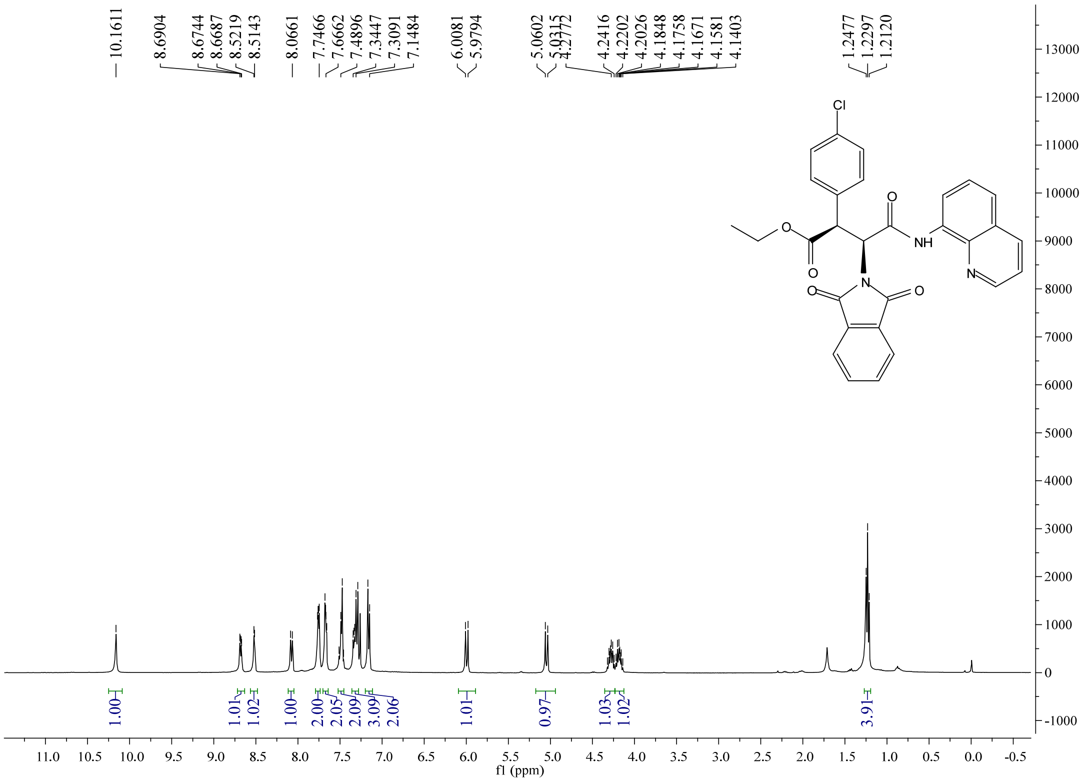

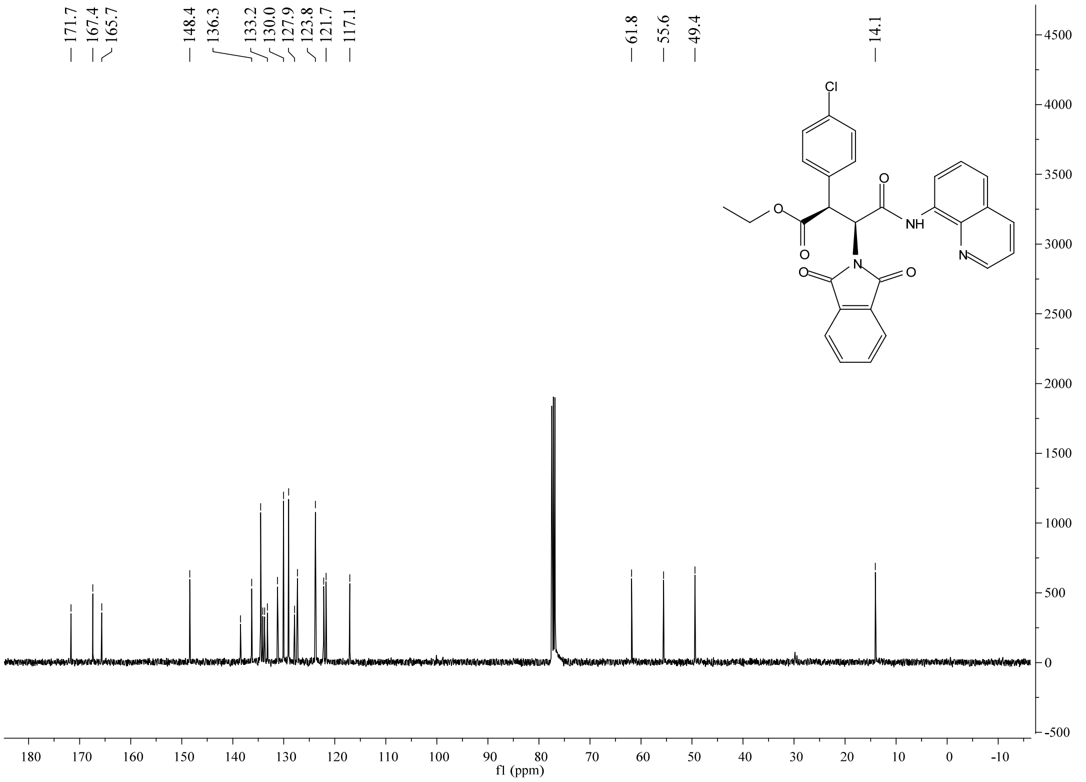

Supplementary Figure 23. ^{1}H and ^{13}C NMR spectra for 3m

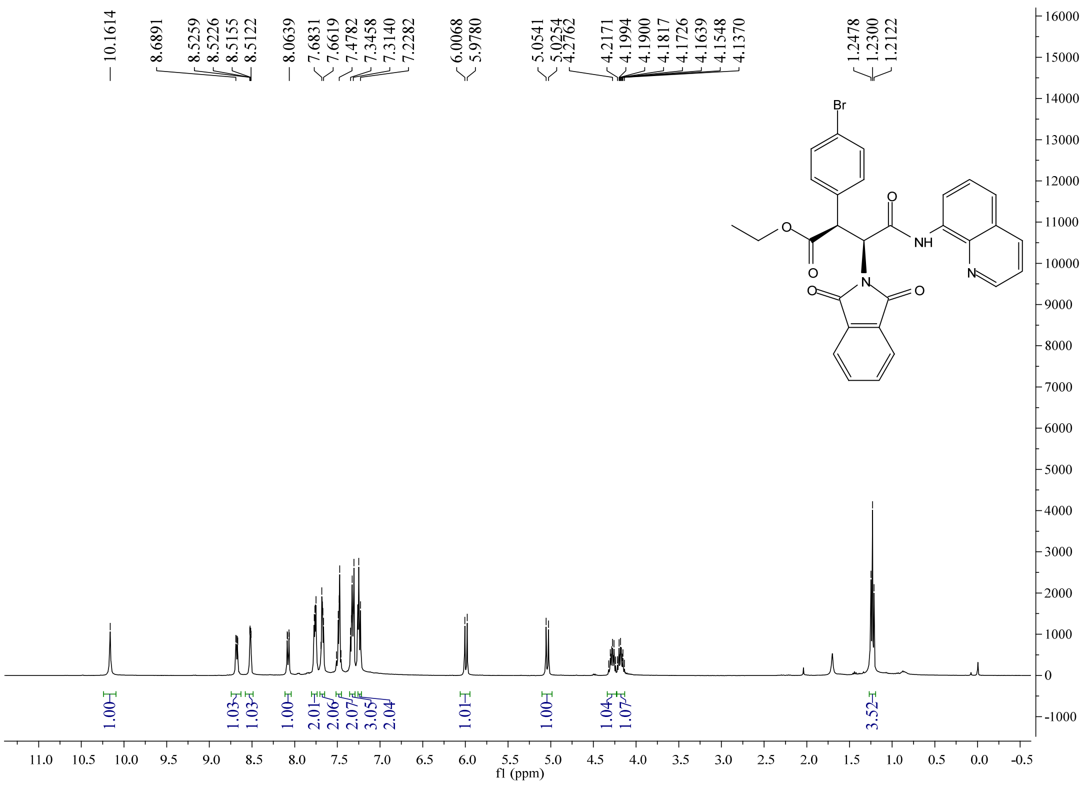

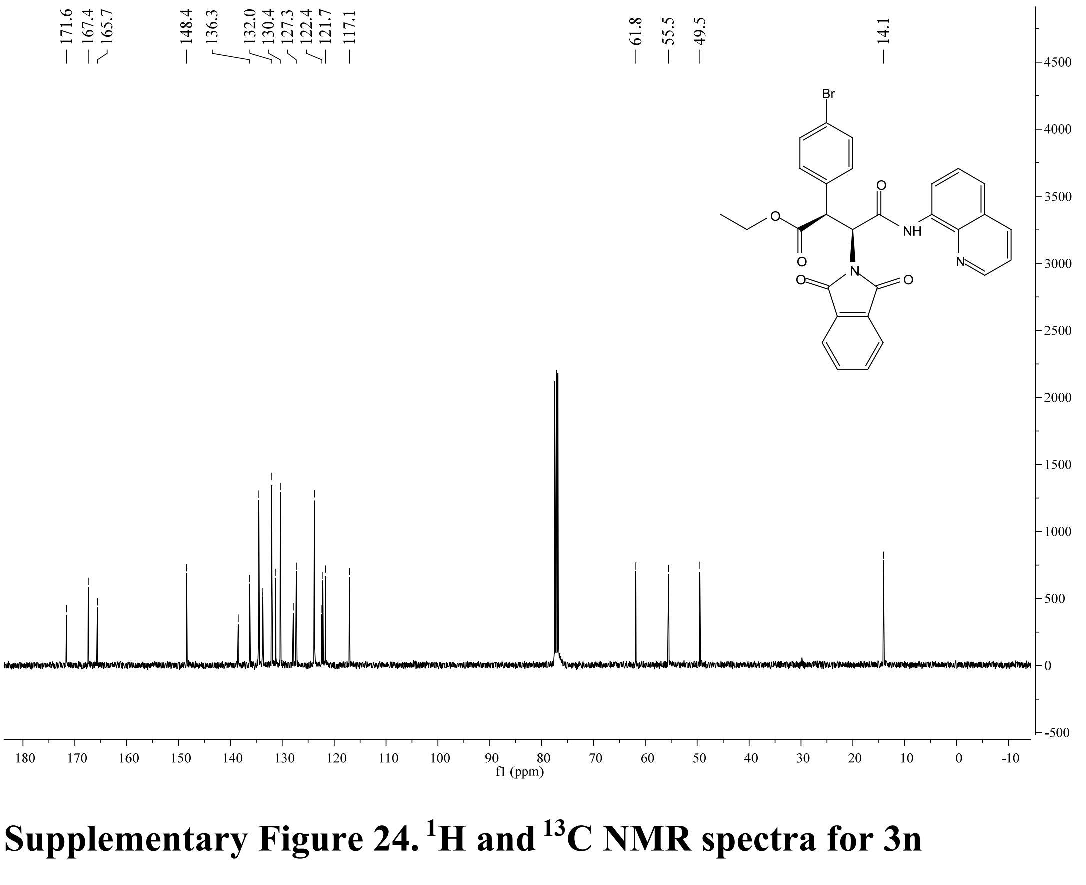

Supplementary Figure 24. ^{1}H and ^{13}C NMR spectra for 3n

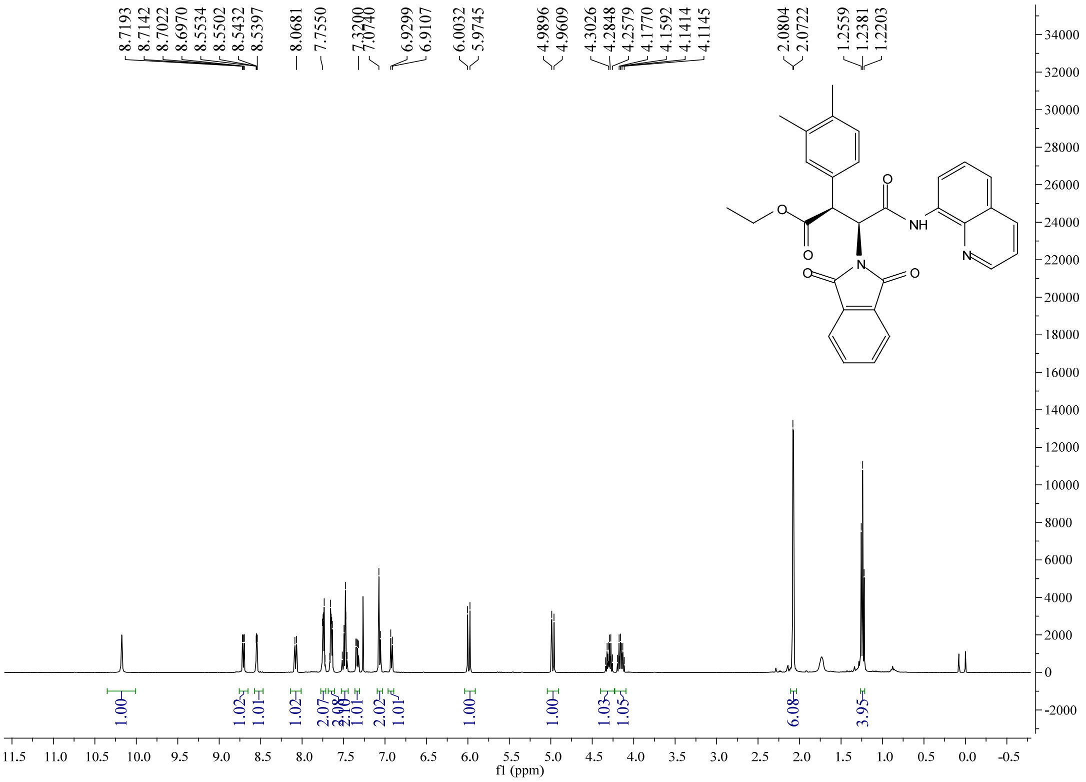

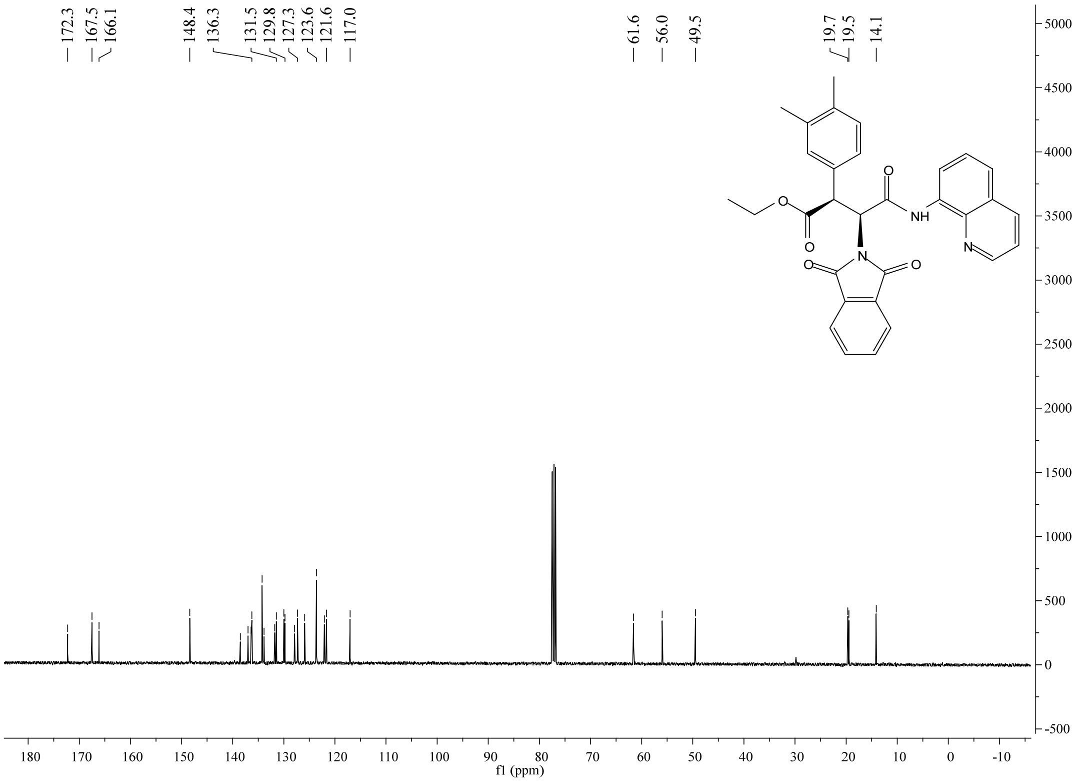

Supplementary Figure 25. ^{1}H and ^{13}C NMR spectra for 3o

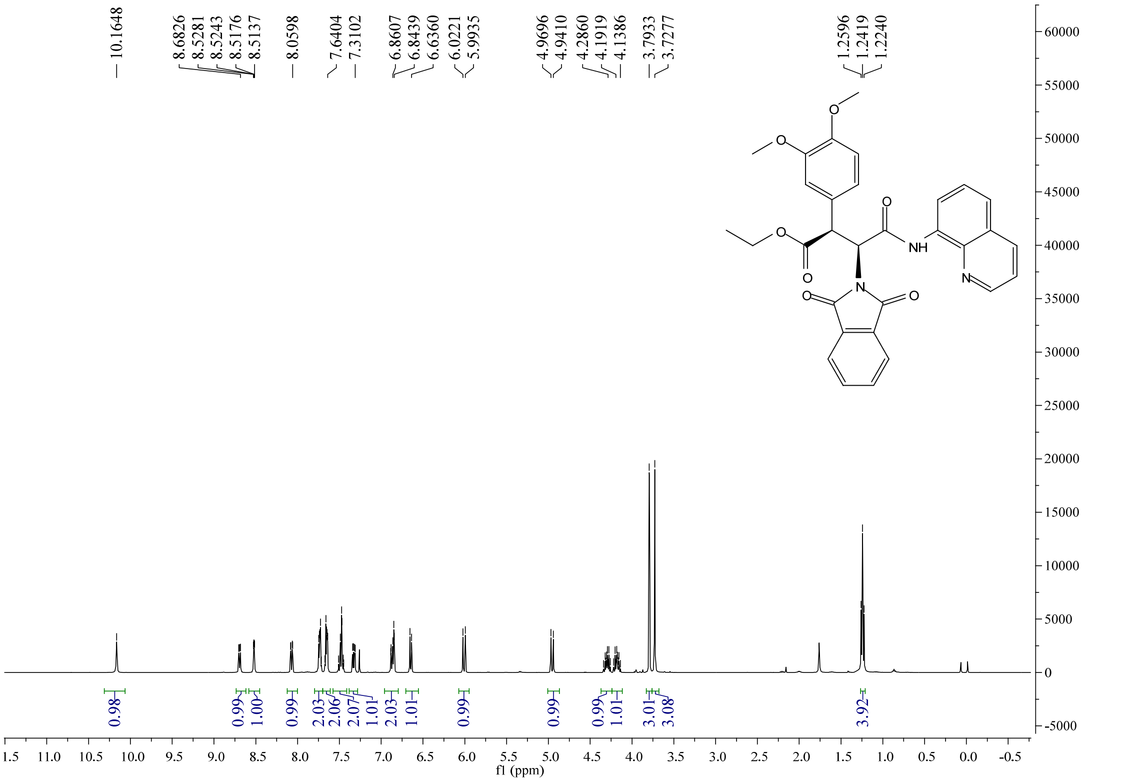

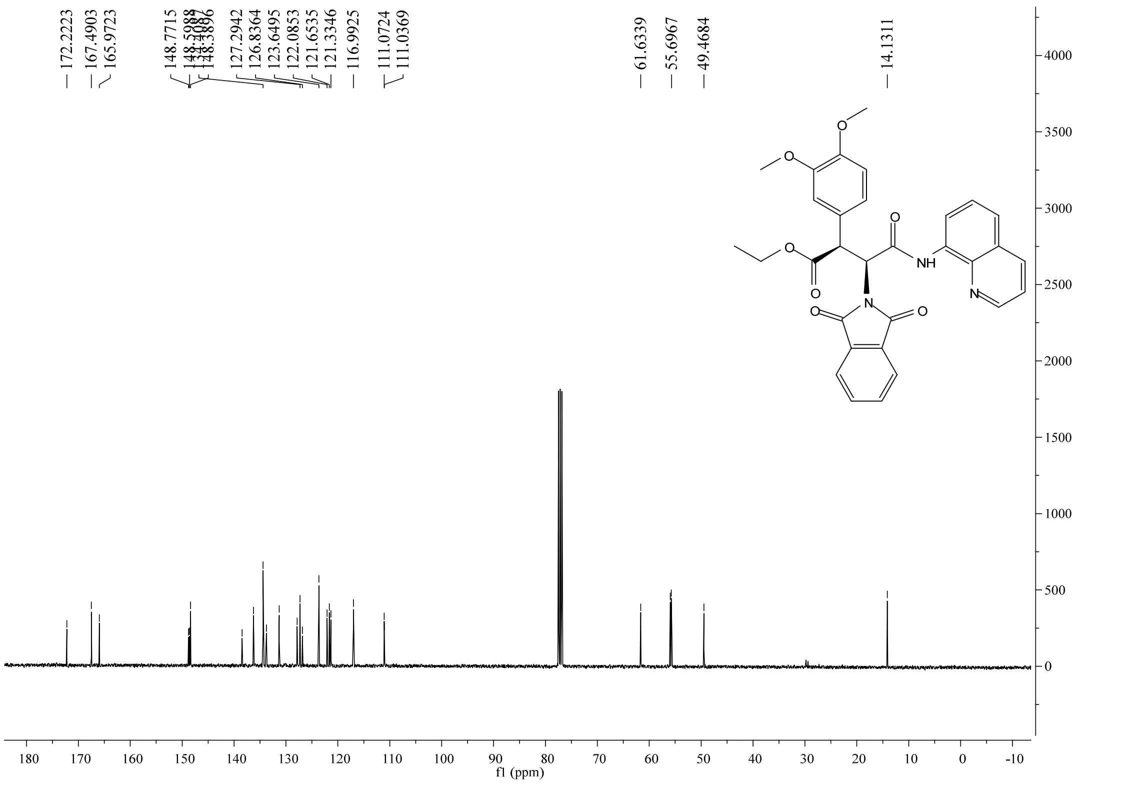

Supplementary Figure 26. ^{1}H and ^{13}C NMR spectra for 3p

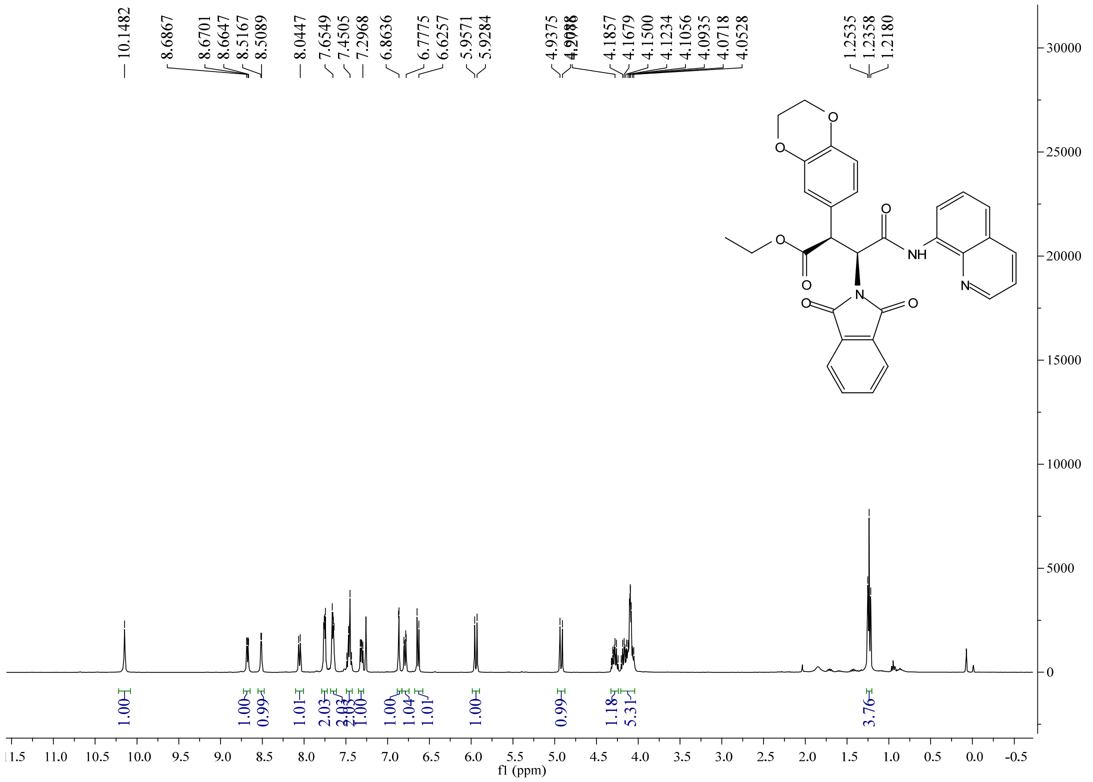

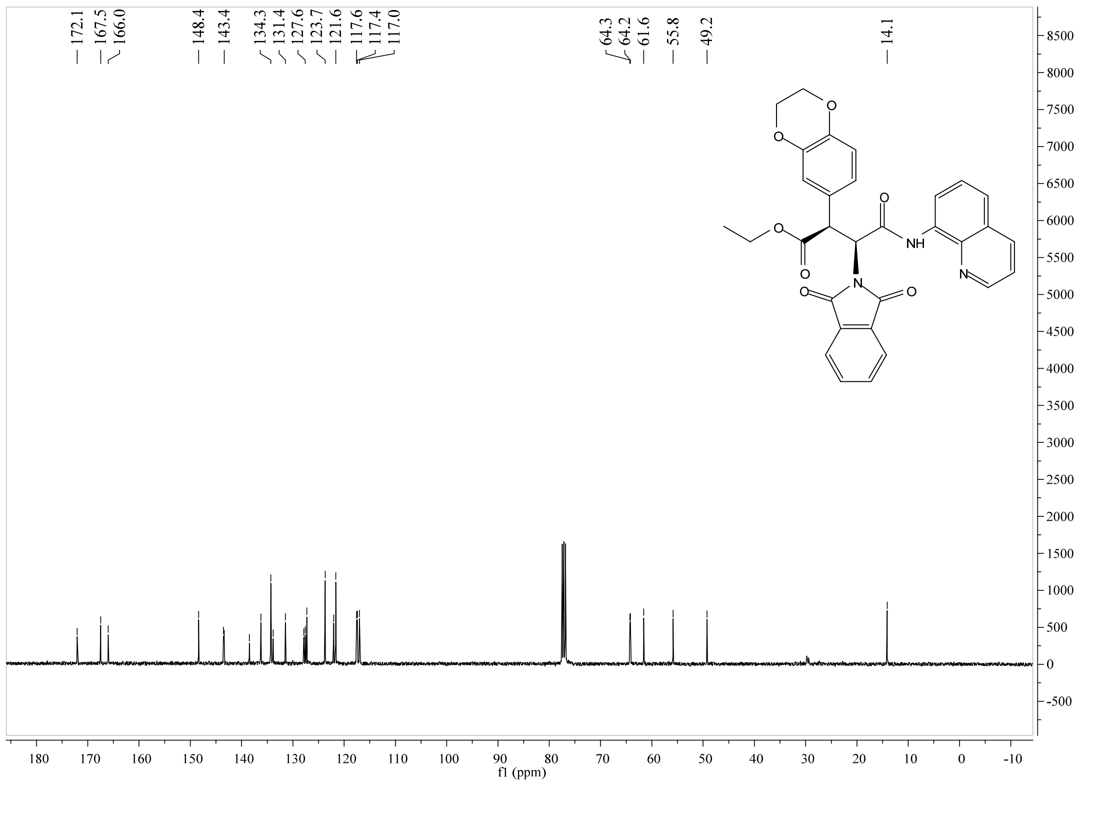

Supplementary Figure 27. ^{1}H and ^{13}C NMR spectra for 3q

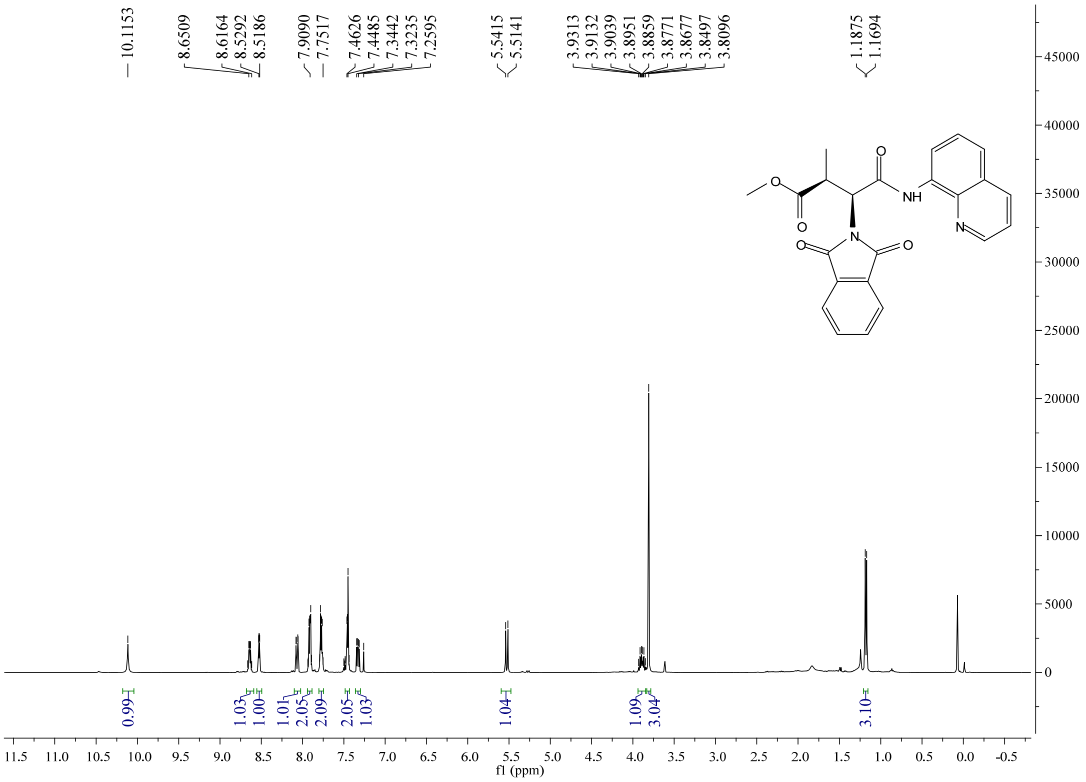

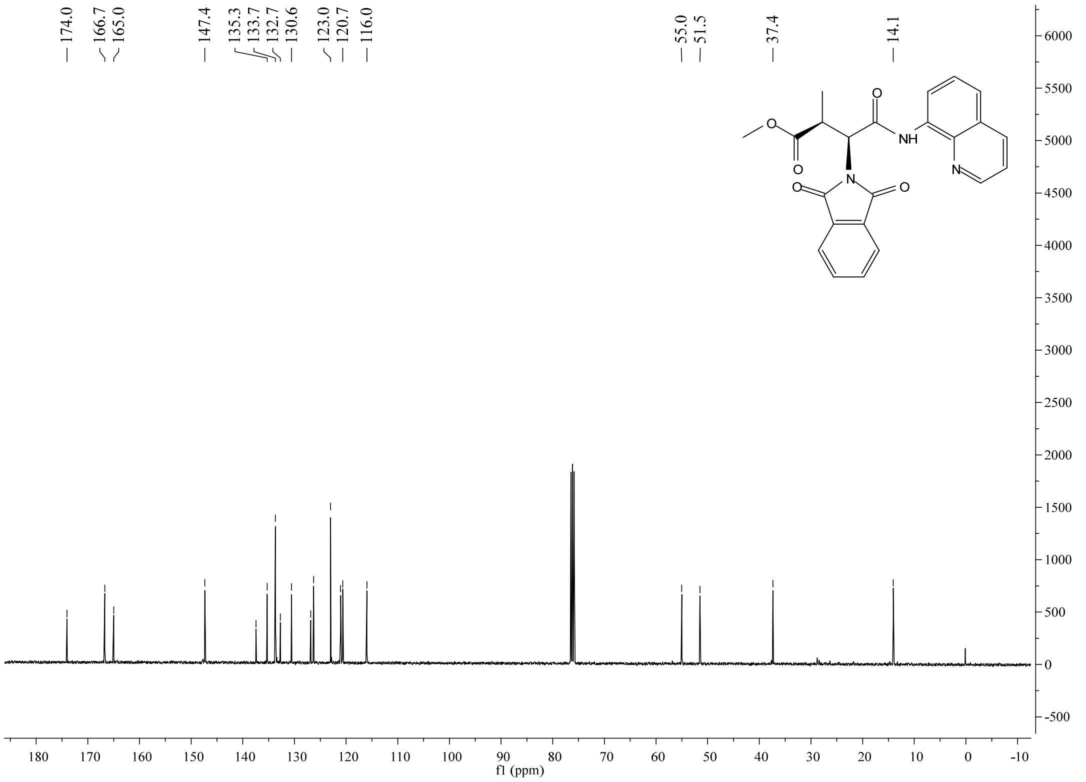

Supplementary Figure 28. ^{1}H and ^{13}C NMR spectra for 3r

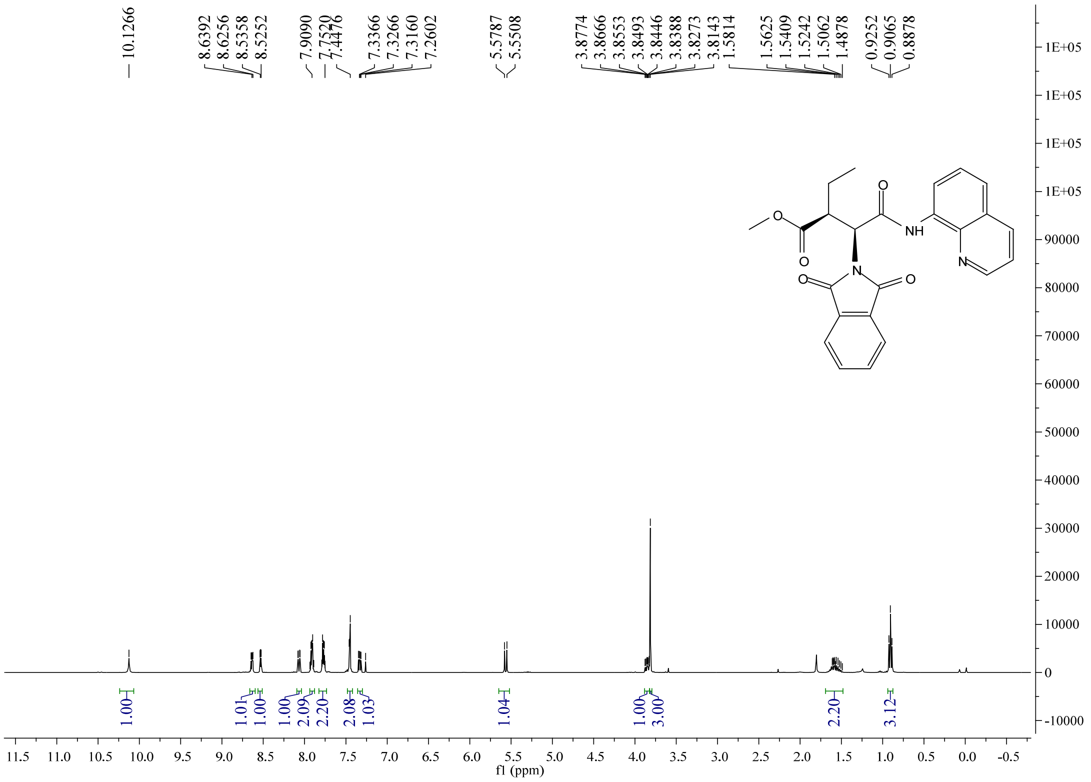

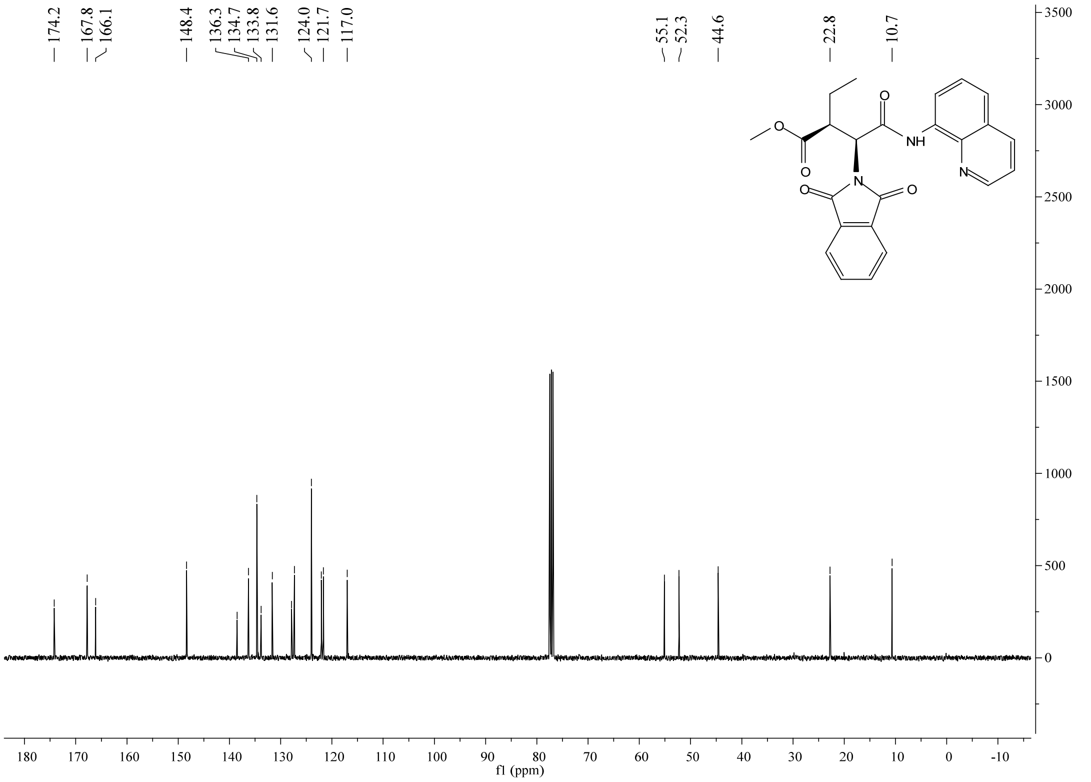

Supplementary Figure 29. ^{1}H and ^{13}C NMR spectra for 3s

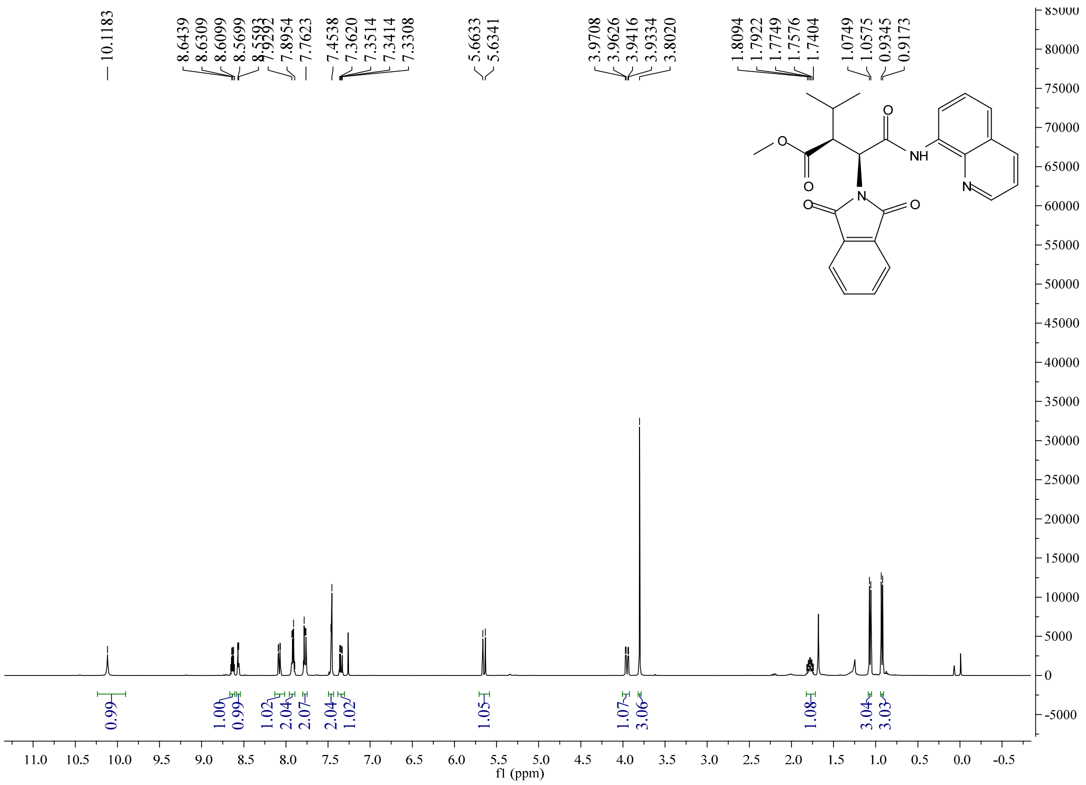

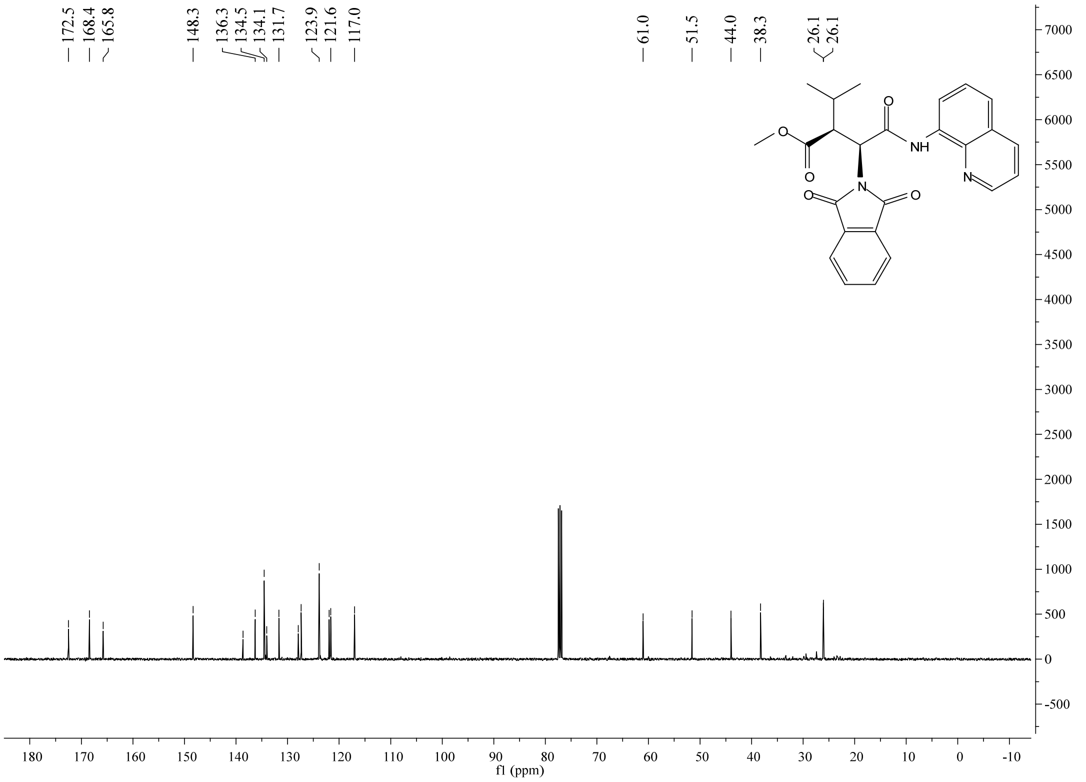

Supplementary Figure 30. ^{1}H and ^{13}C NMR spectra for 3t

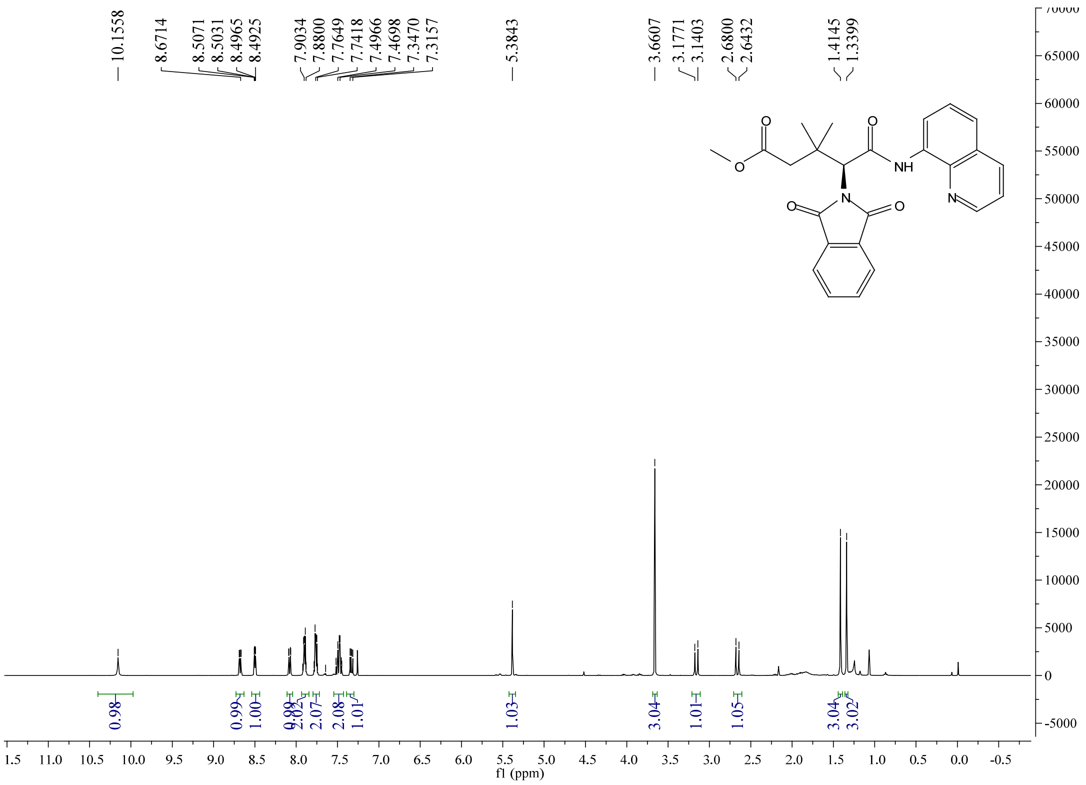

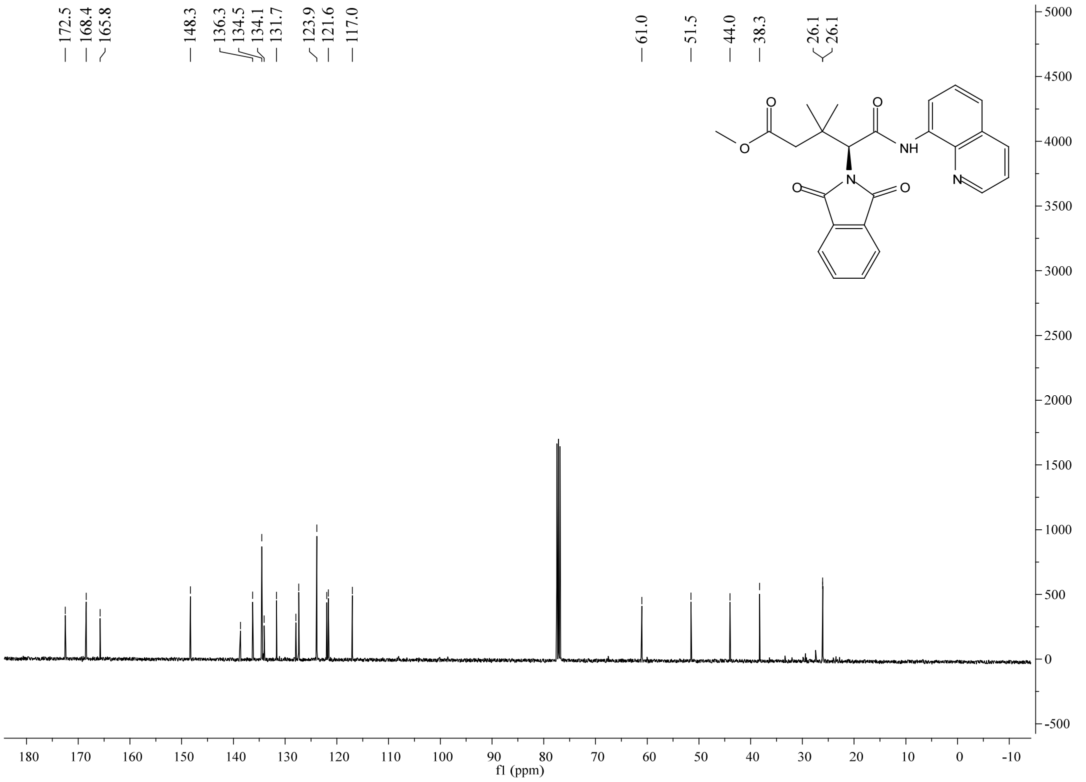

Supplementary Figure 31. ^{1}H and ^{13}C NMR spectra for 3u

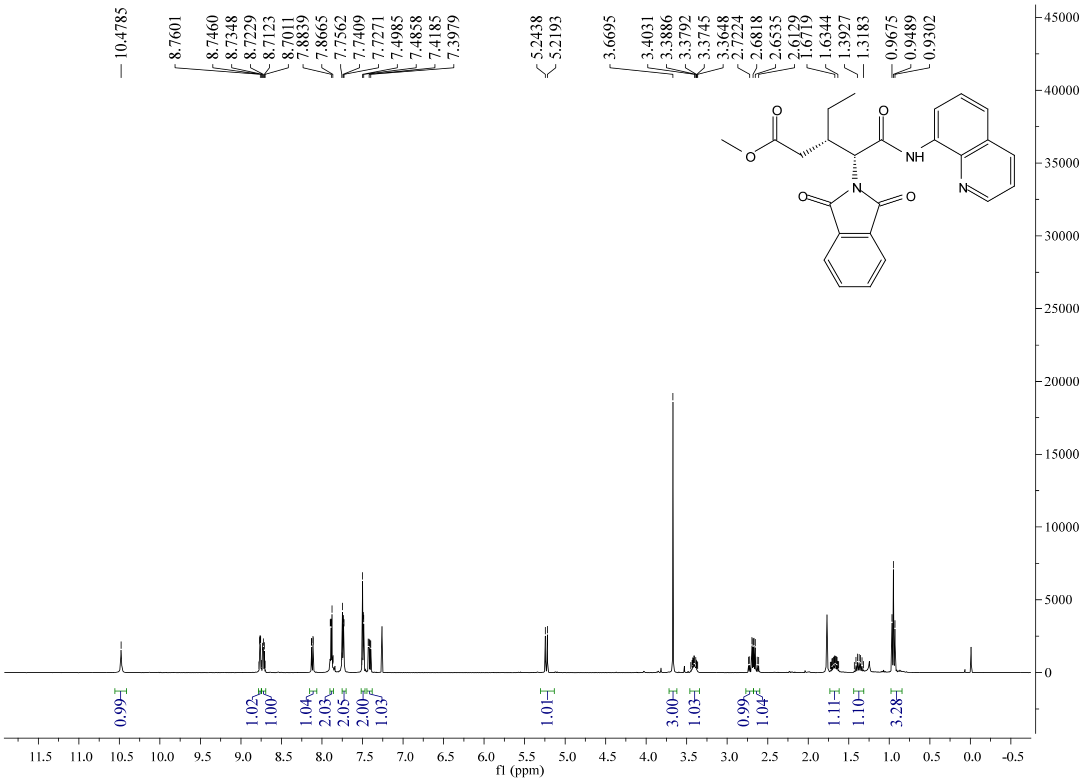

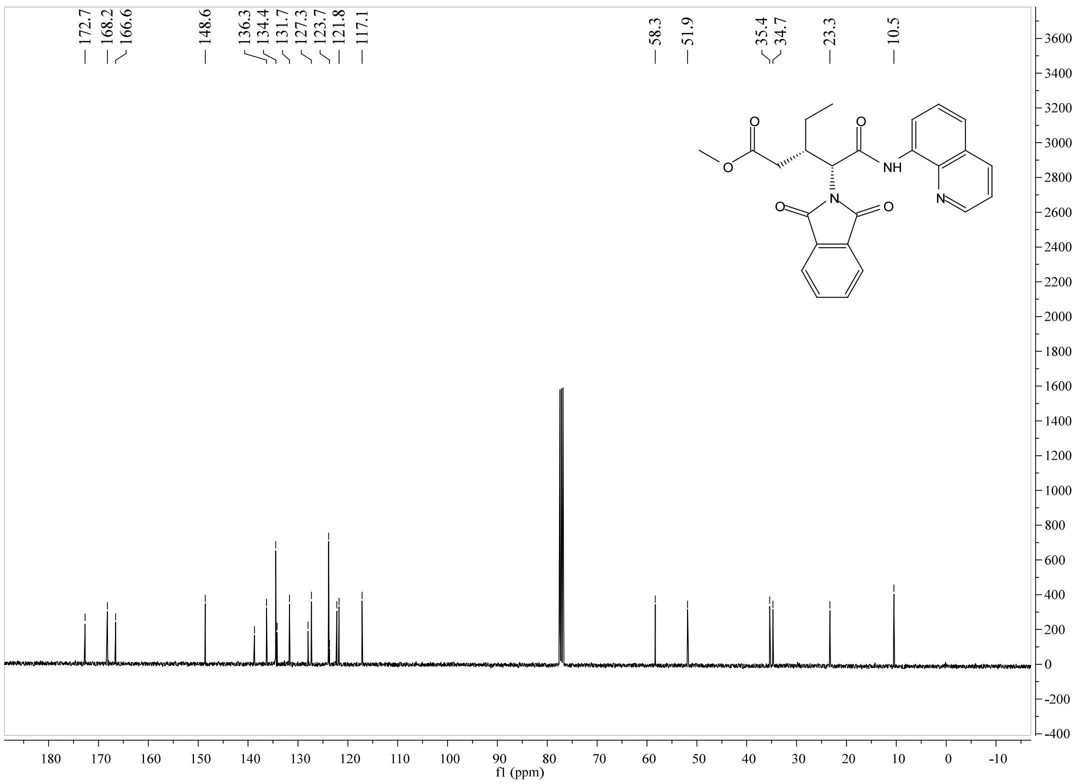

Supplementary Figure 32. ^{1}H and ^{13}C NMR spectra for 3v

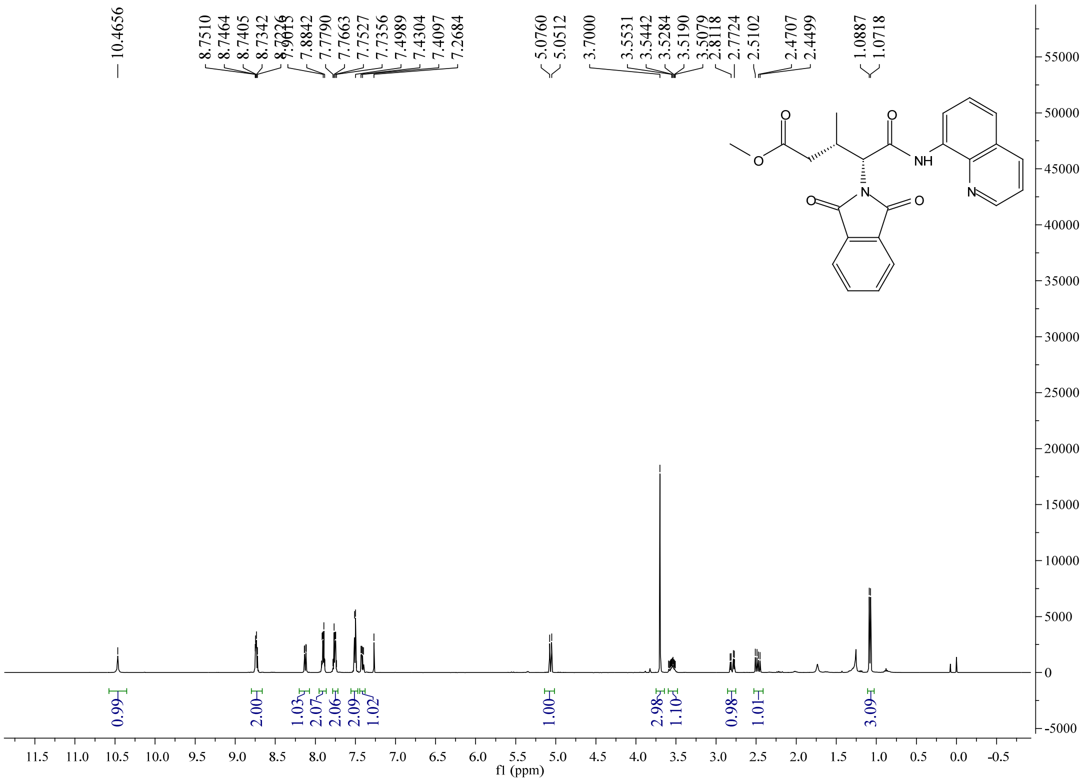

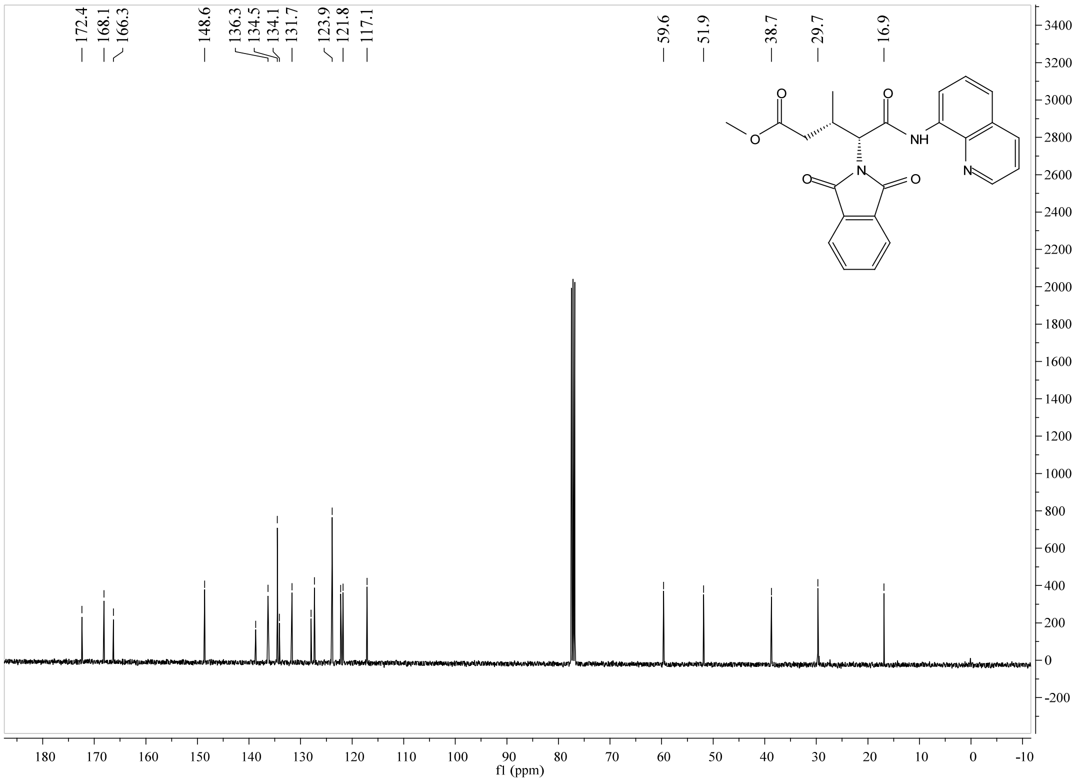

Supplementary Figure 33. ^{1}H and ^{13}C NMR spectra for 3w

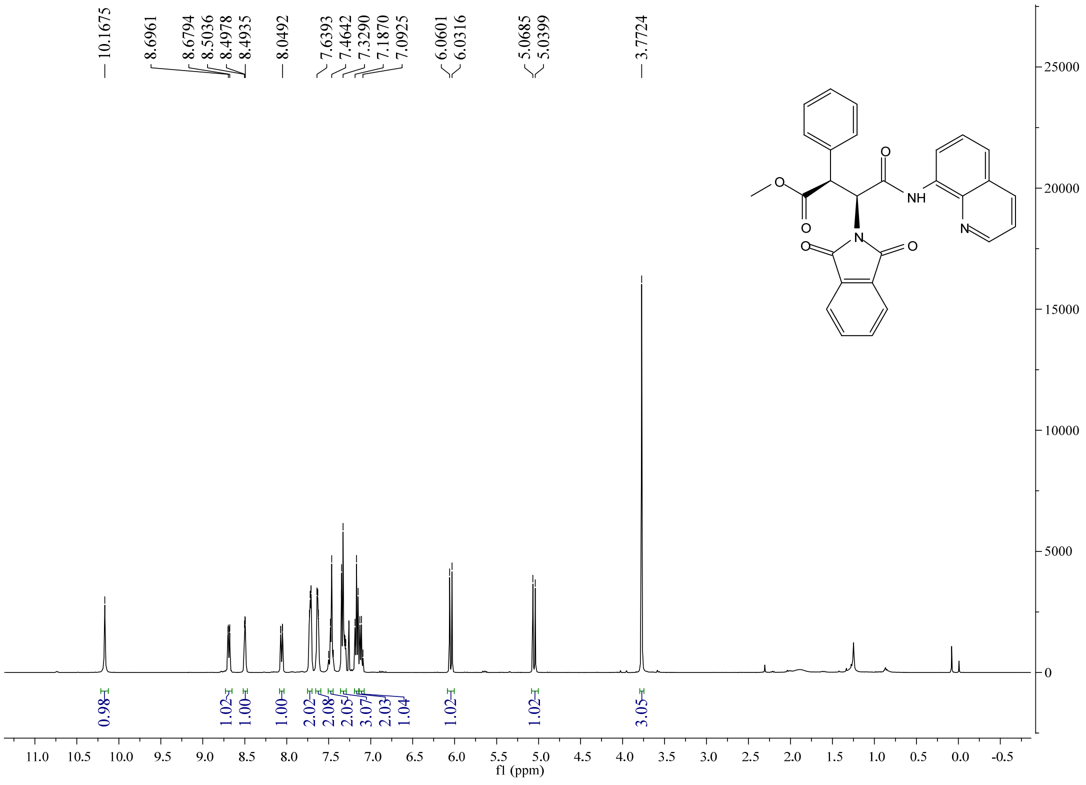

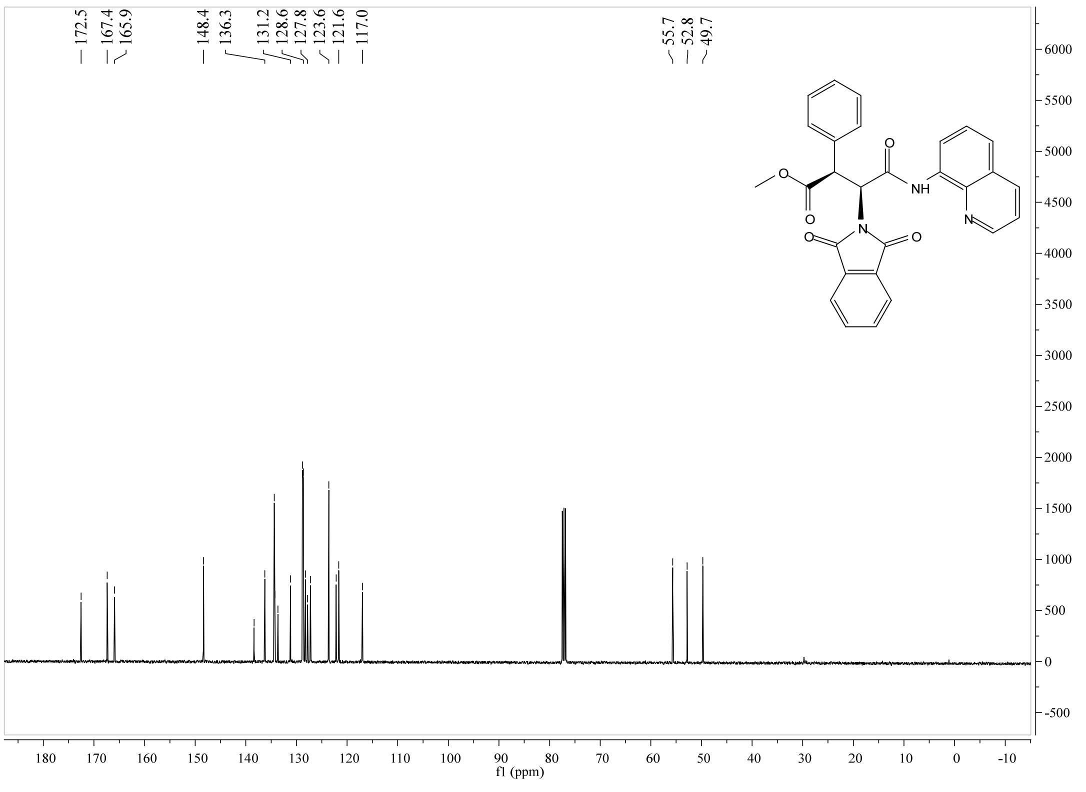

Supplementary Figure 34. ^{1}H and ^{13}C NMR spectra for 4b

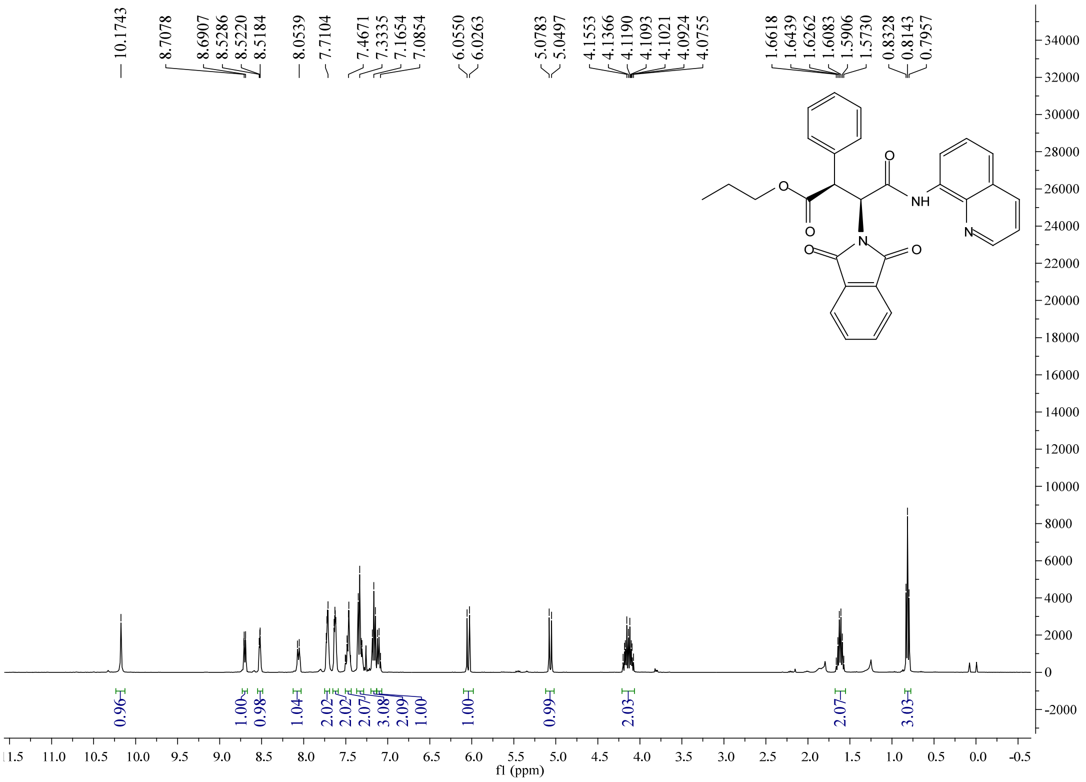

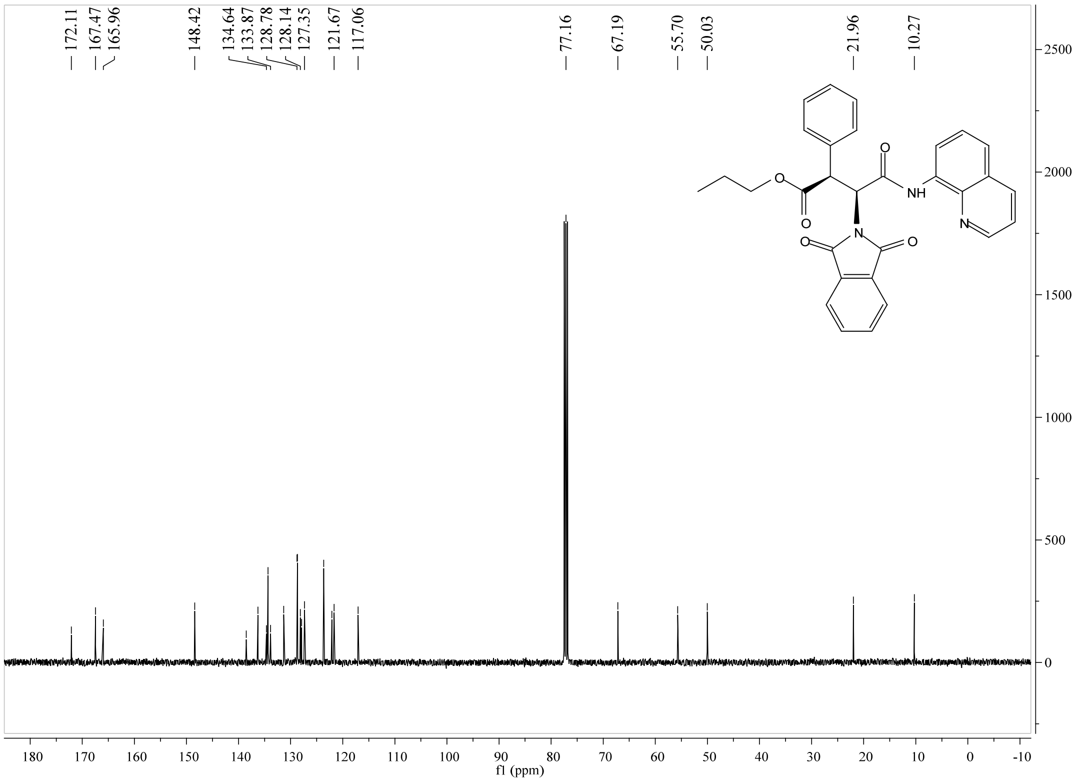

Supplementary Figure 35. ^{1}H and ^{13}C NMR spectra for 4c

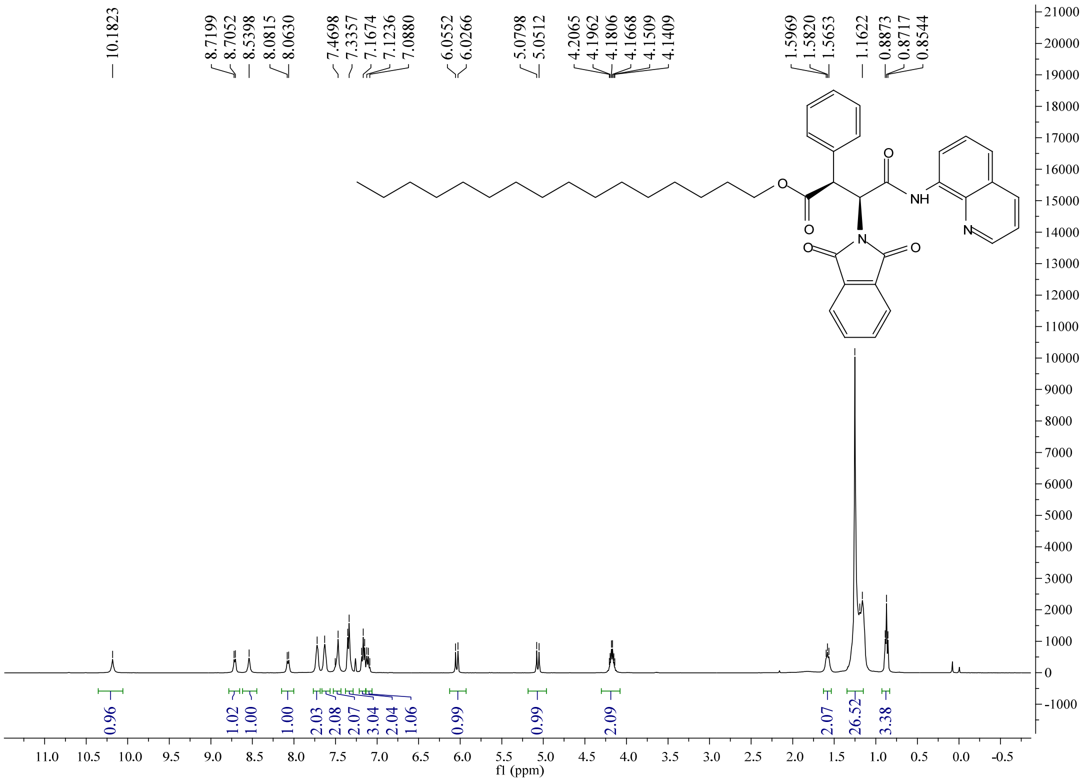

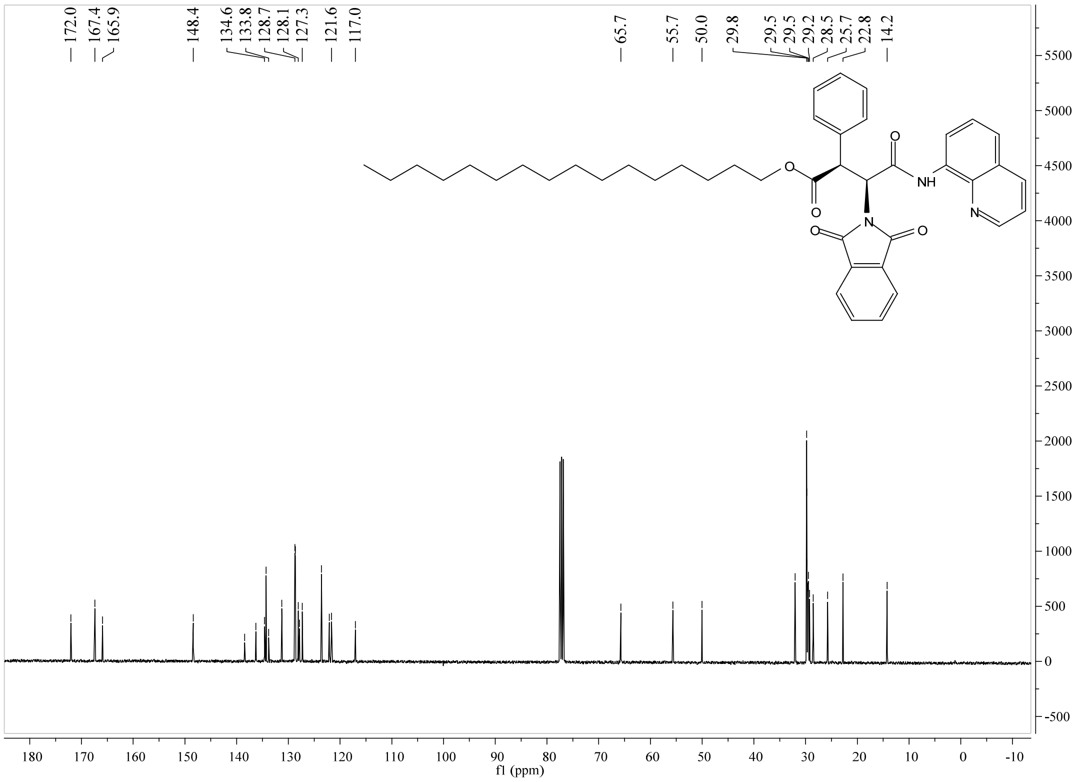

Supplementary Figure 36. ^{1}H and ^{13}C NMR spectra for 4d

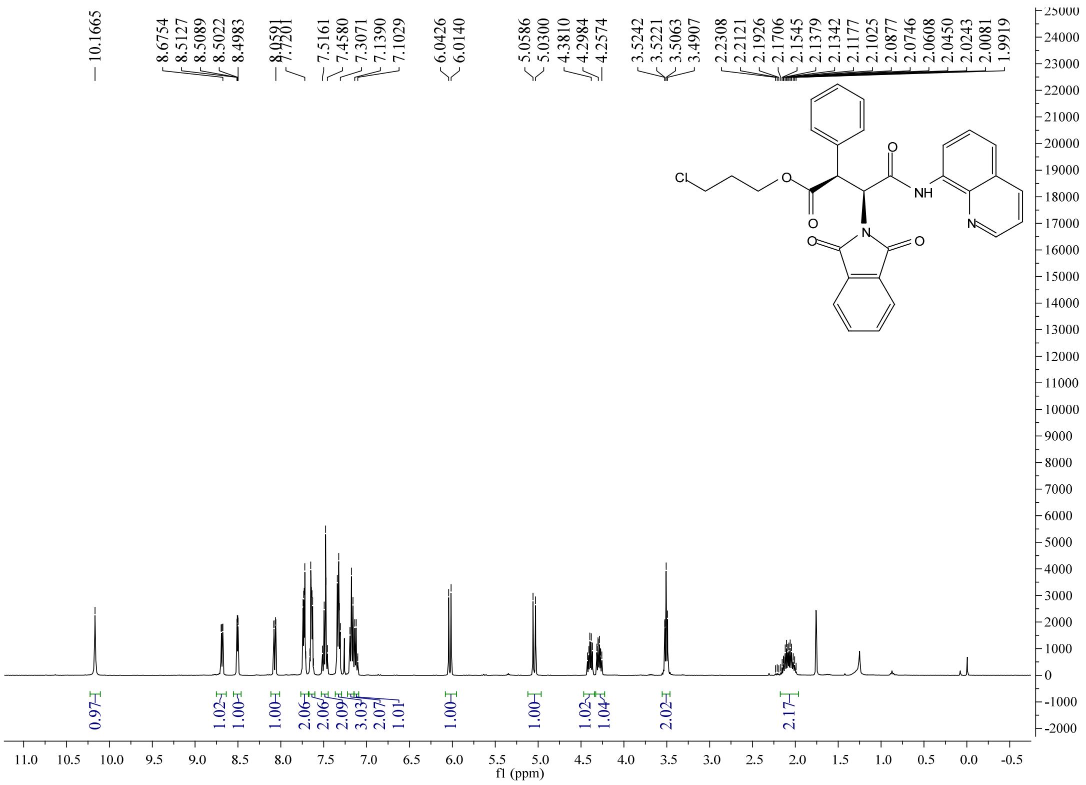

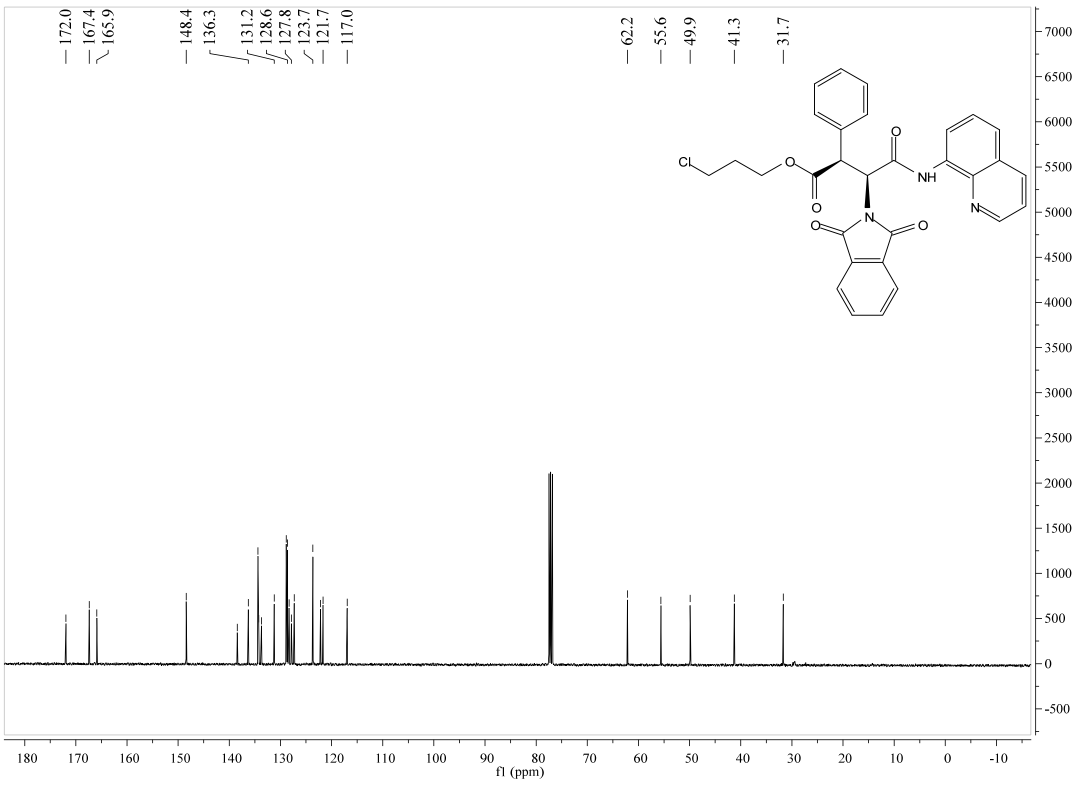

Supplementary Figure 37. ^{1}H and ^{13}C NMR spectra for 4e

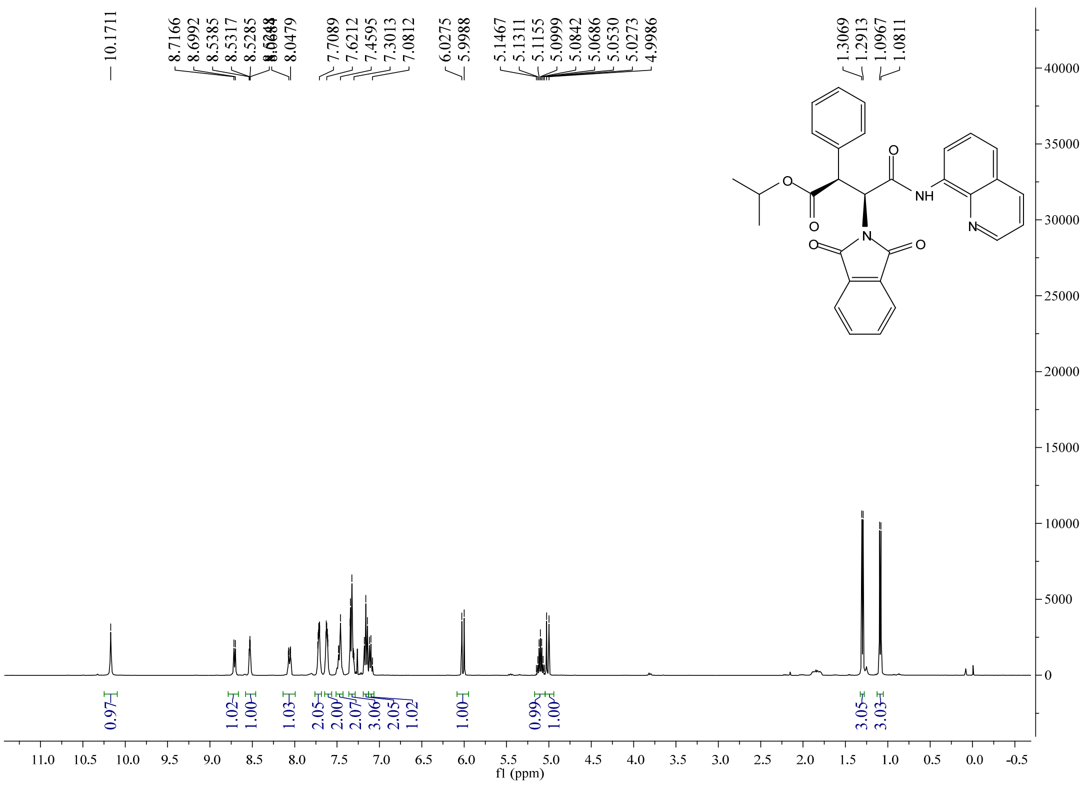

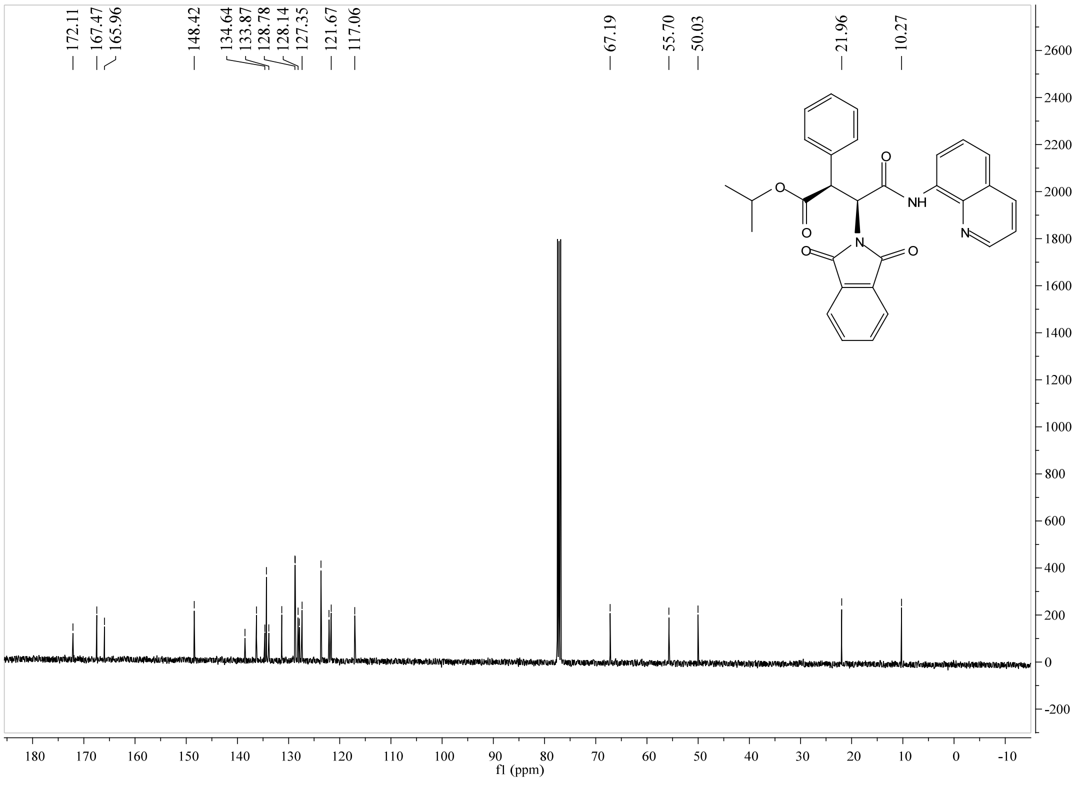

Supplementary Figure 38. ^{1}H and ^{13}C NMR spectra for 4f

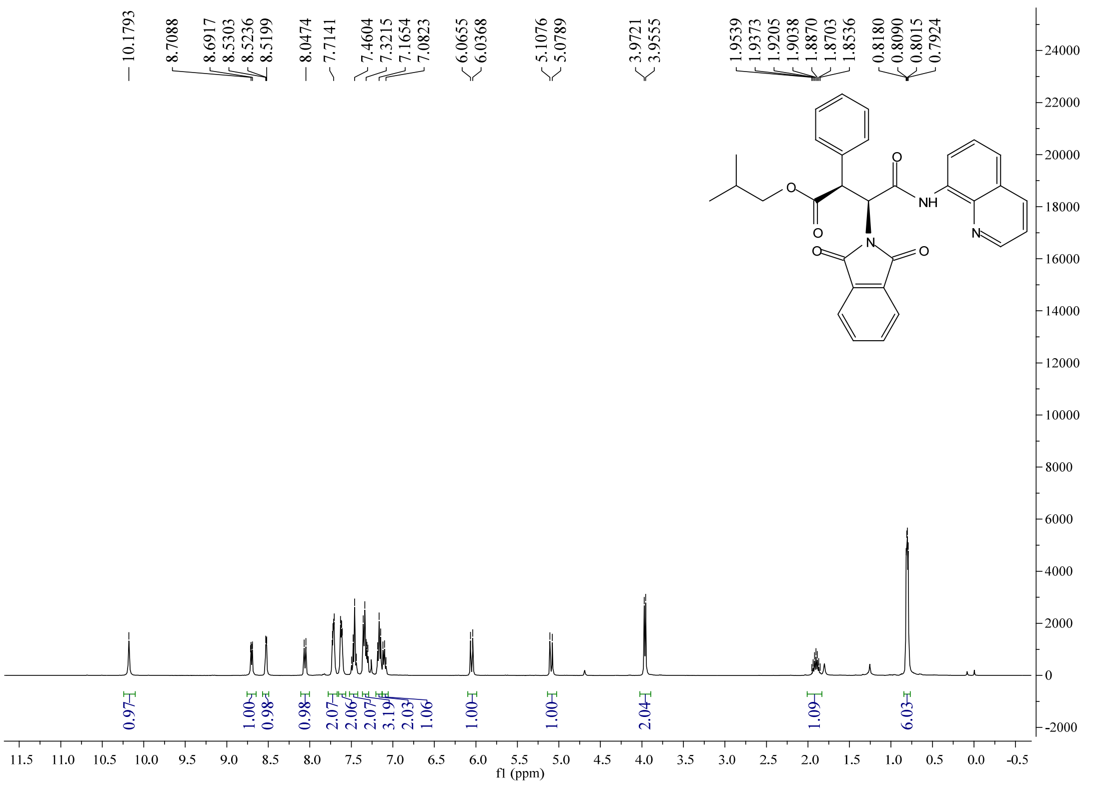

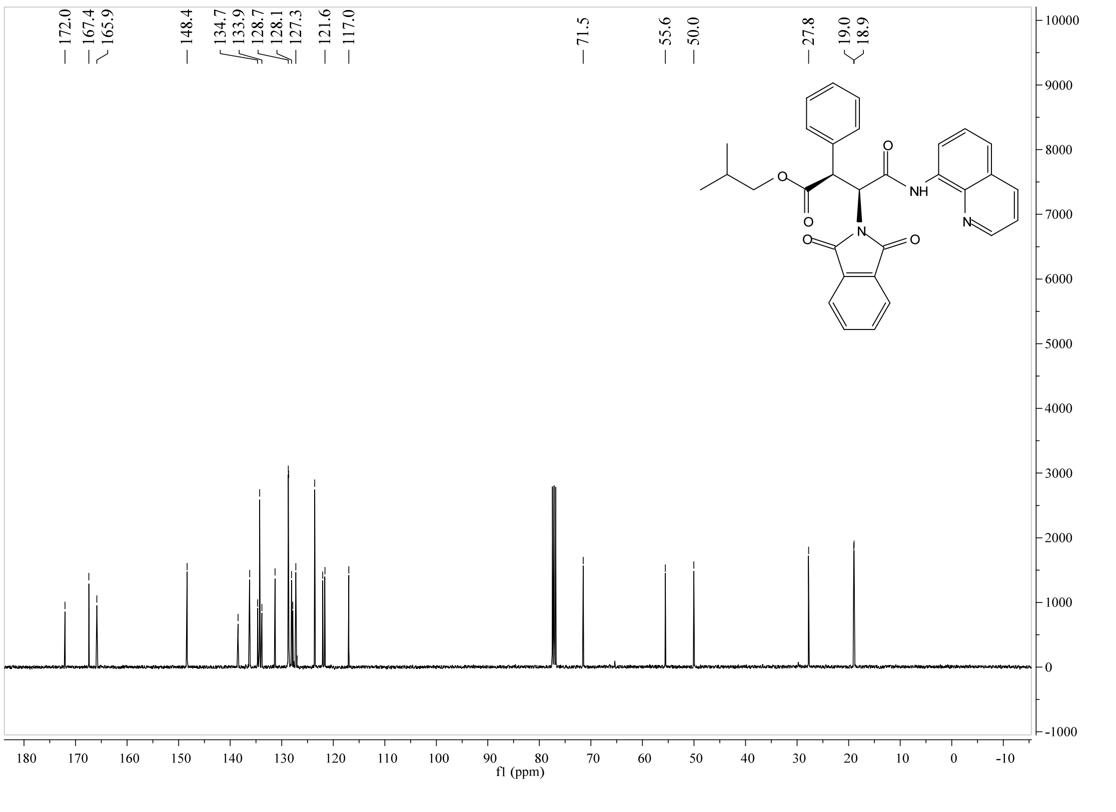

Supplementary Figure 39. ^{1}H and ^{13}C NMR spectra for 4g

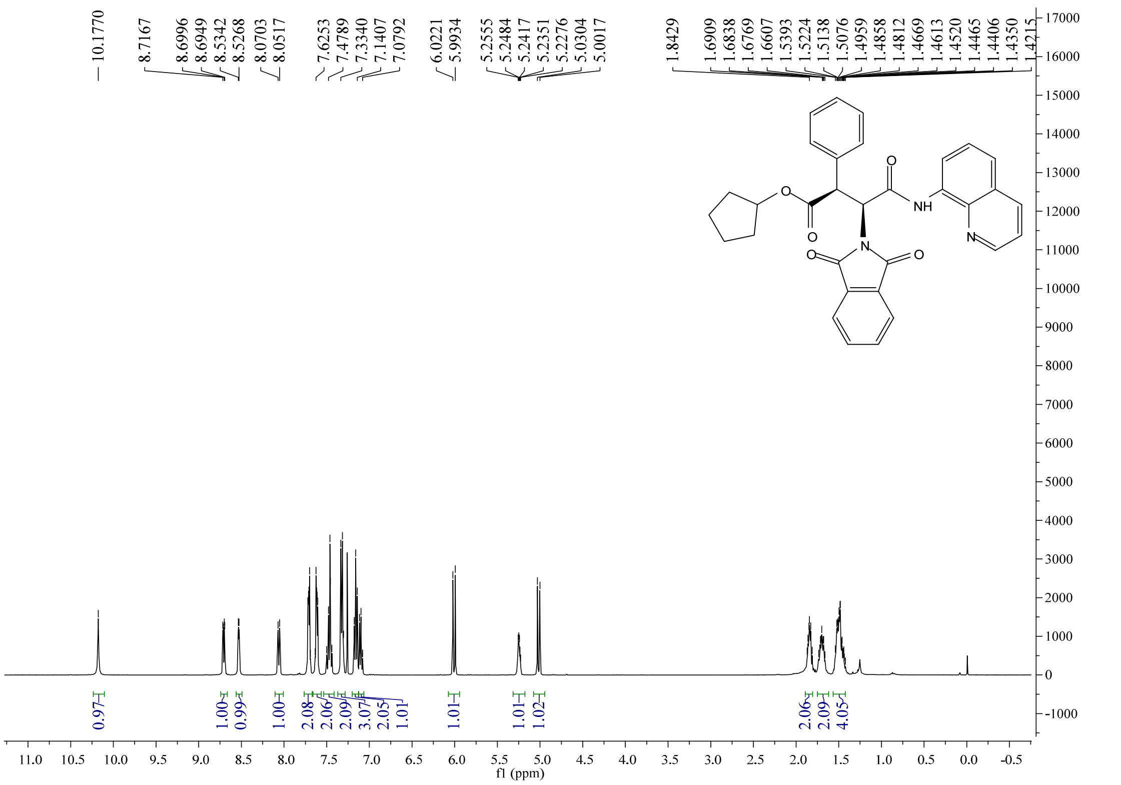

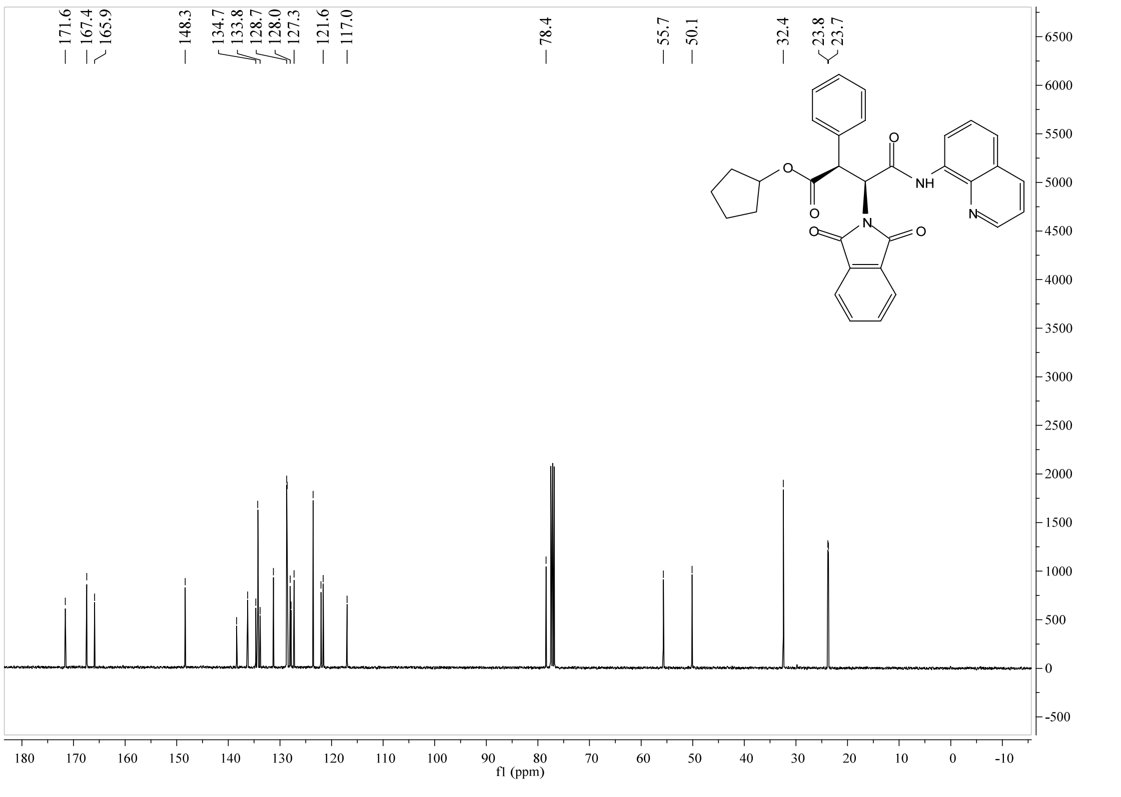

Supplementary Figure 40. ^{1}H and ^{13}C NMR spectra for 4h

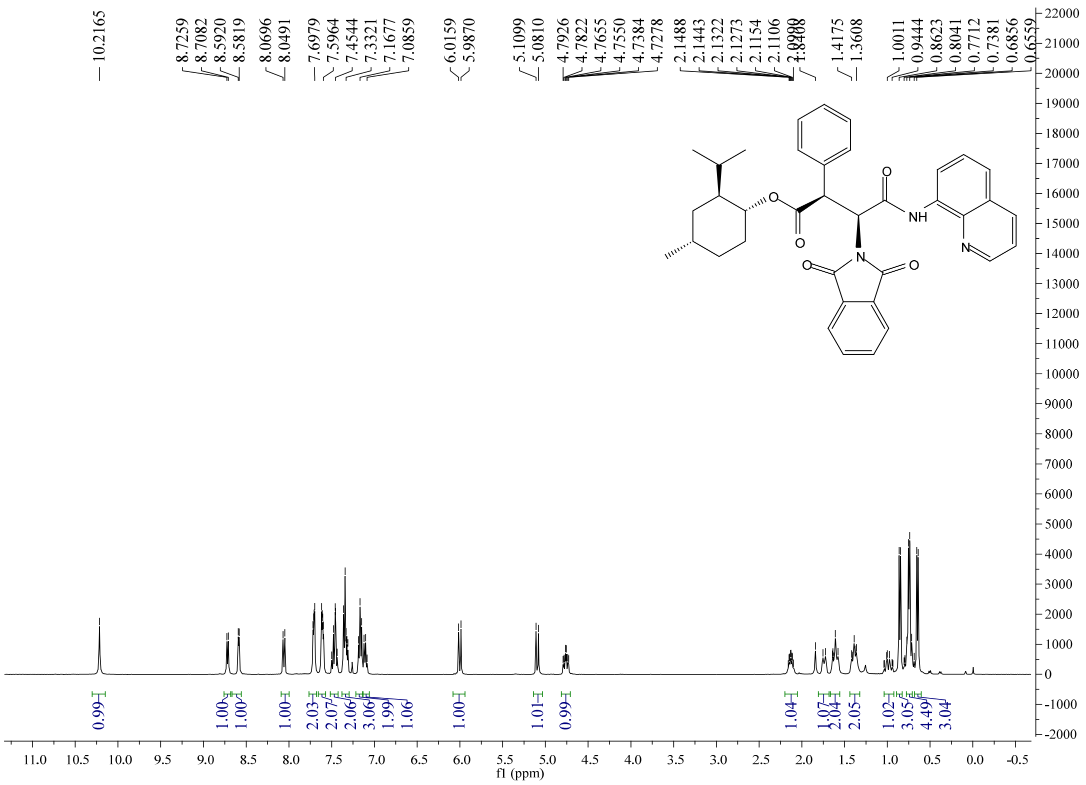

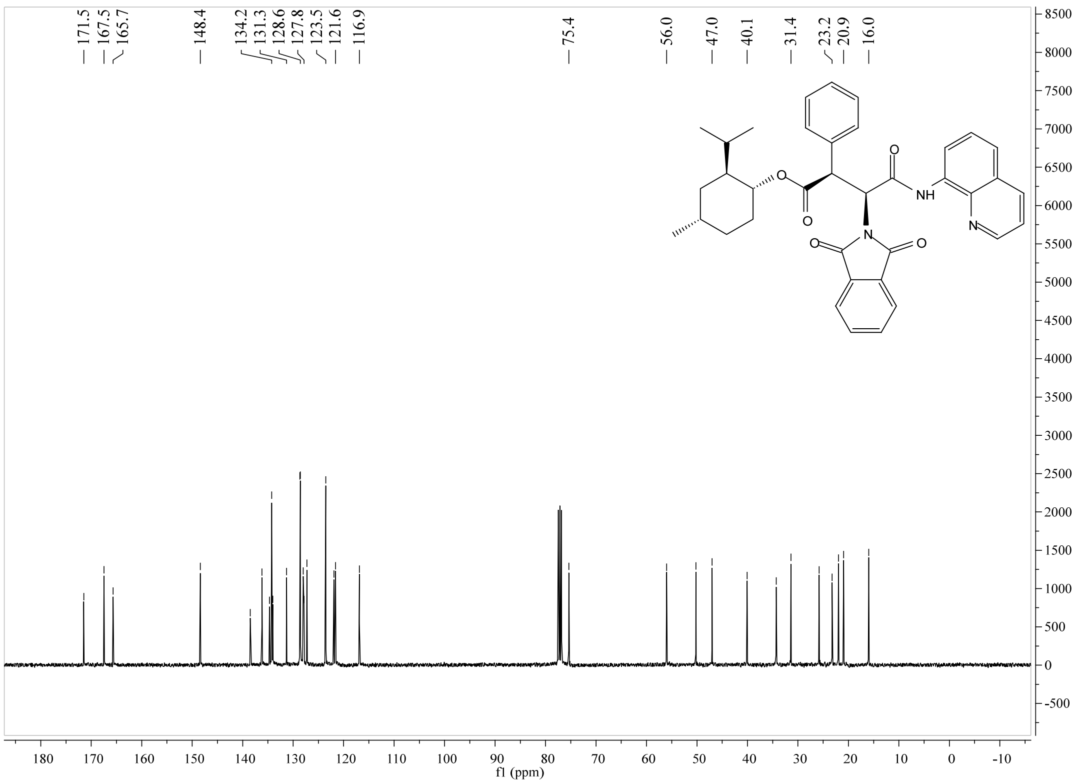

Supplementary Figure 41. ^{1}H and ^{13}C NMR spectra for 4i

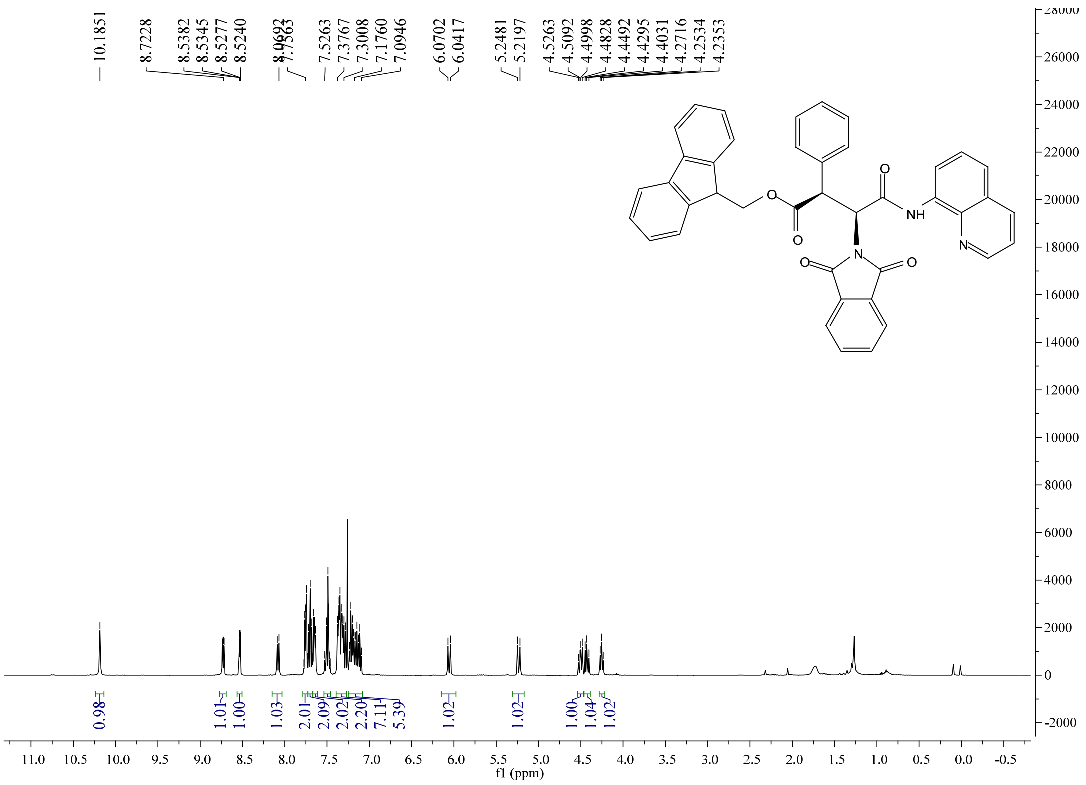

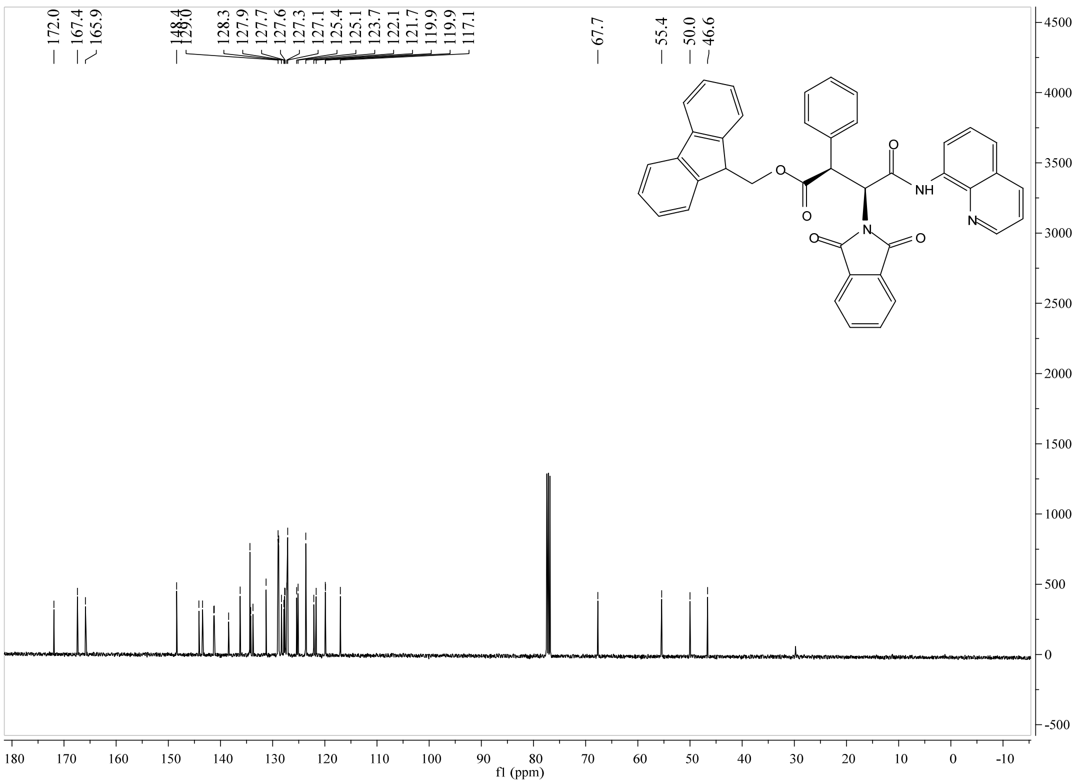

Supplementary Figure 42. ^{1}H and ^{13}C NMR spectra for 4j

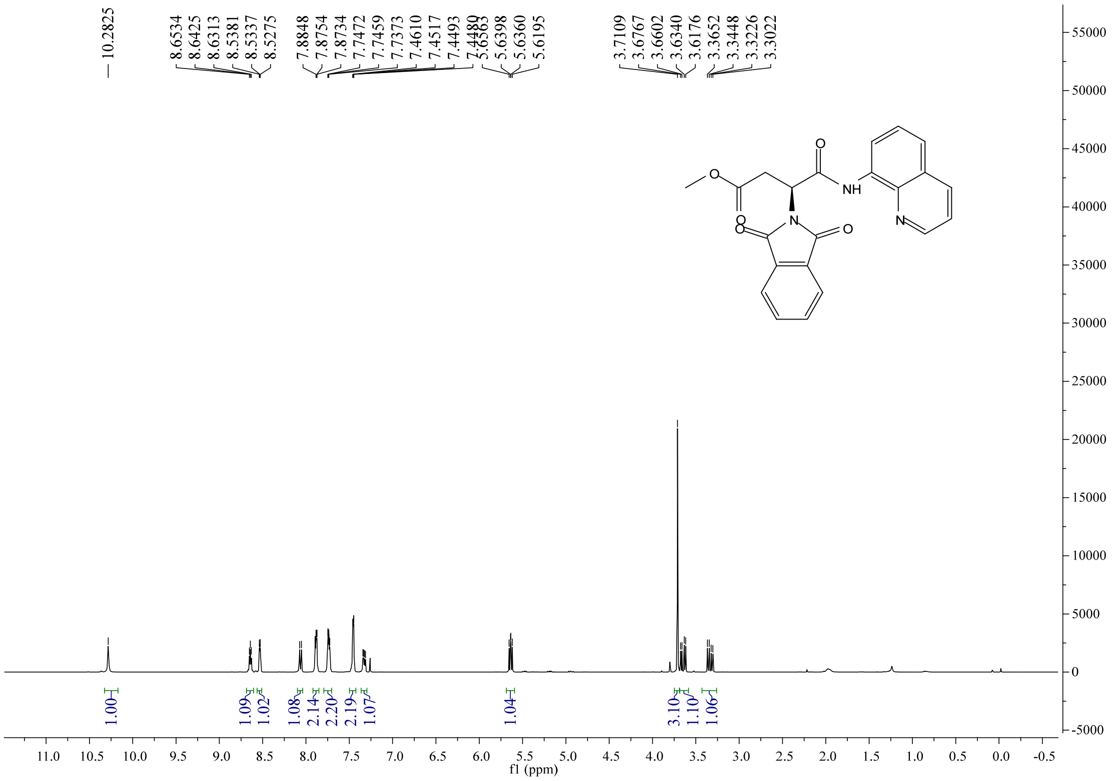

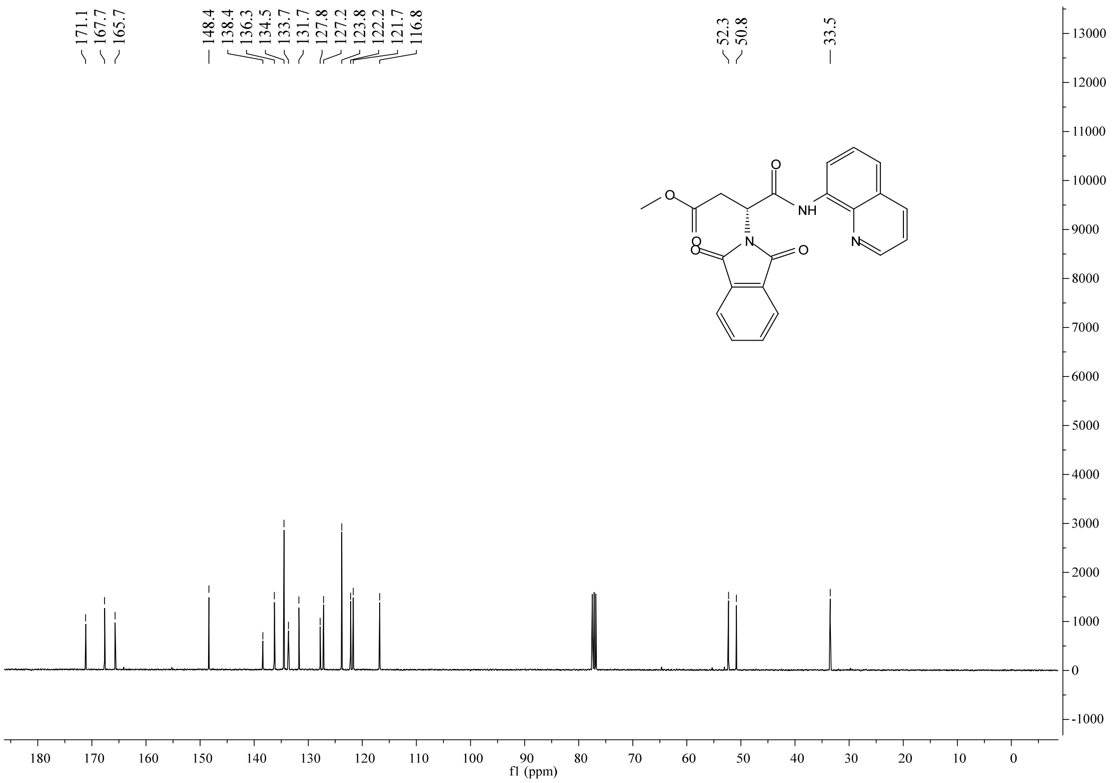

Supplementary Figure 43. ^{1}H and ^{13}C NMR spectra for 6a

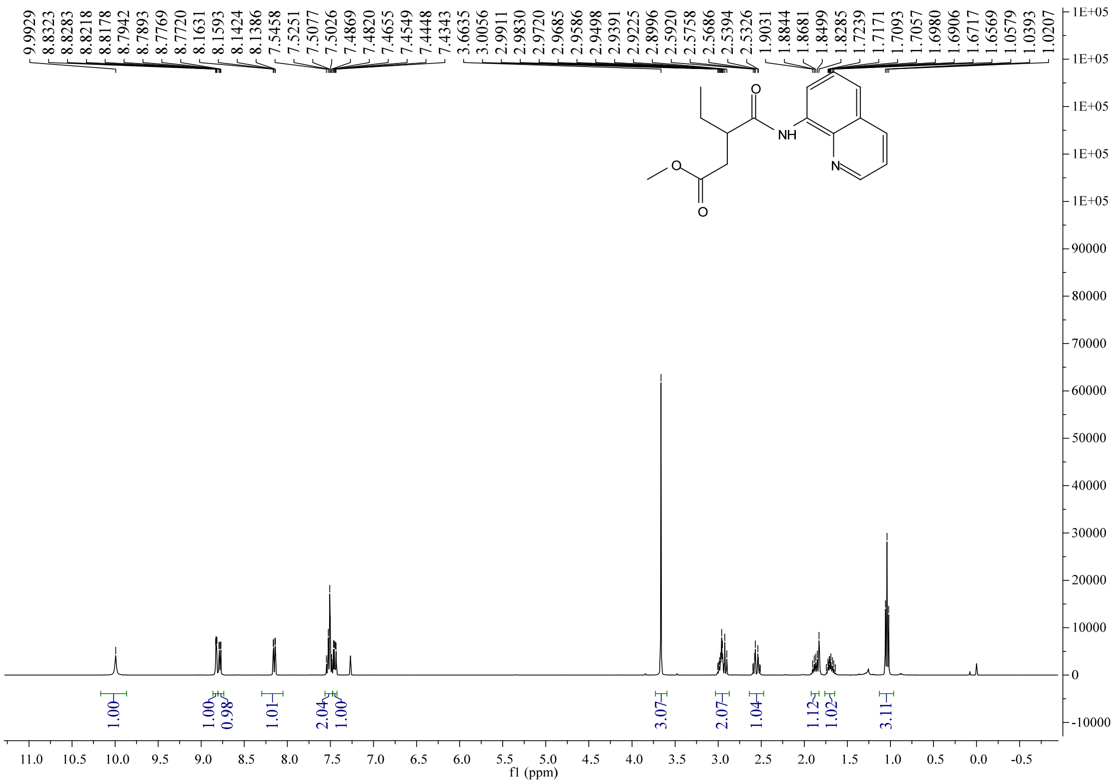

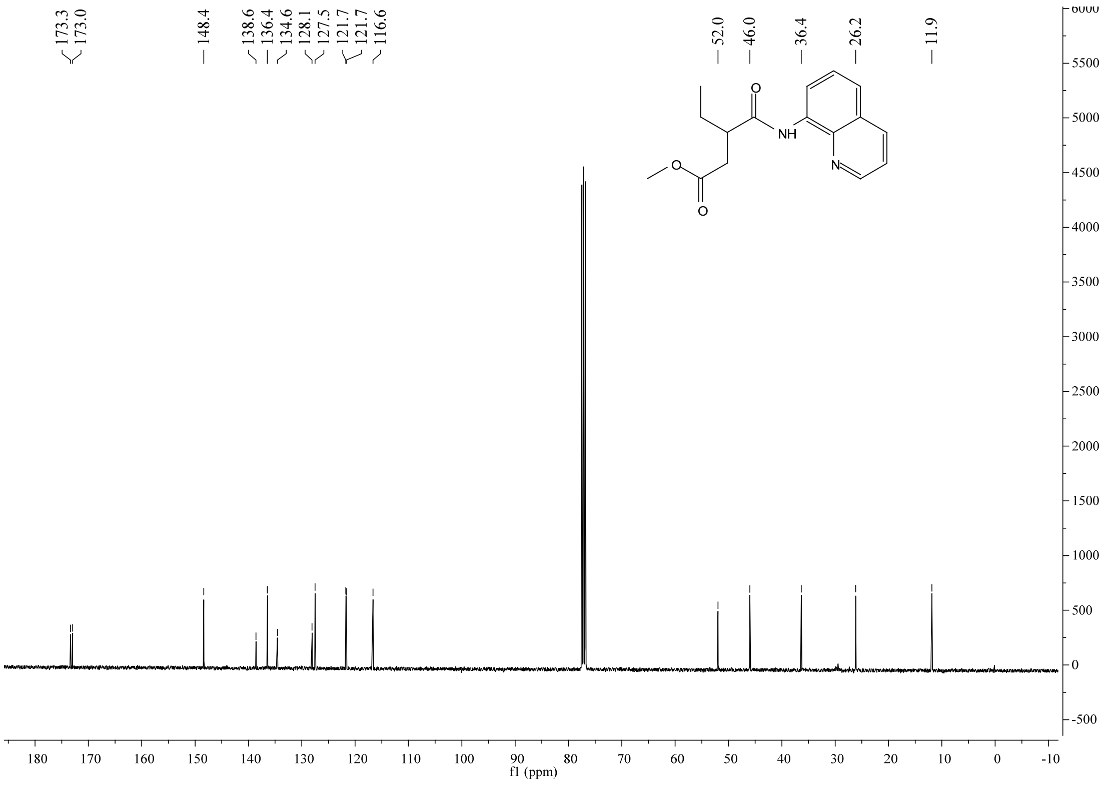

Supplementary Figure 44. ^{1}H and ^{13}C NMR spectra for 6b

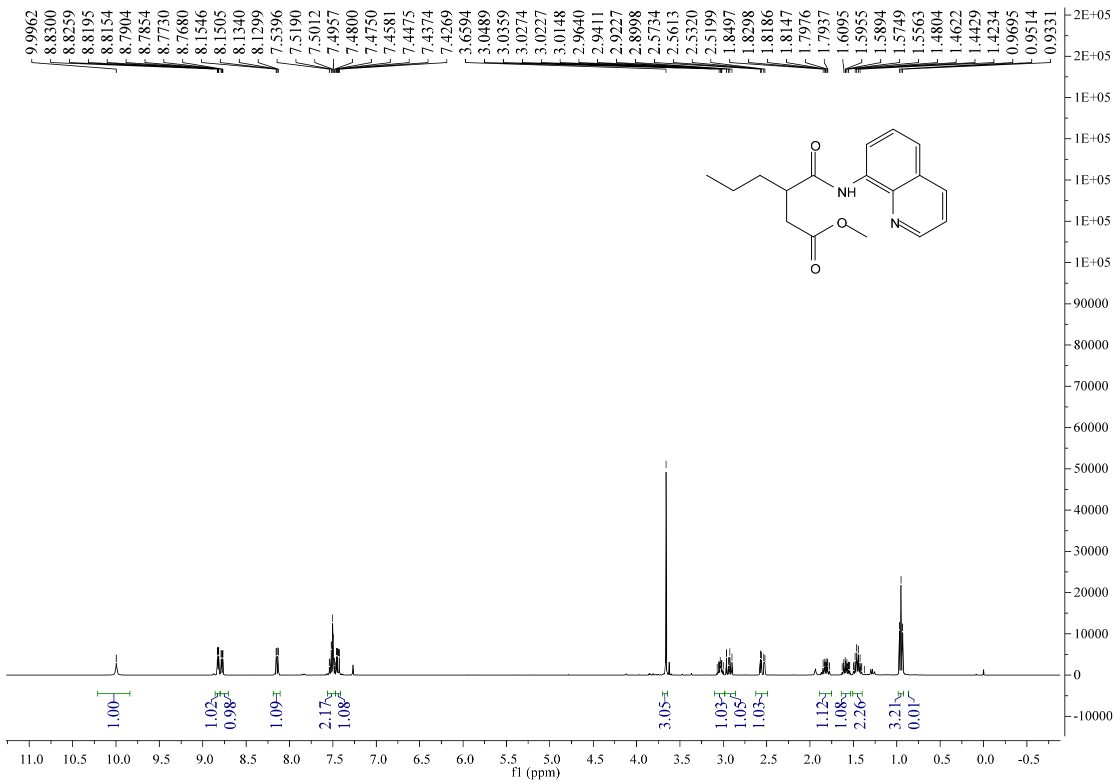

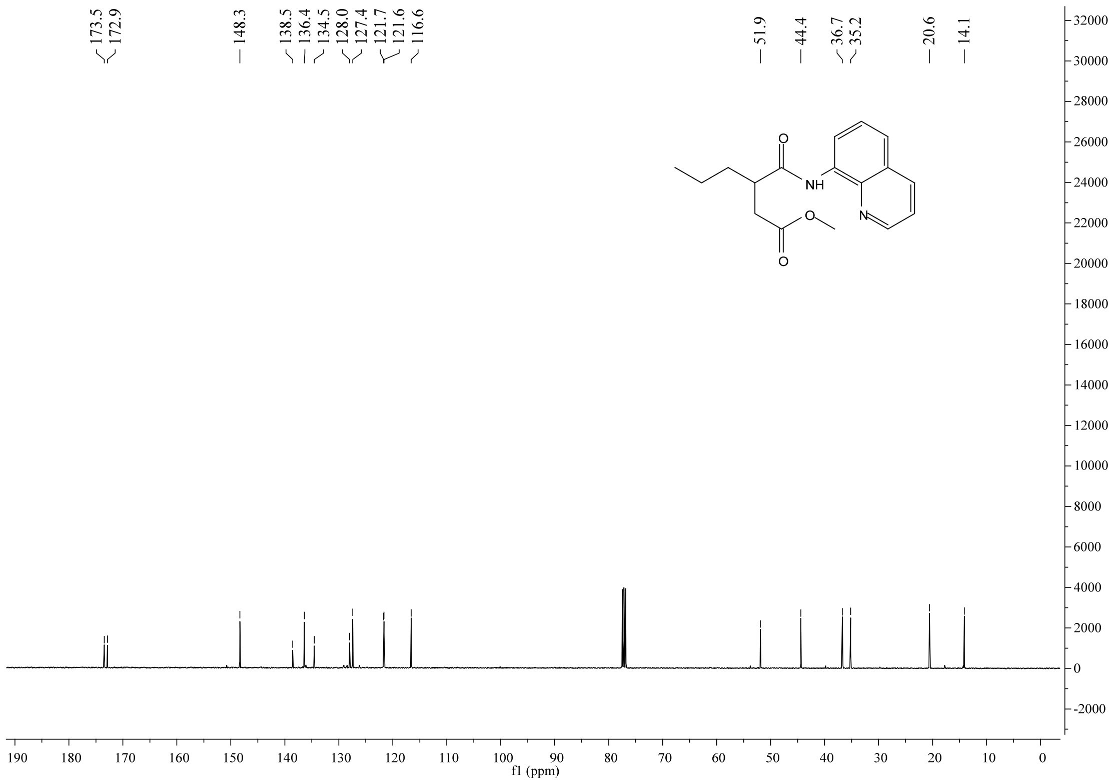

Supplementary Figure 45. ^{1}H and ^{13}C NMR spectra for 6c

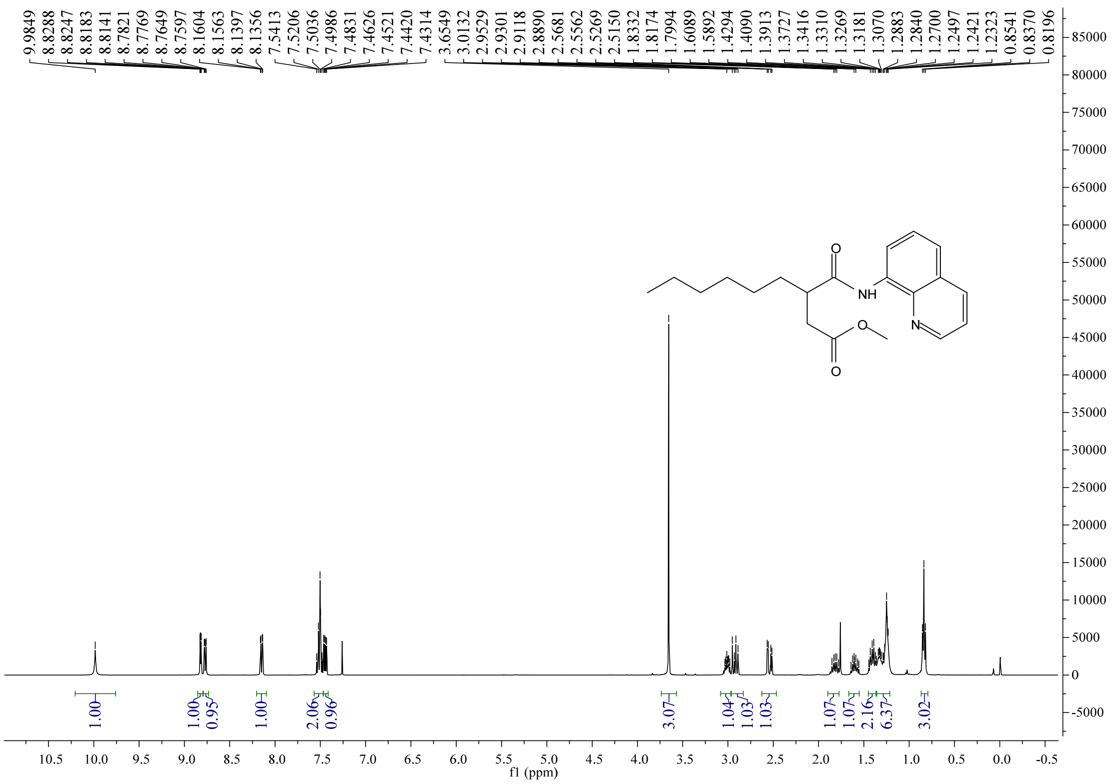

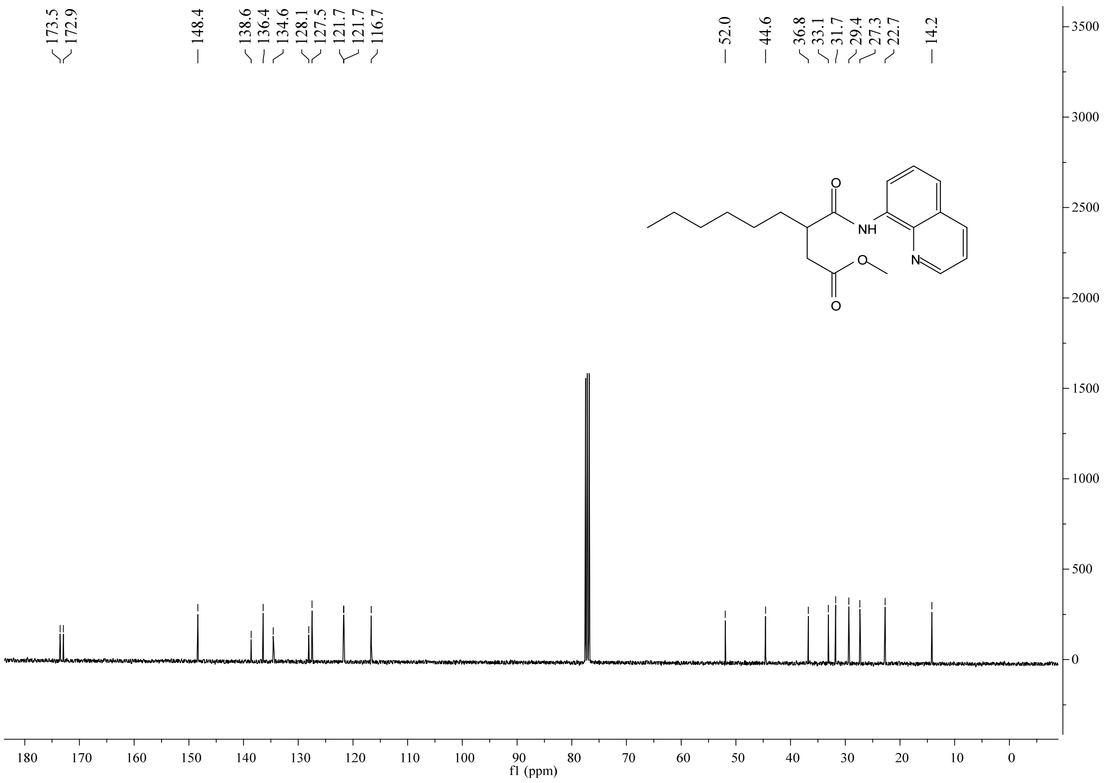

Supplementary Figure 46. ^{1}H and ^{13}C NMR spectra for 6d

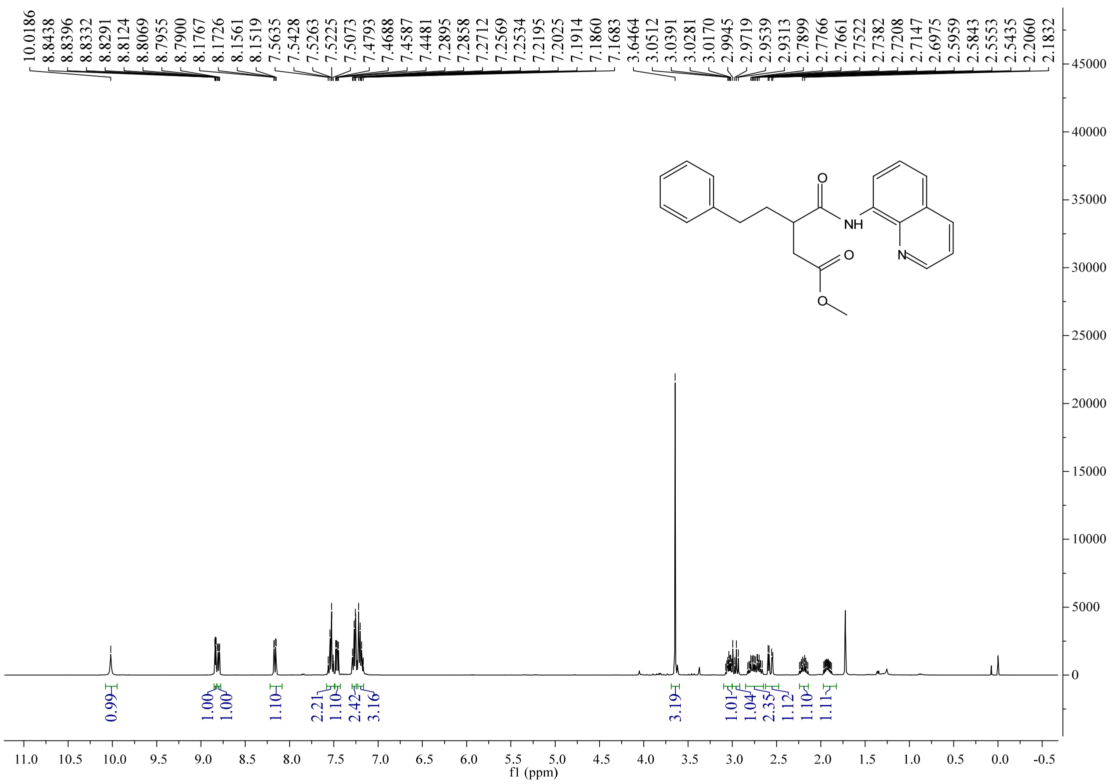

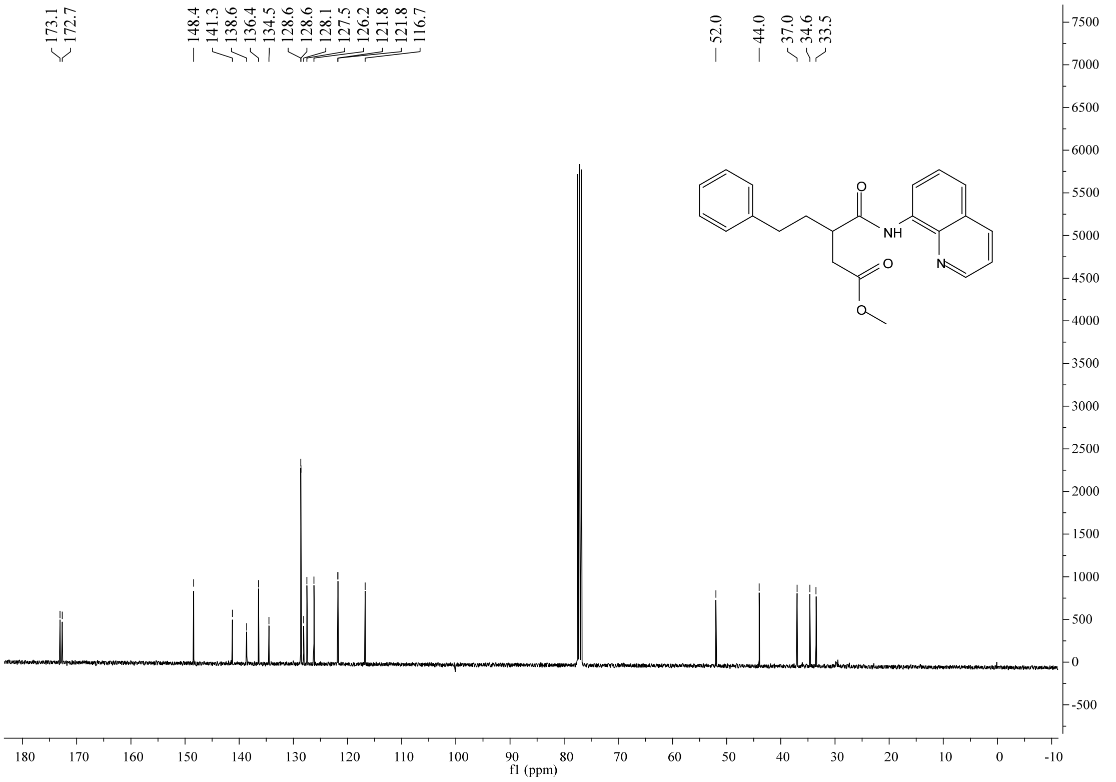

Supplementary Figure 47. ^{1}H and ^{13}C NMR spectra for 6e

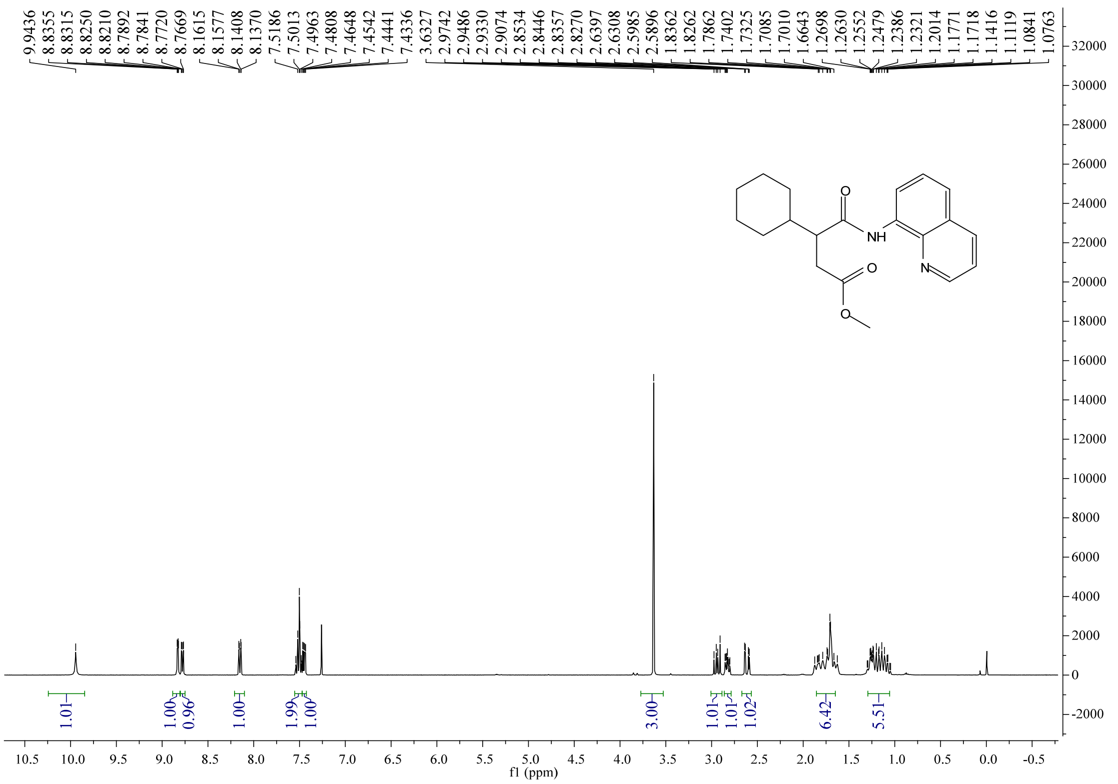

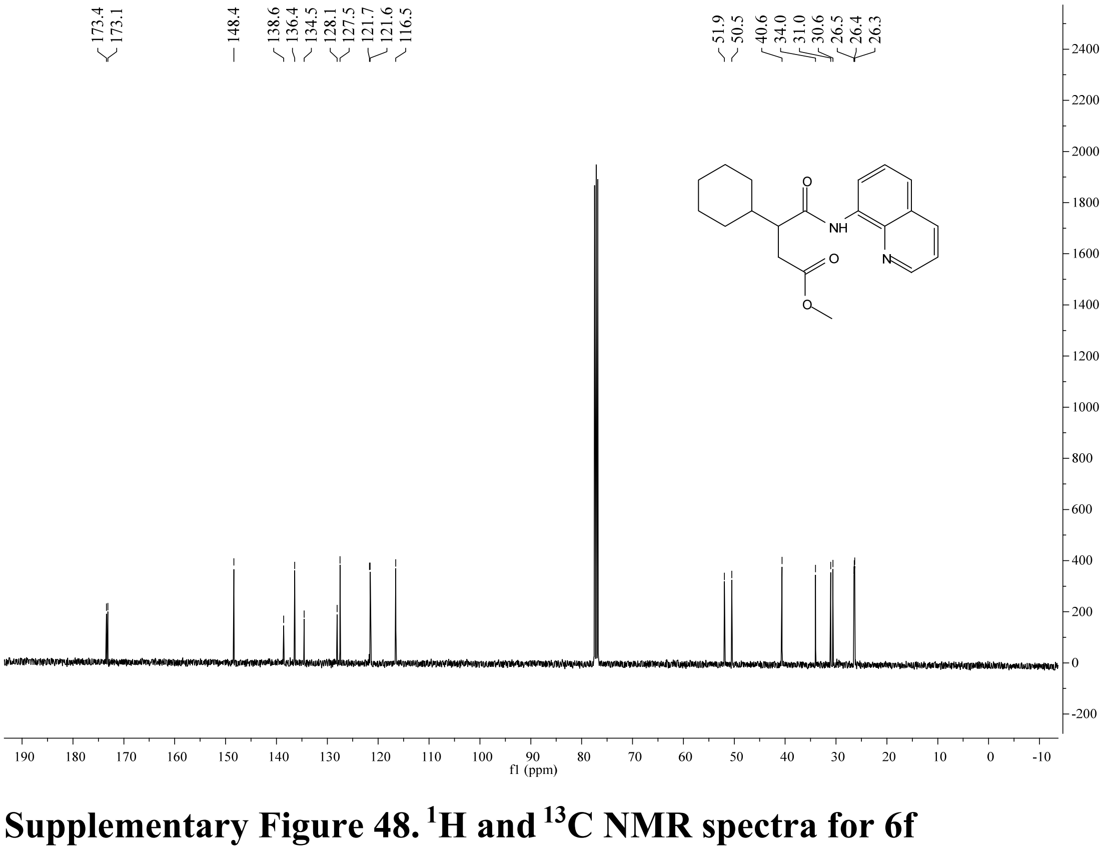

Supplementary Figure 48. ^{1}**H and** ^{13}**C NMR spectra for 6f**

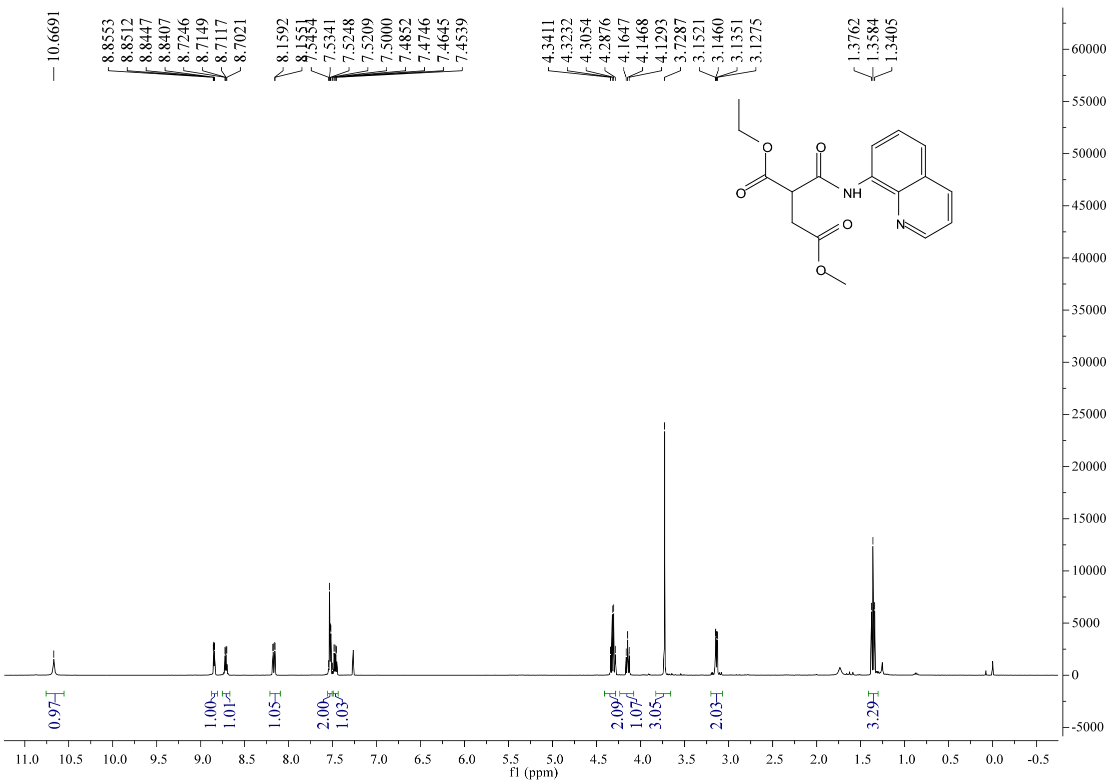

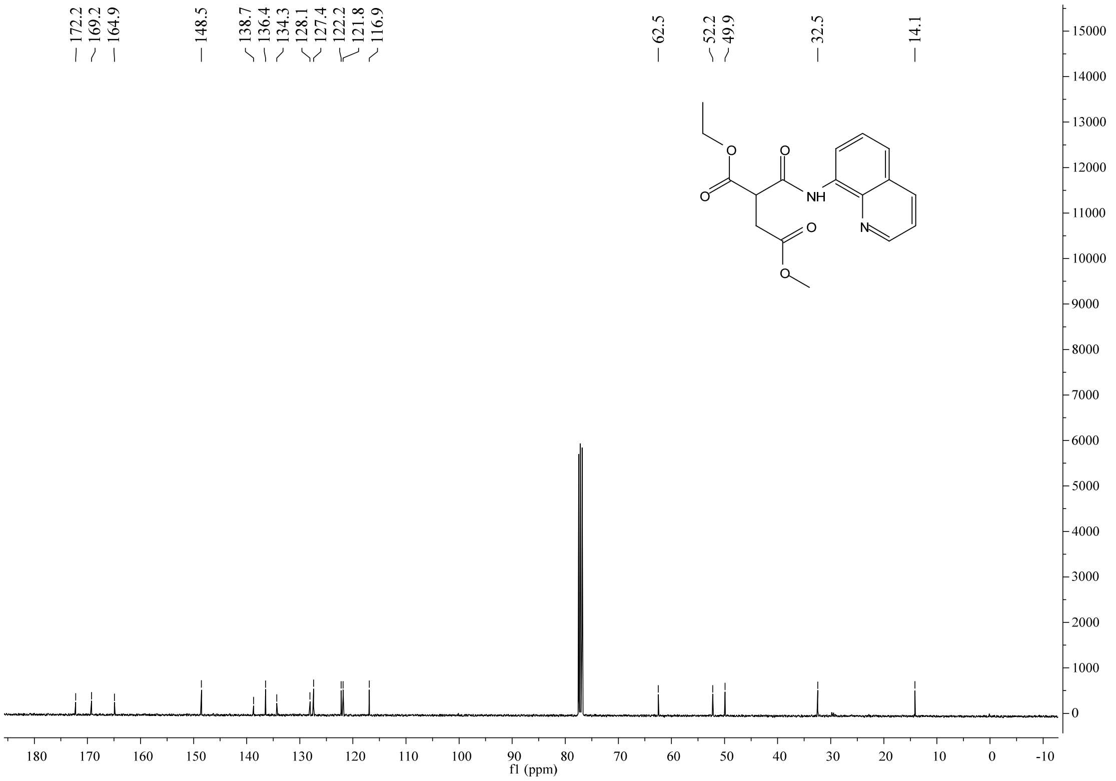

Supplementary Figure 49. ^{1}H and ^{13}C NMR spectra for 6g

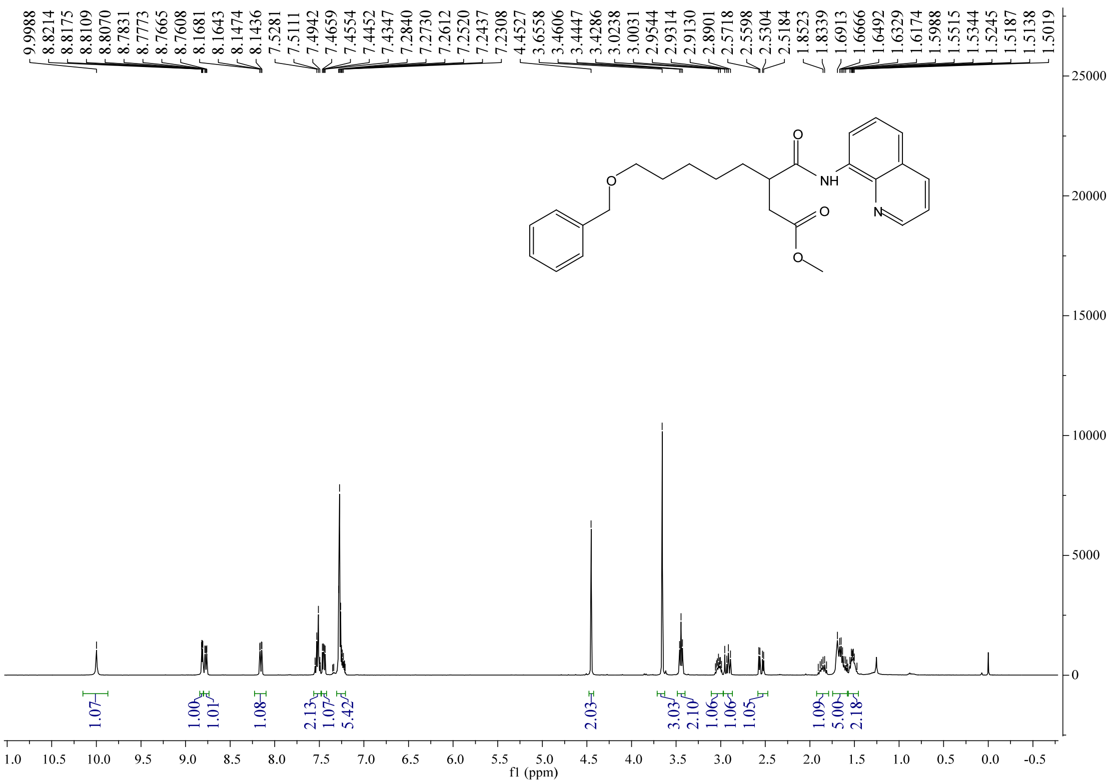

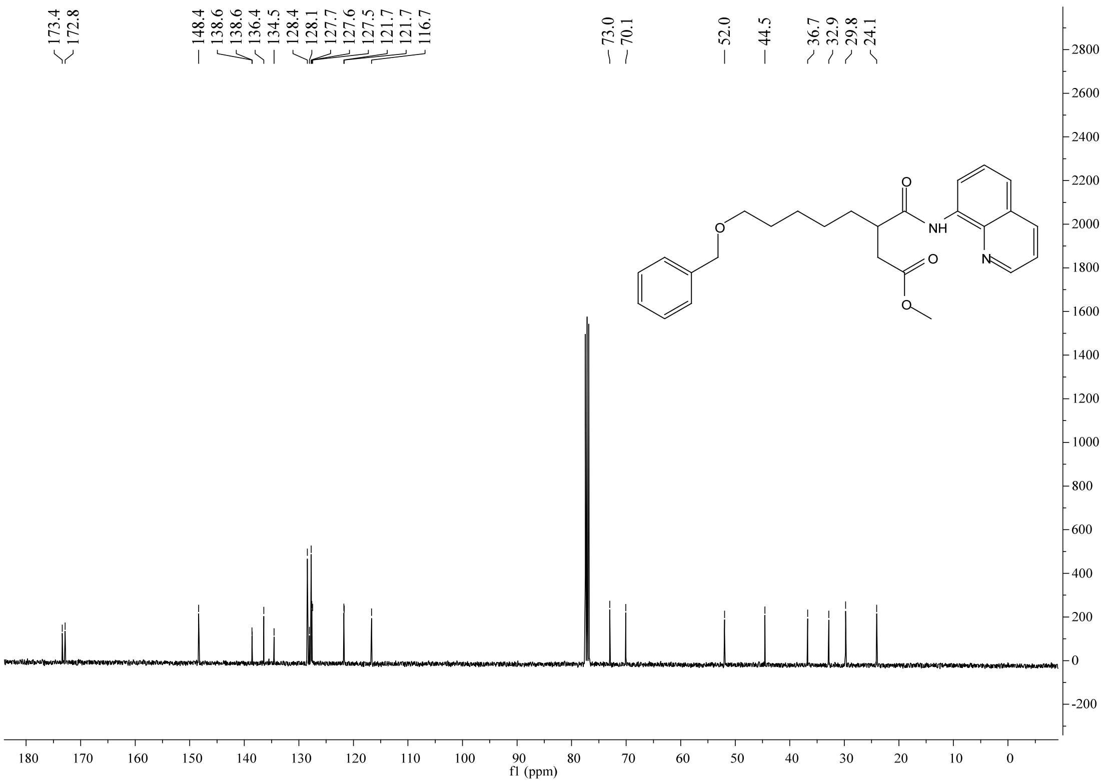

Supplementary Figure 50. ^{1}H and ^{13}C NMR spectra for 6h

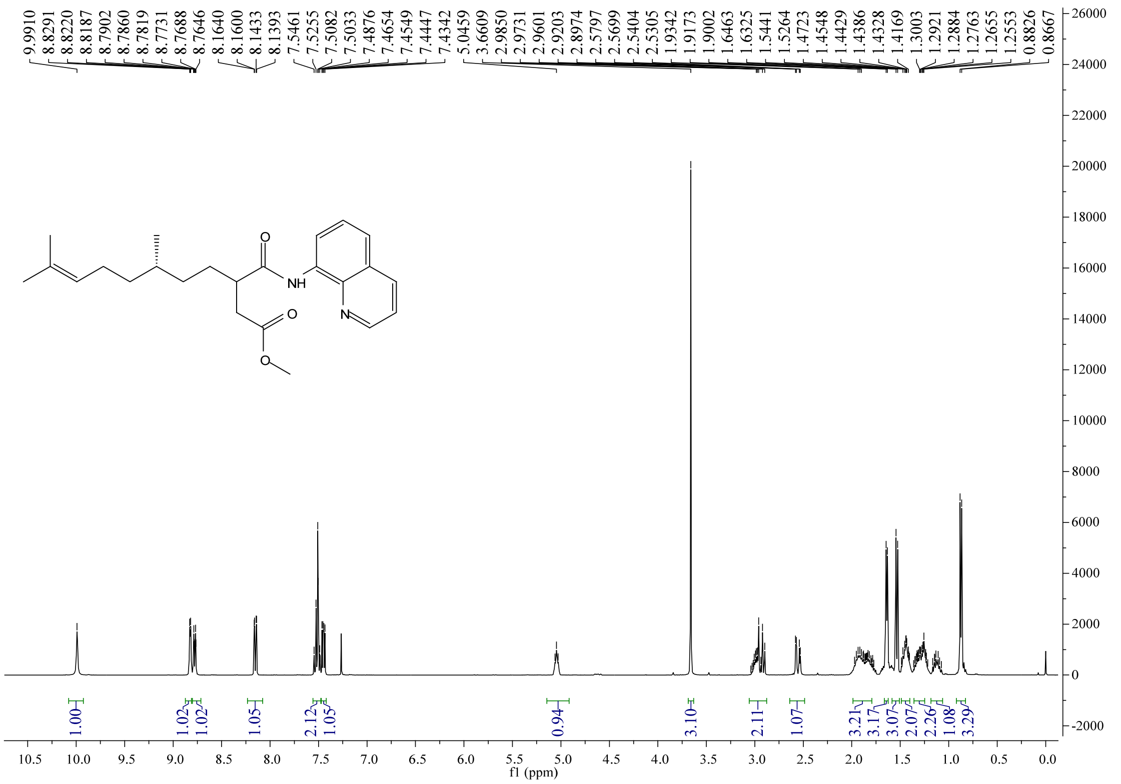

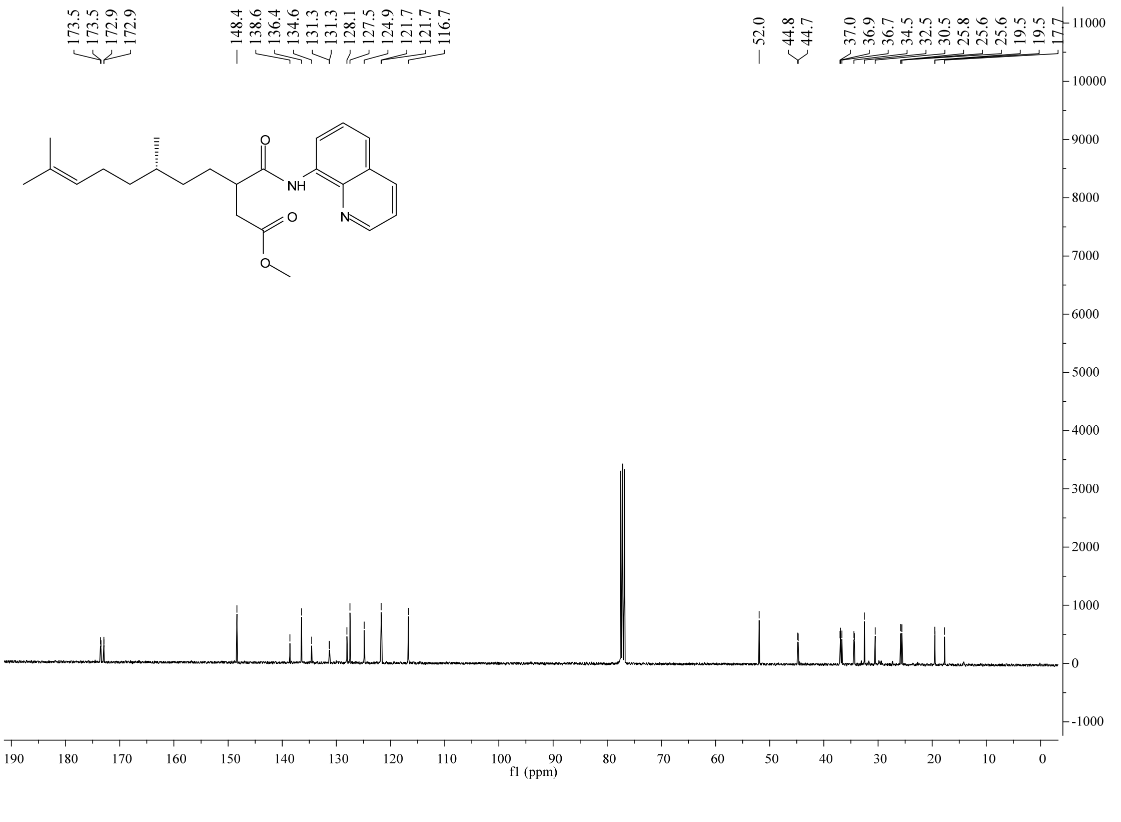

Supplementary Figure 51. ^{1}H and ^{13}C NMR spectra for 6i

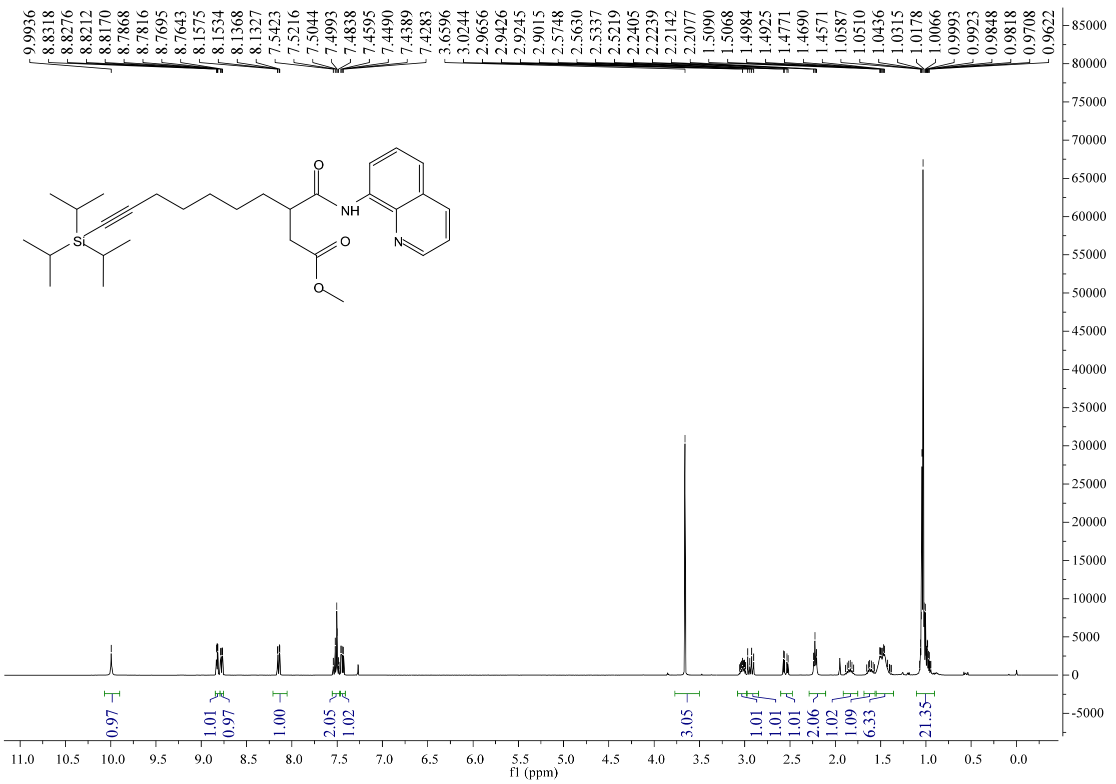

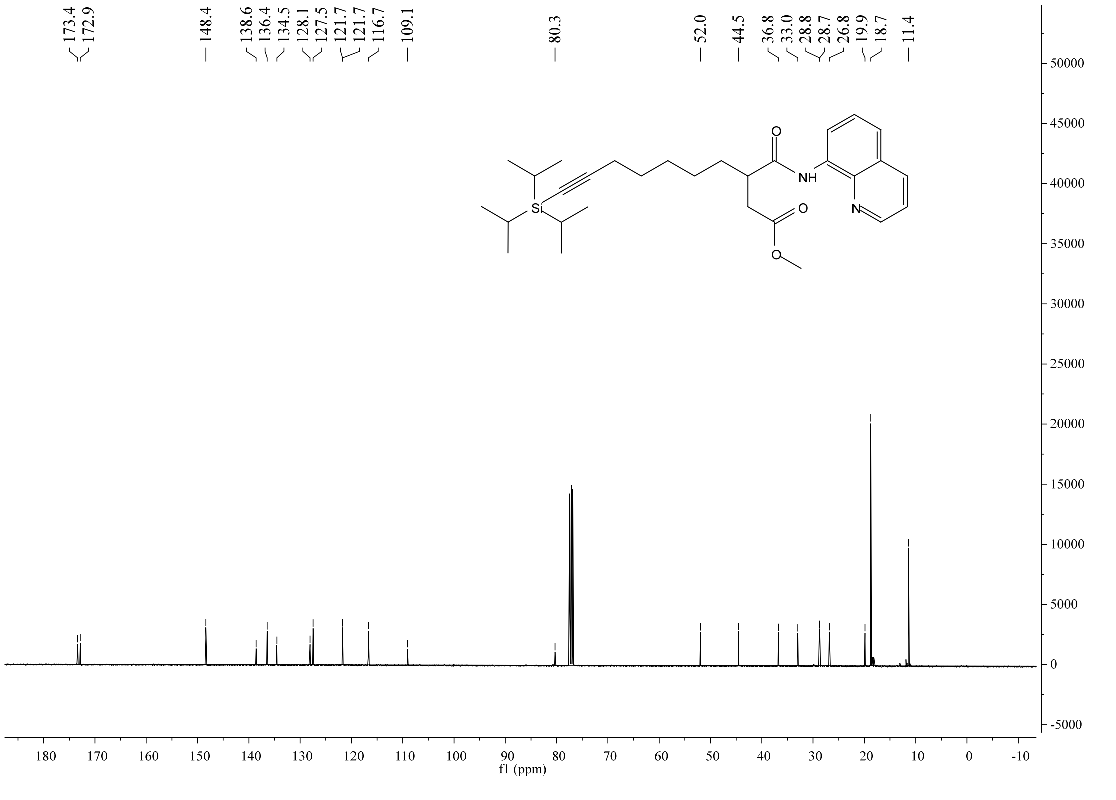

Supplementary Figure 52. ^{1}H and ^{13}C NMR spectra for 6j

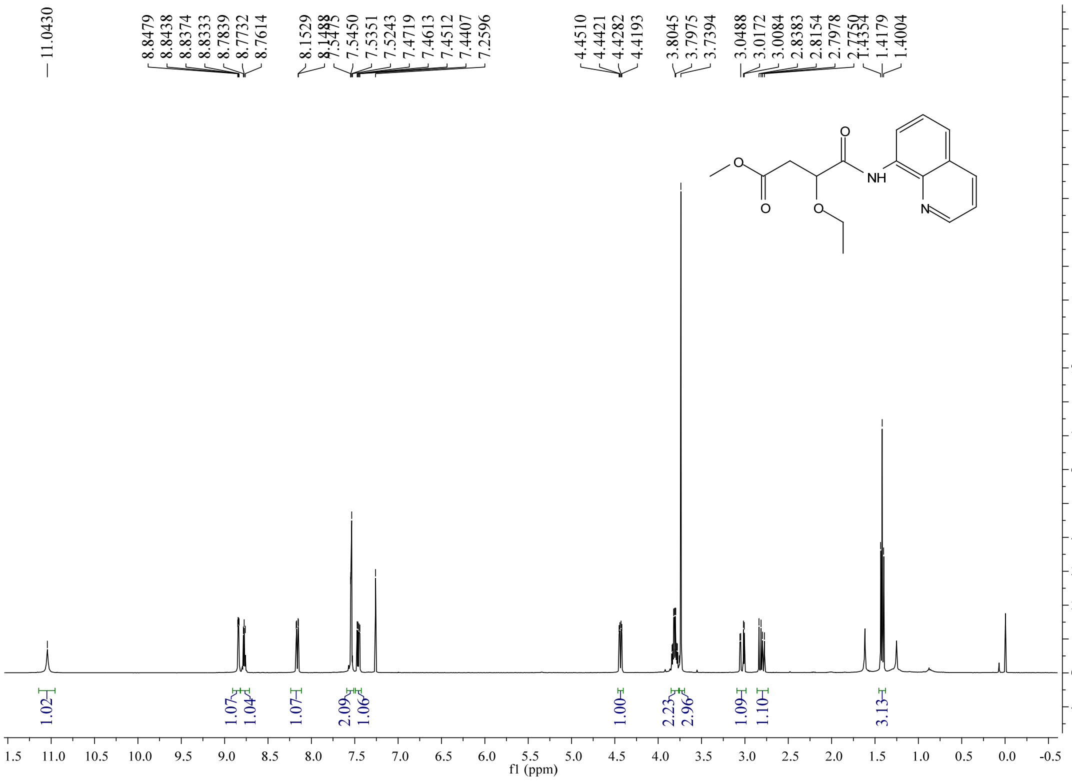

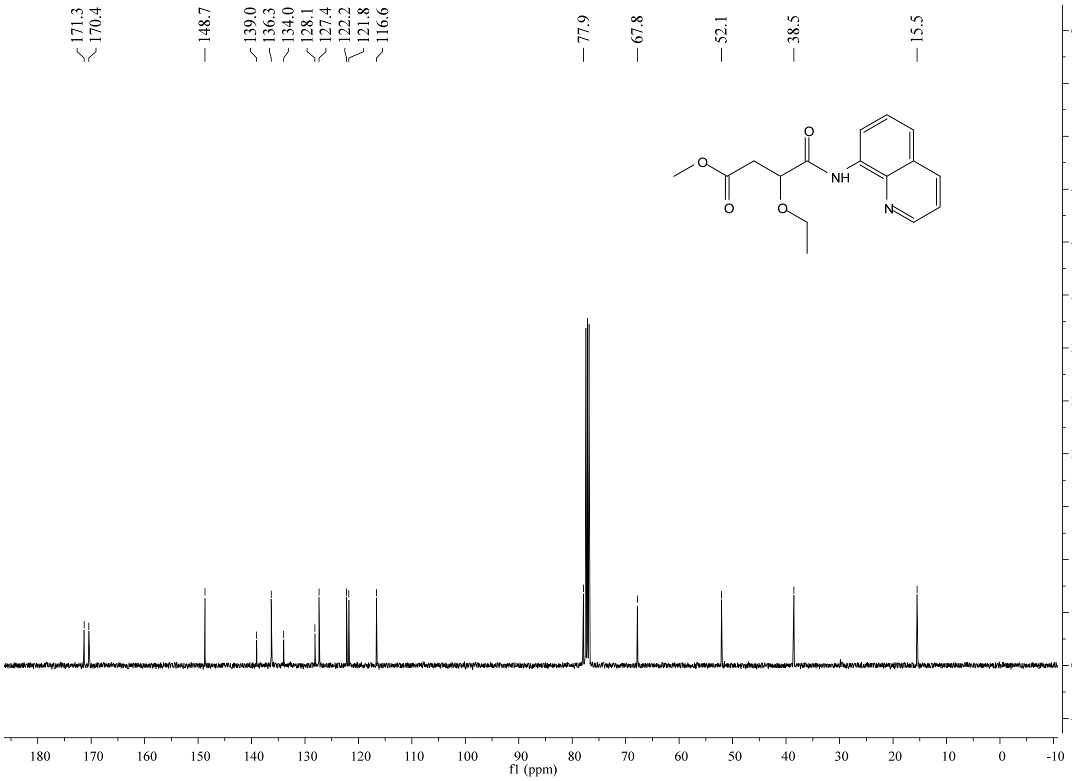

Supplementary Figure 53. ^{1}H and ^{13}C NMR spectra for 6k

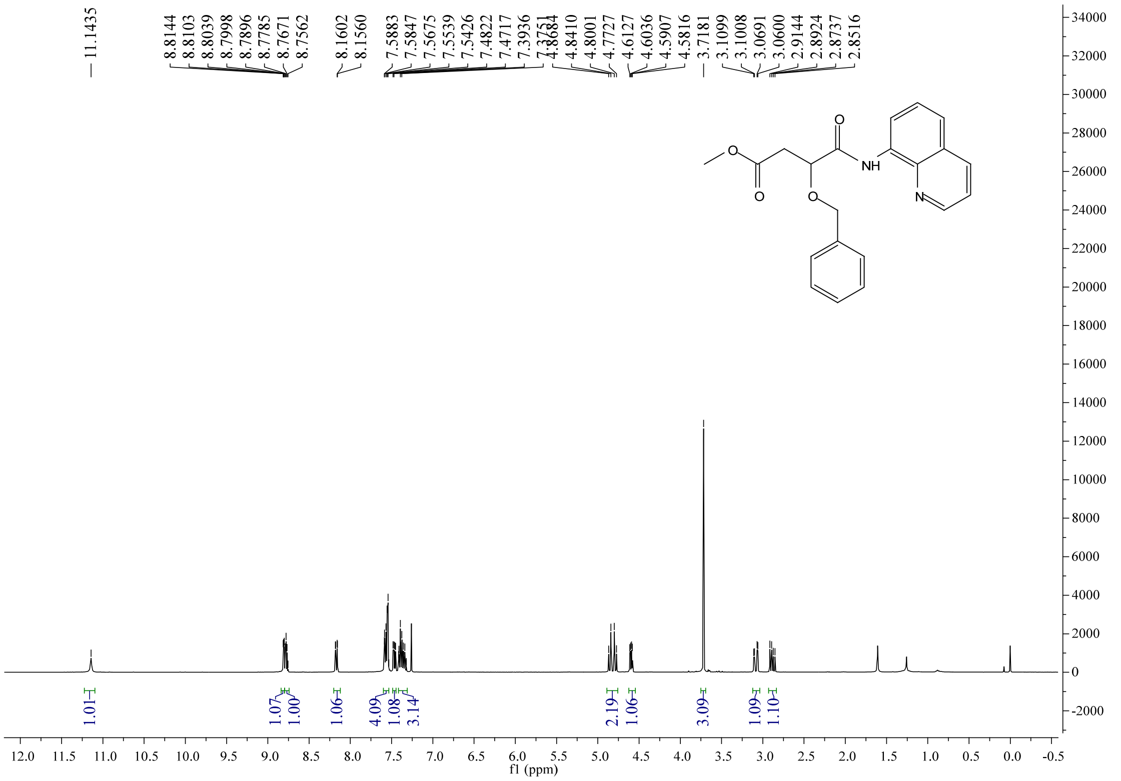

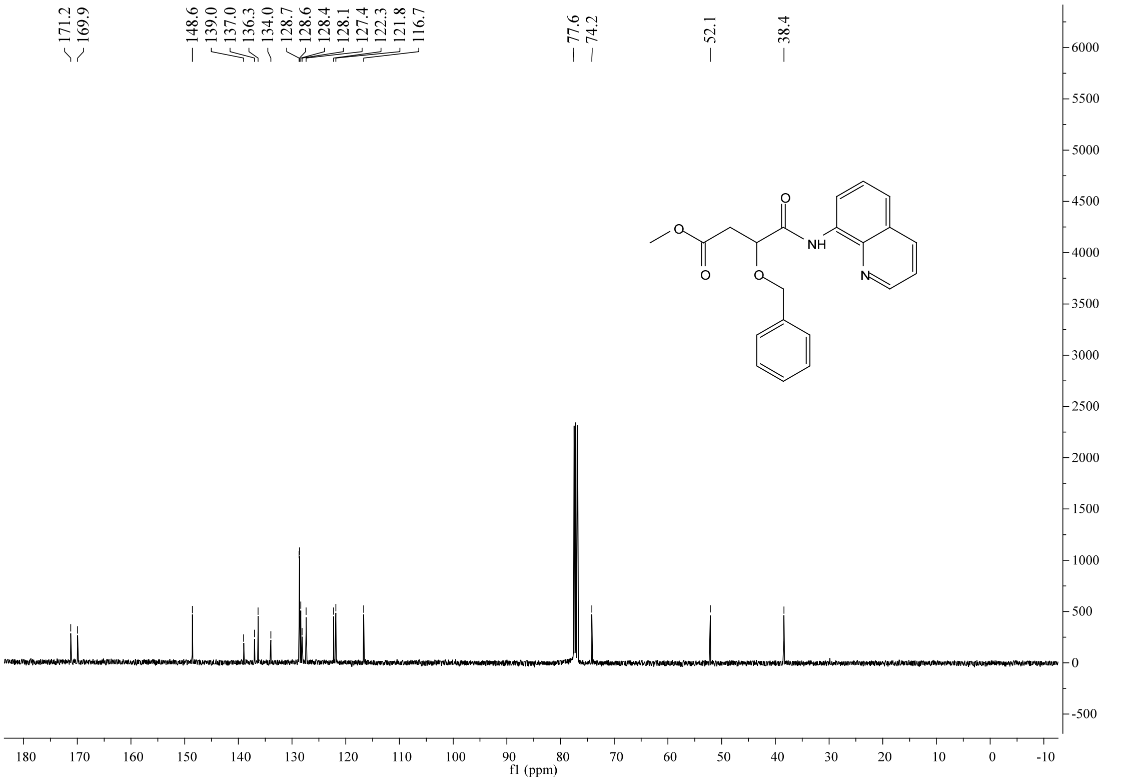

Supplementary Figure 54. ^{1}H and ^{13}C NMR spectra for 6l

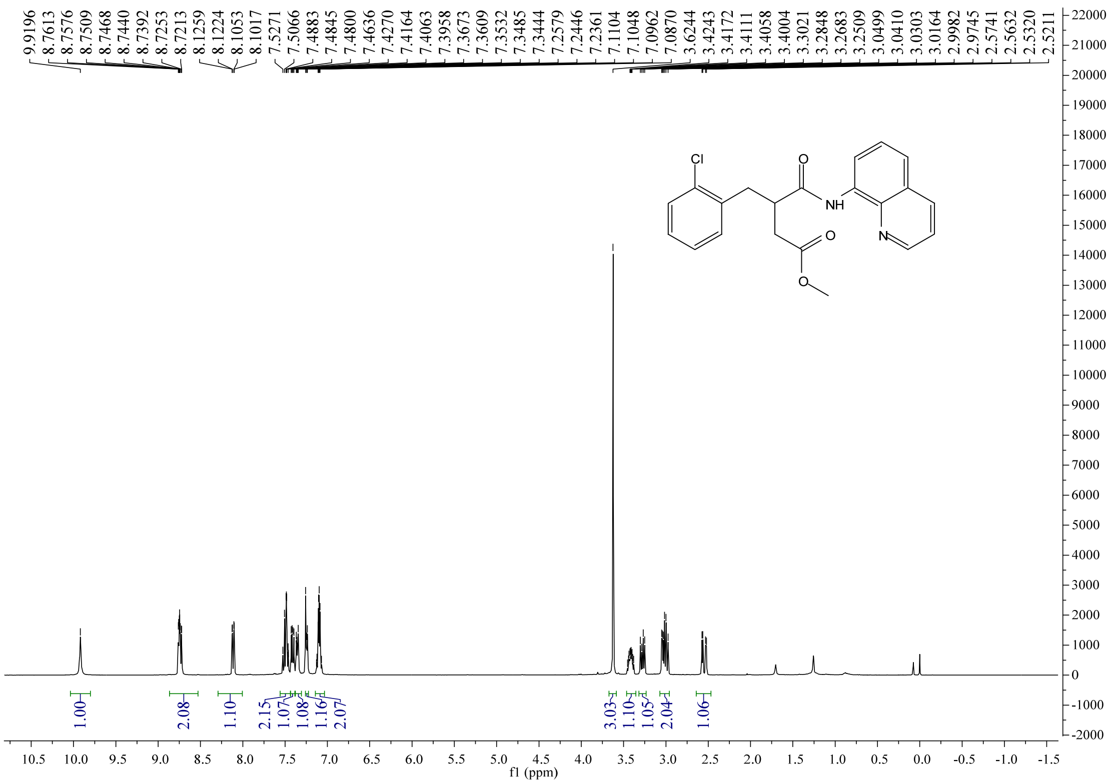

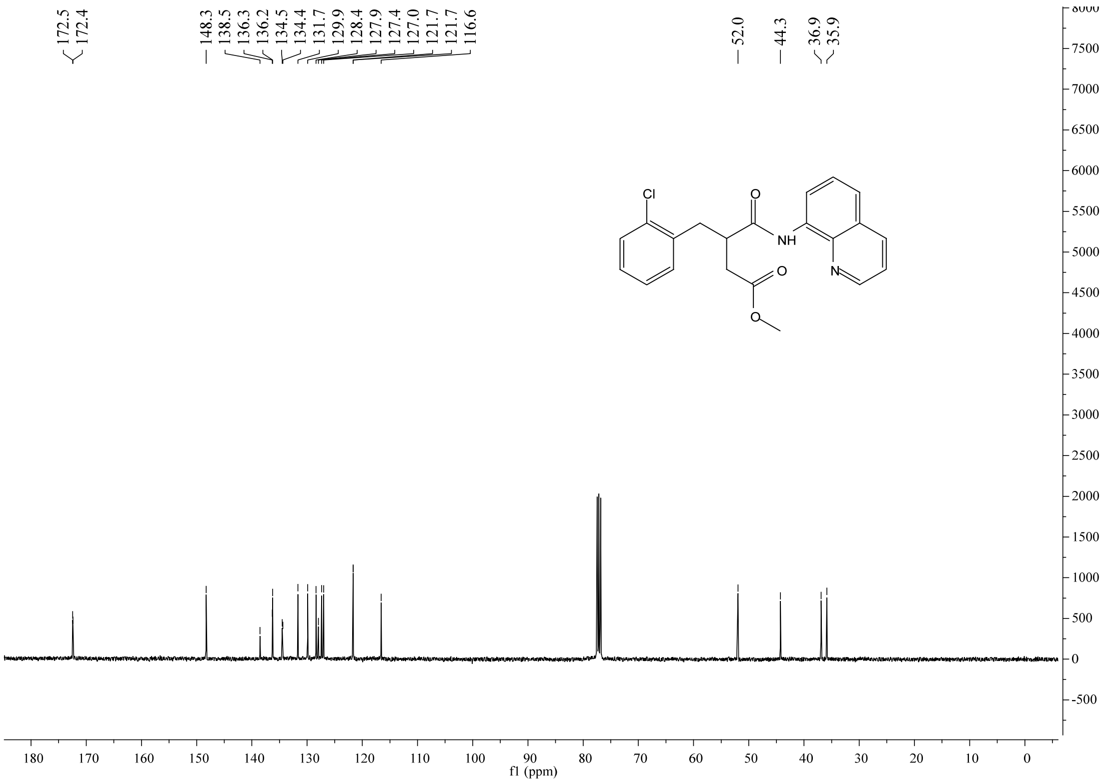

Supplementary Figure 55. ^{1}H and ^{13}C NMR spectra for 6m

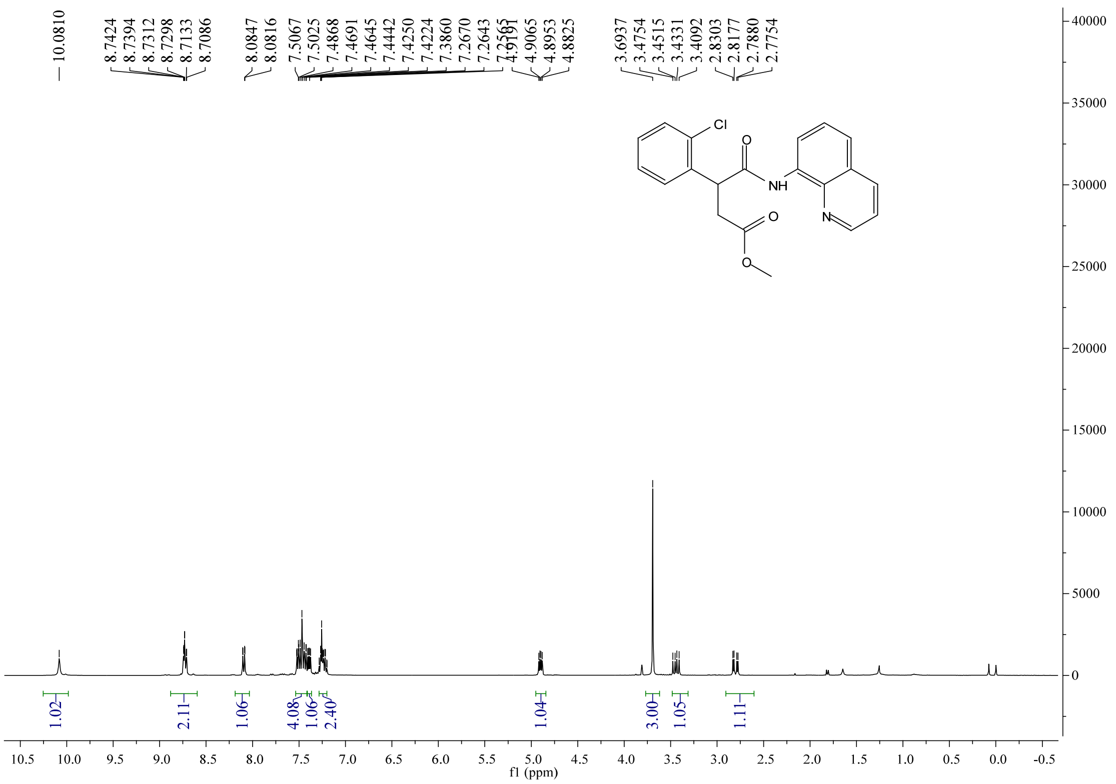

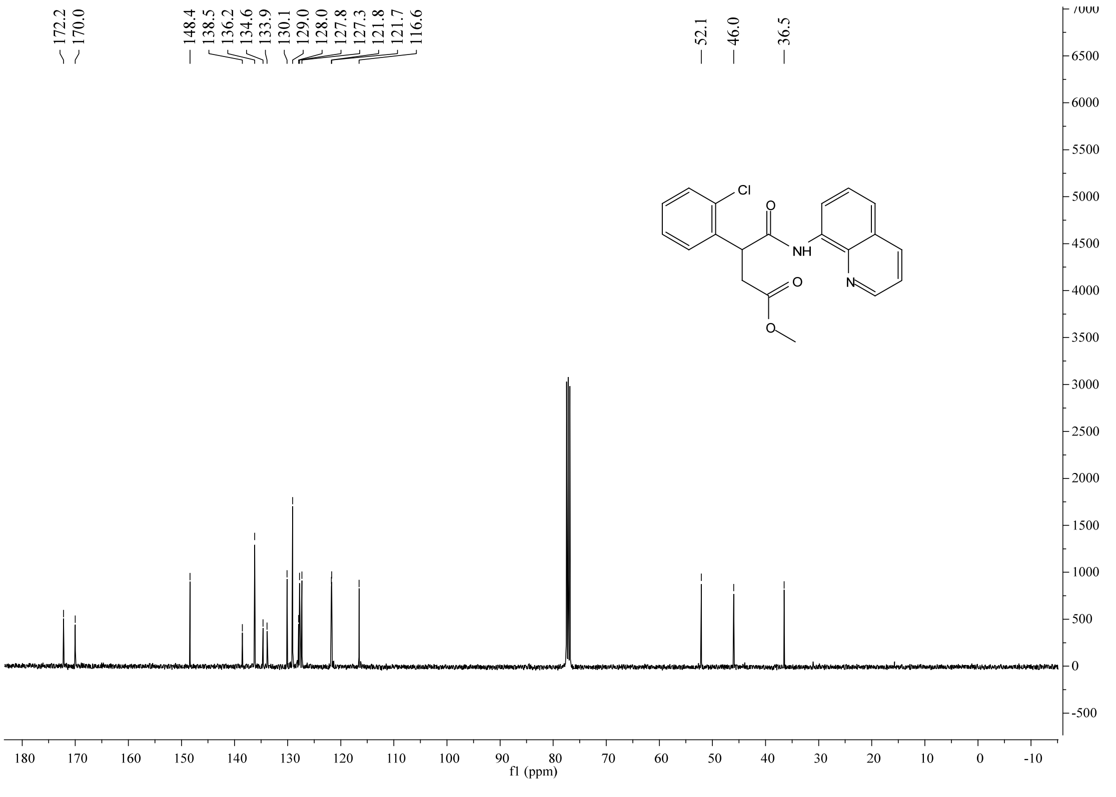

Supplementary Figure 56. ^{1}H and ^{13}C NMR spectra for 6n

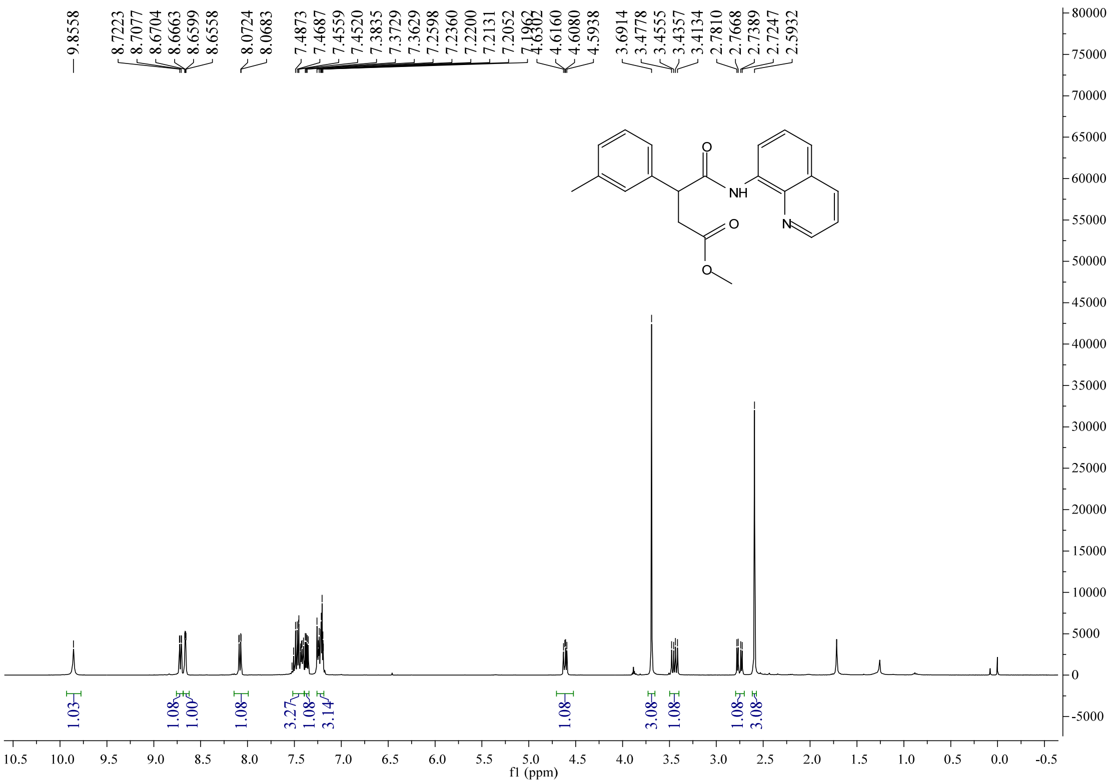

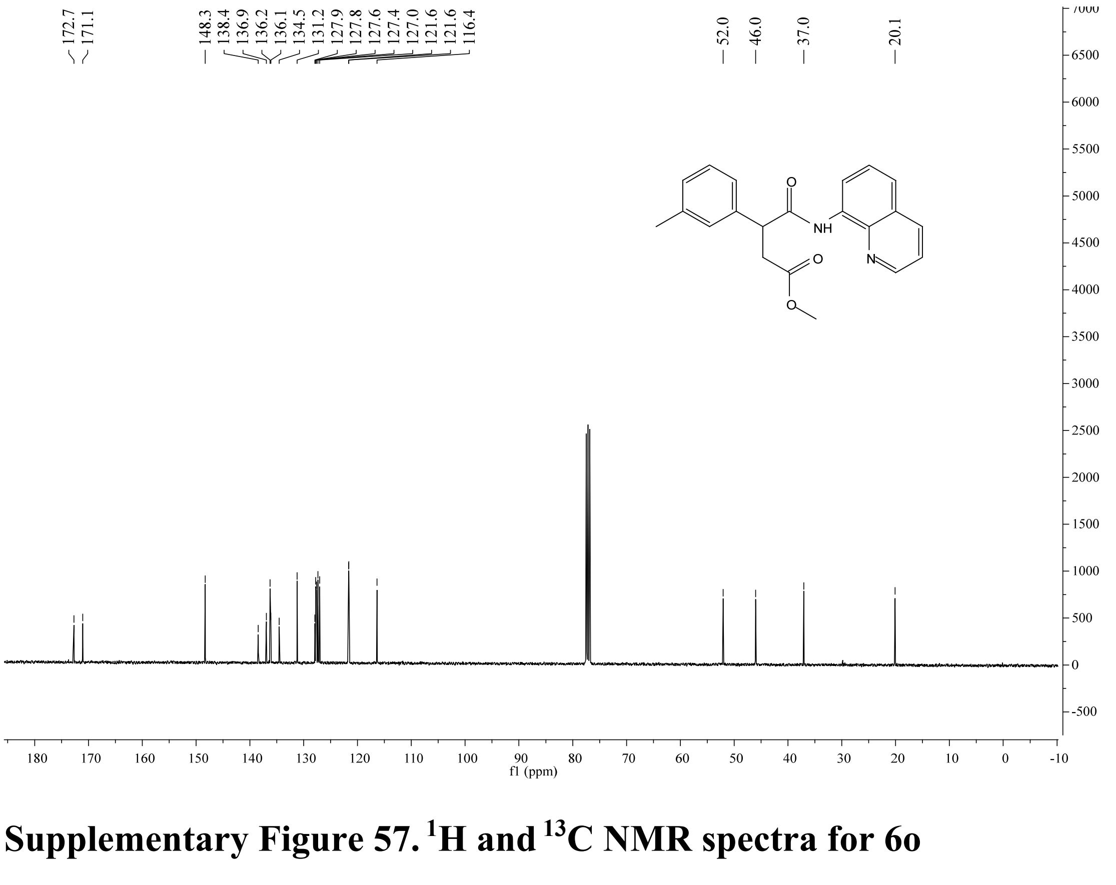

Supplementary Figure 57. ^{1}H and ^{13}C NMR spectra for 6o

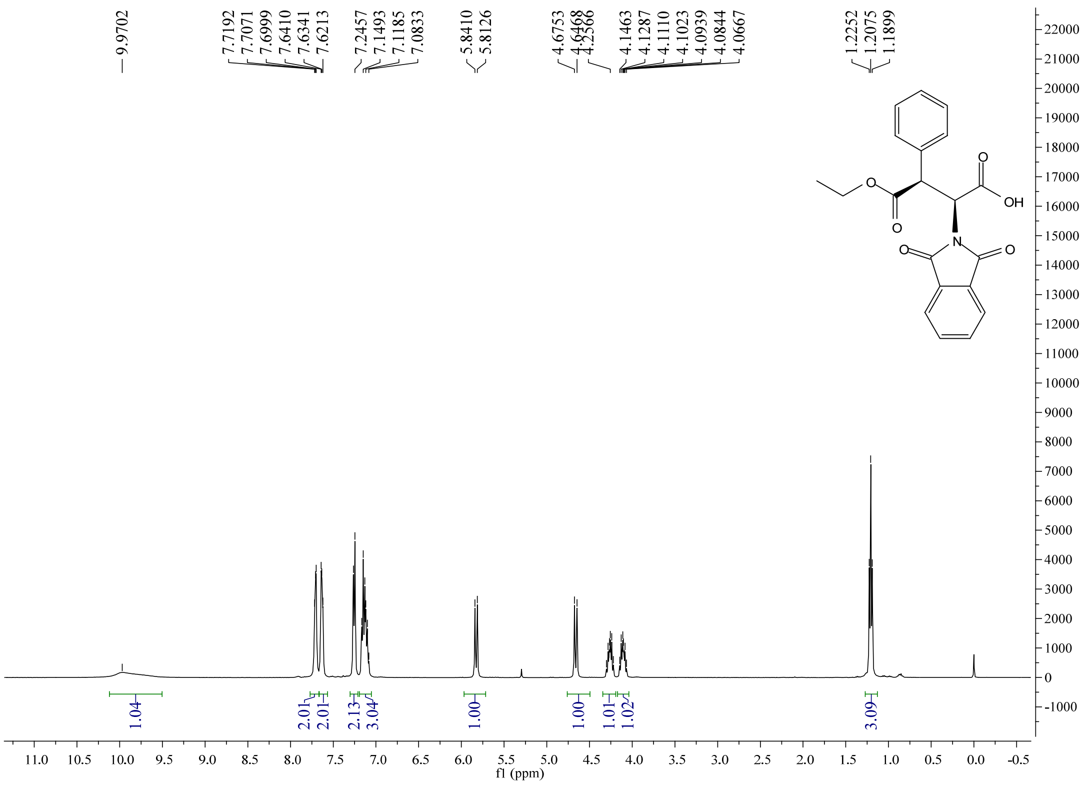

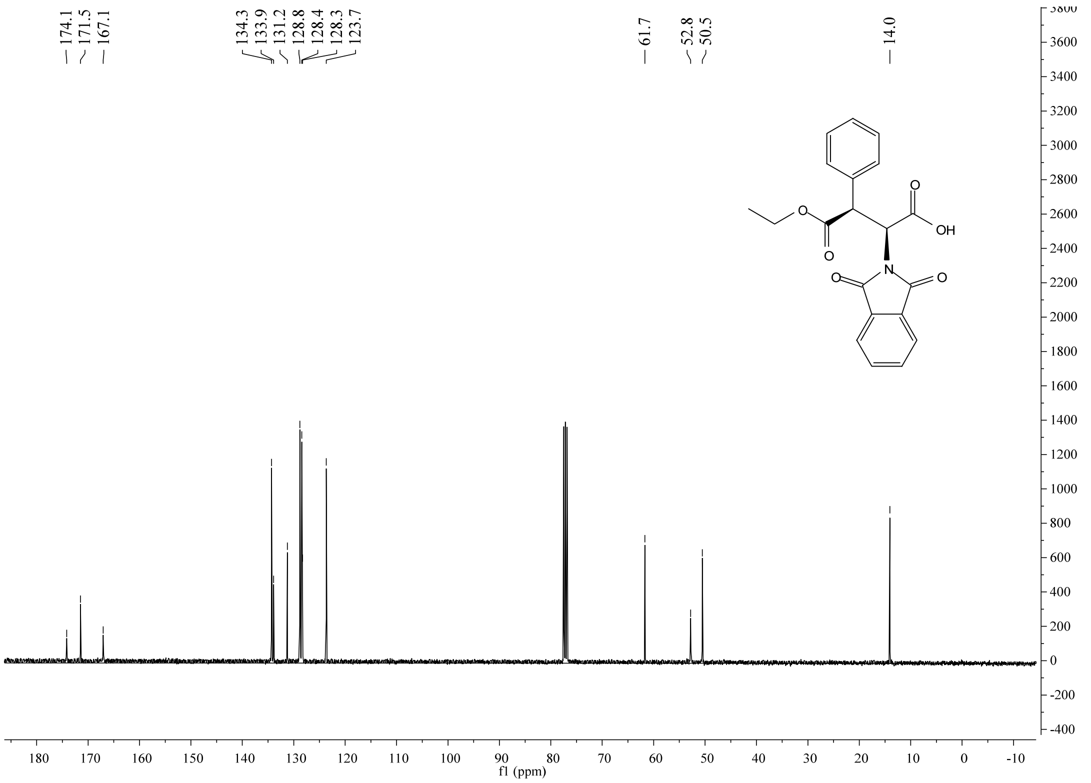

Supplementary Figure 58. ^{1}**H and** ^{13}**C NMR spectra for 7**

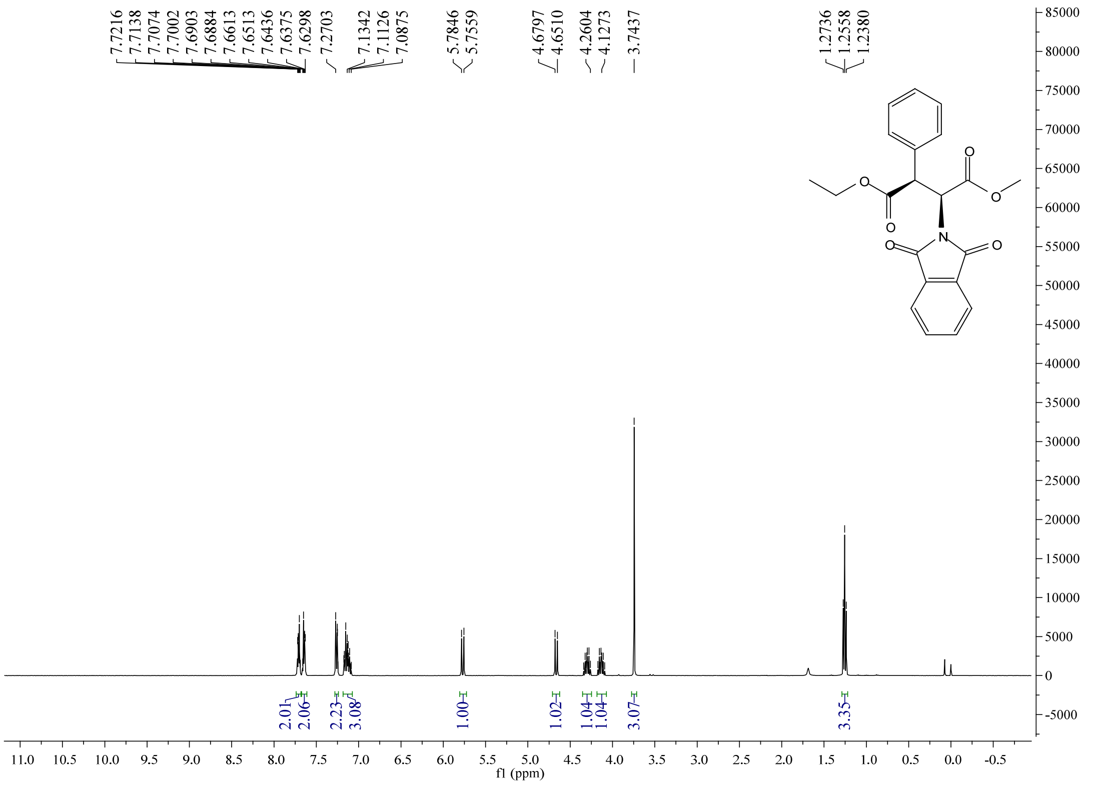

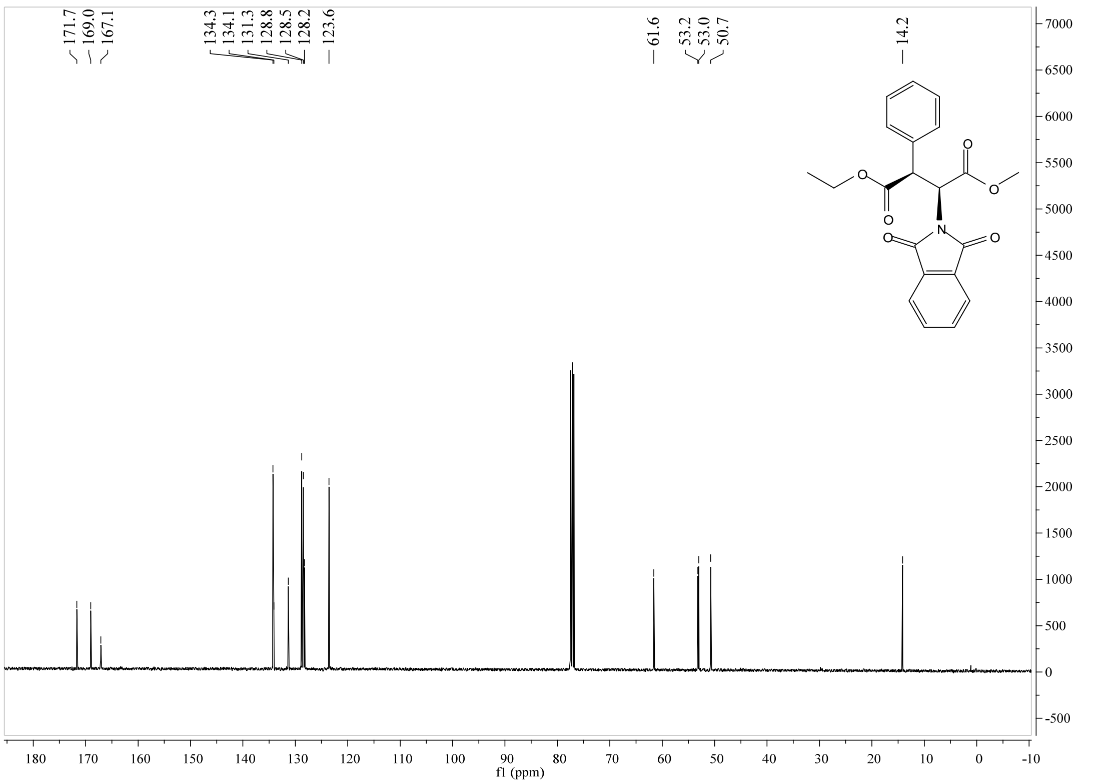

Supplementary Figure 59. ^{1}H and ^{13}C NMR spectra for 8

AD-H , n-hex : iPrOH= 70/30, 1.0 ml/min, 220 nm

分析者 : System Administrator
样品名 : LG-Phe-OEt-OMe
样品ID :
进样体积 : 4
数据文件 : DLPheOEt-OMe1.lcd
方法文件 : 13.lcm
报告格式文件 : xt.lsr
分析日期/时间 : 2016-6-19 19:13:56
处理日期/时间 : 2016-6-19 19:44:03
重复进样计数 : 1
描述 : AD-H , n-hex : iPrOH= 70/30, 1.0 ml/min, 220 nm

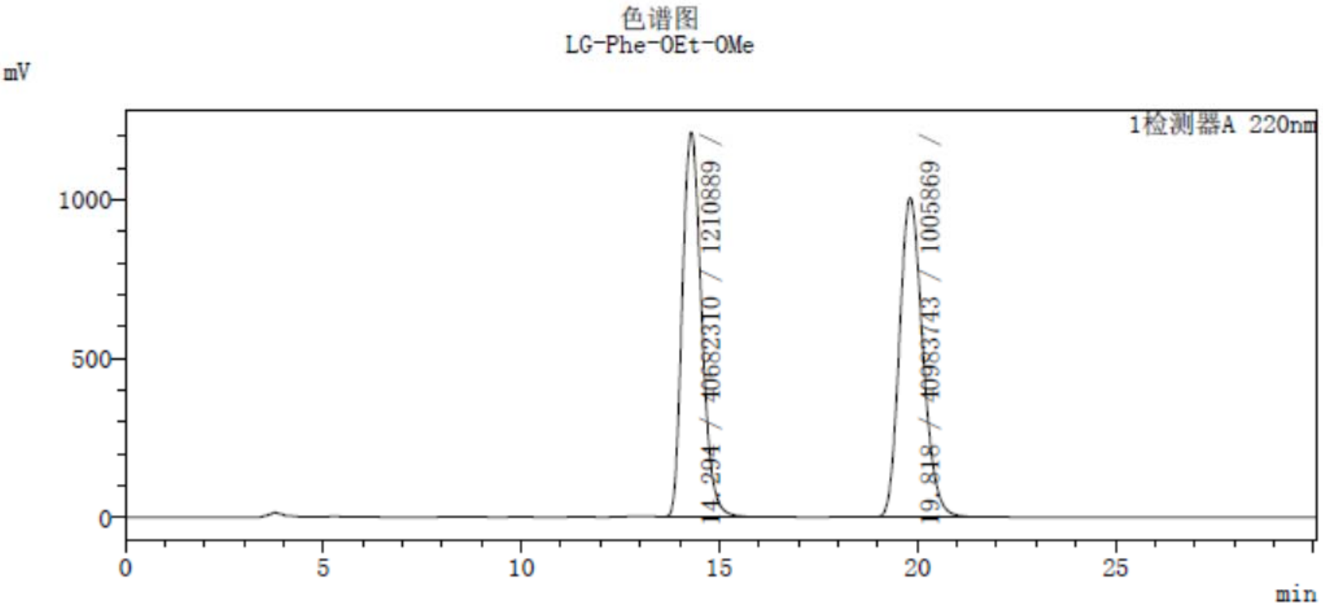

峰表

检测器A 220nm

峰号	保留时间	面积	高度	标记	面积%
1	14.294	40682310	1210889	M	49.815
2	19.818	40983743	1005869	M	50.185
总计		81666052	2216758		100.000

Supplementary Figure 60. HPLC spectrum for racemic 8

[illegible]

AD-H , n-hex : iPrOH= 70/30, 1.0 ml/min, 220 nm

分析者	: System Administrator
样品名	: LPhe-OEt-OMe
样品ID	:
进样体积	: 4
数据文件	: LPheOEt-OMe1.lcd
方法文件	: 13.lcm
报告格式文件	: xt.lsr
分析日期/时间	: 2016-6-19 19:45:11
处理日期/时间	: 2016-6-19 20:14:50
重复进样计数	: 1
描述	: AD-H , n-hex : iPrOH= 70/30, 1.0 ml/min, 220 nm

色谱图
LPhe-OEt-OMe

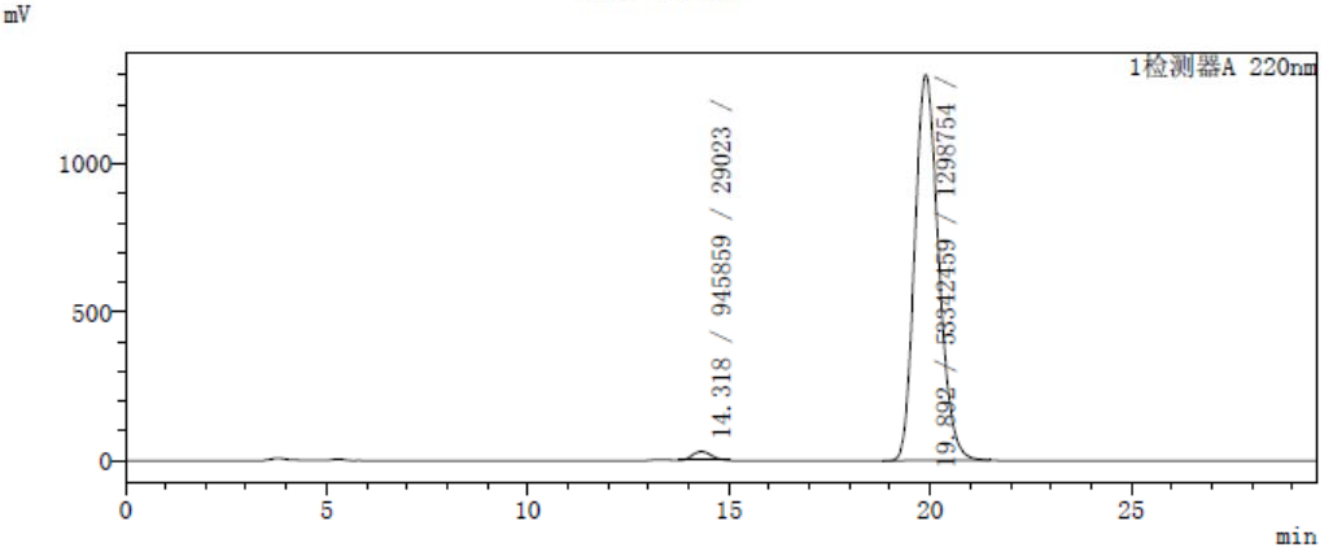

峰表

检测器A 220nm

峰号	保留时间	面积	高度	标记	面积%
1	14.318	945859	29023	M	1.742
2	19.892	53342459	1298754	M	98.258
总计		54288318	1327777		100.000

Supplementary Figure 61. HPLC spectrum for 8

X-Ray Data

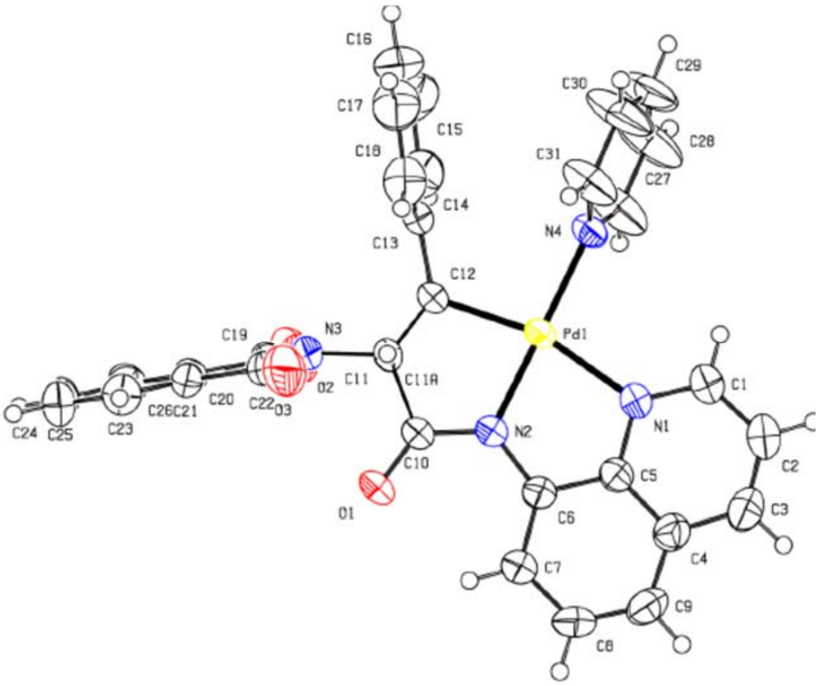

Supplementary Figure 62. X-Ray for complex III

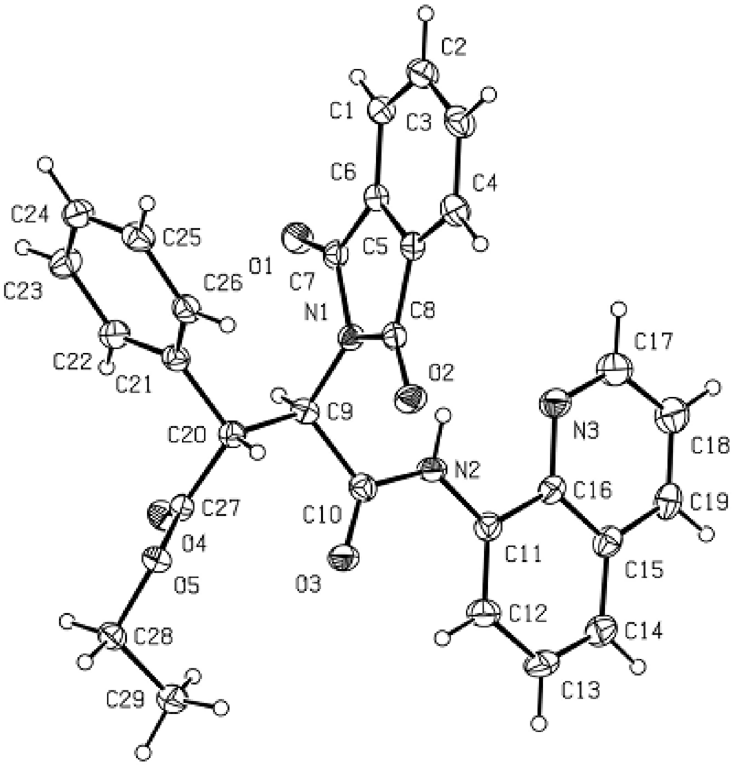

Supplementary Figure 63. Xay for complex 3a

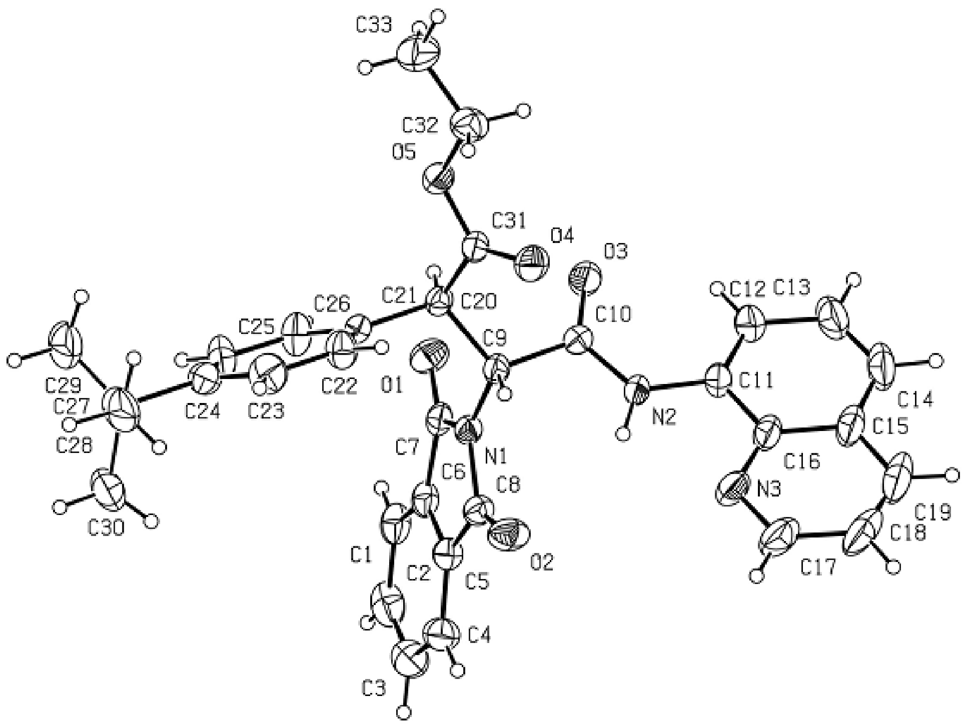

Supplementary Figure 64. Xay for complex 3d

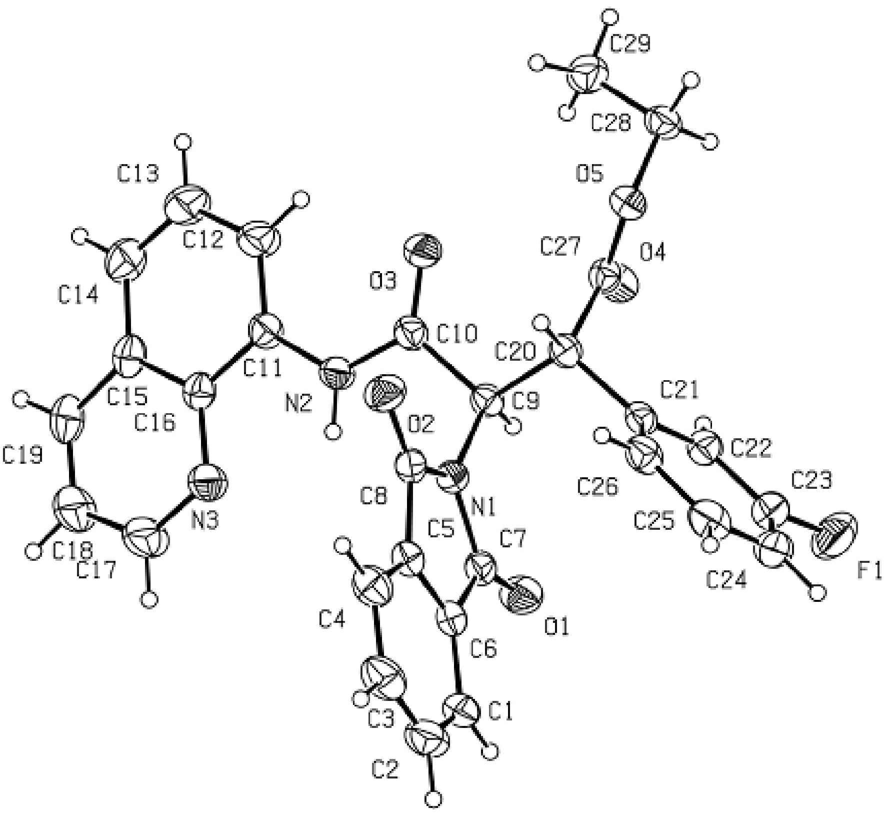

Supplementary Figure 65. Xay for complex 3k

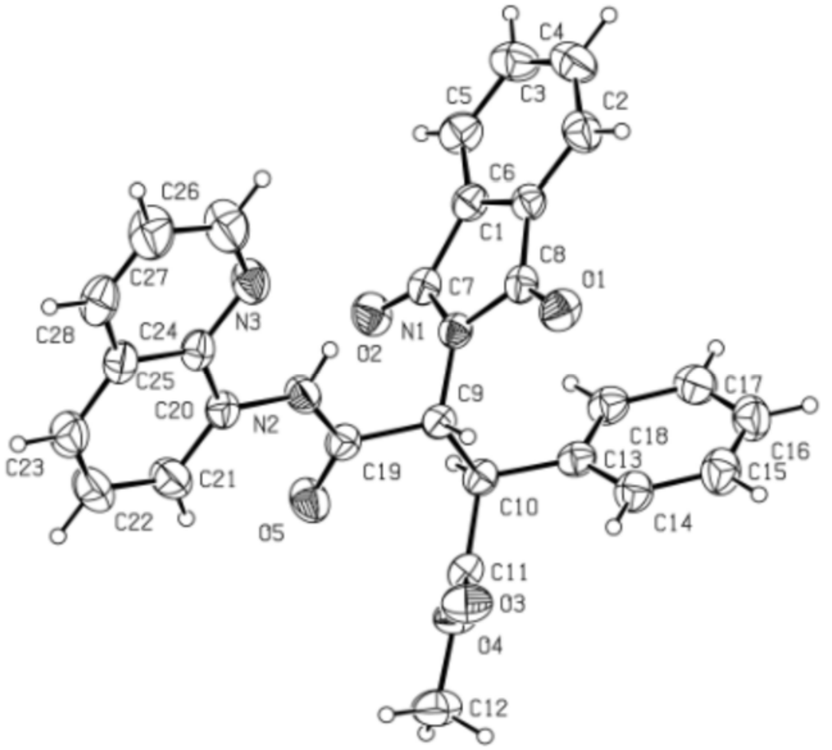

Supplementary Figure 66. Xay for complex 4b

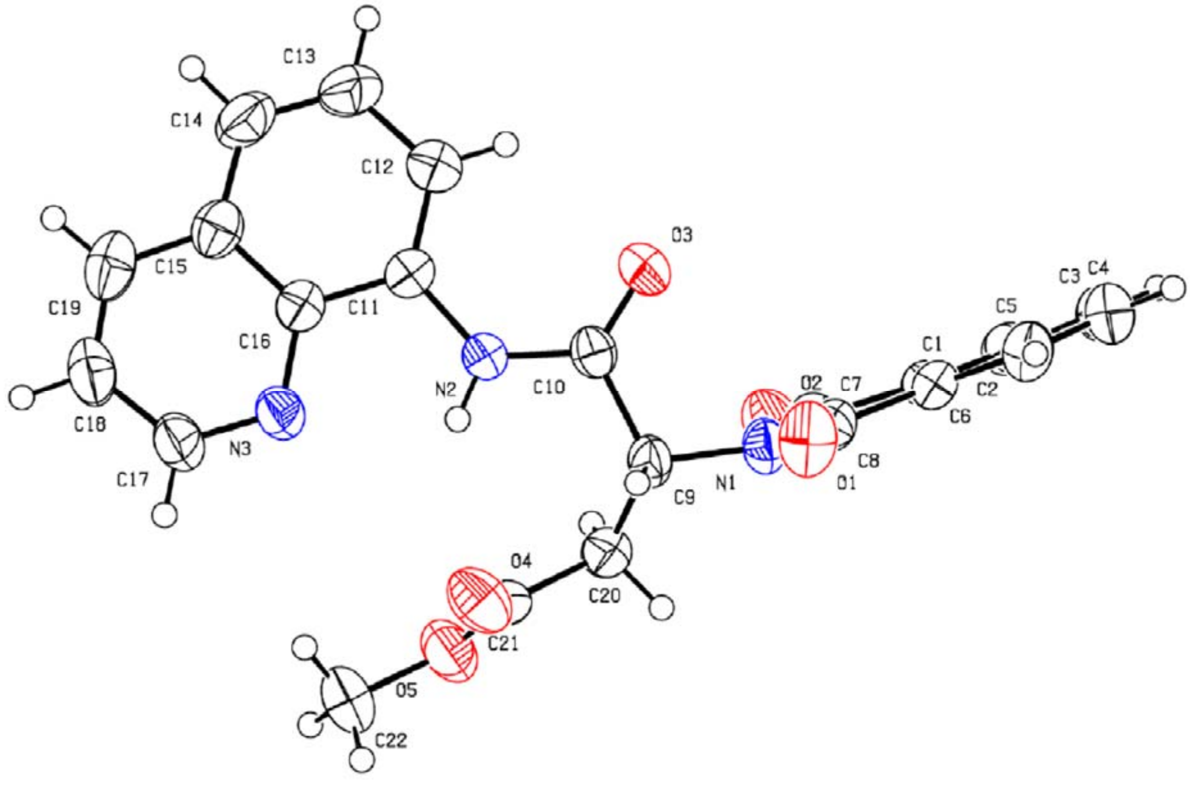

Supplementary Figure 67. Xay for complex 6a

Supplementary Table 1 Natural *α*-amino acids substrates

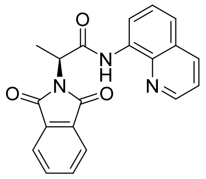

5a

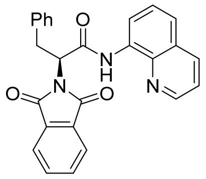

1a

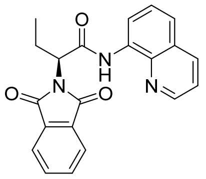

1r

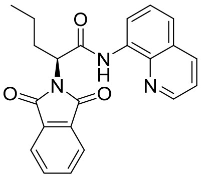

1s

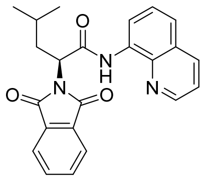

1t

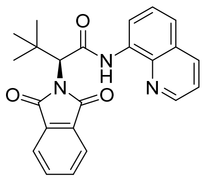

1u

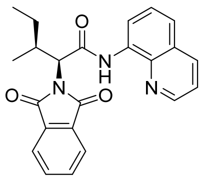

1v

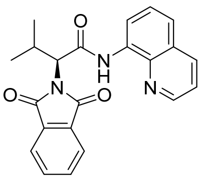

1w

Supplementary Table 2 Substituted phenylalanines substrates

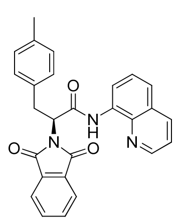

1b

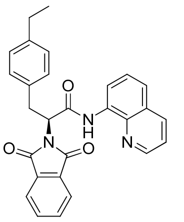

1c

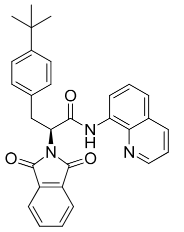

1d

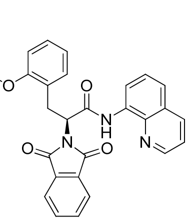

1e

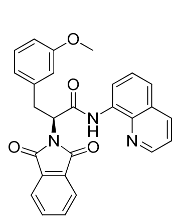

1f

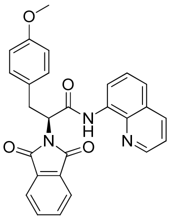

1g

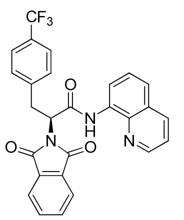

1h

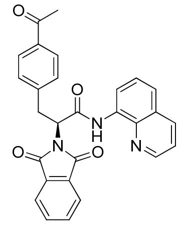

1i

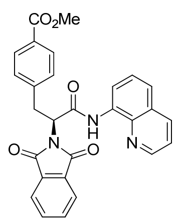

1j

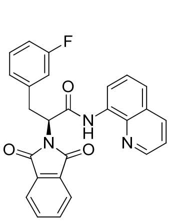

1k

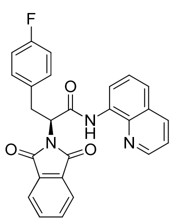

1l

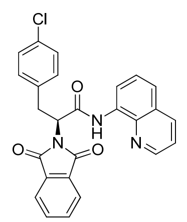

1m

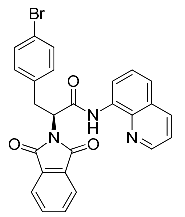

1n

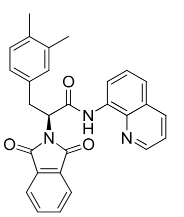

1o

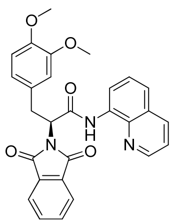

1p

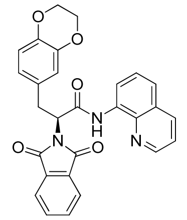

1q

Supplementary Table 3 substrates 5b-5o

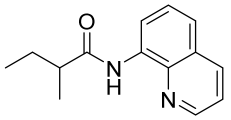

5b

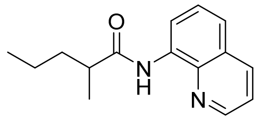

5c

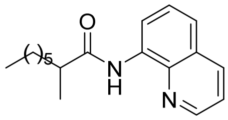

5d

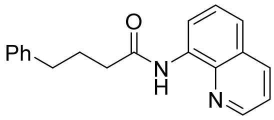

5e

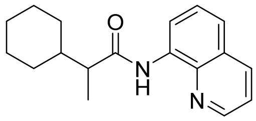

5f

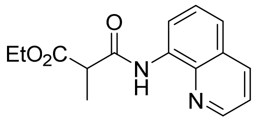

5g

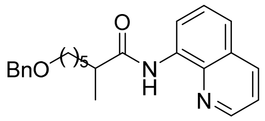

5h

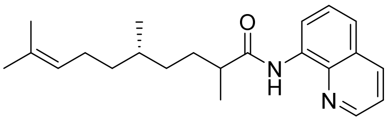

5i

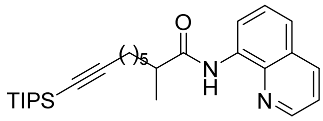

5j

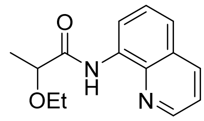

5k

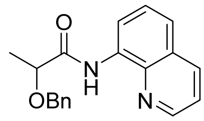

5l

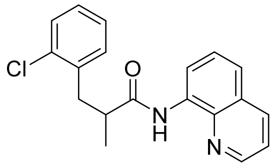

5m

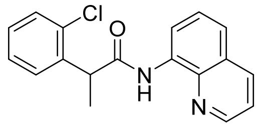

5n

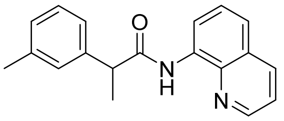

5o

Supplementary Table 4 Screening of solvent

Entry	Solvent	Yield (%)		
		**RSM**	**3a**	**3aa**
1	DCE	36	40	<5
2	CH_3CN	98	n.d.	n.d.
3	DMF	85	n.d.	n.d.
4	*t*AmOH	70	<5	n.d.
5	THF	13	62	<5
6	CH_3OH	60	n.d.	<5
7	DCM	38	34	<5
8	DMSO	80	n.d	n.d
9	1,4-dioxane	52	40	<5
**10**	**Toluene**	**14**	**65**	**<5**

Supplementary Table 5 Screening of the extra additives

Entry	Addtives (1.0 equiv.)	Yield (%)		
		**RSM**	**3a**	**3aa**
1	$NaIO_3$	n.d	72	<5
2	Oxone	n.d	78	n.d.
3	BQ	10	77	n.d.
4	*Selectfluor*	80	n.d	n.d.
5	DDQ	83	<5	n.d
6	NaI	32	35	<5
7	NIS	64	18	<5
8	CuI	11	37	n.d
**9**	**I_2**	**n.d**	**84**	**<5**

Notes: Yields were determined by ^{1}H NMR using CH_2Br_2 as the internal standard. RSM = recovered starting materials. n.d. = not detected. Toluene was used as solvent for screening of extra additive.

Supplementary Table 6 Ee Value and yield of 3a with Prolonged Heating

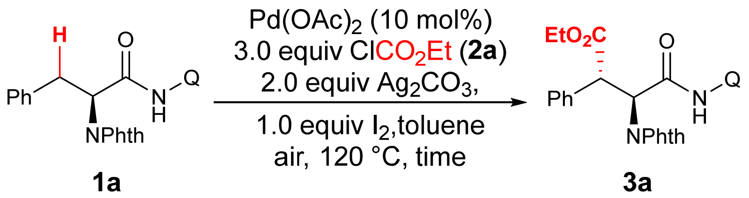

entry	time (h)	yield (%)	ee (%)
1	16	76	99
2	24	51	99
3	48	50	99

Supplementary Table 7 Crystal data and structure refinement for complex III

Bond precision:	C-C = 0.0112 A		Wavelength= 0.71073
Cell:	*a* = 9.7625 (4)	*b* = 10.5921 (3)	*c* = 14.2369 (5)
	α = 76.453 (3)	*β* = 73.120 (3)	*γ* = 67.988 (3)
Temperature:	293 K		
	Calculated		Reported
Volume	1292.94 (9)		1292.94 (8)
Space group	P -1		P -1
Hall group	-P 1		-P 1
Moiety formula	C31 H21 N4 O3 Pd		C31 H21 N4 O3 Pd
Sum formula	C31 H21 N4 O3 Pd		C31 H21 N4 O3 Pd
Mr	603.92		603.92
Dx, g cm^{-3}	1.551		1.551
Z	2		2
Mu (mm^{-1})	0.759		0.759
F*000*	610.0		610.0
F*000'*	608.23		
h, k, l*max*	11, 12, 17		11, 12, 17
N*ref*	4736		4708
T*min*, T*max*	0.833, 0.906		0.939, 1.000
T*min'*	0.827		

Correction method= # Reported T Limits: T*min* =0.939 T*max* = 1.000

AbsCorr = MULTI-SCAN

Data completeness= 0.994 Theta(*max*)= 25.350

R(*reflections*)= 0.0400 (4280) wR2(*reflections*)= 0.1143(4708)

S = 1.194 N*par*= 326

Supplementary Table 8 Crystal data and structure refinement for 3a

Bond precision:	C-C = 0.0051 A		Wavelength= 0.71073
Cell:	*a* = 7.7318 (4)	*b* = 14.2451 (8)	*c* = 21.9203 (17)
	α = 90	β = 90	γ =90
Temperature:	171 K		
	Calculated		Reported
Volume	2414.3 (3)		2414.3 (3)
Space group	P 21 21 21		P 21 21 21
Hall group	P 2ac 2ab		P 2ac 2ab
Moiety formula	C29 H23 N3 O5		C29 H23 N3 O5
Sum formula	C29 H23 N3 O5		C29 H23 N3 O5
Mr	493.50		493.50
Dx, g cm^{-3}	1.358		1.358
Z	4		4
Mu (mm^{-1})	0.094		0.094
F*000*	1032.0		1032.0
F*000’*	1032.49		
h, k, l*max*	9, 17, 26		9, 17, 26
N*ref*	4440 [2546]		2540
T*min*, T*max*	0.956, 0.979		0.891, 1.000
T*min’*	0.955		

Correction method= # Reported T Limits: T*min* =0.891 T*max* = 1.000

AbsCorr = MULTI-SCAN

Data completeness= 1.00 /0.57 Theta(*max*)= 25.350

R(*reflections*)= 0.0414(2106) wR2(*reflections*)= 0.1079(2540)

S = 1.124 N*par*= 335

Supplementary Table 9 Crystal data and structure refinement for 3d

Bond precision:	C-C = 0.0072 A		Wavelength=0.71073
Cell:	*a* =9.2583 (12)	*b* = 14.0257 (14)	*c* = 12.1921 (12)
	α = 90	*β* = 110.498 (12)	*γ* = 90
Temperature:	293 K		
	Calculated		Reported
Volume	1483.0 (3)		1483.0 (3)
Space group	P 21		P 1 21 1
Hall group	P 2yb		P 2yb
Moiety formula	C33 H31 N3 O5		C33 H31 N3 O5
Sum formula	C33 H31 N3 O5		C33 H31 N3 O5
Mr	549.61		549.61
Dx, g cm^{-3}	1.231		1.231
Z	2		2
Mu (mm^{-1})	0.084		0.084
F*000*	580.0		580.0
F*000*’	580.27		
h, k, l*max*	11, 16, 14		11, 16, 14
N*ref*	5423 [2831]		2816
T*min*, T*max*	0.960, 0.973		0.963, 1.000
T*min’*	0.960		

Correction method= # Reported T Limits: T*min*=0.963 T*max*=1.000

AbsCorr = MULTI-SCAN

Data completeness= 0.99/0.52 Theta(*max*)= 25.350

R(*reflections*)= 0.0509 (2207) wR2(r*eflections*)= 0.1250(2816)

S = 1.091 N*par*= 406

Supplementary Table 10 Crystal data and structure refinement for 3k

Bond precision:	C-C = 0.0044 A		Wavelength=0.71073
Cell:	*a* = 7.7205 (4)	*b* = 14.3320 (9)	*c* = 22.282 (1)
	α = 90	*β* = 90	*γ* = 90
Temperature:	293 K		

	Calculated	Reported
Volume	2465.5 (2)	2465.5 (2)
Space group	P 21 21 21	P 21 21 21
Hall group	P 2ac 2ab	P 2ac 2ab
Moiety formula	C29 H22 F N3 O5	C29 H22 F N3 O5
Sum formula	C29 H22 F N3 O5	C29 H22 F N3 O5
Mr	511.50	511.50
Dx, g cm^{-3}	1.378	1.378
Z	4	4
Mu (mm^{-1})	0.101	0.101
F*000*	1064.0	1064.018
F*000'*	1064.56	
h, k, l*max*	9, 17, 26	9, 17, 26
N*ref*	4524 [2592]	2585
T*min*, T*max*	0.952, 0.960	0.922, 1.000
T*min*'	0.952	

Correction method= # Reported T Limits: T*min*=0.922 T*max*=1.000

AbsCorr = MULTI-SCAN

Data completeness= 1.00/0.57 Theta(*max*)= 25.350

R(*reflections*)= 0.0369 (2103) wR2(*reflections*)= 0.0983(2585)

S = 1.057 N*par*= 344

Supplementary Table 11 Crystal data and structure refinement for 4b

Bond precision:	C-C = 0.0047 A		Wavelength=0.71073
Cell:	*a* = 7.6735 (5)	*b* = 14.4087 (11)	*c* = 21.653 (2)
	α = 90	β = 90	γ = 90
Temperature:	293 K		
	Calculated		Reported
Volume	2394.1 (3)		2394.0 (4)
Space group	P 21 21 21		P 21 21 21
Hall group	P 2ac 2ab		P 2ac 2ab
Moiety formula	C28 H21 N3 O5		C28 H21 N3 O5
Sum formula	C28 H21 N3 O5		C28 H21 N3 O5
Mr	479.48		479.48
Dx, g cm^{-3}	1.330		1.330
Z	4		4
Mu (mm^{-1})	0.093		0.093
F*000*	1000.0		1000.0
F*000'*	1000.48		
h, k, l*max*	9, 17, 26		9, 17, 26
N*ref*	4384 [2515]		2509
T*min*, T*max*	0.968, 0.976		0.949, 1.000
T*min'*	0.962		

Correction method= # Reported T Limits: Tmin=0.949 Tmax=1.000

AbsCorr = MULTI-SCAN

Data completeness= 1.00/0.57 Theta(*max*)= 25.350

R(*reflections*)= 0.0400 (1998) wR2(*reflections*)= 0.1024(2509)

S = 1.037 N*par*= 326

Supplementary Table 12 Crystal data and structure refinement for 6a

Bond precision:	C-C = 0.0036 A		Wavelength=0.71073
Cell:	*a* = 10.4773(6)	*b* = 11.3275(6)	*c* = 15.9059(8)
	α = 90	β = 90	γ = 90
Temperature:	293 K		
	Calculated		Reported
Volume	1887.74(18)		1887.74(17)
Space group	P 21 21 21		P 21 21 21
Hall group	P 2ac 2ab		P 2ac 2ab
Moiety formula	C22 H17 N3 O5		C22 H17 N3 O5
Sum formula	C22 H17 N3 O5		C22 H17 N3 O5
Mr	403.39		403.39
Dx,g cm^{-3}	1.419		1.419
Z	4		4
Mu (mm^{-1})	0.103		0.103
F*000*	840.0		840.0
F*000'*			840.43
h,k,l*max*	12, 13, 19		12, 13, 19
N*ref*	3463 [1985]		1980
Tmin,T*max*	0.952, 0.964		0.983, 1.000
Tmin'	0.952		

Correction method= # Reported T Limits: T*min*=0.983 T*max*=1.000

AbsCorr = MULTI-SCAN

Data completeness= 1.00/0.57 Theta(*max*)= 25.350

R(reflections)= 0.0337(1668) wR2(*reflections*)= 0.0847(1980)

S = 1.050 Npar= 272

Supplementary Methods

Instrumentation and Chemical

Unless otherwise noted, all commercial materials were used without further purification. Anhydrous solvents obtained from Aladdin and Adamas were used directly without further purification, and solvents obtained from other commercial suppliers were used after purification as specified in *Purification of Laboratory Chemicals, 6th Ed*. Nuclear magnetic resonance (NMR) spectra were recorded with a Bruker AVANCE 400MHz instrument. ^{1}H and ^{13}C chemical shifts are reported in ppm downfield of tetramethylsilane and referenced to residual solvent peak ($CHCl_3$ = 7.26 (^{1}H NMR), DMSO = 2.50 (^{1}H NMR), $CDCl_3$ = 77.16 (^{13}C NMR)) unless otherwise noted. ^{19}F NMR chemical shifts were determined relative to internal standard ($CFCl_3$ = 0.0 (^{19}F NMR)). Multiplicities are reported using the following abbreviations: s = singlet, d = doublet, t = triplet, q = quartet, m = multiplet, br = broad resonance. HPLC analyses were performed on a Shimadzu SPD-20A. High-resolution mass spectra for new compounds were recorded at Mass Spectrometry Facilities, Zhejiang University. X-ray diffraction experiments were performed at X-Ray Facilities, Zhejiang University.

1. Proof of Concept on Alkoxycarbonylation of Methylene C(sp^3)-H Bonds via Pd(II)/Pd(IV) Catalysis:

1.1. Synthesis and Characterization of Palladacycles

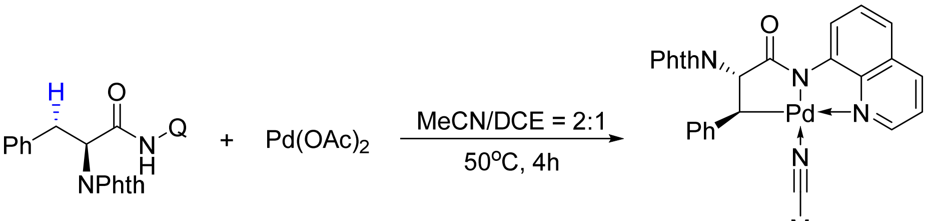

1a

I, 95%, d.r. > 25 : 1

The intermediate **I** was prepared according to literture[4]. ^{1}H NMR (400 MHz, DMSO-d_6) δ 9.34 (dd, J = 4.8, 1.1 Hz, 1H), 8.93 (p, J = 4.2 Hz, 1H), 8.61 (dd, J = 8.3, 1.5 Hz, 1H), 7.83 (s, 4H), 7.73 (dd, J = 8.3, 5.0 Hz, 1H), 7.61 – 7.57 (m, 2H), 7.52 – 7.46 (m, 2H), 7.23 – 7.14 (m, 3H), 5.35 (d, J = 10.1 Hz, 1H), 3.94 (d, J = 10.1 Hz, 1H), 2.07 (s, 3H).

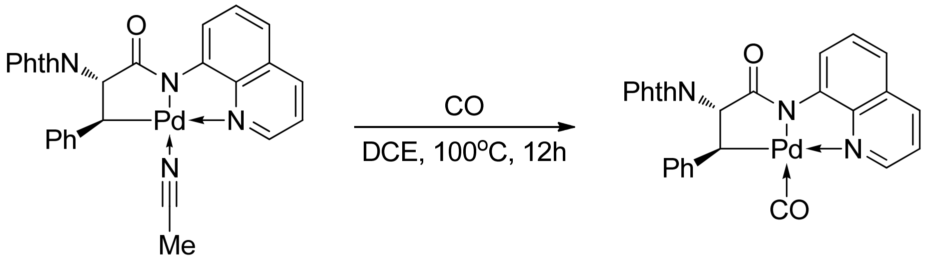

I, 0.1 mmol

II, 80%

To a 50 mL Schlenk tube, **I** (56.7 mg, 0.1 mmol) were added to DCE (2 mL). Then the tube was charged with CO. The reaction was stirred at 100 °C for 12 h, and then the solvent was removed in vacuum. To the crude product, CH_2Cl_2 (1 mL) was added, followed by petroleum ether (5 mL), and then the purified product was collected by vacuum filtration, washed with petroleum ether, and dried under vacuum to afford **II** (44.5 mg, 80%) as a pale yellow powder.

The reaction of palladacycle **I** with CO under various conditions with numerous nucleophiles didn't give any desired products. The treatment of complex **II** under various conditions also failed to give any desired products via Pd(II)/Pd(0).

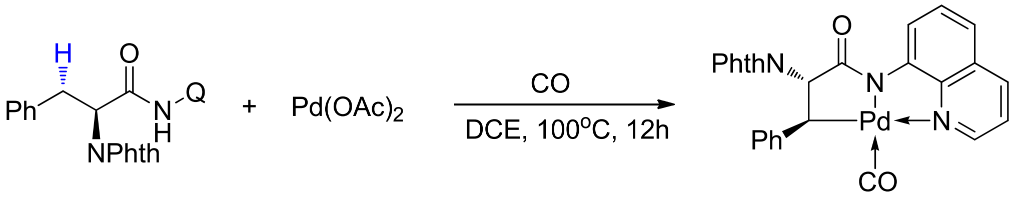

1a **II**, 80%

To a 50 mL Schlenk tube, $Pd(OAc)_2$ (44.9 mg, 0.2 mmol) and **1a** (84.3 mg, 0.2 mmol) were added to DCE (2 mL). Then the tube was charged with CO. The reaction was stirred at 100 °C for 12 h, and then the solvent was removed in vacuum. To the crude product, CH_2Cl_2 (2 mL) was added, followed by petroleum ether (10 mL), and then the purified product was collected by vacuum filtration, washed with petroleum ether, and dried under vacuum to afford **II** (88.5 mg, 80%) as a pale yellow powder. ^1H NMR (400 MHz, $CDCl_3$) δ 8.98 (dd, J = 7.8, 0.9 Hz, 1H), 8.53 (dd, J = 4.8, 1.5 Hz, 1H), 8.35 (dd, J = 8.4, 1.4 Hz, 1H), 7.77 (s, 2H), 7.68 – 7.62 (m, 2H), 7.61 – 7.46 (m, 4H), 7.46 – 7.39 (m, 1H), 7.23 (t, J = 7.6 Hz, 2H), 7.12 (t, J = 7.4 Hz, 1H). ^{13}C NMR (101 MHz, $CDCl_3$) δ 179.0, 175.3, 149.7, 147.8, 146.5, 144.0, 139.6, 133.9, 132.2, 129.9, 129.8, 129.0, 127.3, 126.0, 123.4, 121.7, 121.6, 120.3, 61.1, 40.0. HRMS (ESI): calc. for $C_{27}H_{18}N_3O_4Pd$ ($M+H^+$): 554.0327; Found: 554.0334. ν(CO) = 2095 (s) cm-1

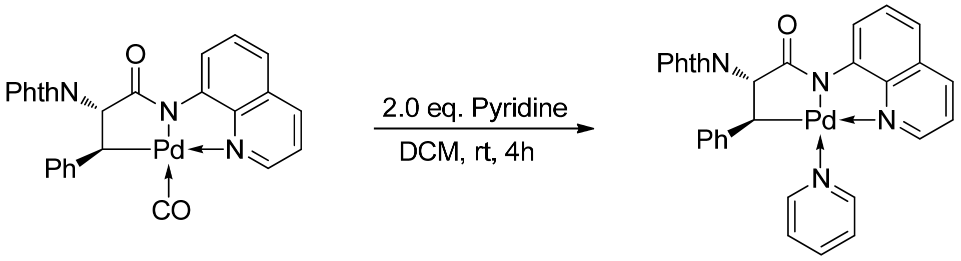

II **III**, 100%

Complex **II** (55.3 mg, 0.1 mmol) and pydrine (15.8, 0.1 mmol) were added to DCE (20 mL). The reaction was stirred at 80 °C for 15 h, and then the solvent was removed in vacuum. To the crude product, CH_2Cl_2 (4 mL) was added, followed by petroleum ether (20 mL), and then the purified product was collected by vacuum filtration, washed with petroleum ether, and dried under vacuum to afford Ⅲ (60.4 mg, 100%) as a orange powder.

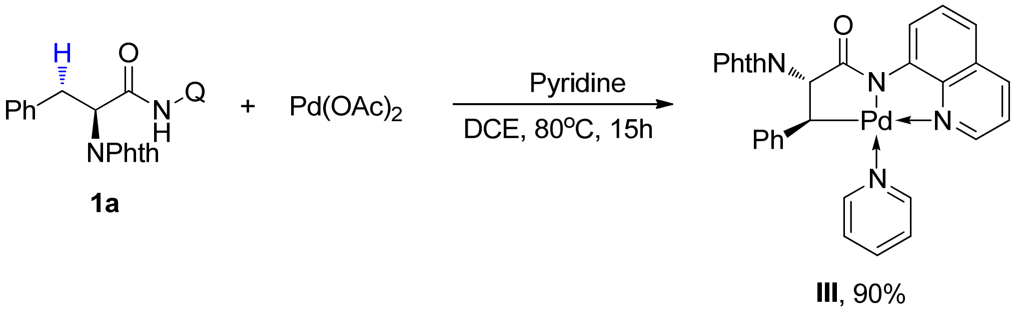

Pd(OAc)2 (224.5 mg, 1.0 mmol) , **1a** (426.0 mg, 1.0 mmol) and pydrine(158.2 mg, 2.0 mmol) were added to DCE (20mL). The reaction was stirred at 80 °C for 15 h, and then the solvent was removed in vacuum. To the crude product, CH_2Cl_2 (4 mL) was added, followed by petroleum ether (20 mL), and then the purified product was collected by vacuum filtration, washed with petroleum ether, and dried under vacuum to afford **III** (543.6 mg, 90%) as a orange powder. ^{1}H NMR (400 MHz, CDCl3) δ 9.12 (d, *J* = 7.8 Hz, 1H), 8.36 (s, 2H), 8.20 (d, *J* = 8.2 Hz, 1H), 7.75 (s, 2H), 7.65 (t, *J* = 7.8 Hz, 1H), 7.62 – 7.57 (m, 3H), 7.53 (t, *J* = 7.9 Hz, 1H), 7.32 (d, *J* = 7.9 Hz, 1H), 7.25 – 7.22 (m, 1H), 7.17 – 7.07 (m, 4H), 6.88 – 6.75 (m, 3H), 5.93 (d, *J* = 10.8 Hz, 1H), 4.46 (d, *J* = 10.8 Hz, 1H). ^{13}C NMR (101 MHz, CDCl3) δ 176.5, 151.8, 147.8, 146. 2, 145.9, 144.8, 138.4, 137.0, 133.5, 129.9, 129.6, 128.2, 126.9, 125.1, 123.2, 121.6, 120.8, 119.2, 61.1, 32.9. HRMS (ESI): calc. for $C_{31}H_{23}N_4O_3Pd$ (M+H$^+$): 605.0800; Found: 605.0804.

1.2. Stoichiometric Reactivity of Complex I with $ClCO_2R$

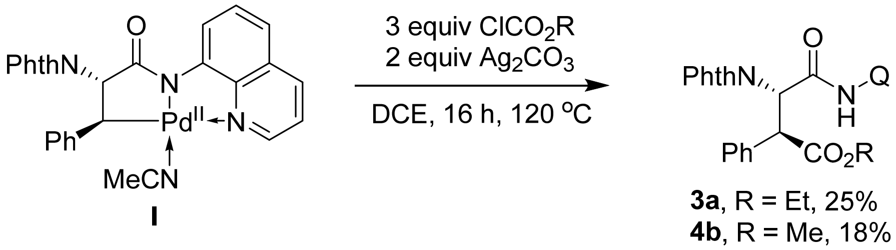

To a 50 mL Schlenk tube were added **I** (28.4 mg, 0.05 mmol), Ag_2CO_3 (27.3 mg, 0.1 mmol) and $ClCO_2Et$ (0.15 mmol, 14.5 μL) in DCE (1.0 mL). The tube was sealed under air. The mixture was stirred at room temperature for 5 minutes then heated at 120 °C for 16 h. After cooling to room temperature, the reaction mixture was diluted with EtOAc (5 mL) and filtered through a short pad of Celite. After concentration in

vacuo, the crude reaction mixture was purified by silica gel column chromatography (petroleum ether: dichloromethane: ethyl acetate = 7: 1: 2, R_f = 0.5). $[\alpha]^{20}_D$ = -127.5 (1.0 M in $CHCl_3$); **3a** was obtained as a pale yellow solid (6.1 mg, 25%). ^{1}H NMR (400 MHz, $CDCl_3$) δ 10.17 (s, 1H), 8.70 (dd, *J* = 6.8, 2.1 Hz, 1H), 8.52 (dd, *J* = 4.2, 1.5 Hz, 1H), 8.07 (dd, *J* = 8.3, 1.5 Hz, 1H), 7.72 (dd, *J* = 5.5, 3.1 Hz, 2H), 7.63 (dd, *J* = 5.5, 3.1 Hz, 2H), 7.52 – 7.42 (m, 2H), 7.37 – 7.28 (m, 3H), 7.17 (t, *J* = 7.3 Hz, 2H), 7.11 (t, *J* = 7.2 Hz, 1H), 6.04 (d, *J* = 11.4 Hz, 1H), 5.04 (d, *J* = 11.5 Hz, 1H), 4.29 (dq, *J* = 10.8, 7.1 Hz, 1H), 4.18 (dq, *J* = 10.8, 7.1 Hz, 1H), 1.23 (t, *J* = 7.1 Hz, 3H). ^{13}C NMR (101 MHz, $CDCl_3$) δ 172.0, 167.4, 166.0, 148.4, 138.5, 136.3, 134.5, 134.3, 133.8, 131.3, 128.8, 128.7, 128.1, 127.9, 127.3, 123.6, 122.1, 121.7, 117.0, 61.6, 55.8, 50.0, 14.1. IR (neat): ν 3334, 2924, 2852, 1778, 1722, 1692, 1529, 1487, 1466 , 1430 cm^{-1}; HRMS (ESI): calc. for $C_{29}H_{23}N_3O_5$ $(M+H^+)$: 494.1710; Found: 494.1711.

When $ClCO_2Me$ **2b** was used, the crude reaction mixture was purified by silica gel column chromatography (petroleum ether: dichloromethane: ethyl acetate = 7: 1: 2, R_f= 0.5). $[\alpha]^{20}_D$ = -137.8 (1.0 M in $CHCl_3$); **4b** was obtained as a yellow solid (4.4 mg, 18%). ^{1}H NMR (400 MHz, $CDCl_3$: δ 10.17 (s, 1H), 8.70 (dd, *J* = 6.6, 1.7 Hz, 1H), 8.54 – 8.47 (m, 1H 8.07 (dd, *J* = 8.2, 0.8 Hz, 1H), 7.76 – 7.69 (m, 2H), 7.67 – 7.61 (m, 2H), 7.51 – 7.43 (m, 2H), 7.38 – 7.29 (m, 3H), 7.20 – 7.15 (m, 2H), 7.15 – 7.09 (m, 1H), 6.05 (d, *J* = 11.4 Hz, 1H), 5.06 (d, *J* = 11.4 Hz, 1H), 3.78 (s, 3H); ^{13}C NMR (101 MHz, $CDCl_3$) δ 172.5, 167.4, 165.9, 148.4, 138.4, 136.3, 134.4, 134.3, 133.6, 131.2, 128.8, 128.6, 128.2, 127.8, 127.2, 123.6, 122.1, 121.7, 117.0, 55.7, 52.8, 49.8; . IR (neat): ν 3330, 2925, 2855, 1775, 1721, 1690, 1529, 1486, 1462, 1428 cm^{-1}; HRMS (ESI): calc. for $C_{28}H_{22}N_3O_5$$(M+H^+)$: 480.1554; Found: 480.1561.

2. Experimental Procedures:

2.1 Preparation of Substrates

2.1.1. General Procedures (GP1) for the Preparation of Natural α-Amino Acids

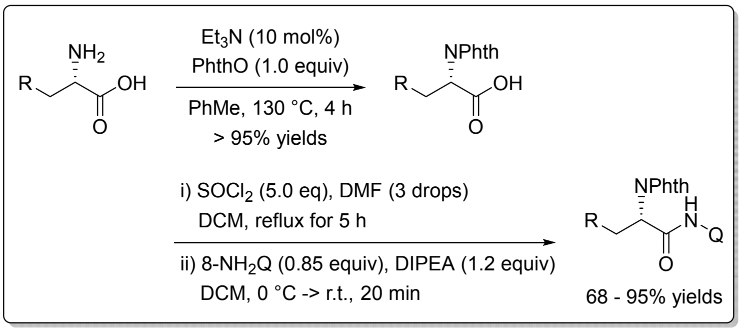

α-Amino acid (0.10 mol), PhthO (14.81 g, 0.10 mol) and Et_3N (1.4 mL, 10 mmol) were added to PhMe (100 mL). The suspension was heated at 130 °C for 4 hours and water produced during the reaction was collected by water separator. After the solution was cooled down to room temperature, the solvent was removed under vaccum. The crude product was then dissolved in DCM (150 mL), and washed by aqueous HCl (0.5-1.0 M, 100 mL) twice and brine (100 mL) once. The combined organic layer was dried over anhydrous $MgSO_4$ and filtered through a pad of Celite and washed by DCM (30 mL). Evaporation of the solvent afforded the corresponding Phth-protected amino acid products in > 95% yields (purity > 95%).

The Phth-protected amino acid (24 mmol), thionyl chloride (8.70 mL, 120 mmol), and 3 drops of DMF were heated in DCM (50 mL) at 55 °C for 5 h. After the reaction, DCM and excess of thionyl chloride were removed under vacuum. The acid chloride was then dissolved in dry DCM (50 mL) and used for the coupling with 8-aminoquinoline (8-NH_2Q).

To a stirred solution of 8-NH_2Q (2.883 g, 20 mmol) and DIPEA (4.76 mL, 28.8 mmol) in dry DCM (50 mL)was added the solution of the acid chloride (24 mmol) in DCM (50 mL) slowly at 0 °C. After the solution was stirred for 20 minutes, the mixture was quenched by DCM (30 mL). Then the mixture was filtered through a pad of Celite to remove the undissolved salts and washed by DCM (30 mL). The collected solution was then washed by aqueous HCl (50 mL, 1 M), saturated $NaHCO_3$ (50 mL), brine (50 mL), and dried over anhydrous $MgSO_4$. Evaporation of the solvent and purification by column

chromatography or recrystallization afforded pure 8-aminoquinoline amides in 68-95% yields.[1] The preparation of **5a**, **1a**, **1r-1w** have been reported.[5]

Chiral HPLC Data for 1a

HPLC Conditions

Chiral Stationary phase: HPLC Chiralpak® AD-H column, n-hexane/isopropanol = 55:45, flow rate = 0.90 mL/min, λ = 220 nm, 16.2 (minor), 24.0 (major), 99% ee.

2.1.2. General Procedures (GP2) for the Preparation of Substituted Phenylalanines

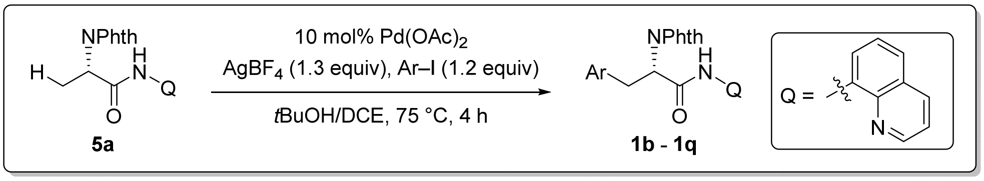

All the substituted phenylalanine substrates were prepared according to the monoarylation method for alanine derivative we have previously reported.[1] To a 100-mL vial was added **0** (690.7 mg, 2.0 mmol), $Pd(OAc)_2$ (44.5 mg, 0.2 mmol), aryl iodide (628.8 mg, 2.4 mmol), $AgBF_4$ (506.2 mg, 2.6 mmol), and t-BuOH/DCE (10 mL + 5 mL). The mixture was stirred at 75 °C for 4 hours. After cooling to room temperature, the reaction was diluted with dichloromethane (50 mL) and triethylamine (2 mL) was added to the mixture. After the mixture was maintained for 6 hours, it was then filtered through a pad of Celite and washed by DCM (60 mL). The filtrate was washed by water (50 mL), and the aqueous phase was extracted with dichloromethane (2 × 30 mL). The combined organic phase was then washed by brine (50 mL), and dried over anhydrous $MgSO_4$. Evaporation of organic solvent and purification by column chromatography (petroleum ether: ethyl acetate: dichloromethane system) gave the corresponding products. Compounds **1b-1p** are known compounds.[5]

(*S*)-3-(2,3-Dihydrobenzo[*b*][1,4]dioxin-6-yl)-2-(1,3-dioxoisoindolin-2-yl)-*N*-(quinolin-8-yl)propanamide (1q)

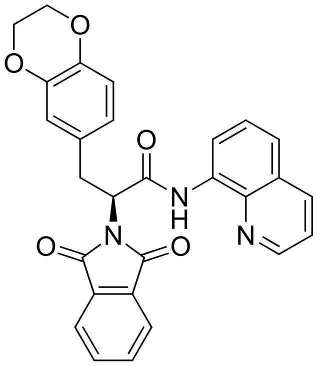

The title compound **1q** was prepared according to **GP1** and obtained as a white solid (758 mg, 79%).$[\alpha]^{20}_{D}$ = -82.2 (1.0 M in $CHCl_3$); ^1H NMR (400 MHz, $CDCl_3$) δ 10.29 (s, 1H), 8.78 – 8.66 (m, 1H), 8.62 (dd, J = 4.1, 1.2 Hz, 1H), 8.11 (d, J = 8.2 Hz, 1H), 7.88 – 7.77 (m, 2H), 7.76 – 7.65 (m, 2H), 7.55 – 7.44 (m, 2H), 7.38 (dd, J = 8.2, 4.2 Hz, 1H), 6.83 (d, J = 1.9 Hz, 1H), 6.76 (dd, J = 8.3, 2.0 Hz, 1H), 6.71 (d, J = 8.2 Hz, 1H), 5.39 (dd, J = 9.9, 6.6 Hz, 1H), 4.16 (s, 3H), 3.80 – 3.60 (m, 2H). ^{13}C NMR (101 MHz, $CDCl_3$) δ 168.1, 166.6, 148.4, 143.7, 142.6, 138.6, 136.4, 134.3, 134.0, 131.9, 130.0, 128,0, 127.4, 123.7, 122.1, 122.0, 121.7, 118.0, 117.5, 116.9, 64.4, 56.5, 34.3, 31.1; IR (neat): ν 3329, 2923, 2859, 1774, 1715, 1532, 1459 cm^{-1}; HRMS (ESI): calc. for $C_{28}H_{22}N_3O_5$ $(M+H^+)$: 480.1554; Found: 480.1561.

2.1.3. Preparation of Substrates 5b-5o

Substrates **5b-5o** were prepared according to literature.[6]

2.2 Optimization of Reaction Conditions for Carbonylation of Methylene C(sp^3)–H Bonds

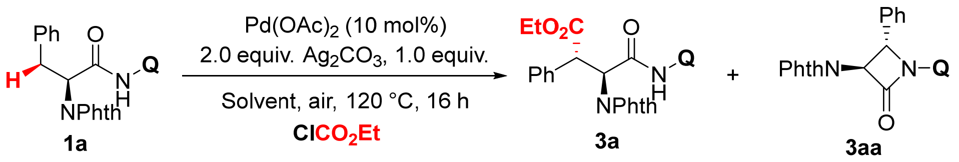

2.3. General Procedure (GP3) for Alkoxycarbonylation of Methylene C(sp^3)–H Bonds

To a 50 mL Schlenk tube, were added **1** (0.15 mmol), $Pd(OAc)_2$ (3.5 mg, 0.015 mmol), Ag_2CO_3 (82.7 mg, 0.3 mmol), I_2 (38.0 mg, 1.0 equiv.), $ClCO_2R$ (0.45 mmol, 3.0 equiv.) and toluene (2.0 mL). The tube was sealed under air. The mixture was stirred at room temperature for 5 minutes then heated at 120 °C for 16 h. After cooling to room temperature, the reaction mixture was diluted with EtOAc (10 mL) and

filtered through a pad of Celite. After concentration in vacuo, the crude reaction mixture was purified by silica gel flash chromatography.

(2S,3S)-Ethyl 3-(1,3-dioxoisoindolin-2-yl)-4-oxo-2-phenyl-4-(quinolin-8-ylamino)butanoate (3a)

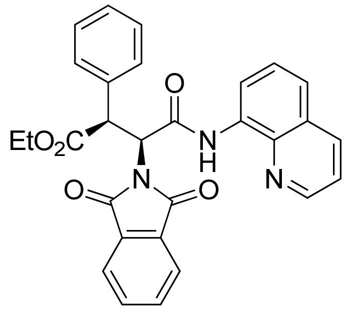

1) Reaction time: 16 h

Phenylalanine derivative **1a** (99% ee) was used as the starting material. The title compound **3a** was prepared under the optimized conditions (16 h) and purified by column chromatography (petroleum ether: dichloromethane: ethyl acetate = 7: 1: 2, R_f = 0.5). **3a** was obtained as a pale yellow solid (56.3 mg, 76%). $[\alpha]^{20}_D$ = -127.5 (1.0 M in $CHCl_3$); ^1H NMR (400 MHz, $CDCl_3$) δ 10.17 (s, 1H), 8.70 (dd, *J* = 6.8, 2.1 Hz, 1H), 8.52 (dd, *J* = 4.2, 1.5 Hz, 1H), 8.07 (dd, *J* = 8.3, 1.5 Hz, 1H), 7.72 (dd, *J* = 5.5, 3.1 Hz, 2H), 7.63 (dd, *J* = 5.5, 3.1 Hz, 2H), 7.52 – 7.42 (m, 2H), 7.37 – 7.28 (m, 3H), 7.17 (t, *J* = 7.3 Hz, 2H), 7.11 (t, *J* = 7.2 Hz, 1H), 6.04 (d, *J* = 11.4 Hz, 1H), 5.04 (d, *J* = 11.5 Hz, 1H), 4.29 (dq, *J* = 10.8, 7.1 Hz, 1H), 4.18 (dq, *J* = 10.8, 7.1 Hz, 1H), 1.23 (t, *J* = 7.1 Hz, 3H). ^{13}C NMR (101 MHz, $CDCl_3$) δ 172.0, 167.4, 166.0, 148.4, 138.5, 136.3, 134.5, 134.3, 133.8, 131.3, 128.8, 128.7, 128.1, 127.9, 127.3, 123.6, 122.1, 121.7, 117.0, 61.6, 55.8, 50.0, 14.1. IR (neat): ν 3334, 2924, 2852, 1778, 1722, 1692, 1529, 1487, 1466 , 1430 cm^{-1}; HRMS (ESI): calc. for $C_{29}H_{23}N_3O_5$ $(M+H^+)$: 494.1710; Found: 494.1711. HPLC Chiralpak® AD-H column, n-hexane/isopropanol = 55:45, flow rate = 0.90 mL/min, λ = 220 nm, 20.6 (minor), 36.5 (major), 99% ee.

2) Reaction time: 24 h

Phenylalanine derivative **1a** (99% ee) was used as the starting material. The title compound **3a** was prepared under the optimized conditions (24 h) and purified by column chromatography (petroleum ether:

dichloromethane: ethyl acetate = 7: 1: 2, $R_f = 0.5$). **3a** was obtained as a pale yellow solid (37.8 mg, 51%). HPLC Chiralpak® AD-H column, n-hexane/isopropanol = 55:45, flow rate = 0.90 mL/min, λ = 220 nm, 20.4 (minor), 38.4 (major), 99% ee.

3) Reaction time: 48 h

Phenylalanine derivative **1a** (99% ee) was used as the starting material. The title compound **3a** was prepared under the optimized conditions (48 h) and purified by column chromatography (petroleum ether: dichloromethane: ethyl acetate = 7: 1: 2, $R_f = 0.5$). **3a** was obtained as a pale yellow solid (37.1 mg, 50%). HPLC Chiralpak® AD-H column, n-hexane/isopropanol = 55:45, flow rate = 0.90 mL/min, λ = 220 nm, 21.5 (minor), 38.8 (major), 99% ee.

2-((3S,4S)-2-oxo-4-phenyl-1-(quinolin-8-yl)azetidin-3-yl)isoindoline-1,3-dione (**3aa**)

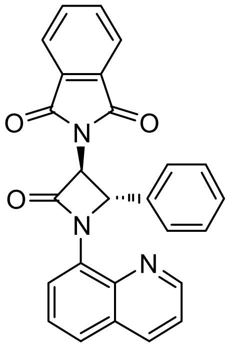

The title compound **3aa** was prepared under the standard conditions (using 1.0 equiv Ag_2CO_3 instead) purified by column chromatography (petroleum ether: dichloromethane: ethyl acetate = 7: 1: 2). **3aa** was obtained as a pale yellow solid (20.0 mg, 30%). ^1H NMR (400 MHz, $CDCl_3$) δ 8.69 (dd, J = 4.1, 1.6 Hz, 1H), 8.28 (dd, J = 7.2, 1.3 Hz, 1H), 7.96 (dd, J = 8.3, 1.6 Hz, 1H), 7.83 (dd, J = 5.5, 3.0 Hz, 2H), 7.70 (dd, J = 5.4, 3.1 Hz, 2H), 7.54 (dd, J = 8.1, 1.4 Hz, 1H), 7.52 – 7.46 (m, 1H), 7.42 (d, J = 7.2 Hz, 2H), 7.25 – 7.12 (m, 4H), 6.58 (d, J = 2.7 Hz, 1H), 5.45 (d, J = 2.7 Hz, 1H). ^{13}C NMR (101 MHz, $CDCl_3$) δ 167.0, 163.5, 149.1, 141.0, 138.4, 135.6, 134.5, 132.4, 131.7, 129.0, 128.8, 128.3, 126.4, 126.7, 125.1, 123.7, 122. 1, 121.4, 65.5, 63.2; HRMS (EI-TOF) calc. for $C_{26}H_{17}N_3O_3$ (M^+):419.1270; Found: 419.1274.

(2*S*,3*S*)-Ethyl 3-(1,3-dioxoisoindolin-2-yl)-4-oxo-4-(quinolin-8-ylamino)-2-(*p*-tolyl)butanoate (3b)

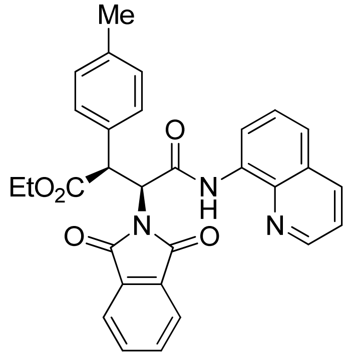

The title compound **3b** was prepared according to **GP3** and was purified by column chromatography (petroleum ether: dichloromethane: ethyl acetate = 7: 1: 2, R_f = 0.6). $[\alpha]^{20}_D$ = -108.5 (0.5 M in $CHCl_3$); **3b** was obtained as pale yellow solid (80.6 mg, 60%). ^1H NMR (400 MHz, $CDCl_3$) δ 10.17 (s, 1H), 8.70 (dd, *J* = 6.8, 1.9 Hz, 1H), 8.53 (dd, *J* = 4.1, 1.2 Hz, 1H), 8.08 (dd, *J* = 8.2, 1.2 Hz, 1H), 7.74 (dd, *J* = 5.3, 3.1 Hz, 2H), 7.64 (dd, *J* = 5.3, 3.1 Hz, 2H), 7.48 (q, *J* = 8.2 Hz, 2H), 7.33 (dd, *J* = 8.2, 4.2 Hz, 1H), 7.22 (d, *J* = 7.9 Hz, 2H), 6.97 (d, *J* = 7.9 Hz, 2H), 6.00 (d, *J* = 11.5 Hz, 1H), 5.01 (d, *J* = 11.5 Hz, 1H), 4.28 (dq, *J* = 10.8, 7.1 Hz, 1H), 4.16 (dq, *J* = 10.6, 7.0 Hz, 1H), 2.17 (s, 3H), 1.23 (t, *J* = 7.1 Hz, 3H); ^{13}C NMR (101 MHz, $CDCl_3$) δ 172.2, 167.5, 166.1, 148.4, 138.6, 137.8, 136.3, 134.3, 133.9, 131.5, 131.4, 129.5, 128.5, 127.9, 127.3, 123.7, 122.1, 121.7, 117.1, 61.6, 55.9, 49.6, 21.1, 14.1; IR (neat): ν 3331, 2923, 2857, 1774, 1725, 1650, 1530, 1459 cm^{-1}; HRMS (ESI): calc. for $C_{30}H_{26}N_3O_5$ $(M+H^+)$: 508.1867; Found: 508.1873.

(2*S*,3*S*)-Ethyl 3-(1,3-dioxoisoindolin-2-yl)-2-(ethylphenyl)-4-oxo-4-(quinolin-8-ylamino) butanoate (3c)

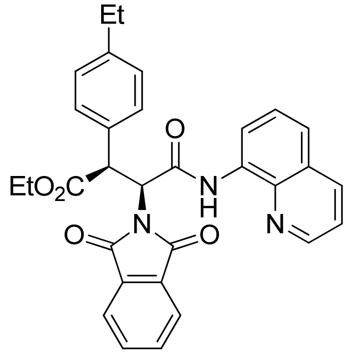

The title compound **3c** was prepared according to **GP3** and was purified by column chromatography (petroleum ether: dichloromethane: ethyl acetate = 7: 1: 2, R_f = 0.6). $[\alpha]^{20}_D$ = -149.7 (1.0 M in $CHCl_3$);**3c** was obtained as a white solid (54.8 mg, 70%). ^{1}H NMR (400 MHz, $CDCl_3$): δ 10.17 (s, 1H), 8.71 (dd, *J* = 6.9, 1.7 Hz, 1H), 8.53 (dd, *J* = 4.1, 1.4 Hz, 1H), 8.07 (dd, *J* = 8.2, 1.4 Hz, 1H), 7.73 (dd, *J* = 5.3, 3.1 Hz, 2H), 7.63 (dd, *J* = 5.4, 3.0 Hz, 2H), 7.55 – 7.41 (m, 2H), 7.33 (dd, *J* = 8.2, 4.2 Hz, 1H), 7.29 – 7.20 (m, 2H), 6.98 (d, *J* = 7.9 Hz, 2H), 6.01 (d, *J* = 11.4 Hz, 1H), 5.00 (d, *J* = 11.4 Hz, 1H), 4.30 (dq, *J* = 10.6, 7.1 Hz, 1H), 4.17 (dq, *J* = 10.8, 7.1 Hz, 1H), 2.46 (q, *J* = 7.5 Hz, 2H), 1.24 (t, *J* = 7.1 Hz, 3H), 1.04 (t, *J* = 7.6 Hz, 3H). ^{13}C NMR (101 MHz, $CDCl_3$) δ 172.2, 167.5, 166.1, 148.4, 144.1, 138.5, 136.3, 134.2, 133.9, 131.7, 131.4, 128.6, 128.2, 127.9, 127.3, 123.6, 122.0, 121.6, 117.0, 61.6, 55.9, 49.7, 28.5, 15.4, 14.1; IR (neat): ν 3335, 2924, 2853, 1775, 1725, 1650, 1531, 1487, 1463, 1426 cm^{-1}; HRMS (ESI): calc. for $C_{31}H_{28}N_3O_5$ (M+H^+): 522.2023; Found: 522.2026.

(2*S*,3*S*)-Ethyl 2-(4-(*tert*-butyl)phenyl)-3-(1,3-dioxoisoindolin-2-yl)-4-oxo-4-(quinolin-8-ylamino)butanoate (3d)

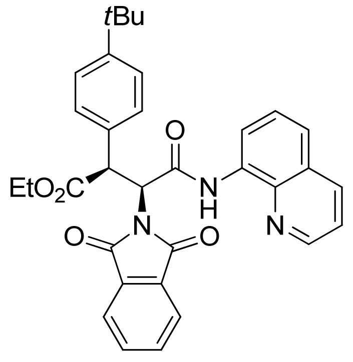

The title compound **3d** was prepared according to **GP3** and was purified by column chromatography (petroleum ether: dichloromethane: ethyl acetate = 7: 1: 2, R_f = 0.6). $[\alpha]^{20}_D$ = -134.5 (0.5 M in $CHCl_3$); **3d** was obtained as a colourless oil (53.4 mg, 65%). ^{1}H NMR (400 MHz, $CDCl_3$) δ 10.16 (s, 1H), 8.72 (dd, *J* = 7.0, 1.4 Hz, 1H), 8.59 – 8.45 (m, 1H), 8.07 (d, *J* = 8.2 Hz, 1H), 7.70 (dd, *J* = 5.3, 3.1 Hz, 2H), 7.62 (dd, *J* = 5.3, 3.1 Hz, 2H), 7.55 – 7.42 (m, 2H), 7.32 (dd, *J* = 8.2, 4.2 Hz, 1H), 7.23 (d, *J* = 8.2 Hz, 2H), 7.13 (d, *J* = 8.3 Hz, 2H), 6.02 (d, *J* = 11.3 Hz, 1H), 4.98 (d, *J* = 11.3 Hz, 1H), 4.31 (dq, *J* = 10.6, 7.1 Hz, 1H), 4.17 (dq, *J* = 10.7, 7.1 Hz, 1H), 1.25 (t, *J* = 7.1 Hz, 3H), 1.11 (s, 9H). ^{13}C NMR (101 MHz,

$CDCl_3$) δ 172.1, 167.5, 166.1, 151.0, 148.4, 138.5, 136.2, 134.2, 133.9, 131.4, 131.4, 128.4, 127.9, 127.3, 125.5, 123.5, 122.0, 121.6, 117.0, 61.6, 56.0, 49.7, 34.5, 31.2, 14.1; IR (neat): ν 3337, 2923, 2859, 1776, 1726, 1690, 1530, 1460 cm^{-1}; HRMS (ESI): calc. for $C_{33}H_{32}N_3O_5$ $(M+H^+)$: 550.2336; Found: 550.2334.

(2*S*,3*S*)-Ethyl 3-(1,3-dioxoisoindolin-2-yl)-2-(2-methoxyphenyl)-4-oxo-4-(quinolin-8-ylamino)butanoate (3e)

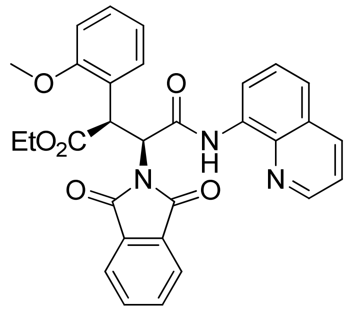

The title compound **3e** was prepared under the optimized conditions and purified by column chromatography (petroleum ether: dichloromethane: ethyl acetate = 7: 1: 2, $R_f = 0.4$). $[\alpha]^{20}_D$ = -154.7 (1.0 M in $CHCl_3$); 3e was obtained as a pale yellow solid (40.0 mg, 51%). ^{1}H NMR (400 MHz, $CDCl_3$) δ 10.12 (s, 1H), 8.77 (dd, *J* = 7.3, 1.1 Hz, 1H), 8.61 (d, *J* = 2.9 Hz, 1H), 8.07 (dd, *J* = 8.2, 1.2 Hz, 1H), 7.73 – 7.67 (m, 2H), 7.65 – 7.59 (m, 2H), 7.53 – 7.43 (m, 2H), 7.39 – 7.31 (m, 2H), 6.02 (d, *J* = 10.9 Hz, 1H), 5.34 (d, *J* = 11.0 Hz, 1H), 4.32 – 4.16 (m, 2H), 3.62 (s, 3H), 1.21 (t, *J* = 7.1 Hz, 3H). ^{13}C NMR (101 MHz, $CDCl_3$) δ 172.0, 167.1, 166.2, 157.4, 148.3, 138.5, 136.2, 134.2, 134.1, 131.5, 131.0, 129.3, 127.9, 127.4, 123.4, 123.3, 121.8, 121.6, 120.7, 117.1, 110.6, 61.3, 55.3, 54.7, 14.2; IR (neat): ν 3341, 2924, 2854 1775, 1723, 1690, 1529, 1488, 1463, 1426 cm^{-1}; HRMS (ESI): calc. for $C_{30}H_{26}N_3O_6$ $(M+H^+)$: 524.1816; Found: 524.1822.

3-(1,3-dioxoisoindolin-2-yl)-2-(3-methoxyphenyl)-4-oxo-4-(quinolin-8-ylamino)butanoate (3f)

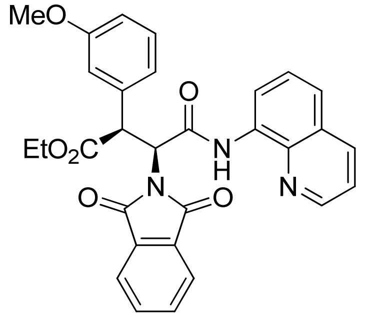

The title compound **3f** was prepared according to **GP3** and was purified by column chromatography (petroleum ether: dichloromethane: ethyl acetate = 7: 1: 2, R_f = 0.4). $[\alpha]^{20}_D$ = -157.5 (1.0 M in $CHCl_3$); **3f** was obtained as a white solid (60.2 mg, 77%). ^1H NMR (400 MHz, $CDCl_3$) δ 10.16 (s, 1H), 8.70 (dd, *J* = 6.9, 1.8 Hz, 1H), 8.57 – 8.48 (m, 1H), 8.07 (d, *J* = 8.2 Hz, 1H), 7.74 (dd, *J* = 5.4, 3.0 Hz, 2H), 7.64 (dd, *J* = 5.4, 3.0 Hz, 2H), 7.54 – 7.42 (m, 2H), 7.32 (dd, *J* = 8.2, 4.2 Hz, 1H), 7.07 (t, *J* = 7.9 Hz, 1H), 6.98 – 6.86 (m, 2H), 6.65 (dd, *J* = 7.9, 1.9 Hz, 1H), 6.03 (d, *J* = 11.4 Hz, 1H), 5.02 (d, *J* = 11.4 Hz, 1H), 4.30 (dq, *J* = 10.8, 7.1 Hz, 1H), 4.19 (dq, *J* = 10.8, 7.1 Hz, 1H), 3.70 (s, 3H), 1.25 (t, *J* = 7.1 Hz, 3H). ^{13}C NMR (101 MHz, $CDCl_3$) δ 171.9, 167.4, 165.9, 159.7, 148.4, 138.5, 136.3, 136.0, 134.3, 133.8, 131.4, 129.7, 127.9, 127.3, 123.6, 122.1, 121.6, 121.2, 117.0, 114.6, 113.3, 61.7, 55.8, 55.31, 45.0, 14.1; IR (neat): ν 3332, 2923, 2855, 1775, 1722, 1692 , 1599, 1529, 1463, 1460 cm^{-1}; HRMS (ESI): calc. for $C_{30}H_{26}N_3O_6$ ($M+H^+$): 524.1816; Found: 524.1819.

(2*S*,3*S*)-Ethyl 3-(1,3-dioxoisoindolin-2-yl)-2-(4-methoxyphenyl)-4-oxo-4-(quinolin-8-ylamino)butanoate (3g)

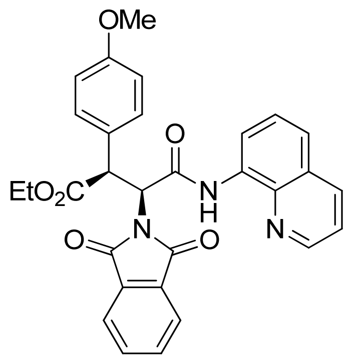

The title compound **3g** was prepared according to **GP3** and was purified by column chromatography (petroleum ether: dichloromethane: ethyl acetate = 7: 1: 2, R_f = 0.4). $[\alpha]^{20}_D$ = -128.4 (1.0 M in $CHCl_3$);

3e was obtained as a white solid (49.9 mg, 64%). ^{1}H NMR (400 MHz, $CDCl_3$) δ 10.16 (s, 1H), 8.70 (dd, *J* = 6.8, 2.0 Hz, 1H), 8.52 (dd, *J* = 4.1, 1.6 Hz, 1H), 8.07 (dd, *J* = 8.3, 1.5 Hz, 1H), 7.74 (dd, *J* = 5.5, 3.0 Hz, 2H), 7.64 (dd, *J* = 5.4, 3.1 Hz, 2H), 7.51 – 7.41 (m, 2H), 7.32 (dd, *J* = 8.2, 4.2 Hz, 1H), 7.29-7.23 (m, 2H), 6.70 (d, *J* = 8.7 Hz, 2H), 5.99 (d, *J* = 11.5 Hz, 1H), 4.99 (d, *J* = 11.5 Hz, 1H), 4.28 (dq, *J* = 10.9, 7.1 Hz, 1H), 4.17 (dq, *J* = 10.8, 7.1 Hz, 1H), 3.66 (s, 3H), 1.23 (t, *J* = 7.1 Hz, 4H). ^{13}C NMR (101 MHz, $CDCl_3$) δ 172.3, 167.5, 166.0, 159.3, 148.4, 138.5, 136.2, 134.3, 133.9, 131.4, 129.8, 127.9, 127.3, 126.6, 123.7, 122.1, 121.6, 117.0, 114.2, 61. 6, 55.8, 55.2, 49.2, 14.1; IR (neat): ν 3333, 2930, 2815, 1775, 1723, 1650, 1613, 1529, 1485, 1426 cm^{-1}; HRMS (ESI): calc. for $C_{30}H_{26}N_3O_6$ $(M+H^+)$: 524.1816; Found: 524.1827.

(2*S*,3*S*)-Ethyl 3-(1,3-dioxoisoindolin-2-yl)-4-oxo-4-(quinolin-8-ylamino)-2-(4-(trifluoromethyl)phenyl)butanoate (3h)

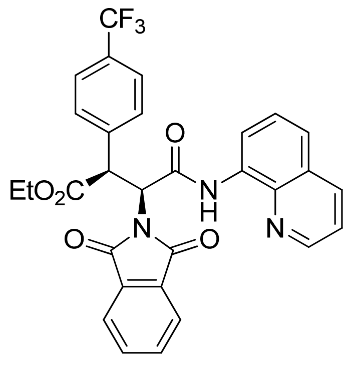

The title compound **3h** was prepared according to **GP3** and was purified by column chromatography (petroleum ether: tetrahydrofuran = 3: 1, R_f = 0.4). $[\alpha]^{20}_{D}$ = -123.3 (1.0 M in $CHCl_3$); **3h** was obtained as a colourless oil (54.6 mg, 65%). ^{1}H ^{1}H NMR (400 MHz, $CDCl_3$) δ 10.18 (s, 1H), 8.69 (dd, *J* = 6.2, 2.7 Hz, 1H), 8.53 (dd, *J* = 4.2, 1.7 Hz, 1H), 8.09 (dd, *J* = 8.3, 1.6 Hz, 1H), 7.75 (dd, *J* = 5.6, 3.0 Hz, 2H), 7.67 (dd, *J* = 5.5, 3.1 Hz, 2H), 7.57 – 7.40 (m, 6H), 7.35 (dd, *J* = 8.3, 4.3 Hz, 1H), 6.04 (d, *J* = 11.5 Hz, 1H), 5.15 (d, *J* = 11.5 Hz, 1H), 4.30 (dq, *J* = 10.8, 7.1 Hz, 1H), 4.18 (dq, *J* = 10.8, 7.1 Hz, 1H), 1.24 (t, *J* = 7.1 Hz, 3H); ^{19}F NMR (376 MHz, $CDCl_3$) δ -62.8; ^{13}C NMR (101 MHz, $CDCl_3$) δ 171.4, 167.4, 165.6, 148.5, 138.7, 138.5, 136.3, 134.6, 133.7, 131.2, 130. 4 (q, $J_{C\text{-}F}$ = 32.3 Hz), 129.2, 127.9, 127.3, 125.8 (q, $J_{C\text{-}F}$ = 3.7 Hz), 123.9 (q, $J_{C\text{-}F}$ =273.6 Hz), 123.8, 122.3, 121.7, 117.1, 62.0, 55.5, 49.9, 14.1; IR (neat): ν 3332,

2923, 2857, 1776, 1725, 1618, 1531, 1460 cm^{-1}; HRMS (ESI): calc. for $C_{30}H_{23}F_3N_3O_5$ $(M+H^+)$: 562.1584; Found: 562.1589.

(2*S*,3*S*)-Ethyl 2-(4-acetylphenyl)-3-(1,3-dioxoisoindolin-2-yl)-4-oxo-4-(quinolin-8-ylamino)butanoate (3i)

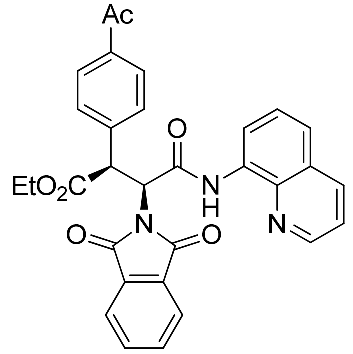

The title compound **3i** was prepared according to **GP3** and was purified by column chromatography (petroleum ether: dichloromethane: ethyl acetate = 7: 1: 2, R_f = 0.3). $[\alpha]^{20}_D$ = -168.9 (1.0 M in $CHCl_3$); **3i** was obtained as a white solid (58.5 mg, 73%). ^{1}H NMR (400 MHz, $CDCl_3$) δ 10.17 (s, 1H), 8.68 (dd, *J* = 6.3, 2.7 Hz, 1H), 8.53 (dd, *J* = 4.2, 1.3 Hz, 1H), 8.18 – 8.00 (m, 1H), 7.78 (d, *J* = 8.3 Hz, 2H), 7.74 (dd, *J* = 5.5, 3.1 Hz, 2H), 7.66 (dd, *J* = 5.5, 3.1 Hz, 2H), 7.54 – 7.42 (m, 4H), 7.34 (dd, *J* = 8.3, 4.2 Hz, 1H), 6.05 (d, *J* = 11.5 Hz, 1H), 5.15 (d, *J* = 11.5 Hz, 1H), 4.29 (dq, *J* = 10.8, 7.1 Hz, 1H), 4.18 (dq, *J* = 10.8, 7.1 Hz, 1H), 2.49 (s, 3H), 1.23 (t, *J* = 7.2 Hz, 3H). ^{13}C NMR (101 MHz, $CDCl_3$) δ 197.8, 171.4, 167.4, 165.6, 148.5, 139.9, 138.5, 136.8, 136.3, 134.6, 133.7, 131.2, 129.0, 128.9, 127. 9, 127.3, 123.8, 122.2, 121.7, 117.1, 62.0, 55.5, 50.0, 26.7, 14.1; IR (neat): ν 3333, 2927, 2855, 1775, 1723, 1688, 1651, 1610, 1527, 1488, 1423cm^{-1}; HRMS (ESI): calc. for $C_{31}H_{26}N_3O_6$ $(M+H^+)$: 536.1816; Found: 536.1819.

Methyl 4-((2*S*,3*S*)-3-(1,3-dioxoisoindolin-2-yl)-1-ethoxy-1,4-dioxo-4-(quinolin-8-ylamino)butan-2-yl)benzoate (3j)

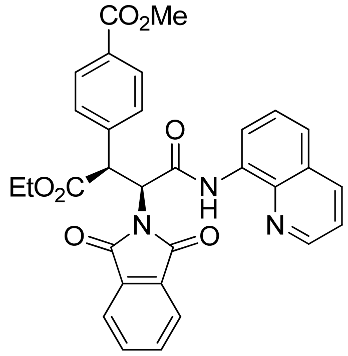

The title compound **3j** was prepared according to **GP3** and was purified by column chromatography (petroleum ether: ethyl acetate = 2: 1, R_f = 0.4). $[\alpha]^{20}_D$ = -159.2 (0.5 M in $CHCl_3$); **3j** was obtained as a white foam (39.7 mg, 48%). ^1H NMR (400 MHz, $CDCl_3$): δ 10.17 (s, 1H), 8.69 (dd, *J* = 6.4, 2.6 Hz, 1H), 8.53 (dd, *J* = 4.2, 1.6 Hz, 1H), 8.09 (dd, *J* = 8.3, 1.6 Hz, 1H), 7.86 (d, *J* = 8.3 Hz, 2H), 7.73 (dt, *J* = 6.9, 3.5 Hz, 2H), 7.71 – 7.61 (m, 2H), 7.53 – 7.46 (m, 2H), 7.44 (d, *J* = 8.3 Hz, 2H), 7.34 (dd, *J* = 8.3, 4.3 Hz, 1H), 6.04 (d, *J* = 11.5 Hz, 1H), 5.14 (d, *J* = 11.5 Hz, 1H), 4.28 (dq, *J* = 10.8, 7.1 Hz, 1H), 4.19 (dq, *J* = 10.8, 7.1 Hz, 1H), 3.83 (s, 3H), 1.22 (t, *J* = 7.1 Hz, 3H). ^{13}C NMR (101 MHz, $CDCl_3$) δ 171.4, 167.4, 166.8, 165.6, 148.5, 139.8, 138.5, 136.3, 134.5, 133.7, 131.2, 130.1, 130.0, 128.8, 127. 9, 127. 3, 123.8, 122.2, 121.7, 117.1, 61.9, 55.5, 52.3, 50.0, 14.1; IR (neat): ν 3335, 2925, 2856, 1775, 1724, 1692, 1531, 1529, 1463 cm^{-1}; HRMS (ESI): calc. for $C_{31}H_{26}N_3O_6$ $(M+H^+)$: 552.1765; Found: 552.1768.

(2*S*,3*S*)-Ethyl 3-(1,3-dioxoisoindolin-2-yl)-2-(3-fluorophenyl)-4-oxo-4-(quinolin-8-ylamino)butanoate (3k)

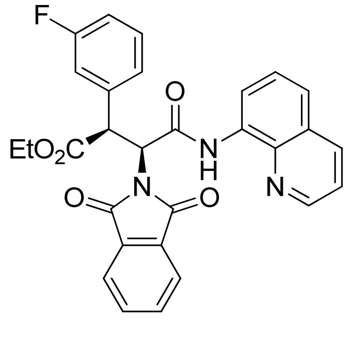

The title compound **3k** was prepared according to **GP3** and was purified by column chromatography (petroleum ether: dichloromethane: ethyl acetate = 7: 1: 2, R_f = 0.5). $[\alpha]^{20}_D$ = -151.8 (1.0 M in $CHCl_3$); 3**k** was obtained as a colourless oil (46.4 mg, 61%). ^1H NMR (400 MHz, $CDCl_3$): δ 10.16 (s, 1H), 8.68

(dd, J = 6.4, 2.0 Hz, 1H), 8.52 (d, J = 3.8 Hz, 1H), 8.07 (d, J = 8.1 Hz, 1H), 7.76 (dd, J = 5.0, 3.0 Hz, 2H), 7.66 (dd, J = 5.2, 3.1 Hz, 2H), 7.54 – 7.43 (m, 2H), 7.33 (dd, J = 8.2, 4.2 Hz, 1H), 7.21 – 7.04 (m, 3H), 6.91 – 6.73 (m, 1H), 6.00 (d, J = 11.4 Hz, 1H), 5.05 (d, J = 11.5 Hz, 1H), 4.30 (dq, J = 10.5, 7.1 Hz, 1H), 4.20 (dq, J = 10.9, 7.0 Hz, 1H), 1.24 (t, J = 7.0 Hz, 3H); ^{19}F NMR (376 MHz, $CDCl_3$) δ -111.9; ^{13}C NMR (101 MHz, $CDCl_3$) δ 171.5, 167.4, 165.7, 162.7 (d, J_{C-F} = 247.2 Hz), 148.4, 138.5, 137.0 (d, J_{C-F} = 7.5 Hz), 136.3, 134.5, 133.8, 131.3, 130.3 (d, J_{C-F} = 8.2 Hz), 127.9, 127.3, 124.4 (d, J_{C-F} = 2.9 Hz), 123.8, 122.2, 121.7, 117.1, 115.8 (d, J_{C-F} = 22.2Hz), 115.3 (d, J_{C-F} = 21.0 Hz), 61.8, 55.7, 49.7, 14.1; IR (neat): ν 3333, 2923, 2856, 1776, 1725, 1692, 1592, 1530, 1458 cm^{-1}; HRMS (ESI): calc. for $C_{29}H_{23}FN_3O_6$ (M+H$^+$): 512.1616; Found: 516.1622.

(2*S*,3S)-Ethyl 3-(1,3-dioxoisoindolin-2-yl)-2-(4-fluorophenyl)-4-oxo-4-(quinolin-8-ylamino)butanoate (3l)

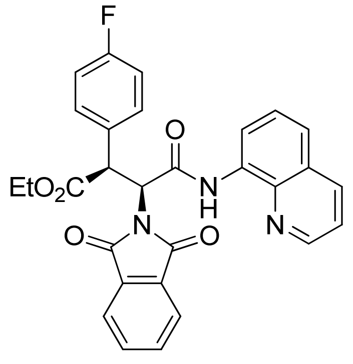

The title compound **3l** was prepared according to **GP3** and was purified by column chromatography (petroleum ether: dichloromethane: ethyl acetate = 7: 1: 2, R_f= 0.5). $[\alpha]^{20}_D$ = -123.6 (0.5 M in $CHCl_3$); **3l** was obtained as a white solid (38.8 mg, 51%). ^{1}H NMR (400 MHz, $CDCl_3$): δ 10.16 (s, 1H), 8.69 (dd, J = 6.5, 2.4 Hz, 1H), 8.52 (dd, J = 4.2, 1.5 Hz, 1H), 8.08 (dd, J = 8.3, 1.5 Hz, 1H), 7.75 (dt, J = 7.0, 3.5 Hz, 2H), 7.70 – 7.65 (m, 2H), 7.55 – 7.44 (m, 2H), 7.38 – 7.27 (m, 3H), 6.90 – 6.83 (m, 2H), 5.99 (d, J = 11.5 Hz, 1H), 5.03 (d, J = 11.5 Hz, 1H), 4.29 (dq, J = 10.8, 7.1 Hz, 1H), 4.19 (dq, J = 10.8, 7.1 Hz, 1H), 1.23 (t, J = 7.1 Hz, 3H); ^{19}F NMR (376 MHz, $CDCl_3$) δ -113.8(s, 1F); ^{13}C NMR (101 MHz, $CDCl_3$) δ 171.9, 167.4, 165. 8, 162. 3(d, J_{C-F} =246.9 Hz), 148.4, 138.5, 136.3, 134.5, 133.8, 131.2, 130.4 (d, J_{C-F} = 8.0 Hz), 130.4, 127. 9, 127.3, 123.8, 122.2, 121.7, 117.1, 115.8 (d, J_{C-F} =21.5 Hz), 61.8, 55.7, 49.3, 14.1; IR

(neat): ν 3358, 2921, 2852, 1755, 1720, 1680, 1531, 1463 cm^{-1}; HRMS (ESI): calc. for $C_{29}H_{23}FN_3O_6$ ($M+H^+$): 512.1616; Found: 512.1628.

(2*S*,3*S*)-Ethyl 2-(4-chlorophenyl)-3-(1,3-dioxoisoindolin-2-yl)-4-oxo-4-(quinolin-8-ylamino)butanoate (3m)

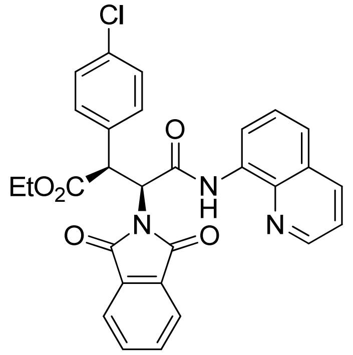

The title compound **3m** was prepared according to **GP3** and was purified by column chromatography (petroleum ether: dichloromethane: ethyl acetate = 3: 2: 1, R_f = 0.4). $[\alpha]^{20}_D$ = -123.0 (0.5 M in $CHCl_3$); **3m** was obtained as a white solid (49.4 mg, 62%). ^1H NMR (400 MHz, $CDCl_3$): δ 10.16 (s, 1H), 8.68 (dd, *J* = 6.4, 2.3 Hz, 1H), 8.52 (d, *J* = 3.0 Hz, 1H), 8.08 (d, *J* = 8.2 Hz, 1H), 7.76 (dd, *J* = 5.3, 3.0 Hz, 2H), 7.67 (dd, *J* = 5.3, 3.1 Hz, 2H), 7.55 – 7.42 (m, 2H), 7.39 – 7.27 (m, 3H), 7.16 (d, *J* = 8.3 Hz, 2H), 5.99 (d, *J* = 11.5 Hz, 1H), 5.05 (d, *J* = 11.5 Hz, 1H), 4.28 (dq, *J* = 10.7, 7.1 Hz, 1H), 4.23 – 4.12 (m, 1H), 1.23 (t, *J* = 7.2 Hz, 3H); ^{13}C NMR (101 MHz, $CDCl_3$) δ 171.7, 167.4, 165.7, 148.4, 138.5, 136.3, 134.5, 134.1, 133.8, 133.2, 131.25, 130.1, 129.1, 127. 9, 127.3, 123.8, 122.2, 121.7, 117.1, 61.8, 55.6, 49.4, 14.1; IR (neat): ν 3338, 2922, 2854, 1774, 1727, 1692, 1615, 1531, 1458 cm^{-1}; HRMS (ESI): calc. for $C_{29}H_{23}ClN_3O_5$ ($M+H^+$): 528.1321; Found: 528.1326.

(2*S*,3*S*)-Ethyl 2-(4-bromophenyl)-3-(1,3-dioxoisoindolin-2-yl)-4-oxo-4-(quinolin-8-ylamino)butanoate (3n)

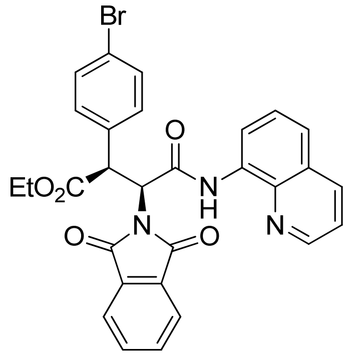

The title compound **3n** was prepared according to **GP3** and was purified by column chromatography (petroleum ether: dichloromethane: ethyl acetate = 7: 1: 2, R_f = 0.5). $[\alpha]^{20}_D$ = -146.2 (1.0 M in $CHCl_3$); **3n** was obtained as a colourless oil (57.3 mg, 67%). ^1H NMR (400 MHz, $CDCl_3$): δ 10.16 (s, 1H), 8.68 (dd, J = 6.4, 2.5 Hz, 1H), 8.52 (dd, J = 4.1, 1.3 Hz, 1H), 8.08 (dd, J = 8.2, 1.3 Hz, 1H), 7.76 (dd, J = 5.4, 3.1 Hz, 2H), 7.71 – 7.63 (m, 2H), 7.55 – 7.43 (m, 2H), 7.38 – 7.27 (m, 3H), 7.24 (d, J = 8.4 Hz, 2H), 5.99 (d, J = 11.5 Hz, 1H), 5.04 (d, J = 11.5 Hz, 1H), 4.28 (dq, J = 10.7, 7.1 Hz, 1H), 4.18 (dq, J = 10.7, 7.1 Hz, 1H), 1.23 (t, J = 7.1 Hz, 3H). ^{13}C NMR (101 MHz, $CDCl_3$) δ 171.6, 167.4, 165.7, 148.4, 138.5, 136.3, 134.5, 133.8, 133.7, 132.0, 131.3, 130.4, 127. 9, 127.3, 123.8, 122.4, 122.2, 121.7, 117.1, 61.8, 55.5, 49.5, 14.1; IR (neat): ν 3338, 2922, 2858, 1775, 1725, 1694, 1531, 1458 cm^{-1}; HRMS (ESI): calc. for $C_{29}H_{23}BrN_3O_5$ $(M+H^+)$: 572.0816; Found: 572.0811.

(2*S*,3*S*)-Ethyl 2-(3,4-dimethylphenyl)-3-(1,3-dioxoisoindolin-2-yl)-4-oxo-4-(quinolin-8-ylamino)butanoate (3o)

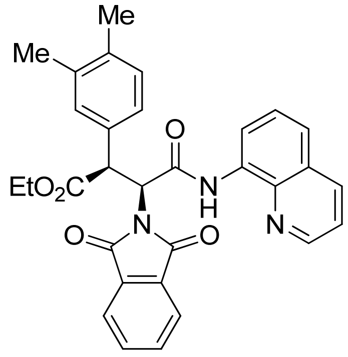

The title compound **3o** was prepared according to **GP3** and was purified by column chromatography (petroleum ether: dichloromethane: ethyl acetate = 7: 1: 2, R_f= 0.6). $[\alpha]^{20}_D$ = -168.2 (0.5 M in $CHCl_3$); **3o** was obtained as a colourless oil (58.0 mg, 74%). ^1H NMR (400 MHz, $CDCl_3$): δ 10.17 (s, 1H), 8.71 (dd,

J = 6.9, 2.1 Hz, 1H), 8.55 (dd, J = 4.1, 1.4 Hz, 1H), 8.08 (d, J = 7.6 Hz, 1H), 7.74 (dt, J = 6.9, 3.5 Hz, 2H), 7.69 – 7.59 (m, 2H), 7.55 – 7.43 (m, 2H), 7.34 (dd, J = 8.3, 4.2 Hz, 1H), 7.07 (d, J = 7.1 Hz, 2H), 6.92 (d, J = 7.7 Hz, 1H), 5.99 (d, J = 11.5 Hz, 1H), 4.98 (d, J = 11.5 Hz, 1H), 4.30 (dq, J = 10.8, 7.1 Hz, 1H), 4.15 (dq, J = 10.8, 7.1 Hz, 1H), 2.08 (s, 3H), 2.07 (s, 3H), 1.24 (t, J = 7.1 Hz, 3H); ^{13}C NMR (101 MHz, $CDCl_3$) δ 172.3, 167.5, 166.1, 148.4, 138.5, 137.0, 136.4, 136.3, 134.3, 133.9, 131.8, 131.5, 130.0, 129.8, 127.9, 127.3, 125.9, 123.6, 122.1, 121.7, 117.0, 61.6, 56.0, 49.5, 19.7, 19.5, 14.1; IR (neat): ν 3337, 2928, 2855, 1775, 1723, 1694, 1530, 1486, 1426 cm^{-1}; HRMS (ESI): calc. for $C_{31}H_{28}N_3O_5$ (M+H$^+$): 522.2023; Found: 522.2035.

(2*S*,3*S*)-Ethyl 2-(3,4-dimethoxyphenyl)-3-(1,3-dioxoisoindolin-2-yl)-4-oxo-4-(quinolin-8-ylamino)butanoate (3p)

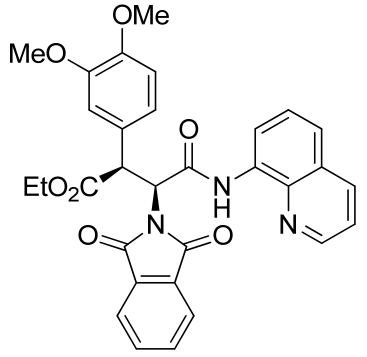

The title compound **3p** was prepared according to **GP3** and was purified by column chromatography (petroleum ether: dichloromethane: ethyl acetate = 7: 1: 2, R_f = 0.3). $[\alpha]^{20}_D$ = -157.6 (1.0 M in $CHCl_3$); **3p** was obtained as white solid (66.8 mg, 81%). ^{1}H NMR (400 MHz, $CDCl_3$): δ 10.16 (s, 1H), 8.69 (dd, J = 6.7, 2.2 Hz, 1H), 8.52 (dd, J = 4.2, 1.6 Hz, 1H), 8.07 (dd, J = 8.3, 1.5 Hz, 1H), 7.73 (dt, J = 7.0, 3.5 Hz, 2H), 7.70 – 7.60 (m, 2H), 7.55 – 7.40 (m, 2H), 7.33 (dd, J = 8.3, 4.2 Hz, 1H), 6.93 – 6.79 (m, 2H), 6.65 (d, J = 8.2 Hz, 1H), 6.01 (d, J = 11.4 Hz, 1H), 4.96 (d, J = 11.4 Hz, 1H), 4.30 (dq, J = 10.8, 7.1 Hz, 1H), 4.18 (dq, J = 10.8, 7.1 Hz, 1H), 3.79 (s, 3H), 3.73 (s, 3H), 1.24 (t, J = 7.1 Hz, 3H); ^{13}C NMR (101 MHz, $CDCl_3$) δ 172.2, 167.5, 166.0, 148.8, 148.6, 148.4, 138.5, 136.3, 134.4, 133.8, 131.3, 127.9, 127.3, 126.8, 123.7, 122.1, 121.7, 121. 3, 117.0, 111.1, 111.0, 61.6, 55.9, 55.8, 55.7, 49.5, 14.1; IR (neat): ν

3333, 2926, 2859, 1776, 1721, 1595, 1522, 1463, 1425 cm^{-1}; HRMS (ESI): calc. for $C_{31}H_{28}N_3O_7$ ($M+H^+$): 554.1922; Found: 554.1927.

(2*S*,3*S*)-Ethyl 2-(2,3-dihydrobenzo[*b*][1,4]dioxin-6-yl)-3-(1,3-dioxoisoindolin-2-yl)-4-oxo-4-(quinolin-8-ylamino)butanoate (3q)

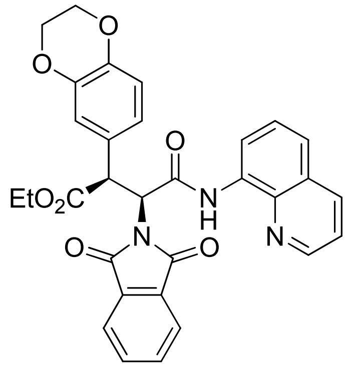

The title compound **3q** was prepared according to **GP3** and was purified by column chromatography (petroleum ether: dichloromethane: ethyl acetate = 7: 1: 2, R_f = 0.4). $[\alpha]^{20}_D$ = -150.6 (0.5 M in $CHCl_3$); **3q** was obtained as a white solid (60.0 mg, 73%). ^{1}H NMR (400 MHz, $CDCl_3$): δ 10.15 (s, 1H), 8.68 (dd, *J* = 6.7, 2.2 Hz, 1H), 8.51 (d, *J* = 3.1 Hz, 1H), 8.05 (d, *J* = 8.2 Hz, 1H), 7.75 (dd, *J* = 5.4, 3.1 Hz, 2H), 7.65 (dd, *J* = 5.4, 3.1 Hz, 2H), 7.50 – 7.43 (m, 2H), 7.31 (dd, *J* = 8.2, 4.2 Hz, 1H), 6.87 (d, *J* = 2.0 Hz, 1H), 6.79 (dd, *J* = 8.4, 2.1 Hz, 1H), 6.64 (d, *J* = 8.3 Hz, 1H), 5.94 (d, *J* = 11.5 Hz, 1H), 4.92 (d, *J* = 11.5 Hz, 1H), 4.28 (dq, *J* = 10.7, 7.1 Hz, 1H), 4.21 – 4.02 (m, 5H), 1.24 (t, *J* = 7.1 Hz, 3H); ^{13}C NMR (101 MHz, $CDCl_3$) δ 172.1, 167.5, 166.0, 148.4, 143.5, 143.4, 138.5, 136.2, 134.3, 133.8, 131.5, 127.9, 127.6, 127.3, 123.7, 122.1, 121.6, 117.6, 117.4, 117.0, 64.3, 64.2, 61.6, 55.8, 49.2, 14.1; IR (neat): ν 3335, 2984, 2856), 1774, 1723, 1650, 1531, 1427 cm^{-1}; HRMS (ESI): calc. for $C_{31}H_{26}N_3O_7$ ($M+H^+$): 552.1765; Found: 552.1776.

(2*S*,3*S*)-Methyl 3-(1,3-dioxoisoindolin-2-yl)-2-ethyl-4-oxo-4-(quinolin-8-ylamino)butanoate (3r)

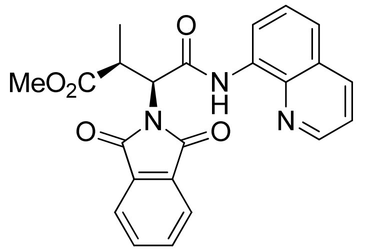

The title compound **3r** was prepared according to **GP3** with the addition of 20 mol% succinc anhydride and was purified by column chromatography (petroleum ether: dichloromethane: ethyl acetate = 7: 1: 2, R_f = 0.5). $[\alpha]^{20}_D$ = -32.6 (0.5 M in $CHCl_3$); **3r** was obtained as a colourless oil (31.0 mg, 50%). ^{1}H NMR (400 MHz, $CDCl_3$): δ 10.12 (s, 1H), 8.74 – 8.58 (m, 1H), 8.53 (dd, *J* = 4.2, 1.6 Hz, 1H), 8.07 (dd, *J* = 8.3, 1.6 Hz, 1H), 7.94 – 7.89 (m, 2H), 7.83 – 7.74 (m, 2H), 7.51 – 7.43 (m, 2H), 7.33 (dd, *J* = 8.3, 4.2 Hz, 1H), 3.95 – 3.85 (m, 1H), 3.81 (s, 3H), 1.18 (d, *J* = 7.2 Hz, 4H); ^{13}C NMR (101 MHz, $CDCl_3$) δ 174.0, 166.7, 165.0, 147.4, 137.4, 135.3, 133.7, 132.7, 130.6, 126.8, 126.3, 123.0, 121.1, 120.7, 116.0, 55.0, 51.5, 37.4, 14.1; IR (neat): ν 3335, 2926, 2855, 1774, 1721, 1651, 1536, 1459 cm^{-1}; HRMS (ESI): calc. for $C_{23}H_{20}N_3O_5$(M+H$^+$): 418.1397; Found: 418.1407.

(2*S*,3*S*)-Methyl 3-(1,3-dioxoisoindolin-2-yl)-2-ethyl-4-oxo-4-(quinolin-8-ylamino)butanoate (3s)

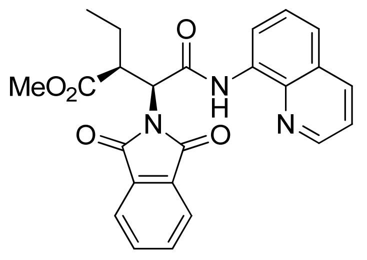

The title compound **3s** was prepared according to **GP3** with the addition of 20 mol% succinc anhydride and was purified by column chromatography (petroleum ether: dichloromethane: ethyl acetate = 7: 1: 2, R_f = 0.6). $[\alpha]^{20}_D$ = -44.0 (0.25 M in $CHCl_3$); **3s** was obtained as a colourless oil (29.0 mg, 45%). 1H NMR (400 MHz, $CDCl_3$) δ 10.13 (s, 1H), 8.64 (dd, *J* = 5.4, 3.6 Hz, 1H), 8.53 (dd, *J* = 4.2, 1.6 Hz, 1H), 8.07 (dd, *J* = 8.3, 1.6 Hz, 1H), 7.95 – 7.88 (m, 2H), 7.81 – 7.74 (m, 2H), 7.48 – 7.43 (m, 2H), 7.33 (dd, *J* = 8.3, 4.2 Hz, 1H), 5.56 (d, *J* = 11.2 Hz, 1H), 3.90 – 3.82 (m, 1H), 3.81 (s, 3H), 1.67 – 1.44 (m, 2H), 0.91 (t, *J* = 7.5 Hz, 3H); ^{13}C NMR (101 MHz, $CDCl_3$) δ 174.2, 167.8, 166.1, 148.4, 138.5, 136.3, 134.7, 133.8,

131.6, 127.9, 127.3, 124.0, 122.1, 121.7, 117.0, 55.1, 52.3, 44.6, 22.8, 10.7; IR (neat): ν 3334, 2927, 2858, 1775, 1721, 1692, 1530, 1485, 1428 cm^{-1}; HRMS (ESI): calc. for $C_{24}H_{22}N_3O_5(M+H^+)$: 432.1554; Found: 432.1559.

(2*S*,3*S*)-Methyl 3-(1,3-dioxoisoindolin-2-yl)-2-isopropyl-4-oxo-4-(quinolin-8-ylamino)butanoate (3t)

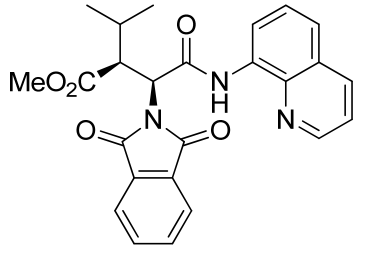

The title compound **3t** was prepared according to **GP3** with the addition of 20 mol% succinc anhydride at 140 °C and was purified by column chromatography (petroleum ether: dichloromethane: ethyl acetate = 7: 1: 2, R_f = 0.6). $[\alpha]^{20}_D$ = -53.0 (0.25 M in $CHCl_3$); **3t** was obtained as a colourless oil (33.6 mg, 50%). ^1H NMR (400 MHz, $CDCl_3$): δ 10.12 (s, 1H), 8.67 – 8.60 (m, 1H), 8.57 (dd, *J* = 4.2, 1.6 Hz, 1H), 8.08 (dd, *J* = 8.3, 1.6 Hz, 1H), 7.91 (td, *J* = 5.2, 2.1 Hz, 2H), 7.81 – 7.75 (m, 2H), 7.50 – 7.43 (m, 2H), 7.35 (dd, *J* = 8.3, 4.2 Hz, 1H), 5.65 (d, *J* = 11.7 Hz, 1H), 3.95 (dd, *J* = 11.7, 3.3 Hz, 1H), 3.80 (s, 3H), 1.83-1.73 (m, 1H), 1.07 (d, *J* = 6.9 Hz, 3H), 0.93 (d, *J* = 6.9 Hz, 3H); ^{13}C NMR (101 MHz, $CDCl_3$) δ 172.5, 168.4, 165. 8, 148.3, 138.6, 136.3, 134.5, 134.1, 131.7, 127.9, 127.4, 123.9, 122.0, 121.6, 117.0, 61.0, 51.5, 44.0, 38.3, 26.1, 26.1; IR (neat): ν 3334, 2925, 2859, 1774, 1725, 1650, 1532, 1460 cm^{-1}; HRMS (ESI): calc. for $C_{24}H_{22}N_3O_5(M+H^+)$: 446.1710; Found: 446.1715.

(*S*)-Methyl 4-(1,3-dioxoisoindolin-2-yl)-3,3-dimethyl-5-oxo-5-(quinolin-8-ylamino)pentanoate (3u)

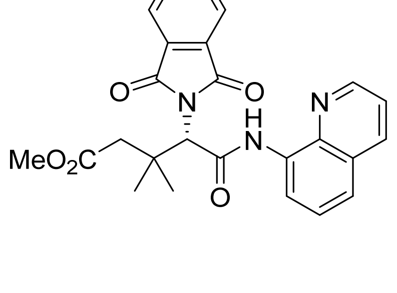

The title compound **3u** was prepared according to **GP3** with the addition of 20 mol% succinc anhydride at 140 °C and was purified by column chromatography (petroleum ether: tetrahydrofuran = 3: 1, R_f = 0.4). $[\alpha]^{20}_D$ = -15.5 (0.5 M in $CHCl_3$); **3u** was obtained as a pale while solid (20.0 mg, 30%). ^1H NMR (400 MHz, $CDCl_3$): δ 10.16 (s, 1H), 8.68 (dd, *J* = 7.1, 1.8 Hz, 1H), 8.50 (dd, *J* = 4.2, 1.6 Hz, 1H), 8.08 (dd, *J* = 8.3, 1.6 Hz, 1H), 7.92 – 7.87 (m, 2H), 7.83 – 7.73 (m, 2H), 7.54 – 7.42 (m, 2H), 7.33 (dd, *J* = 8.3, 4.2 Hz, 1H), 5.38 (s, 1H), 3.66 (s, 3H), 3.16 (d, *J* = 14.7 Hz, 1H), 2.66 (d, *J* = 14.7 Hz, 1H), 1.41 (s, 3H), 1.34 (s, 3H); ^{13}C NMR (101 MHz, $CDCl_3$) δ 172.5, 168.4, 165. 8, 148.3, 138.6, 136.3, 134.5, 134.1, 131.7, 127.9, 127.4, 123.9, 122.0, 121.6, 117.0, 61.0, 51.5, 44.0, 38.3, 26.1, 26.1; IR (neat): ν 3338, 2926, 2857, 1773, 1722, 1692, 1530, 1464 cm^{-1}; HRMS (ESI): calc. for $C_{24}H_{22}N_3O_5(M+H^+)$: 446.1710; Found: 446.1729.

(3*S*,4*R*)-Methyl 4-(1,3-dioxoisoindolin-2-yl)-3-ethyl-5-oxo-5-(quinolin-8-ylamino)pentanoate (3v)

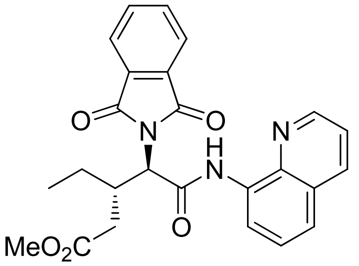

The title compound **3v** was prepared according to **GP3** with the addition of 20 mol% succinc anhydride at 140 °C and was purified by column chromatography (petroleum ether: tetrahydrofuran = 3: 1, R_f = 0.6). $[\alpha]^{20}_D$ = -15.0 (0.25 M in $CHCl_3$); **3v** was obtained as a pale while solid (23.1 mg, 30%). ^1H NMR (400 MHz, $CDCl_3$): δ 10.48 (s, 1H), 8.76 (dd, *J* = 4.2, 1.7 Hz, 1H), 8.75 – 8.69 (m, 1H), 8.12 (dd, *J* = 8.3, 1.7 Hz, 1H), 7.89 (dd, *J* = 5.5, 3.0 Hz, 2H), 7.74 (dd, *J* = 5.5, 3.0 Hz, 2H), 7.52 – 7.45 (m, 2H), 7.41 (dd, *J* = 8.3, 4.2 Hz, 1H), 5.23 (d, *J* = 9.8 Hz, 1H), 3.67 (s, 3H), 3.47 – 3.35 (m, 1H), 2.71 (dd, *J* = 16.2, 5.5 Hz, 1H), 2.64 (dd, *J* = 16.2, 6.5 Hz, 1H), 1.67 (m, 1H), 1.37 (tt, *J* = 14.8, 7.4 Hz, 1H), 0.95 (t, *J* = 7.5 Hz, 3H). ^{13}C NMR (101 MHz, $CDCl_3$) δ 172.7, 168.2, 166.6, 148.6, 138.7, 136.3, 134.5, 134.4, 134.2, 131.7, 128.0, 127.3, 123.8, 123.7, 122.2, 121.8, 117.1, 58.3, 51.9, 35.4, 34.7, 23.3, 10.5; IR (neat): ν 3330, 2923, 2857,

1773, 1720, 1650, 1531, 1459 cm^{-1}; HRMS (ESI): calc. for $C_{24}H_{22}N_3O_5(M+H^+)$: 446.1710; Found: 446.1713.

(3*S*,4*R*)-Methyl 4-(1,3-dioxoisoindolin-2-yl)-3-ethyl-5-oxo-5-(quinolin-8-ylamino)pentanoate (3w)

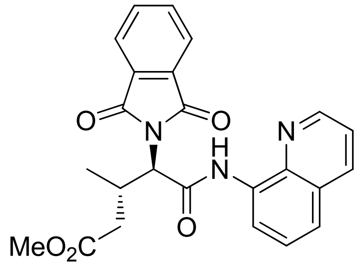

The title compound **3w** was prepared according to **GP3** with the addition of 20 mol% succinc anhydride at 140 °C and was purified by column chromatography (petroleum ether: tetrahydrofuran = 3: 1, R_f= 0.4). $[\alpha]^{20}_D$ = -13.0 (0.25 M in $CHCl_3$); **3w** was obtained as a pale while solid (20.0 mg, 31%). ^1H NMR (400 MHz, $CDCl_3$): δ 10.47 (s, 1H), 8.82 – 8.64 (m, 2H), 8.13 (dd, *J* = 8.3, 1.6 Hz, 1H), 7.95 – 7.87 (m, 2H), 7.83 – 7.72 (m, 2H), 7.50 (d, *J* = 4.2 Hz, 2H), 7.41 (dd, *J* = 8.3, 4.2 Hz, 1H), 5.06 (d, *J* = 9.9 Hz, 1H), 3.70 (s, 3H), 3.60 – 3.48 (m, 1H), 2.80 (dd, *J* = 15.8, 4.4 Hz, 1H), 2.48 (dd, *J* = 15.8, 8.3 Hz, 1H), 1.08 (d, *J* = 6.8 Hz, 3H); ^{13}C NMR (101 MHz, $CDCl_3$) δ 172.4, 168.1, 166.3, 148.6, 138.7, 136.3, 134.5, 134.1, 131.7, 128.0, 127.3, 123.9, 122.2, 121.8, 117.1, 59.6, 51.9, 38.7, 29.7, 16.9; IR (neat): ν 3335, 2925, 2858, 1771, 1719, 1692, 1531, 1462 cm^{-1}; HRMS (ESI): calc. for $C_{24}H_{22}N_3O_5(M+H^+)$: 432.1554; Found: 432.1554.

(2*S*,3*S*)-Methyl 3-(1,3-dioxoisoindolin-2-yl)-4-oxo-2-phenyl-4-(quinolin-8-ylamino)butanoate (4b)

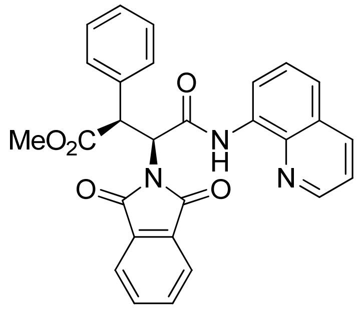

The title compound **4b** was prepared according to **GP3** and was purified by column chromatography (petroleum ether: dichloromethane: ethyl acetate = 7: 1: 2, R*f*=0.6). **4b** was obtained as a yellow solid (51.8 mg, 72%).

(2*S*,3*S*)-Propyl 3-(1,3-dioxoisoindolin-2-yl)-4-oxo-2-phenyl-4-(quinolin-8-ylamino)butanoate (4c)

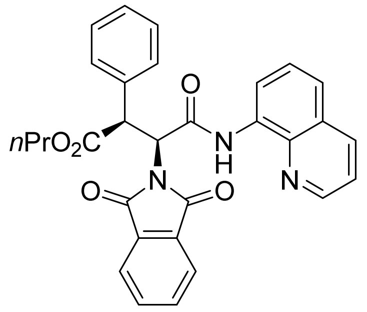

The title compound **4c** was prepared according to **GP3** and was purified by column chromatography (petroleum ether: dichloromethane: ethyl acetate = 7: 1: 2, R*f* = 0.6). $[\alpha]^{20}_{D}$ = -136.0(1.0 M in $CHCl_3$); **4c** was obtained as a white solid (54.9 mg, 72%). ^{1}H NMR (400 MHz, $CDCl_3$): δ 10.17 (s, 1H), 8.70 (dd, *J* = 6.8, 1.7 Hz, 1H), 8.56 – 8.47 (m, 1H), 8.06 (d, *J* = 8.3 Hz, 1H), 7.72 (dd, *J* = 5.4, 2.9 Hz, 2H), 7.63 (dd, *J* = 5.3, 3.1 Hz, 2H), 7.50 – 7.42 (m, 2H), 7.38-7.28 (m, 3H), 7.16 (t, *J* = 7.4 Hz, 2H), 7.10 (t, *J* = 7.2 Hz, 1H), 6.04 (d, *J* = 11.5 Hz, 1H), 5.06 (d, *J* = 11.5 Hz, 1H), 4.22 – 4.04 (m, 2H), 1.71 – 1.54 (m, 2H), 0.81 (t, *J* = 7.4 Hz, 3H); ^{13}C NMR (101 MHz, $CDCl_3$) δ 172.1, 167.5, 166.0, 148.4, 138.5, 136.3, 134.6, 134.3, 133.9, 131.3, 128.8, 128.7, 128.1, 127. 9, 127.4, 123.7, 122.1, 121. 7, 117.1, 67.2, 55.7, 50.0, 22.0, 10.3; IR (neat): ν 3334, 2923, 2858, 1775, 1725, 1650, 1531, 1459 cm^{-1}; HRMS (ESI): calc. for $C_{30}H_{26}N_3O_5$(M+H$^+$): 508.1867; Found: 508.1867.

(2*S*,3*S*)-Hexadecyl 3-(1,3-dioxoisoindolin-2-yl)-4-oxo-2-phenyl-4-(quinolin-8-ylamino)butanoate (4d)

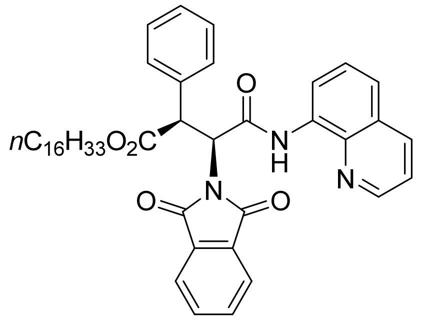

The title compound **4d** was prepared according to **GP3** and was purified by column chromatography (petroleum ether: dichloromethane: ethyl acetate = 8: 1: 1, R_f = 0.6). $[\alpha]^{20}_D$ = -111.7 (1.0 M in $CHCl_3$); **4d** was obtained as a colourless oil (74.1mg, 73%). ^{1}H NMR (400 MHz, $CDCl_3$): δ 10.18 (s, 1H), 8.71 (d, *J* = 5.9 Hz, 1H), 8.54 (s, 1H), 8.07 (d, *J* = 7.4 Hz, 1H), 7.72 (s, 2H), 7.63 (s, 2H), 7.55 – 7.44 (m, 2H), 7.38 – 7.30 (m, 3H), 7.17 (t, *J* = 7.3 Hz, 2H), 7.11 (d, *J* = 7.1 Hz, 1H), 6.04 (d, *J* = 11.5 Hz, 1H), 5.07 (d, *J* = 11.4 Hz, 1H), 4.47 – 3.98 (m, 2H), 1.79 – 1.44 (m, 2H), 1.34 – 1.18 (m, 26H), 0.87 (t, *J* = 6.6 Hz, 3H); ^{13}C NMR (101 MHz, $CDCl_3$) δ 172.0, 167.4, 165.9, 148.4, 138.4, 136.3, 134.6, 134.3, 133.8, 131.3, 128.7, 128.7, 128.1, 127.9, 127.3, 123.6, 122.1, 121.7, 117.1, 65.7, 55.7, 50.0, 32.0, 29.8, 29.8, 29.7, 29.6, 29.5, 29.5, 29.5, 29.2, 28.5, 25.7, 22.8, 14.2; IR (neat): ν 3334, 2924, 2859, 1774, 1725, 1649, 1531, 1458 cm^{-1}; HRMS (ESI): calc. for $C_{43}H_{52}N_3O_5$(M+H$^+$): 690.3901; Found: 690.3919.

(2*S*,3*S*)-3-Chloropropyl 3-(1,3-dioxoisoindolin-2-yl)-4-oxo-2-phenyl-4-(quinolin-8-ylamino)butanoate (4e)

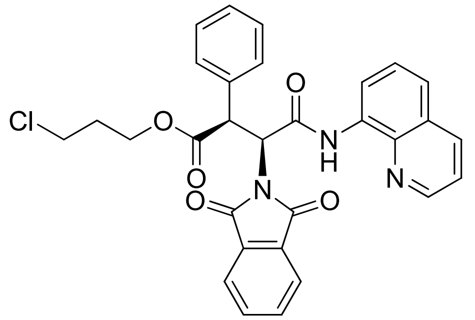

The title compound **4e** was prepared according to **GP3** and was purified by column chromatography (petroleum ether : ethyl acetate = 2 : 1, R_f = 0.5). $[\alpha]^{20}_D$ = -143.8 (0.5 M in $CHCl_3$); **4e** was obtained as a colourless oil (56.9 mg, 70%). ^{1}H NMR (400 MHz, $CDCl_3$): ^{1}H NMR (400 MHz, $CDCl_3$): δ 10.17 (s, 1H), 8.69 (dd, *J* = 6.9, 2.0 Hz, 1H), 8.51 (dd, *J* = 4.2, 1.5 Hz, 1H), 8.07 (dd, *J* = 8.3, 1.5 Hz, 1H), 7.77 –

7.69 (m, 2H), 7.68 – 7.62 (m, 2H), 7.54 – 7.43 (m, 2H), 7.36 – 7.30 (m, 3H), 7.21 – 7.16 (m, 2H), 7.14 – 7.08 (m, 1H), 6.03 (d, *J* = 11.4 Hz, 1H), 5.04 (d, *J* = 11.5 Hz, 1H), 4.47 – 4.35 (m, 1H), 4.32 – 4.25 (m, 1H), 3.63 – 3.36 (m, 2H), 2.41 – 1.93 (m, 2H); ^{13}C NMR (101 MHz, $CDCl_3$) δ 172.0, 167.4, 166.0, 148.4, 138.4, 136.3, 134.4, 134.3, 133.7, 131.2, 128.9, 128.6, 128.3, 127.9, 127.3, 123.7, 122.2, 121.7, 117.0, 62.2, 55.6, 49.9, 41.3, 31.7; IR (neat): ν 3334, 2927, 2858, 1775, 1723, 1655, 1530, 1486, 1426 cm^{-1}; HRMS (ESI): calc. for $C_{30}H_{25}ClN_3O_5$(M+H$^+$): 542.1477; Found: 542.1477.

(2*S*,3*S*)-*iso*-Propyl 3-(1,3-dioxoisoindolin-2-yl)-4-oxo-2-phenyl-4-(quinolin-8-ylamino)butanoate (4f)

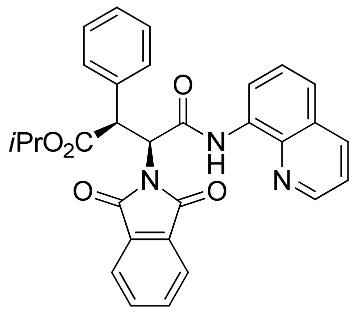

The title compound **4f** was prepared according to **GP3** and was purified by column chromatography (petroleum ether: dichloromethane: ethyl acetate = 7: 1: 2, R_f = 0.5). $[\alpha]^{20}_D$ = -150.3 (1.0 M in $CHCl_3$); **4f** was obtained as a colourless oil (72.7 mg, 96%). ^{1}H NMR (400 MHz, $CDCl_3$): δ 10.17 (s, 1H), 8.71 (d, *J* = 7.0 Hz, 1H), 8.53 (dd, *J* = 3.4, 2.1 Hz, 1H), 8.16 – 7.98 (m, 1H), 7.75-7.66 (m, 2H), 7.66 – 7.56 (m, 2H), 7.55 – 7.41 (m, 2H), 7.39 – 7.27 (m, 3H), 7.16 (t, *J* = 7.3 Hz, 2H), 7.13-7.06 (m, 1H), 6.01 (d, *J* = 11.5 Hz, 1H), 5.16 - 5.04 (m, 1H), 5.01 (t, *J* = 11.5 Hz, 1H), 1.30 (d, *J* = 6.3 Hz, 3H), 1.09 (d, *J* = 6.2 Hz, 3H); ^{13}C NMR (101 MHz, $CDCl_3$) δ 172.1, 167.5, 166.0, 148.4, 138.5, 136.3, 134.6, 134.3, 133.9, 131.3, 128.8, 128.7, 128.1, 127.9, 127.4, 123.7, 122.1, 121. 7, 117.1, 67.2, 55.7, 50.0, 22.0, 10.3; IR (neat): ν 3333, 2926, 2858, 1775, 1723, 1653, 1530, 1486, 1461, 1426 cm^{-1}; HRMS (ESI): calc. for $C_{30}H_{26}N_3O_5$(M+H$^+$): 508.1867; Found: 508.1879.

(2*S*,3*S*)-*iso*-Butyl 3-(1,3-dioxoisoindolin-2-yl)-4-oxo-2-phenyl-4-(quinolin-8-ylamino)butanoate (4g)

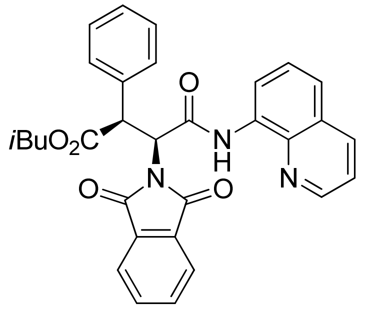

The title compound **4g** was prepared according to **GP3** and was purified by column chromatography (petroleum ether: dichloromethane: ethyl acetate = 7: 1: 2, R_f= 0.6). $[\alpha]^{20}_{D}$ = -126.3 (0.5 M in $CHCl_3$); **4g** was obtained as a colourless oil (63.7 mg, 82%). ^{1}H NMR (400 MHz, $CDCl_3$): δ 10.18 (s, 1H), 8.70 (dd, J = 6.8, 1.8 Hz, 1H), 8.58 – 8.46 (m, 1H), 8.06 (d, J = 8.2 Hz, 1H), 7.72 (dd, J = 5.4, 3.0 Hz, 2H), 7.62 (dd, J = 5.4, 3.1 Hz, 2H), 7.52-7.43 (m, 2H), 7.40 – 7.28 (m, 3H), 7.17 (t, J = 7.4 Hz, 2H), 7.13-7.06 (m, 1H), 6.05 (d, J = 11.5 Hz, 1H), 5.09 (d, J = 11.5 Hz, 1H), 3.96 (d, J = 6.7 Hz, 2H), 1.99 – 1.83 (m, 1H), 0.90-0.76 (m, 6H). ^{13}C NMR (101 MHz, $CDCl_3$) δ 172.0, 167.4, 165.9, 148.4, 138.5, 136.25, 134.7, 134.3, 133.9, 131.3, 128.7, 128.7, 128.1, 127.9, 127.3, 123.6, 122.0, 121.6, 117.0, 71.5, 55.6, 50.0, 27. 8, 19.0, 18.9; IR (neat): ν 3331, 2924, 2858, 1775, 1724, 1651, 1532, 1488, 1460 cm^{-1}; HRMS (ESI): calc. for $C_{31}H_{28}N_3O_5$(M+H$^+$): 522.2023; Found: 522.2043.

(2*S*,3*S*)-Cyclopentyl 3-(1,3-dioxoisoindolin-2-yl)-4-oxo-2-phenyl-4-(quinolin-8-ylamino)butanoate (4h)

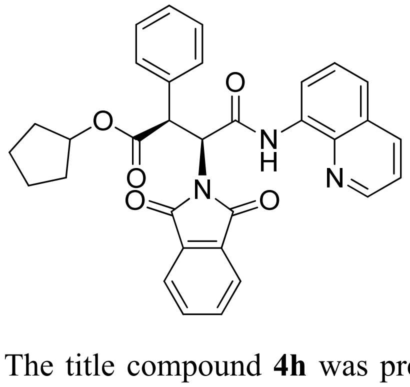

The title compound **4h** was prepared according to **GP3** and was purified by column chromatography (petroleum ether: dichloromethane: ethyl acetate = 7: 1: 2, R_f= 0.6). $[\alpha]^{20}_{D}$ = -86.8 (0.5 M in $CHCl_3$); **4h** was obtained as a colourless oil (24.9 mg, 39%). ^{1}H NMR (400 MHz, $CDCl_3$): δ 10.18 (s, 1H), 8.71 (dd, J = 6.9, 1.8 Hz, 1H), 8.53 (d, J = 3.0 Hz, 1H), 8.06 (d, J = 7.5 Hz, 1H), 7.71 (dt, J = 6.9, 3.5 Hz, 2H), 7.65

– 7.56 (m, 2H), 7.55 – 7.39 (m, 2H), 7.32 (t, *J* = 6.3 Hz, 3H), 7.16 (t, *J* = 7.3 Hz, 2H), 7.13 – 7.05 (m, 1H), 6.01 (d, *J* = 11.5 Hz, 1H), 5.30 – 5.15 (m, 1H), 5.02 (d, *J* = 11.5 Hz, 1H), 1.91 – 1.79 (m, 2H), 1.78-1.62 (m, 2H), 1.58 – 1.39 (m, 4H); ^{13}C NMR (101 MHz, $CDCl_3$) δ 171.6, 167.4, 165.9, 148.4, 138.4, 136.3, 134.7, 134.3, 133.8, 131.3, 128.7, 128.6, 128.0, 127.8, 127.3, 123.6, 122.0, 121.6, 117.0, 78.4, 55.7, 50.1, 32.4, 23.8, 23.7; IR (neat): ν 3333, 2922, 2853, 1776, 1723, 1653, 1529, 1464, 1426 cm^{-1}; HRMS (ESI): calc. for $C_{32}H_{28}N_3O_5$(M+H$^+$): 534.2023; Found: 534.2033.

(2*S*,3*S*)-(1*R*,2*S*,4*S*)-2-*iso*-Propyl-4-methylcyclohexyl 3-(1,3-dioxoisoindolin-2-yl)-4-oxo-2-phenyl-4-(quinolin-8-ylamino)butanoate (4i)

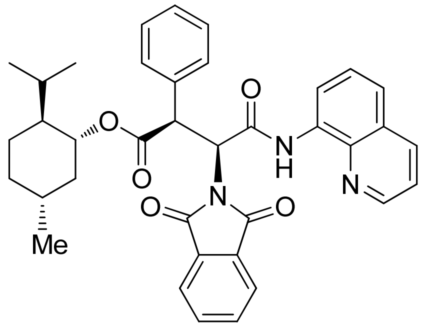

The title compound **4i** was prepared according to **GP3** purified by column chromatography (petroleum ether: ethyl acetate = 3: 1, R*f*= 0.5). $[\alpha]^{20}_D$ = -79.5 (1.0 M in $CHCl_3$); **4i** was obtained as a colourless oil (65.2 mg, 72%). ^{1}H NMR (400 MHz, $CDCl_3$): δ 10.22 (s, 1H), 8.72 (d, *J* = 7.1 Hz, 1H), 8.59 (d, *J* = 4.1 Hz, 1H), 8.06 (d, *J* = 8.2 Hz, 1H), 7.75 – 7.66 (m, 2H), 7.64 – 7.58 (m, 2H), 7.52 – 7.42 (m, 2H), 7.39 – 7.29 (m, 3H), 7.17 (t, *J* = 7.4 Hz, 2H), 7.10 (t, *J* = 7.2 Hz, 1H), 6.00 (d, *J* = 11.6 Hz, 1H), 5.10 (d, *J* = 11.6 Hz, 1H), 4.76 (td, *J* = 10.9, 4.2 Hz, 1H), 2.18 – 2.08 (m, 1H), 1.74 (d, *J* = 12.0 Hz, 1H), 1.65 – 1.58 (m, 2H), 1.44 – 1.35 (m, 2H), 1.06 – 0.91 (m, 1H), 0.85 (d, *J* = 7.0 Hz, 3H), 0.81 – 0.68 (m, 4H), 0.65 (d, *J* = 6.8 Hz, 3H); ^{13}C NMR (101 MHz, $CDCl_3$) δ 171.5, 167.5, 165.7, 148.4, 138.5, 136.2, 134.7, 134.2, 134.0, 131.3, 128.7, 128.6, 128.0, 127.8, 127.3, 123.6, 121.9, 121.6, 116.9, 75.4, 56.0, 50.2, 47.0, 40.1, 34.3, 31.4, 25.8, 23. 2, 22.0, 20.9, 16.0; IR (neat): ν 3333, 2924, 2863, 1776, 1723, 1653, 1529, 1486, 1459, 1426 cm^{-1}; HRMS (ESI): calc. for $C_{37}H_{38}N_3O_5$(M+H$^+$): 604.2806; Found: 604.2806.

(2*S*,3*S*)-(9*H*-Fluoren-9-yl)methyl 3-(1,3-dioxoisoindolin-2-yl)-4-oxo-2-phenyl-4-(quinolin-8-ylamino)butanoate (4j)

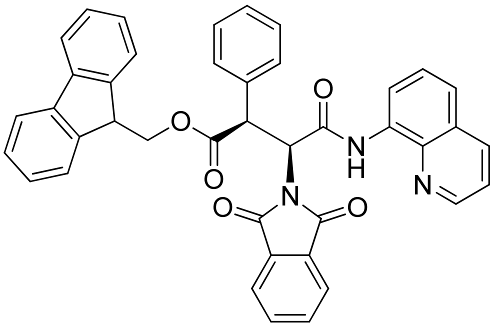

The title compound **4j** was prepared according to **GP3** and purified by column chromatography (petroleum ether: dichloromethane: ethyl acetate = 6: 1: 3, R_f = 0.6). $[\alpha]^{20}_D$ = -92.4 (1.0 M in $CHCl_3$); **4j** was obtained as colourless oil (68.8 mg, 71%). ^1H NMR (400 MHz, $CDCl_3$): δ 10.19 (s, 1H), 8.73 (dd, *J* = 6.8, 2.0 Hz, 1H), 8.53 (dd, *J* = 4.2, 1.5 Hz, 1H), 8.08 (d, *J* = 8.2 Hz, 1H), 7.78 – 7.73 (m, 2H), 7.70 (t, *J* = 7.6 Hz, 2H), 7.67-7.62 (m, 2H), 7.56 – 7.46 (m, 2H), 7.39-7.27 (m, 7H), 7.25 – 7.08 (m, 5H), 6.06 (d, *J* = 11.4 Hz, 1H), 5.23 (d, *J* = 11.4 Hz, 1H), 4.50 (dd, *J* = 10.6, 6.8 Hz, 1H), 4.46 – 4.38 (m, 1H), 4.25 (t, *J* = 7.3 Hz, 1H). ^{13}C NMR (101 MHz, $CDCl_3$) δ 172.0, 167.4, 165.9, 148.4, 144.1, 143.5, 141.3, 141. 2, 138. 5, 136.3, 134.4, 134.2, 133.8, 131.3, 129.0, 128.9, 128.3, 127.9, 127.7, 127.6, 127.3, 127.1, 125.4, 125.1, 123.7, 122.1, 121.7, 119.9, 119.9, 117.1, 67.7, 55. 5, 50.0, 46.6; IR (neat): ν 3334, 2922, 2857, 1775, 1724, 1656, 1531, 1457 cm^{-1}; HRMS (ESI): calc. for $C_{41}H_{30}N_3O_5$(M+H$^+$): 644.2180; Found: 644.2184.

2.4. General Procedure (GP4) for Alkoxycarbonylation of Methyl C(sp^3)–H Bonds

To a 50 mL Schlenk tube, were added **1** (0.15 mmol), $Pd(OTFA)_2$ (5.0 mg, 0.015 mmol), Ag_2CO_3 (82.7 mg, 0.3 mmol), Na_3PO_4 (49.0 mg, 0.3 mmol), $ClCO_2Me$ (0.45 mmol, 3.0 equiv.) and toluene (2.0 mL). The tube was sealed under air. The mixture was stirred at room temperature for 5 minutes then heated at 120 °C for 20 h. After cooling to room temperature, the reaction mixture was diluted with EtOAc (10 mL) and filtered through a pad of Celite. After concentration in vacuo, the crude reaction mixture was purified by silica gel flash chromatography.

(*R*)-methyl 3-(1,3-dioxoisoindolin-2-yl)-4-oxo-4-(quinolin-8-ylamino)butanoate (6a)

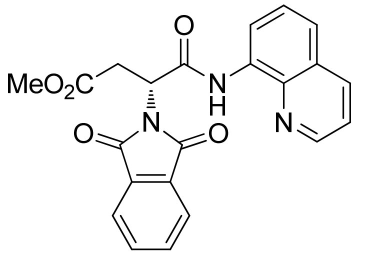

The title compound **6a** was prepared according to **GP4** in the absence of sodium phosphate and purified by column chromatography (petroleum ether: dichloromethane: ethyl acetate = 7: 1: 2), R_f = 0.5. $[\alpha]^{20}_D$ = + 11.5 (1.0 M in $CHCl_3$); **6a** was obtained as a pale yellow solid (43.0 mg, 71%). ^{1}H NMR (400 MHz, $CDCl_3$) δ 10.28 (s, 1H), 8.71 – 8.60 (m, 1H), 8.57 – 8.46 (m, 1H), 8.06 (d, *J* = 8.3 Hz, 1H), 7.93 – 7.81 (m, 2H), 7.78 – 7.70 (m, 2H), 7.50 – 7.42 (m, 2H), 7.33 (dd, *J* = 8.0, 4.0 Hz, 1H), 5.64 (dd, *J* = 8.1, 6.6 Hz, 1H), 3.71 (s, 3H), 3.65 (dd, *J* = 17.1, 6.6 Hz, 1H), 3.33 (dd, *J* = 17.0, 8.2 Hz, 1H). ^{13}C NMR (101 MHz, $CDCl_3$) δ 171.2, 167.7, 165.7, 148.4, 138.4, 136.3, 134.5, 133.7, 131.7, 127.8, 127.2, 123.8, 122.2, 121.7, 116. 8, 52.3, 50.8, 33.5; IR (neat): ν 3330, 2874, 2748, 1775, 1723, 1648, 1530, 1476, 1451 cm^{-1};HRMS (ESI): calc. for $C_{22}H_{17}N_3O_5Na$(M + Na^+): 426.1060; Found: 426.1071.

Methyl 3-(quinolin-8-ylcarbamoyl)pentanoate (6b)

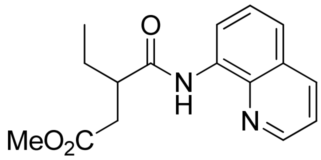

The title compound **6b** was prepared according to **GP3** and was purified by column chromatography (petroleum ether : ethyl acetate = 6: 1, R_f = 0.5). **6b** was obtained as a white solid (24.0 mg, 56%). ^{1}H NMR (400 MHz, $CDCl_3$) δ 9.99 (s, 1H), 8.83 (dd, *J* = 4.2, 1.6 Hz, 1H), 8.78 (dd, *J* = 6.9, 2.0 Hz, 1H), 8.15 (dd, *J* = 8.3, 1.5 Hz, 1H), 7.56 – 7.48 (m, 2H), 7.45 (dd, *J* = 8.3, 4.2 Hz, 1H), 3.66 (s, 3H), 3.03 – 2.86 (m, 2H), 2.63 – 2.45 (m, 1H), 1.92 – 1.81 (m, 2H), 1.76 – 1.61 (m, 1H), 1.04 (t, *J* = 7.4 Hz, 3H). ^{13}C NMR (101 MHz, $CDCl_3$) δ 173.4, 173.0, 148.4, 138.6, 136.4, 134.6, 128.1, 127.5, 121.3, 121.7, 116.7,

52.0, 46.0, 36.4, 26.2, 11.9; IR (neat): ν 3354, 2966, 2878, 1737, 1687, 1528, 1485, 1429 cm^{-1}; HRMS (ESI): calc. for $C_{16}H_{18}N_2O_3$ (M^+): 286.1312; Found: 286.1321.

Methyl 3-(quinolin-8-ylcarbamoyl)hexanoate (6c)

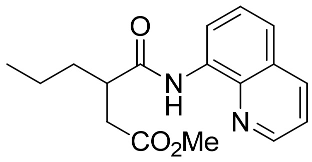

The title compound **6c** was prepared according to **GP3** and was purified by column chromatography (petroleum ether : ethyl acetate = 6 : 1, R_f = 0.6). **6c** was obtained as a white solid (20.2 mg, 45%). ^1H NMR (400 MHz, $CDCl_3$) δ 10.00 (s, 1H), 8.82 (dd, J = 4.2, 1.7 Hz, 1H), 8.78 (dd, J = 7.0, 2.0 Hz, 1H), 8.14 (dd, J = 8.3, 1.6 Hz, 1H), 7.51 (m, 2H), 7.44 (dd, J = 8.3, 4.2 Hz, 1H), 3.66 (s, 3H), 3.04 (m, 1H), 2.93 (dd, J = 16.5, 9.2 Hz, 1H), 2.55 (dd, J = 16.6, 4.8 Hz, 1H), 1.81 (m, 1H), 1.58 (m, 1H), 1.44 (m, 2H), 0.95 (t, J = 7.3 Hz, 3H); ^{13}C NMR (101 MHz, $CDCl_3$) δ 173.5, 172.9, 148.3, 138.5, 136.4, 134.5, 128.0, 127.4, 121.7, 121.6, 116.6, 51.9, 44.4, 36.7, 35.2, 20.6, 14.1; IR (neat): ν 3353, 2957, 2870, 1736, 1686, 1526, 1484, 1429 cm^{-1}; HRMS (ESI): calc. for $C_{17}H_{20}N_2O_3$ (M^+): 300.1474; Found: 300.1468.

Methyl 3-(quinolin-8-ylcarbamoyl)nonanoate (6d)

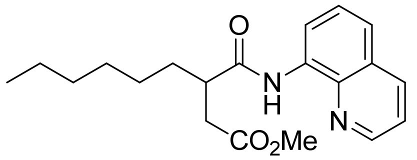

The title compound **6d** was prepared according to **GP3** and was purified by column chromatography (petroleum ether : ethyl acetate = 7: 1, R_f = 0.6). **6d** was obtained as a colourless oil (31.3 mg, 61%). ^1H NMR (400 MHz, $CDCl_3$) δ 9.98 (s, 1H), 8.82 (dd, J = 4.2, 1.6 Hz, 1H), 8.77 (dd, J = 6.9, 2.1 Hz, 1H), 8.15 (dd, J = 8.3, 1.6 Hz, 1H), 7.55 – 7.48 (m, 2H), 7.45 (dd, J = 8.3, 4.2 Hz, 1H), 3.65 (s, 3H), 3.09 – 2.96 (m, 1H), 2.92 (dd, J = 16.5, 9.1 Hz, 1H), 2.54 (dd, J = 16.5, 4.8 Hz, 1H), 1.91 – 1.78 (m, 1H), 1.67 – 1.53 (m, 1H), 1.48 – 1.39 (m, 2H), 1.35 – 1.21 (m, 6H), 0.84 (t, J = 6.9 Hz, 3H). ^{13}C NMR (101 MHz, $CDCl_3$) δ 173.5, 173.0, 148.4, 138.6, 136.4, 134.6, 128.1, 127.5, 121.7, 121.7, 116.7, 52.0, 44.6, 36.8,

33.1, 31.8, 29.4, 27.3, 22.7, 14.2; IR (neat): ν 3355, 2930, 2859, 1738, 1688, 1527, 1485, 1429 cm^{-1}; HRMS (ESI): calc. for $C_{20}H_{26}N_2O_3$ (M^+): 342.1938; Found: 342.1939.

Methyl 5-phenyl-3-(quinolin-8-ylcarbamoyl)pentanoate (6e)

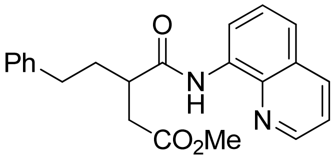

The title compound **6e** was prepared according to **GP3** and was purified by column chromatography (petroleum ether : ethyl acetate = 6: 1, R_f= 0.6). **6e** was obtained as white solid (26.0 mg, 48%). ^{1}H NMR (400 MHz, $CDCl_3$) δ 10.02 (s, 1H), 8.84 (dd, J = 4.2, 1.7 Hz, 1H), 8.80 (dd, J = 6.8, 2.2 Hz, 1H), 8.16 (dd, J = 8.3, 1.6 Hz, 1H), 7.58 – 7.49 (m, 2H), 7.48 – 7.42 (m, 1H), 7.30 – 7.24 (m, 2H), 7.24 – 7.15 (m, 3H), 3.65 (s, 3H), 3.09 – 3.01 (m, 1H), 2.96 (dd, J = 16.3, 9.1 Hz, 1H), 2.84 – 2.65 (m, 2H), 2.57 (dd, J = 16.3, 4.7 Hz, 1H), 2.25 – 2.12 (m, 1H), 2.00 – 1.84 (m, 1H); ^{13}C NMR (101 MHz, $CDCl_3$) δ 173.1, 172.7, 148.4, 141.3, 138.6, 136.4, 134.5, 128.6, 128.6, 128.1, 127.5, 126.2, 121.8, 121.8, 116.7, 52.0, 44.0, 37.0, 34.6, 33.5; IR (neat): ν 3353, 2948, 2870, 1736, 1686, 1526, 1484, 1429 cm^{-1}; HRMS (ESI): calc. for $C_{22}H_{22}N_2O_3$ (M^+):362.1625; Found: 362.1626.

Methyl 3-cyclohexyl-4-oxo-4-(quinolin-8-ylamino)butanoate (6f)

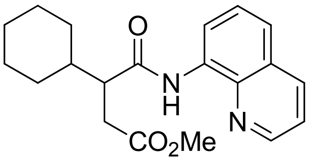

The title compound **6f** was prepared according to **GP3** and was purified by column chromatography (petroleum ether : ethyl acetate = 6: 1, R_f = 0.5). **6f** was obtained as a colourless oil (31.0 mg, 61%). ^{1}H NMR (400 MHz, $CDCl_3$) δ 9.94 (s, 1H), 8.83 (dd, J = 4.2, 1.6 Hz, 1H), 8.78 (dd, J = 6.9, 2.1 Hz, 1H), 8.15 (dd, J = 8.3, 1.5 Hz, 1H), 7.57 – 7.48 (m, 2H), 7.45 (dd, J = 8.3, 4.2 Hz, 1H), 3.63 (s, 3H), 2.94 (dd, J = 16.5, 10.2 Hz, 1H), 2.87 – 2.77 (m, 1H), 2.61 (dd, J = 16.5, 3.6 Hz, 1H), 1.93 – 1.55 (m, 6H), 1.34 – 0.97 (m, 5H); ^{13}C NMR (101 MHz, $CDCl_3$) δ 173.4, 173.1, 148.4, 138.6, 136.4, 134.54, 128.1, 127.5,

121.7, 121.6, 116.5, 51.9, 50.5, 40.6, 34.0, 31.0, 30.6, 26.5, 26.4, 26.3; IR (neat): ν 3355, 2926, 2852, 1735, 1683, 1524, 1483, 1428 cm^{-1}; HRMS (ESI): calc. for $C_{20}H_{24}N_2O_3$ (M^+): 340.1781; Found: 340.1786.

1-ethyl 4-methyl 2-(quinolin-8-ylcarbamoyl)succinate (6g)

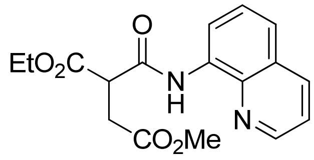

The title compound **6g** was prepared according to **GP3** and was purified by column chromatography (petroleum ether : ethyl acetate = 6: 1, R_f = 0.5). **6g** was obtained as a colourless oil (31.0 mg, 40%). ^1H NMR (400 MHz, $CDCl_3$) δ 10.67 (s, 1H), 8.85 (dd, *J* = 4.2, 1.6 Hz, 1H), 8.71 (dd, *J* = 5.1, 3.8 Hz, 1H), 8.17 (dd, *J* = 8.3, 1.6 Hz, 1H), 7.55 – 7.49 (m, 2H), 7.47 (dd, *J* = 8.3, 4.2 Hz, 1H), 4.31 (q, *J* = 7.1 Hz, 2H), 4.15 (t, *J* = 7.1 Hz, 1H), 3.73 (s, 3H), 3.14 (dd, *J* = 7.1, 2.7 Hz, 2H), 1.36 (t, *J* = 7.1 Hz, 3H); ^{13}C NMR (101 MHz, $CDCl_3$) δ 172.2, 169.2, 164.9, 148.5, 138.7, 136.5, 134.3, 128.1, 127.4, 122.2, 121.9, 116.9, 62.5, 52.2, 49.9, 32.5, 14.1; IR (neat): ν 3358, 2988, 2865, 1739, 1691, 1533, 1486, 1433 cm^{-1}; HRMS (ESI): calc. for $C_{17}H_{18}N_2O_5$(M^+): 330.1210; Found: 330.1219.

Methyl 8-(benzyloxy)-3-(quinolin-8-ylcarbamoyl)octanoate (6h)

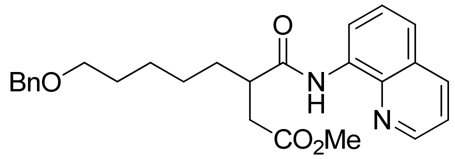

The title compound **6h** was prepared according to **GP3** and was purified by column chromatography (petroleum ether : ethyl acetate = 3 : 1, R_f= 0.5). **6h** was obtained as a colourless oil (33.8 mg, 52%). ^1H NMR (400 MHz, $CDCl_3$) δ 10.00 (s, 1H), 8.81 (dd, *J* = 4.2, 1.6 Hz, 1H), 8.77 (dd, *J* = 6.6, 2.3 Hz, 1H), 8.16 (dd, *J* = 8.3, 1.5 Hz, 1H), 7.57 – 7.48 (m, 2H), 7.45 (dd, *J* = 8.3, 4.2 Hz, 1H), 7.35 – 7.20 (m, 5H), 4.45 (s, 2H), 3.66 (s, 3H), 3.44 (t, *J* = 6.4 Hz, 2H), 3.10 – 2.98 (m, 1H), 2.92 (dd, *J* = 16.6, 9.2 Hz, 1H), 2.55 (dd, *J* = 16.6, 4.8 Hz, 1H), 1.93 – 1.79 (m, 1H), 1.75 – 1.58 (m, 5H), 1.57 – 1.44 (m, 2H); ^{13}C ^{13}C NMR (101 MHz, $CDCl_3$) δ 173.4, 172.8, 148.4, 138.6, 138.6, 136.4, 134.5, 128.5, 128.1, 127.8, 127.6,

127.5, 121.7, 121.7, 116.7, 73.0, 70.1, 52.0, 44.6, 36.7, 32.9, 29.8, 24.1; IR (neat): ν 3358, 2923, 2853, 1738, 1688, 1528, 1486, 1427 cm^{-1}; HRMS (ESI): calc. for $C_{26}H_{30}N_2O_4(M^+)$: 434.2200; Found: 434.2207.

(6*S*)-methyl 6,10-dimethyl-3-(quinolin-8-ylcarbamoyl)undec-9-enoate (6i)

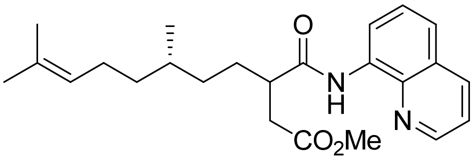

The title compound **6i** was prepared according to **GP3** and was purified by column chromatography (petroleum ether : ethyl acetate = 7: 1, R_f= 0.5). **6i** was obtained as a pale while solid (36.1 mg, 61%). ^{1}H NMR (400 MHz, $CDCl_3$) δ 9.99 (s, 1H), 8.86 – 8.80 (m, 1H), 8.78 (dt, *J* = 6.9, 1.7 Hz, 1H), 7.55 – 7.48 (m, 2H), 7.45 (dd, *J* = 8.3, 4.2 Hz, 1H), 5.05 (t, *J* = 6.4 Hz, 1H), 3.66 (s, 3H), 3.06 – 2.86 (m, 2H), 2.56 (dd, *J* = 15.8, 3.9 Hz, 1H), 2.01 – 1.71 (m, 3H), 1.64 (d, *J* = 5.5 Hz, 3H), 1.54 (d, *J* = 7.1 Hz, 3H), 1.50 – 1.39 (m, 2H), 1.38 – 1.21 (m, 2H), 1.18 – 1.06 (m, 1H), 0.87 (d, *J* = 6.4 Hz, 3H); ^{13}C NMR (101 MHz, $CDCl_3$) δ 173.5, 173.5, 172.9, 172.9, 148.4, 138.6, 136.4, 134.6, 131.3, 131.3, 128.1, 127.5, 124.9, 121.2, 121.7, 116.7, 52.0, 44.8, 44.8, 37.0, 36.9, 36.9, 36.7, 34.5, 34.4, 32.5, 30.5, 25.8, 25.6, 25.6, 19.5, 19.5, 17.7; IR (neat): ν 3355, 2925, 2851, 1738, 1688, 1527, 1485, 1458, 1430 cm^{-1}; HRMS (ESI): calc. for $C_{24}H_{32}N_2O_3(M^+)$: 396.2407; Found: 396.2407.

Methyl 3-(quinolin-8-ylcarbamoyl)-10-(triisopropylsilyl)dec-9-ynoate (6j)

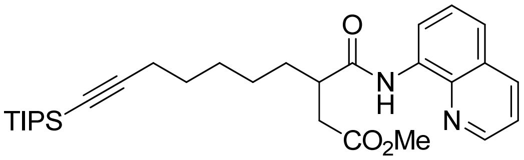

The title compound **6j** was prepared according to **GP3** and was purified by column chromatography (petroleum ether : ethyl acetate = 7: 1, R_f= 0.5). **6j** was obtained as a colourless oil (45.7 mg, 60%). ^{1}H ^{1}H NMR (400 MHz, $CDCl_3$) δ 9.99 (s, 1H), 8.82 (dd, *J* = 4.2, 1.7 Hz, 1H), 8.78 (dd, *J* = 6.9, 2.1 Hz, 1H), 8.15 (dd, *J* = 8.3, 1.7 Hz, 1H), 7.51 (m, 2H), 7.44 (dd, *J* = 8.3, 4.2 Hz, 1H), 3.66 (s, 3H), 3.02 (m, 1H), 2.93 (dd, *J* = 16.4, 9.2 Hz, 1H), 2.55 (dd, *J* = 16.5, 4.7 Hz, 1H), 2.22 (dd, *J* = 8.5, 4.6 Hz, 2H), 1.84 (m, *J* = 14.8, 7.4 Hz, 1H), 1.61 (m, 1H), 1.45 (m, 6H), 1.00 (m, 21H); ^{13}C NMR (101 MHz, $CDCl_3$) δ 173.4,

172.9, 148.4, 138.6, 136.4, 134.5, 128.1, 127.5, 121.7, 121.7, 116.7, 109.1, 80.3, 52.0, 44.5, 36.8, 33.0, 28.8, 28.7, 26.8, 19.9, 18.7, 11.4; IR (neat): ν 3355, 2938, 2862, 2169, 1738, 1689, 1526, 1484, 1462, 1429 cm^{-1}; HRMS (ESI): calc. for $C_{30}H_{44}N_2O_3Si(M^+)$:508.3116; Found:508.3123.

Methyl 3-ethoxy-4-oxo-4-(quinolin-8-ylamino)butanoate (6k)

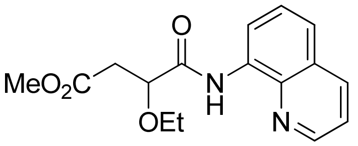

The title compound **6k** was prepared according to **GP3** and was purified by column chromatography (petroleum ether: tetrahydrofuran = 3: 1, R_f = 0.4). **6k** was obtained as a pale while solid (17.6 mg, 39%). ^{1}H NMR (400 MHz, $CDCl_3$) δ 11.04 (s, 1H), 8.84 (dd, J = 4.2, 1.7 Hz, 1H), 8.77 (m, 1H), 8.16 (dd, J = 8.3, 1.7 Hz, 1H), 7.54 (m, 2H), 7.46 (dd, J = 8.3, 4.2 Hz, 1H), 4.44 (dd, J = 9.1, 3.5 Hz, 1H), 3.81 (m, 2H), 3.74 (s, 3H), 3.03 (dd, J = 16.2, 3.5 Hz, 1H), 2.81 (dd, J = 16.2, 9.1 Hz, 1H), 1.42 (t, J = 7.0 Hz, 3H). ^{13}C NMR (101 MHz, $CDCl_3$) δ 171.3, 170.4, 148.7, 139.0, 136.3, 134.0, 128.1, 127.4, 122.2, 121.8, 116.6, 77.9, 67.8, 52.1, 38.5, 15.5; IR (neat): ν 3333, 2925, 2842, 1741, 1686, 1528, 1485, cm^{-1}; HRMS (ESI): calc. for $C_{16}H_{18}N_2O_4(M^+)$: 302.1621; Found: 302.1264.

Methyl 3-(benzyloxy)-4-oxo-4-(quinolin-8-ylamino)butanoate (6l)

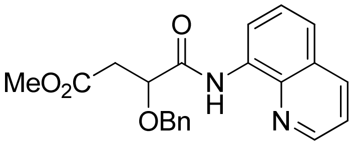

The title compound **6l** was prepared according to **GP3** and was purified by column chromatography (petroleum ether: tetrahydrofuran = 3: 1, R_f = 0.5). **6l** was obtained as a pale while solid (25.1mg, 46%). ^{1}H NMR (400 MHz, $CDCl_3$) δ 11.14 (s, 1H), 8.81 (dd, J = 4.2, 1.6 Hz, 1H), 8.78 (t, J = 4.5 Hz, 1H), 8.17 (dd, J = 8.3, 1.7 Hz, 1H), 7.61 – 7.53 (m, 4H), 7.47 (dd, J = 8.3, 4.2 Hz, 1H), 7.43 – 7.30 (m, 3H), 4.82 (dd, J = 27.3, 11.0 Hz, 2H), 4.68 – 4.54 (m, 1H), 3.72 (s, 3H), 3.08 (dd, J = 16.3, 3.6 Hz, 1H), 2.88 (dd, J = 16.3, 8.8 Hz, 1H); ^{13}C NMR (101 MHz, $CDCl_3$) δ 171.2, 169.9, 148.6, 139.0, 137.0, 136.3, 134.0,

128.7, 128.6, 128.4, 128.1, 127.4, 122.3, 121.8, 116.7, 77.6, 74.2, 52.1, 38.4; IR (neat): ν 3335, 2950, 2852, 1738, 1684, 1526, 1485, 1457, 1431 cm^{-1}; HRMS (ESI): calc. for $C_{21}H_{20}N_2O_4(M^+)$: 364.1418; Found: 364.1418.

Methyl 3-(2-chlorobenzyl)-4-oxo-4-(quinolin-8-ylamino)butanoate (6m)

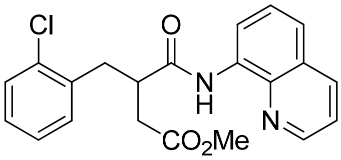

The title compound **6m** was prepared according to **GP3** and was purified by column chromatography (petroleum ether : ethyl acetate = 6: 1, R_f = 0.5). **6m** was obtained as a colourless oil (35.5 mg, 62%). ^1H NMR (400 MHz, $CDCl_3$) δ 9.92 (s, 1H), 8.74 (m, 2H), 8.11 (dd, *J* = 8.3, 1.4 Hz, 1H), 7.49 (m, 2H), 7.41 (dd, *J* = 8.3, 4.2 Hz, 1H), 7.35 (m, 1H), 7.25 (m, 1H), 7.10 (m, 2H), 3.62 (s, 3H), 3.42 (m, 1H), 3.28 (dd, *J* = 13.5, 6.9 Hz, 1H), 3.02 (m, 2H), 2.55 (dd, *J* = 16.9, 4.4 Hz, 1H); ^{13}C NMR (101 MHz, $CDCl_3$) δ 172.5, 172.4, 148.3, 138.5, 136.3, 136.2, 134.5, 134.4, 131.7, 129.9, 128.4, 127.9, 127.4, 127.0, 121.7, 121.7, 116.6, 52.0, 44.3, 36.9, 35.9; IR (neat): ν 3343, 2950, 2862, 1735, 1686, 1526, 1482, 1430, cm^{-1}; IR (neat): HRMS (ESI): calc. for $C_{21}H_{19}N_2O_3Cl(M^+)$: 382.1079; Found: 382.1082.

Methyl 3-(2-chlorophenyl)-4-oxo-4-(quinolin-8-ylamino)butanoate (6n)

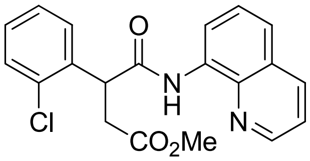

The title compound **6n** was prepared according to **GP3** and was purified by column chromatography (petroleum ether : ethyl acetate = 5: 1, R_f = 0.4). **6n** was obtained as a white solid (23.1mg, 42%). ^1H NMR (400 MHz, $CDCl_3$) δ 10.08 (s, 1H), 8.73 (m, 2H), 8.09 (dd, *J* = 8.3, 1.2 Hz, 1H), 7.48 (m, 4H), 7.39 (dd, *J* = 8.3, 4.2 Hz, 1H), 7.24 (m, 2H), 4.90 (dd, *J* = 9.6, 5.1 Hz, 1H), 3.69 (s, 3H), 3.44 (dd, *J* = 16.9, 9.6 Hz, 1H), 2.80 (dd, *J* = 16.9, 5.1 Hz, 1H); ^{13}C NMR (101 MHz, $CDCl_3$) δ 172.21, 170.00, 148.39, 138.53, 136.22, 134.63, 133.87, 130.13, 129.05, 127.96, 127.75, 127.32, 121.77, 121.70, 116.56, 52.09,

46.00, 36.49; IR (neat): ν 3345, 2953, 2872, 1735, 1688, 1525, 1489, 1450 cm^{-1}; HRMS (ESI): calc. for $C_{20}H_{17}N_2O_3Cl(M^+)$: 368.0922; Found: 368.0927.

Methyl 4-oxo-4-(quinolin-8-ylamino)-3-(m-tolyl)butanoate (6o)

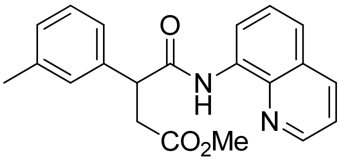

The title compound **6o** was prepared according to **GP3** and was purified by column chromatography (petroleum ether : ethyl acetate = 4: 1, R_f = 0.5). **6o** was obtained as a colourless oil (18.2 mg, 35%). ^{1}H NMR (400 MHz, $CDCl_3$) δ 9.86 (s, 1H), 8.72 (dd, *J* = 7.4, 1.5 Hz, 1H), 8.66 (dd, *J* = 4.2, 1.6 Hz, 1H), 8.08 (dd, *J* = 8.3, 1.6 Hz, 1H), 7.46 (m, 3H), 7.37 (dd, *J* = 8.3, 4.2 Hz, 1H), 7.22 (m, 3H), 4.61 (dd, *J* = 8.9, 5.7 Hz, 1H), 3.69 (s, 3H), 3.45 (dd, *J* = 16.9, 8.9 Hz, 1H), 2.75 (dd, *J* = 16.9, 5.7 Hz, 1H), 2.59 (s, 3H); ^{13}C NMR (101 MHz, $CDCl_3$) δ 172.7, 171.1, 148.3, 138.4, 136.9, 136.3, 136.1, 134.6, 131.2, 127.9, 127.8, 127.6, 127.4, 127.0, 121.6, 121.6, 116.4, 52.0, 46.0, 37.1, 20.1; IR (neat): ν 3346, 2951, 2870, 1737, 1688, 1527, 1485, 1428, cm^{-1}; HRMS (ESI): calc. for $C_{21}H_{20}N_2O_3(M^+)$: 348.1468; Found: 348.1473.

2.5 Synthetic Potential

2.5.1. Gram-scale Synthesis of Chiral *β*-Ester *α*-Amino Acid

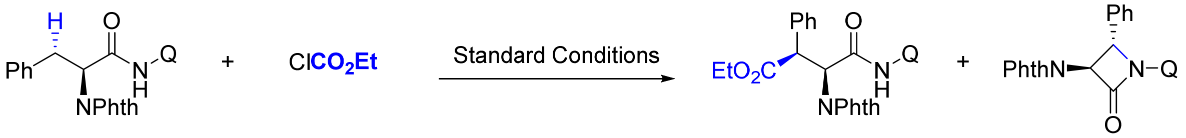

1a (3 mmol)
99% ee

2a, 3.0 equiv.

3a, 74%, 1.10 g
99% ee

3aa, 12%, 0.15 g

Compound **3a** was prepared according to **GP3** in 3.0-mmol scale and was purified by column chromatography (petroleum ether: dichloromethane: ethyl acetate = 7: 1: 2). **3a** was obtained as a pale yellow solid (1.10 g, 74%), with the byproduct **3aa** (0.15 g, 12%).

2.5.2. Removal of Directing Group

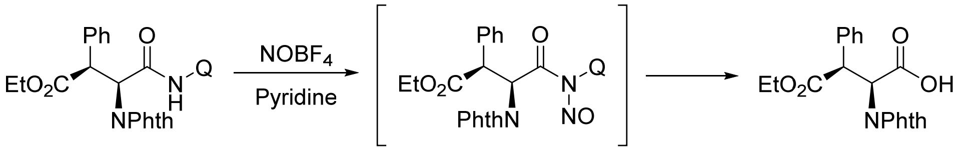

3a, 99% ee **7**, 62%

To a 50 mL Schlenk tube were added **3a** (49.4 mg, 0.1mmol) and pyridine (2.0 mL). $NOBF_4$ (116.8 mg, 1.0 mmol) was added to the solution in one portion and the resulting mixture was stirred for 3 hours at -30 °C then 16 hours at room temperature. The reaction was quenched with an aqueous solution of HCl (6.0 M) until the pH was around 1, then the mixture was extracted with ethyl acetate three times. The combined organic layer was washed by aqueous HCl (1.0 M) twice, dried over $MgSO_4$. Evaporation of the solvent under vaccum and further purification by column chromatography (DCM: MeOH: AcOH = 100: 10: 1, R_f= 0.3) gave the compound **7** (22.6 mg, 62 %) as a slightly yellow oil. $[\alpha]^{20}_D$ = -219.3 (1.0 M in $CHCl_3$); ^{1}H NMR (400 MHz, $CDCl_3$) : δ 9.97 (s, 1H), 7.84 – 7.66 (m, 2H), 7.67 – 7.53 (m, 2H), 7.29 – 7.22 (m, 2H), 7.19 – 7.07 (m, 3H), 5.83 (d, *J* = 11.4 Hz, 1H), 4.66 (d, *J* = 11.4 Hz, 1H), 4.31 – 4.21 (m, 1H), 4.17 – 4.05 (m, 1H), 1.21 (t, *J* = 7.1 Hz, 3H); ^{13}C NMR (101 MHz, $CDCl_3$) : δ 174.2, 171.5, 167.1, 134.3, 133.9, 131.2, 128.8, 128.4, 128.3, 123.7, 61.7, 52.8, 50.5, 14.0; IR (neat): ν 2925, 2751, 1775, 1717, 1684, 1461 cm^{-1}; HRMS (ESI): calc. for $C_{20}H_{18}NO_6(M+H^+)$: 368.1129; Found: 368.1125.

2.5.3. Esterification of Chiral *β*-Ester *α*-Amino Acid

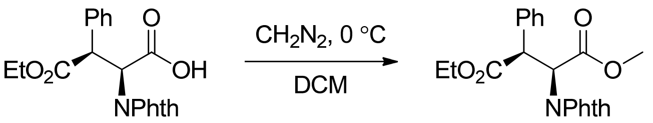

7 **8**, 91%, 97% ee

1-Methyl-1-nitrosourea was added in one portion to a stirring mixture of ethyl ether (20 mL) and aqueous KOH (3.00 g in H_2O, 50%) in an ice bath. To the ethyl ether (5 mL) solution of **7** (36.7 mg, 0.1 mmol) were added the light yellow upper layer until the latter solution also turned to the same colour. Evaporation of the solvent and purification by column chromatography (petroleum ether: ethyl acetate = 4: 1, R_f= 0.4). $[\alpha]^{20}_D$ = -183.5 (0.5 M in $CHCl_3$); **8** was obtained as a while solid (34.7 mg, 91%). ^{1}H NMR (400 MHz, $CDCl_3$) : δ 7.75 – 7.68 (m, 1H), 7.67 – 7.62 (m, 1H), 7.26 (dt, *J* = 3.6, 1.9 Hz, 1H), 7.19 – 7.08 (m, 2H),

5.77 (d, J = 11.5 Hz, 1H), 4.67 (d, J = 11.5 Hz, 1H), 4.30 (dq, J = 10.8, 7.1 Hz, 1H), 4.13 (dq, J = 10.8, 7.1 Hz, 1H), 3.74 (s, 2H), 3.74 (s, 1H), 1.26 (t, J = 7.1 Hz, 2H); ^{13}C NMR (101 MHz, $CDCl_3$) δ 171.7, 169.0, 167.1, 134.3, 134.1, 131.3, 128.8, 128.5, 128.2, 123.6, 61.6, 53.2, 53.0, 50.8, 14.2; IR (neat): ν 2926, 2752, 1775, 1722, 1684, 1532, 1494, 1461 cm^{-1}; HRMS (ESI): calc. for $C_{21}H_{20}NO_6$ $(M+H^+)$: 382.1285; Found: 382.1288. HPLC Chiralpak® AD-H column, n-hexane/isopropanol = 70:30, flow rate = 1.0 mL/min, λ = 220 nm, 14.3 (minor), 19.9 (major), 97% ee.

2.6 Mechanistic Investigations

2.6.1 KIE Experiments

(*S*)-*N*-(3,3,3-*tri*D-2-Phthalimidopropionyl)-8-aminoquinoline (6a-*d*$_3$)

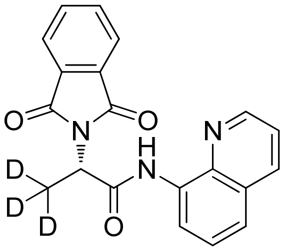

To a 30-mL resealable Schlenk flask was added **6a** (1.38 g, 4.0 mmol), $Pd(OAc)_2$ (90.0 mg, 0.4 mmol), CH_3CO_2D (10mL). The mixture was stirred at 90 °C for 20 hours. This procedure was repeated twice, and the product was purified on flash silica gel chromatography (ethyl acetate : dichloromethane = 1 : 99). The compound **6a-*d*$_3$** was a known compound.[4] ^{1}H NMR (400 MHz, $CDCl_3$) δ 10.32 (s, 1H), 8.72 (dd, J = 5.4, 3.6 Hz, 1H), 8.69 (dd, J = 4.3, 1.7 Hz, 1H), 8.15 (dd, J = 8.3, 1.7 Hz, 1H), 7.90 (dd, J = 5.4, 3.1 Hz, 2H), 7.75 (dd, J = 5.5, 3.1 Hz, 2H), 7.56 – 7.48 (m, 2H), 7.42 (dd, J = 8.3, 4.2 Hz, 1H), 5.25 (s, 1H).

(*S*)-*N*-(3,3-*di*D-3-Phenyl-2-phthalimidopropionyl)-8-aminoquinoline (1a-*d*$_2$)

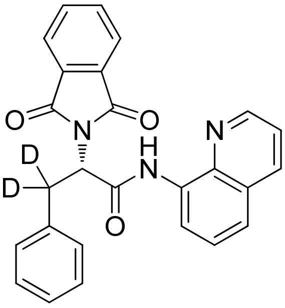

To a 100-mL vial was added **6a-*d3*** (348.6 mg, 1.0 mmol), $Pd(OAc)_2$ (22.4 mg, 0.1 mmol, 10 mol%), PhI (244.8 mg, 1.2 mmol, 1.2 eq), $AgBF_4$ (253.0 mg, 1.3 mmol, 1.3 eq), and *t*-BuOH (10 mL). The mixture was stirred at 90 °C for 14 hours. After cooling to room temperature, the reaction was diluted with dichloromethane (10 mL) and triethylamine (2.0 mL) was added to the mixture. After the mixture was maintained for 6 hours, it was then filtered through a pad of Celite and washed by dichloromethane (30 mL). The filtrate was washed by water (15 mL), and the aqueous phase was extracted with dichloromethane (2 × 20 mL). The combined organic phase was then washed by brine (15 mL), and dried over anhydrous $MgSO_4$. Evaporation of organic solvent and purification by column chromatography in petroleum ether: dichloromethane: ethyl acetate = 6:3:1 gave the corresponding product **1a-*d2***. ^{1}H NMR (400 MHz, $CDCl_3$) δ 10.33 (s, 1H), 8.74 (dd, *J* = 6.2, 2.8 Hz, 1H), 8.61 (dd, *J* = 4.3, 1.6 Hz, 1H), 8.13 (dd, *J* = 8.3, 1.7 Hz, 1H), 7.83 (dd, *J* = 5.5, 3.1 Hz, 2H), 7.71 (dd, *J* = 5.5, 3.1 Hz, 2H), 7.56 – 7.49 (m, 2H), 7.40 (dd, *J* = 8.3, 4.2 Hz, 1H), 7.29 (d, *J* = 7.1 Hz, 2H), 7.23 (t, *J* = 7.3 Hz, 2H), 7.16 (t, *J* = 7.2 Hz, 1H), 5.44 (s, 1H). HRMS (ESI): calc. for $C_{26}H_{18}D_2N_3O_3M+H^+$): 424.1625; Found: 424.1637.

KIE studies:

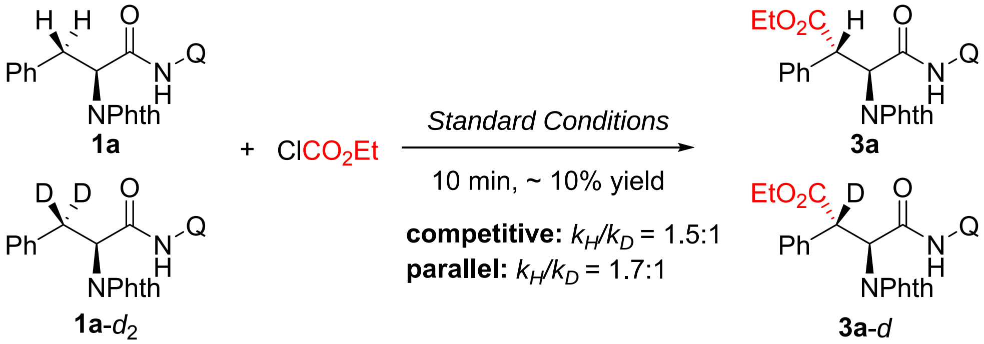

For competitive reaction, to a 50 mL Schlenk tube were added **1a** (31.6 mg, 0.075 mmol), **1a**-*d*2 (31.6 mg, 0.075 mmol), Ag_2CO_3 (82.7 mg, 0.3 mmol), I_2 (38.0 mg, 0.15 mmol), $ClCO_2Et$ (0.45 mmol, 45.0 µL), Toluene (2.0 mL). The tube was sealed under air. The mixture was stirred at room temperature for 5 minutes then heated at 120 °C for 10 minutes. After cooling to room temperature, the reaction mixture was diluted with EtOAc (10 mL) and filtered through a short pad of Celite. After concentration, the crude product was added CH_2Br_2 as internal standard and characterized by ^1H NMR.

For parallel reaction, to a 50 mL Schlenk tube were added **1a** (31.6 mg, 0.075 mmol), Ag_2CO_3 (41.4 mg, 0.15 mmol), I_2 (19.0 mg, 0.075 mmol), $ClCO_2Et$ (0.225 mmol, 22.5.0 µL), toluene (1.0 mL). The tube was sealed under air; To another 50 mL Schlenk tube were added **1a**-*d*2 (31.6 mg, 0.075 mmol), Ag_2CO_3 (41.4 mg, 0.15 mmol), I_2 (19.0 mg, 0.075 mmol), $ClCO_2Et$ (0.225 mmol, 22.5.0 µL), toluene (1.0 mL). The tube was sealed under air. The mixtures were stirred at room temperature for 5 minutes then heated at 120 °C for 10 minutes. After cooling to room temperature, the reaction mixtures were diluted with EtOAc (5 mL) and filtered through a short pad of Celite. After concentration, the crude products were added CH_2Br_2 as internal standard and tested ^1H NMR. The yields of the target products were both calculated according to ^1H NMR.

2.6.2 Stoichiometric Reactivity of Complex I

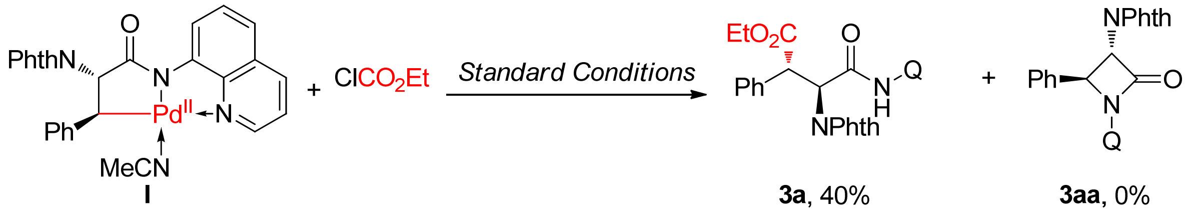

WITH Ag_2CO_3: To a 50 mL Schlenk tube were added **I** (28.4 mg, 0.05 mmol), Ag_2CO_3 (27.3 mg, 0.1 mmol), I_2 (13.0 mg, 0.05 mmol), $ClCO_2Et$ (0.15 mmol, 14.5 µL), toluene (1.0 mL). The tube was sealed under air. The mixture was stirred at room temperature for 5 minutes then heated at 120 °C for 16 h. After cooling to room temperature, the reaction mixture was diluted with EtOAc (5 mL) and filtered through a short pad of Celite. After concentration in vacuo, the crude reaction mixture was purified by silica gel

column chromatography (petroleum ether: dichloromethane: ethyl acetate = 7: 1: 2). **3a** was obtained as a pale yellow solid (9.7 mg, 40%) and no **3aa** was observed.

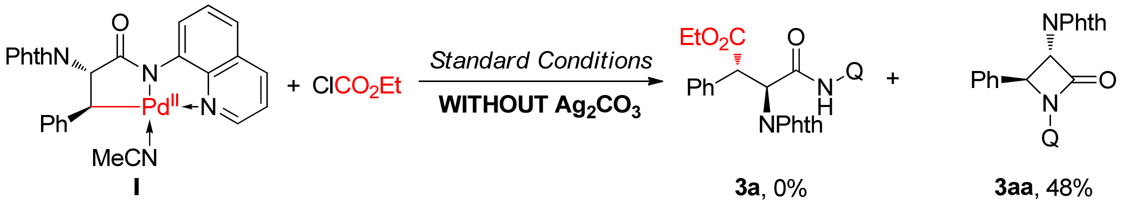

WITHOUT Ag_2CO_3: To a 50 mL Schlenk tube were added **I** (42.6 mg, 0.075 mmol) and I_2 (19.0 mg, 0.075 mmol), $ClCO_2Et$ (0.225 mmol, 22.7 μL), toluene (1.0 mL). The tube was sealed under air. The mixture was stirred at room temperature for 5 minutes then heated at 120 °C for 16 h. After cooling to room temperature, the reaction mixture was diluted with EtOAc (5 mL) and filtered through a short pad of Celite. After concentration in vacuo, the crude reaction mixture was characterized by ^1H NMR and no desired product **3a** was observed. **3aa** was purified on flash silica gel chromatography (petroleum ether: dichloromethane: ethyl acetate = 7: 1: 2). **3aa** was obtained as a pale yellow solid (15.0 mg, 48%).

2.6.3 Catalytic Reactivity of Complex I

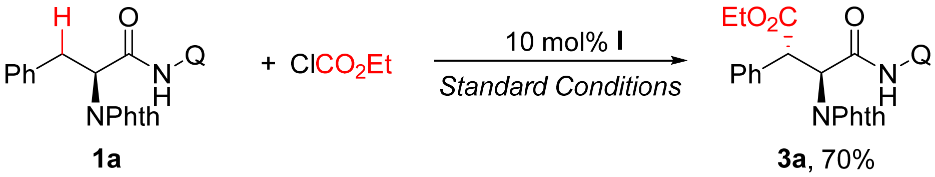

To a 50 mL Schlenk tube were added **1a** (63.2 mg, 0.15 mmol), **I** (8.5 mg, 0.015 mmol), Ag_2CO_3 (82.7 mg, 0.3 mmol, I_2 (38.0 mg, 0.15 mmol), $ClCO_2Et$ (0.45 mmol, 45.0 μL), Toluene (2.0 mL). The tube was sealed under air. The mixture was stirred at room temperature for 5 minutes then heated at 120 °C for 16 h. After cooling to room temperature, the reaction mixture was diluted with EtOAc (10 mL) and filtered through a short pad of Celite. After concentration in vacuo, the crude reaction mixture was purified by silica gel column chromatography (petroleum ether: dichloromethane: ethyl acetate = 7: 1: 2). **3a** was obtained as a pale yellow solid (57.2 mg, 70%).

Supplementary References

[1] Zhang, Q.; Yin, X.-S.; Zhao, S.; Fang, S.-L.; Shi, B.-F. *Chem. Commun*. **2014**, *50*, 8353.

[2] Zhang, Q.; Chen, K.; Rao, W.; Zhang, Y.; Chen, F. -J.; Shi, B. -F. *Angew. Chem. Int. Ed*. **2013**, *52*, 13588.

[3] Zhao, C. -J.; Xue, D.; Jia, Z. -H.; Wang, C; Xiao, J. *Synlett*. **2014**, *25*, 1577.

[4] Wang, B.; Nack, W. A.; He, G.; Zhang, S. -Y.; Chen, G. *Chem. Sci.* **2014**, *5*, 3952.

[5] Chen, K.; Shi, B. -F. *Angew. Chem. Int. Ed*. **2014**, *53*, 11950.

[6] Rao, W.-H.; Zhan, B.-B.; Chen, K.; Ling, P.-X.; Zhang, Z.-Z; Shi, B.-F. *Org. Lett.***2015**, *17*, 3552.

[7] Barry, C. S.; Cocinero, E. J.; Çarçabal, P.; Gamblin, D. P.; Stanca-Kaposta, E. C.; Remmert, S. M.; Fernández-Alonso, M. C.; Rudić, S.; Simons, J. P.; Davis, B. G. *J. Am. Chem. Soc.* **2013**, *135*, 16895.

[8] Makino, K.; Goto, T.; Ohtaka, J.; Hamada, Y. *Heterocycles*. **2009**, *77*, 629.
